# Anticancer Evaluation of Novel Benzofuran–Indole Hybrids as Epidermal Growth Factor Receptor Inhibitors against Non-Small-Cell Lung Cancer Cells

**DOI:** 10.3390/ph17020231

**Published:** 2024-02-09

**Authors:** Yechan Lee, Sunhee Lee, Younho Lee, Doona Song, So-Hyeon Park, Jieun Kim, Wan Namkung, Ikyon Kim

**Affiliations:** 1 College of Pharmacy and Yonsei Institute of Pharmaceutical Sciences, Yonsei University, 85 Songdogwahak-ro, Yeonsu-gu, Incheon 21983, Republic of Korea; llyycc94@naver.com (Y.L.); lovelysh.94@hanmail.net (S.L.); zz265zz@hanmail.net (Y.L.); sohyeon0605@yonsei.ac.kr (S.-H.P.); jieunkimx@yonsei.ac.kr (J.K.); 2 Department of Biotechnology, College of Life Science and Biotechnology, Yonsei University, Seoul 03722, Republic of Korea; doona.s@yonsei.ac.kr

**Keywords:** benzofuran, indole, hybrid structure, NSCLC, EGFR

## Abstract

The epidermal growth factor receptor (EGFR), also known as ErbB1 and HER1, belongs to the receptor tyrosine kinase family. EGFR serves as the primary driver in non-small-cell lung cancer (NSCLC) and is a promising therapeutic target for NSCLC. In this study, we synthesized a novel chemical library based on a benzofuran–indole hybrid scaffold and identified **8aa** as a potent and selective EGFR inhibitor. Interestingly, **8aa** not only showed selective anticancer effects against NSCLC cell lines, PC9, and A549, but it also showed significant inhibitory effects against the double mutant L858R/T790M EGFR, which frequently occurs in NSCLC. In addition, in PC9 and A549 cells, **8aa** potently blocked the EGFR signaling pathway, cell viability, and cell migration. These findings suggest that **8aa**, a benzofuran–indole hybrid derivative, is a novel EGFR inhibitor that may be a potential candidate for the treatment of NSCLC patients with EGFR mutations.

## 1. Introduction

**Figure 1:**
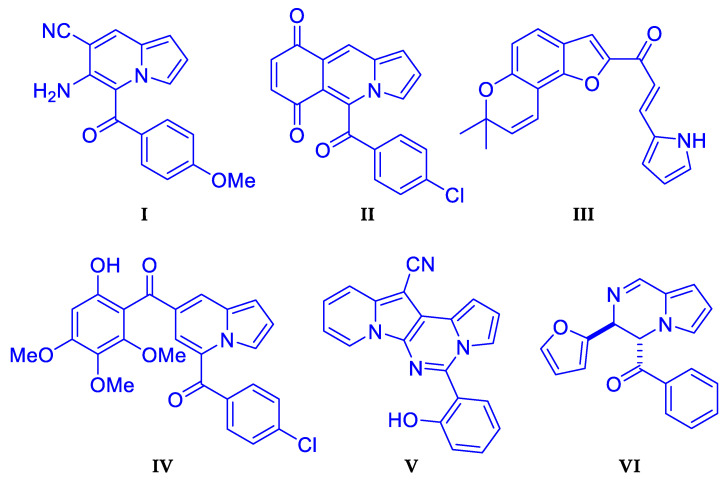
Bioactive heterocycles developed in our laboratory.

The identification of new chemical entities with pharmacologically modulating properties is important in the early stages of drug discovery processes. Accordingly, the generation of new drug-like chemical scaffolds and their derivatives for biological screening is highly desired. Against this backdrop, we were able to find several chemical motifs **(I–VI)** with significant pharmacological functions through the synthesis and biological evaluation of novel heterocycles (Figure 1) [1,2,3].

Lung cancer is the most the common cause of cancer-related death worldwide, with a 5-year patient survival rate of less than 15%. Non-small-cell lung cancer (NSCLC), which is commonly found in lung cancer, accounts for 85% of lung cancer cases [4,5].

The epidermal growth factor receptor (EGFR) belongs to the receptor tyrosine kinase family, which is highly expressed in NSCLC patients [6]. Given the pivotal role of the EGFR signaling pathway in regulating tumorigenesis, cell growth, and proliferation in NSCLC, the EGFR emerges as an attractive therapeutic target [7]. For example, EGFR overexpression or mutation has been demonstrated in 43–89% of NSCLC patients [8]. In addition, it has been observed that 25% of NSCLC patients exhibited mutations in the EGFR tyrosine kinase domain, with 75% of these mutations being associated with overexpression of EGFR [9]. EGFR overexpression or abnormalities trigger sustained signal transduction, promoting cell survival, proliferation, relapse, tumorigenesis, and metastasis in NSCLC through the MAPK, PI3K/AKT, and signal transducer and activator of transcription (STAT) factors [10,11]. To date, clinically available EGFR inhibitors comprise EGFR tyrosine kinase inhibitors (TKIs) like erlotinib, gefitinib, afatinib, and osimertinib, and monoclonal antibodies (mAbs) such as panitumumab and cetuximab [12]. However, despite initial robust responses to first- and second-generation EGFR-TKIs, a considerable number of NSCLC patients develop acquired resistance during EGFR-TKI treatment within 9 to 14 months after starting treatment [13]. Therefore, there remains a necessity for the development of novel EGFR inhibitors to address drug resistance in the treatment of NSCLC.

**Figure 2:**
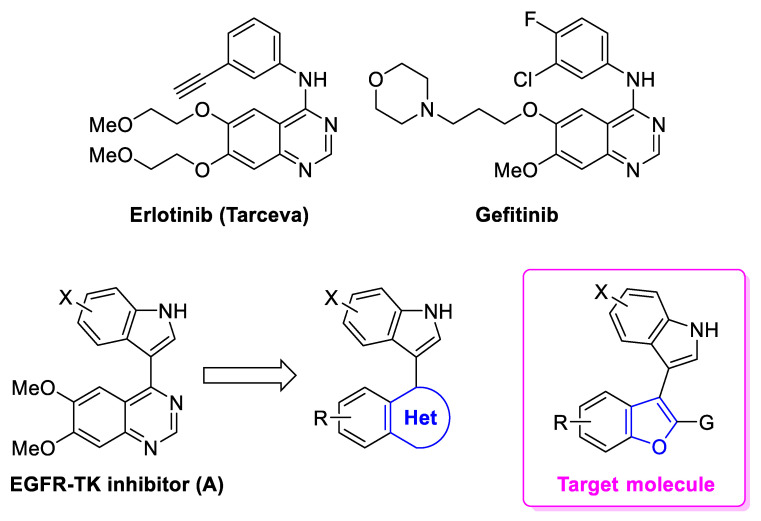
Design for developing new EGFR inhibitors.

Erlotinib and gefitinib are two representative EGFR-TKIs with a 4-anilinoquinazoline skeleton (Figure 2). In 2008, Lüth and Löwe reported the synthesis of quinazoline–indole hybrids **(A)** which were found to exhibit EGFR inhibitory activity [14]. In connection with our continued interest in the design and synthesis of new anticancer agents [15,16], we hoped to find a new heterocyclic skeleton to replace the quinazoline moiety while retaining the indole group. Along this line, we wondered whether benzofuran could be used instead of quinazoline.

**Figure 3:**
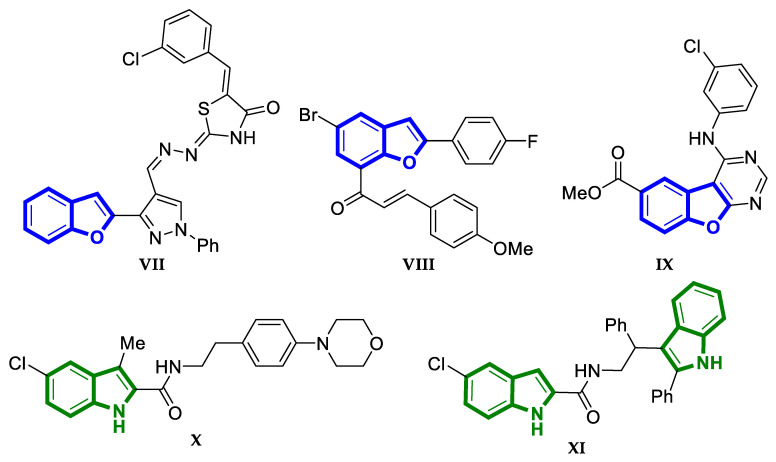
Benzofuran and indole EGFR inhibitors.

Benzofuran has been employed as a key pharmacophore of a number of small molecules with biological activities, such as anti-inflammatory, antimicrobial, antifungal, antioxidant, antiviral, and antitumor properties [17]. As an another important privileged structure, indole constitutes a core skeleton in many bioactive natural products and pharmaceuticals [18]. Although several benzofuran- or indole-based EGFR inhibitors **(VII–XI)** have been discussed in the literature (Figure 3) [19,20,21,22,23], no chemical scaffolds consisting of both benzofuran and indole have been reported as EGFR inhibitors. Here, we wish to describe the modular synthesis and biological evaluation of benzofuran–indole hybrids as a new class of highly promising EGFR inhibitors.

**Scheme 1:**
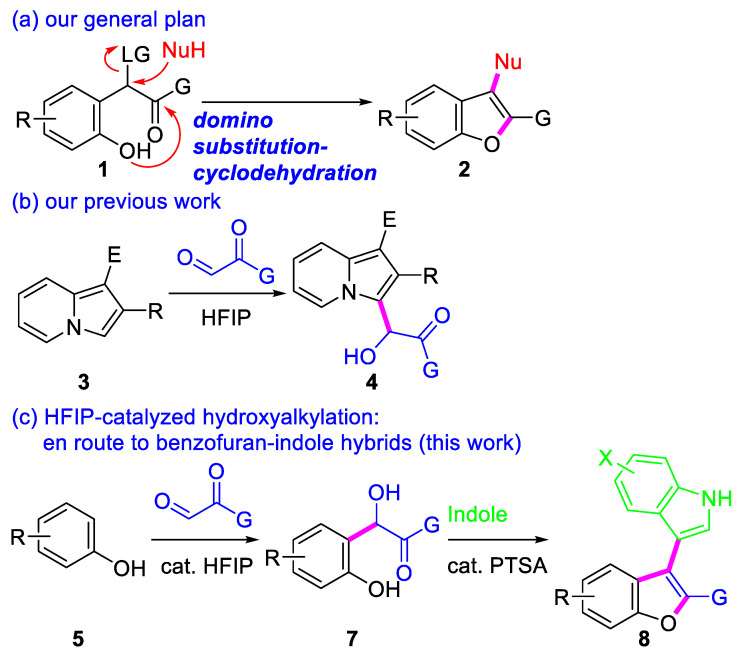
Synthetic plans.

As part of our general plan to make poly-functionalized benzofurans, a domino nucleophilic substitution–dehydrative cyclization procedure with **1** was deemed to give 2,3-disubstituted benzofuran **2** (Scheme 1a). To validate our hypothesis, the requisite starting material **1** (when LG is OH) was envisioned to be easily prepared via a Friedel–Crafts-type reaction between phenol and arylglyoxal [24]. Inspired by our recent success in achieving the hexafluoroisopropanol (HFIP)-mediated hydroxyalkylation of indolizine **3** to arylglyoxal to afford **4** (Scheme 1b) [25], we expected that a HFIP-promoted Friedel–Crafts-type reaction between phenol **5** and arylglyoxal would give rise to **7**, which could be converted to benzofuran **8**, having an indole at the C3 position upon exposure to indole and *p*-toluenesulfonic acid (PTSA) (Scheme 1c). The biological investigation of benzofuran–indole hybrid **8** [26] revealed that this class of compounds exhibit anticancer activity against PC9 and A549 lung cancer cells via the inhibition of phosphorylated EGFR. Here, we wish to describe our findings along this line.

## 2. Results and Discussion

### 2.1. Design and Synthesis of Benzofuran–Indole Hybrids

**Scheme 2:**
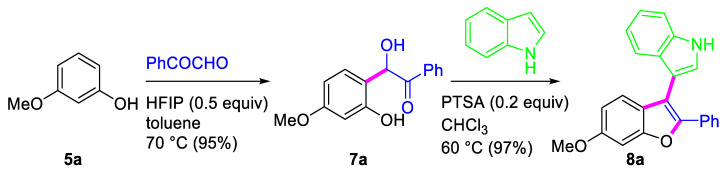
Synthesis of **8a** *^a,b^. ^a^* A mixture of 5a (2 equiv), phenylglyoxal (0.33 mmol, 1 equiv), and HFIP (0.5 equiv) in toluene (4 mL) was stirred at 70 °C for 36 h. A mixture of **7a** (0.08 mmol, 1 equiv), indole (1.5 equiv), and PTSA (0.2 equiv) in CHCl_3_ (2 mL) was stirred at 60 °C for 16 h. *^b^* Isolated yield (%).

When we reacted **5a** (2 equiv) with phenylglyoxal (1 equiv) in the presence of HFIP (0.5 equiv) in toluene at 70 °C, the desired product **7a** was isolated in 95% yield (Scheme 2). The subsequent treatment of **7a** with indole and PTSA (0.2 equiv) in CHCl_3_ at 60 °C provided benzofuran possessing an indole at the C3 site in 97% yield.

Having found the optimal conditions for the synthesis of **7** and **8**, we examined the reaction scope with several phenols, (hetero)arylglyoxals, and indoles (Table 1). In general, these two-step sequences allowed for a variety of 2-arylbenzofurans **8** bearing an indole at the C3 site via intermediates of **7** in good to excellent yields. Various functional groups, such as alkoxy, alkyl, and halogen, were well tolerated under these conditions.

**Figure 4:**
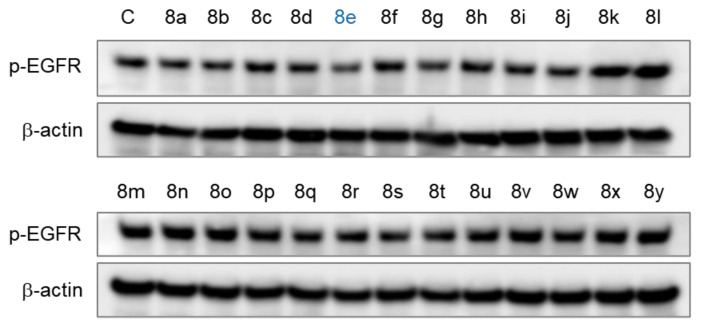
**The inhibitory effects of 8a–y on the EGFR in PC9 cells.** PC9 cells were pretreated with 10 μM of **8a–y** for 6 h, and then the cells were treated with EGF (20 ng/mL) for 30 min. The expression levels of p-EGFR were observed by immunoblot analysis.

An immunoblot analysis of these compounds indicated that **8e** significantly inhibited the phosphorylation of the EGFR (Figure 4). In addition, **8g** showed a weak ability to reduce p-EGFR levels. The cytotoxicity of these benzofuran–indole hybrids **8** against lung cancer cell lines was evaluated in PC9 cells (Table 2). Consistent with the immunoblot analysis results, both **8e** and **8g** showed potent cytotoxicity in PC9 cells.

**Figure 5:**
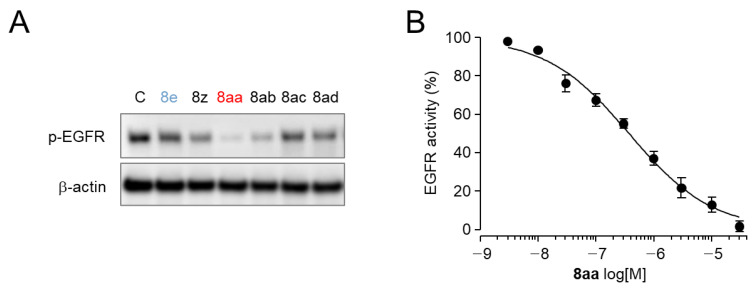
**The inhibitory effects of 8e and 8z–ad on the phosphorylation of EGFR in PC9 cells.** (**A**) PC9 cells were pretreated with 10 μM of **8e** and **8z–ad** for 6 h, and then the cells were treated with EGF (20 ng/mL) for 30 min. The expression levels of p-EGFR were observed by immunoblot analysis. (**B**) The kinase inhibitory activity of **8aa** on the EGFR was assessed using an EGFR kinase assay kit.

As **8e** showed promising anticancer activity, more close analogs (**8z–ad**) were synthesized for secondary screening. Our immunoblot analysis showed that **8aa** reduced p-EGFR more than **8e**, and **8aa** inhibited EGFR kinase activity with IC_50_ values of 0.44 ± 0.02 µM (Figure 5). The cytotoxic activities of these derivatives against the lung cancer cell lines (PC9 and A549) indicated that **8aa** exhibited the most remarkable cytotoxicity among the derivatives, with IC_50_ values of 0.32 ± 0.05 µM and 0.89 ± 0.10 µM, respectively (Table 3). To identify whether **8aa** is a potent EGFR inhibitor, further studies were conducted.

### 2.2. Inhibitory Effect of ***8aa*** on EGFR Signaling Pathways in PC9 and A549 Cells

**Figure 6:**
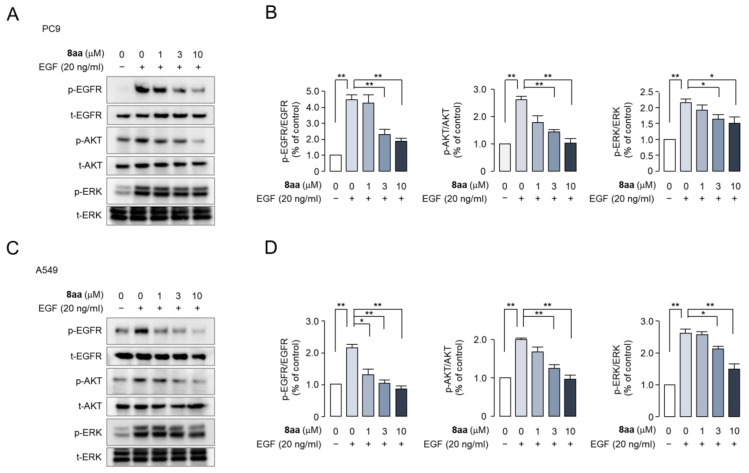
**The inhibitory effects of 8aa on the EGFR signaling pathways in PC9 and A549 cells.** (**A**,**C**) PC9 and A549 cells were pretreated with **8aa** at the indicated concentrations for 6 h, and then the cells were incubated with EGF (20 ng/mL) for 30 min. The expression levels of p-EGFR, t-EGFR, p-AKT, t-AKT, p-ERK1/2, and t-ERK1/2 were measured by immunoblotting. (**B**,**D**) p-EGFR, p-AKT, and p-ERK1/2 protein intensities were normalized to t-EGFR, t-AKT, and t-ERK1/2, respectively (mean ± S.E., *n* = 3). * *p* < 0.05; ** *p* < 0.01.

Previous studies have reported that the upregulation of the EGFR occurs frequently in NSCLC, and the EGFR plays an important role in the development and progression of NSCLC [27,28]. To investigate the effects of **8aa** on multiple EGFR-mediated signaling pathways, we performed an immunoblot analysis on the EGF-induced phosphorylation of the EGFR, AKT, and ERK1/2 in NSCLC cell lines, namely PC9 and A549 cells. As shown in Figure 6, EGF strongly increased the phosphorylation of EGFR, and **8aa** significantly reduced the EGF-induced phosphorylation of the EGFR in a dose-dependent manner. In addition, **8aa** also reduced the phosphorylation of AKT and ERK1/2, downstream signaling pathways of the EGFR. These results indicated that **8aa** can effectively block the signal transduction pathway through EGFR phosphorylation.

### 2.3. Molecular Modeling of ***8aa***

**Figure 7:**
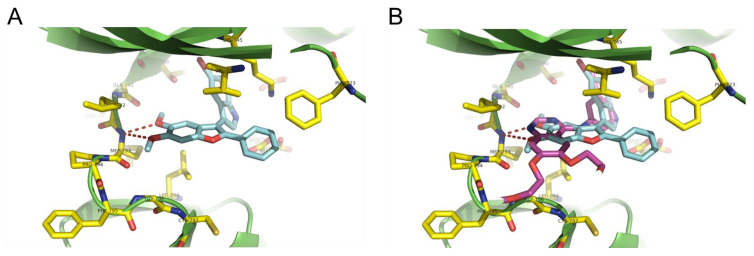
**Binding mode of 8aa with the EGFR.** (**A**) A structural simulation of the **8aa**-EGFR complex showed that some residues (yellow stick) were involved in binding with **8aa** (cyan—carbon), including non-bonded interactions (Phe723, Val726, Met677, and Leu844) and red dot hydrogen bonds (Met793). (**B**) A super-imposed model of the co-crystal structure (1M17.pdb) of **8aa** and erlotinib (magenta—carbon).

To elucidate the underlying mechanism driving the preferential binding of **8aa** to the active conformation of EGFR-TKD, molecular docking studies were carried out using the tyrosine kinase domain of the EGFR (PDB ID: 1M17), which provided the initial erlotinib conformation [29]. The binding mode of **8aa** to the EGFR is depicted in Figure 7, showing its possible molecular interactions. The methoxy oxygens at the C5 and C6 positions of **8aa** form hydrogen bond interactions with the kinase hinge that is an amide backbone of Met793 (Figure 7). The benzofuran moiety within **8aa**, situated at the core, maintains hydrophobic interactions with Val726 and Leu844. In addition, the phenyl group at the C1 site of benzofuran **8aa** is shown to have a π-π interaction with Phe723. Based on these results, compound **8aa** induced the intended mechanism of action by conserving the overall interaction with the tyrosine kinase domain of the EGFR.

### 2.4. Effect of ***8aa*** on Cell Viability in PC9, A549, MCF7, HepG2, PC3, HT29, HaCaT, and HEK293T Cells

**Figure 8:**
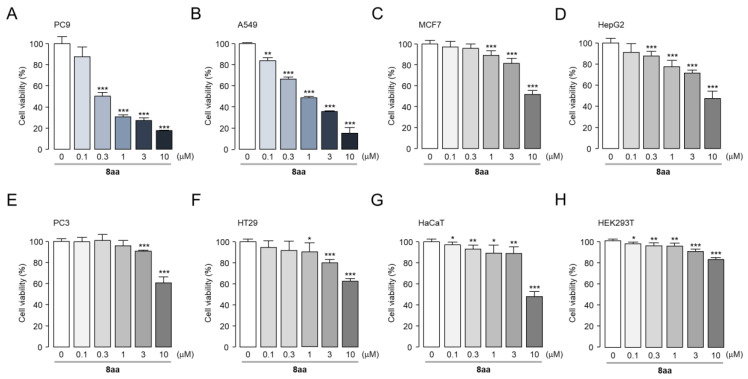
**Effects of 8aa on cell viability in PC9, A549, MCF7, HepG2, PC3, HT29, HaCaT, and HEK293T cells**. (**A**–**H**) PC9, A549, MCF7, HepG2, PC3, HT29, HaCaT, and HEK293T cells were treated with **8aa** at the indicated concentrations for 72 h, and the medium was changed every 24 h with newly added **8aa**. Cell viability was estimated with the MTS assay (mean ± S.D., *n* = 6). * *p* < 0.05; ** *p* < 0.01; *** *p* < 0.001.

To investigate whether **8aa** shows selective cytotoxicity on lung cancer cells, we performed cell proliferation assays on PC9 and A549 non-small-cell lung adenocarcinoma, MCF7 breast adenocarcinoma, HepG2 hepatocellular carcinoma, PC3 prostate adenocarcinoma, HT29 colorectal adenocarcinoma, HaCaT human skin keratinocyte, and HEK293T human embryonic kidney cells. As expected, **8aa** significantly inhibited cell viability in both the PC9 and A549 cells with IC_50_ values of 0.32 ± 0.05 and 0.89 ± 0.10 µM, respectively (Figure 8A,B). Interestingly, **8aa** showed weak inhibitory effects on other cancer cell lines, namely MCF7, HepG2, PC3, and HT29 (Figure 8C–F). In addition, **8aa** weakly reduced cell viability in the non-tumorigenic cell lines, including HaCaT and HEK293T (Figure 8G,H). These findings indicate that **8aa** has the potential to serve as a potent and selective anticancer agent for NSCLC.

### 2.5. ***8aa*** Inhibits Cell Migration in PC9 and A549 Cells

**Figure 9:**
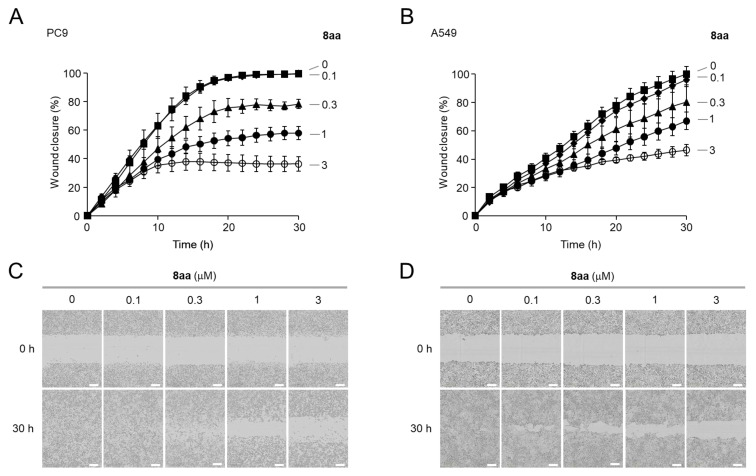
**Effect of 8aa on cell migration in PC9 and A549 cells.** (**A**,**B**) An in vitro wound healing assay was performed on PC9 and A549 cells for 30 h (mean ± S.D., *n* = 3). The PC9 and A549 cells were treated with the indicated concentrations of **8aa**, and time-lapse images were obtained every 2 h after wound infliction. (**C**,**D**) Representative wound images were taken at 0 h and 30 h following the administration of **8aa** at the indicated concentrations. The scale bars represent 300 μm.

To assess the potential effect of **8aa** on NSCLC cell migration, an in vitro wound healing assay was performed using PC9 and A549 cells. Interestingly, **8aa** significantly inhibited cell migration in both the PC9 and A549 cells in a dose-dependent manner. In the PC9 cells, treatment with 0.1, 1, and 3 μM of **8aa** reduced cell migration by 21.6%, 42.0%, and 63.7%, respectively. Similarly, in the A549 cells, exposure to 0.1, 1, and 3 μM of **8aa** inhibited cell migration by 19.7%, 33.0%, and 59.6%, respectively (Figure 9).

### 2.6. ***8aa*** Significantly Induces Apoptosis in PC9 and A549 Cells

**Figure 10:**
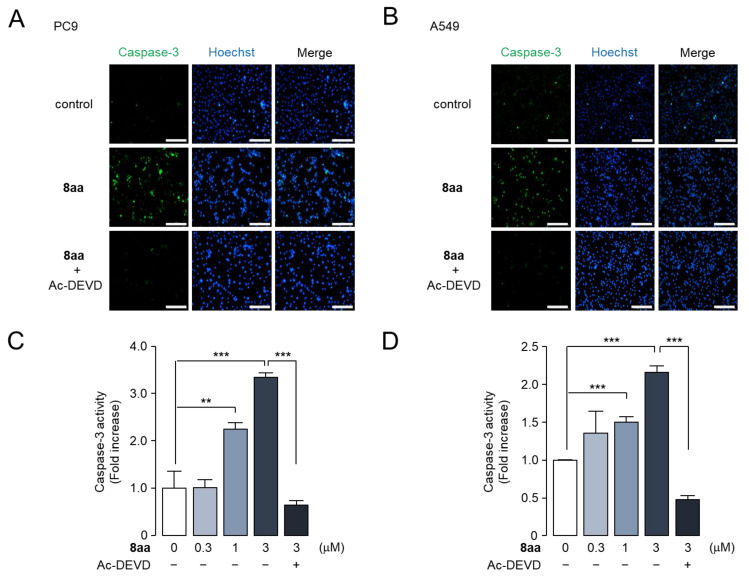

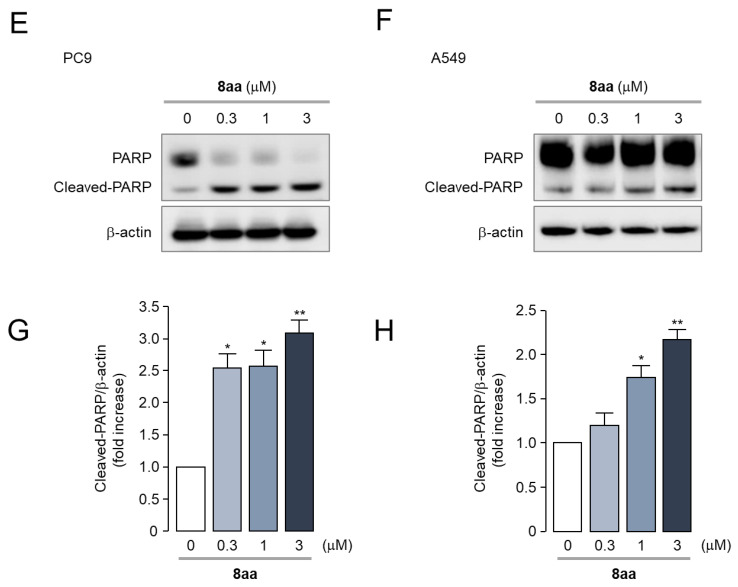
**Effects of 8aa on caspase-3 activity and PARP cleavage in PC9 and A549 cells.** (**A**,**B**) PC9 and A549 cells were treated with 3 µM of **8aa** for 24 h and then incubated with 1 µM of caspase-3 substrate (green) and 1 µM of Hoechst 33342 (blue) for 30 min before image acquisition. The scale bars represent 200 μm. (**C**,**D**) The PC9 and A549 cells were treated with **8aa** at the indicated concentrations for 24 h, and then 1 μM of caspase-3 substrate was treated for 30 min. Caspase-3 activity was inhibited by 20 μM of Ac-DEVD-CHO (mean ± S.D., *n* = 3). (**E**,**F**) The cells were treated with **8aa** at the indicated concentrations for 24 h, and the expression levels of PARP, cleaved-PARP, and β-actin were measured by immunoblotting. (**G**,**H**) Cleaved-PARP protein intensities were normalized to β-actin (mean ± S.E., *n* = 3). * *p* < 0.05; ** *p* < 0.01; *** *p* < 0.001.

The pharmacological inhibition of the EGFR signaling pathway causes apoptosis in various solid tumors [30,31]. To investigate the apoptotic potential of **8aa** in PC9 and A549 cells, we evaluated its influence on caspase-3 activity and PARP cleavage, established markers of apoptotic signaling. Interestingly, caspase-3 activity was significantly increased by **8aa** in the PC9 and A549 cells in a dose-dependent manner, and the increased caspase-3 activity was completely inhibited by AC-DEVD-CHO, a potent caspase-3 inhibitor (Figure 10A–D). In addition, the expression levels of cleaved PARP were significantly increased by **8aa** in both the PC9 and A549 cells in a dose-dependent manner (Figure 10E–H). These results reveal that **8aa** exhibits potent anticancer effects by inducing apoptosis in NSCLC cells.

**Figure 11:**
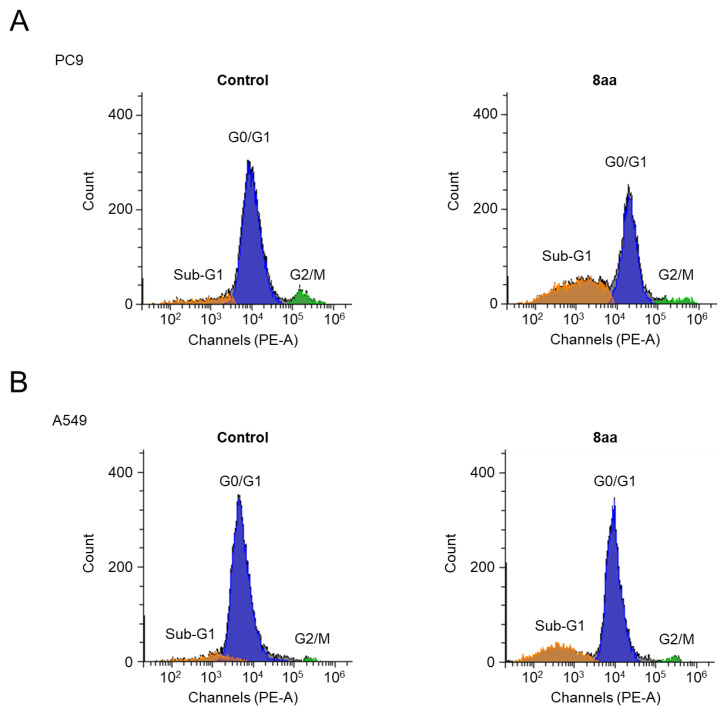
**Effect of 8aa on cell cycles of PC9 and A549 cells.** (**A**,**B**) PC9 and A549 cells were treated with 10 µM of **8aa** for 24 h and then cell cycle phases were estimated by using propidium iodide (PI) staining followed by cell cycle analysis.

To further investigate the effect of **8aa** on the cell cycles of PC9 and A549 cells, we carried out cell cycle analysis using propidium (PI) staining. As shown in Figure 11, **8aa** significantly promoted the ratios in the Sub-G1 (apoptotic peak) phase compared to the control group, but the G2/M phase was not affected by **8aa**. In the case of the **8aa** treatment group, the G0/G1 phase reduced from 73.01% to 47.50% and from 81.64% to 64.24% in the PC9 and A549 cells, respectively. Also, the Sub-G1 phase increased from 8.52% to 35.23% and from 8.48% to 21.40% in the PC9 and A549 cells, respectively. These results suggest that **8aa** significantly induces apoptosis without exerting an effect on cell cycle arrest.

### 2.7. ***8aa*** Potently Inhibits EGFR^L858R/T790M^ in H1975 Cells

**Figure 12:**
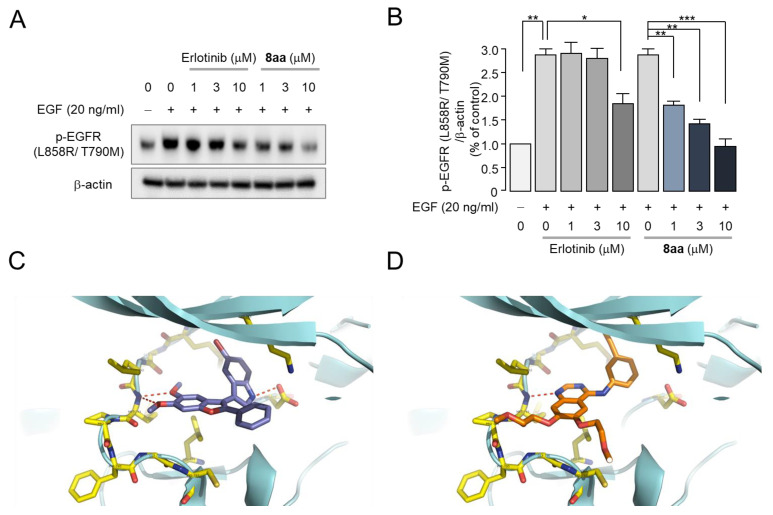
**Effect of erlotinib and 8aa on EGFR^L858R/T790M^.** (**A**) H1975 cells were pretreated with erlotinib and **8aa** at the indicated concentrations for 6 h, and then EGF (20 ng/mL) was treated for 30 min. (**B**) p-EGFR (L858R/T790M) band intensities were normalized to β-actin (mean ± S.E., *n* = 3). (**C**) A structural simulation of the **8aa** complex showed that some residues (yellow stick) were involved in binding with **8aa** (purple—carbon) red dot hydrogen bonds (Met793 and Asp855). (**D**) A docking model of erlotinib (orange—carbon) established by using the co-crystal structures of EGFR^L858R/T790M^ (4I22.pdb). * *p* < 0.05; ** *p* < 0.01; *** *p* < 0.001.

Drug resistance in NSCLC patients is predominantly attributed to EGFR mutations, with the L858R and T790M mutations being the most prevalent EGFR mutations in NSCLC [32,33,34], and these mutations are associated with resistance to EGFR-TKIs in NSCLC [35,36]. To investigate whether **8aa** inhibits EGFR^L858R/T790M^, we performed immunoblot analysis on the EGF-induced phosphorylation of EGFR^L858R/T790M^ in H1975 cells expressing both EGFR mutations L858R and T790M. Notably, **8aa** potently inhibited the EGF-induced phosphorylation of EGFR^L858R/T790M^ compared to erlotinib (Figure 12A,B). In addition, a structural simulation of the **8aa** and EGFR^L858R/T790M^ complex revealed that **8aa** can interact with Asp855 of EGFR^L858R/T790M^. The indole N-H bond seemed to form a hydrogen bond with the carboxylic acid of Asp855, whereas the same type of hydrogen bonding interaction was not observed in erlotinib (Figure 12C,D). These results suggest that **8aa** can potently inhibit EGFR^L858R/T790M^ in NSCLC.

## 3. Experimental Section

### 3.1. General Methods

Unless specified, all reagents and starting materials were purchased from commercial sources and used as received without purification. “Concentrated” refers to the removal of volatile solvents via distillation using a rotary evaporator. “Dried” refers to pouring onto or passing through anhydrous magnesium sulfate followed by filtration. Flash chromatography was performed using silica gel (230–400 mesh) with hexanes, ethyl acetate, and dichloromethane as the eluents. All reactions were monitored by thin-layer chromatography on 0.25 mm silica plates (F-4) visualized with UV light. Melting points were measured by using a capillary melting point apparatus. ^1^H and ^13^C NMR spectra were recorded on a 400 MHz NMR spectrometer and were described as chemical shifts, multiplicity (s, singlet; d, doublet; t, triplet; q, quartet; m, multiplet), coupling constant in hertz (Hz), and number of protons. High-Resolution Mass Spectra (HRMS) were measured with an electrospray ionization (ESI) and Q-TOF mass analyzer.

#### 3.1.1. General Procedure for the Synthesis of **7**

A reaction mixture of glyoxal (0.33 mmol, 1 equiv), **5** (2.0 equiv), and HFIP (0.5 equiv) in toluene (4.0 mL) was stirred at 70 °C for 36 h. The reaction mixture was concentrated in vacuo to give the crude residue, which was purified by silica gel column chromatography (hexane/ethyl acetate/dichloromethane = 8:1:2) to afford **7**.

**2-Hydroxy-2-(2-hydroxy-4-methoxyphenyl)-1-phenylethan-1-one (7a).** Ivory solid, mp: 108.1–108.8 °C (81 mg, 95%); **^1^H NMR** (400 MHz, (CD_3_)_2_CO) δ 8.04 (d, *J* = 8.0 Hz, 2H), 7.55 (t, *J* = 7.6 Hz, 1H), 7.44 (t, *J* = 7.6 Hz, 2H), 7.05 (d, *J* = 8.4 Hz, 1H), 6.42 (d, *J* = 2.4 Hz, 1H), 6.37 (dd, *J* = 8.4, 2.4 Hz, 1H), 6.32 (s, 1H), 3.68 (s, 3H); **^13^C NMR** (100 MHz, CDCl_3_) δ 199.3, 161.0, 155.5, 133.9, 133.5, 129.6, 129.0, 128.6, 117.3, 106.9, 102.6, 71.7, 55.2; **HRMS** (ESI-QTOF) *m*/*z* [M+H]^+^ calcd for C_15_H_15_O_4_ 259.0965, found 259.0993.

**2-Hydroxy-2-(2-hydroxy-4-methoxyphenyl)-1-(3-methoxyphenyl)ethan-1-one (7b).** Ivory solid, mp: 105.9–106.2 °C (91 mg, 96%); **^1^H NMR** (400 MHz, (CD_3_)_2_CO) δ 7.70–7.55 (m, 2H), 7.34 (t, *J* = 8.0 Hz, 1H), 7.10 (d, *J* = 6.8 Hz, 1H), 7.05 (d, *J* = 8.0 Hz, 1H), 6.44 (s, 1H), 6.38 (d, *J* = 8.0 Hz, 1H), 6.31 (s, 1H), 3.80 (s, 3H), 3.68 (s, 3H); **^13^C NMR** (100 MHz, (CD_3_)_2_CO) δ 199.0, 160.8, 159.7, 155.4, 152.5, 135.7, 129.5, 120.8, 119.4, 118.8, 113.1, 105.5, 101.5, 70.1, 54.8, 54.5; **HRMS** (ESI-QTOF) *m*/*z* [M+Na]^+^ calcd for C_16_H_16_NaO_5_ 311.0890, found 311.0908.

**2-Hydroxy-2-(2-hydroxy-4-methoxyphenyl)-1-(naphthalen-2-yl)ethan-1-one (7c).** Ivory solid, mp: 107.2–107.9 °C (65 mg, 64%); **^1^H NMR** (400 MHz, CDCl_3_) δ 8.53 (s, 1H), 8.00 (d, *J* = 8.4 Hz, 1H), 7.90 (d, *J* = 8.0 Hz, 1H), 7.86–7.82 (m, 2H), 7.59 (t, *J* = 6.8 Hz, 1H), 7.53 (t, *J* = 7.2 Hz, 1H), 7.02 (d, *J* = 8.8 Hz, 1H), 6.69 (s, 1H), 6.35 (s, 3H), 4.48 (s, 1H), 3.67 (s, 3H); **^13^C NMR** (100 MHz, CDCl_3_) δ 199.2, 161.1, 155.8, 135.9, 132.3, 131.2, 131.0, 129.9, 129.8, 129.0, 128.6, 127.8, 126.9, 124.1, 117.1, 106.9, 102.9, 72.7, 55.3; **HRMS** (ESI-QTOF) *m*/*z* [M+Na]^+^ calcd for C_19_H_16_NaO_4_ 331.0941, found 331.0938.

**1-(3-Chlorophenyl)-2-hydroxy-2-(2-hydroxy-4-methoxyphenyl)ethan-1-one (7d).** Ivory solid, mp: 107.4–107.9 °C (86 mg, 89%); **^1^H NMR** (400 MHz, (CD_3_)_2_CO) δ 8.02 (s, 1H), 7.96 (d, *J* = 7.2 Hz, 1H), 7.58 (d, *J* = 8.4 Hz, 1H), 7.47 (t, *J* = 7.6 Hz, 1H), 7.09 (d, *J* = 8.4 Hz, 1H), 6.43 (s, 1H), 6.40 (d, *J* = 8.4 Hz, 1H), 6.28 (s, 1H), 3.69 (s, 3H); **^13^C NMR** (100 MHz, CDCl_3_) δ 198.1, 161.2, 155.3, 135.1, 135.0, 133.8, 130.0, 129.8, 128.9, 127.0, 116.6, 107.0, 102.8, 72.1, 55.3; **HRMS** (ESI-QTOF) *m*/*z* [M+Na]^+^ calcd for C_15_H_13_ClNaO_4_ 315.0395, found 315.0411.

**1-(4-Bromophenyl)-2-hydroxy-2-(2-hydroxy-4-methoxyphenyl)ethan-1-one (7e).** Ivory solid, mp: 108.1–108.8 °C (72 mg, 65%); **^1^H NMR** (400 MHz, (CD_3_)_2_CO) δ 7.95 (d, *J* = 7.6 Hz, 2H), 7.62 (d, *J* = 8.4 Hz, 2H), 7.07 (d, *J* = 8.4 Hz, 1H), 6.43 (s, 1H), 6.39 (d, *J* = 7.2 Hz, 1H), 6.26 (s, 1H), 3.68 (s, 3H); **^13^C NMR** (100 MHz, (CD_3_)_2_CO) δ 160.9, 155.6, 155.5, 133.8, 131.7, 130.3, 129.6, 127.5, 118.3, 105.6, 101.5, 70.5, 54.5; **HRMS** (ESI-QTOF) *m*/*z* [M+Na]^+^ calcd for C_15_H_13_BrNaO_4_ 358.9889, found 358.9912.

**1-(5-Bromothiophen-2-yl)-2-hydroxy-2-(2-hydroxy-4-methoxyphenyl)ethan-1-one (7f).** Yellow solid, mp: 125.3–125.9 °C (92 mg, 81%); **^1^H NMR** (400 MHz, (CD_3_)_2_CO) δ 7.76 (d, *J* = 3.6 Hz, 1H), 7.26–7.17 (m, 2H), 6.47–6.42 (m, 2H), 6.05 (s, 1H), 3.71 (s, 3H); **^13^C NMR** (100 MHz, (CD_3_)_2_CO) δ 191.4, 161.0, 155.6, 142.6, 133.7, 131.8, 129.7, 121.8, 118.5, 105.6, 101.7, 71.3, 54.6; **HRMS** (ESI-QTOF) *m*/*z* [M+H]^+^ calcd for C_13_H_12_BrO_4_S 342.9634, found 342.9626.

**2-Hydroxy-2-(2-hydroxy-4,5-dimethoxyphenyl)-1-phenylethan-1-one (7g).** Ivory solid, mp: 145.9–146.3 °C (93 mg, 98%); **^1^H NMR** (400 MHz, CDCl_3_) δ 7.97 (d, *J* = 7.6 Hz, 2H), 7.51 (t, *J* = 7.2 Hz, 1H), 7.37 (t, *J* = 7.6 Hz, 2H), 6.51 (s, 1H), 6.33 (s, 1H), 6.24 (s, 1H), 3.67 (s, 3H), 3.66 (s, 3H); **^13^C NMR** (100 MHz, CDCl_3_) δ 199.3, 150.2, 148.4, 143.2, 134.0, 133.4, 128.9, 128.6, 115.5, 111.3, 101.8, 71.5, 56.4, 55.7; **HRMS** (ESI-QTOF) *m*/*z* [M+Na]^+^ calcd for C_16_H_17_O_5_ 311.0890, found 311.0908.

**2-Hydroxy-2-(2-hydroxy-4,5-dimethoxyphenyl)-1-(3-methoxyphenyl)ethan-1-one (7h).** Ivory solid, mp: 162.9–163.2 °C (85 mg, 81%); **^1^H NMR** (400 MHz, (CD_3_)_2_CO) δ 7.66 (d, *J* = 6.8 Hz, 1H), 7.62 (s, 1H), 7.34 (t, *J* = 8.0 Hz, 1H), 7.10 (d, *J* = 6.4 Hz, 1H), 6.73 (s, 1H), 6.51 (s, 1H), 6.32 (s, 1H), 3.81 (s, 3H), 3.70 (s, 3H), 3.64 (s, 3H); **^13^C NMR** (100 MHz, (CD_3_)_2_CO) δ 199.1, 159.7, 150.5, 148.6, 143.1, 135.8, 129.5, 120.8, 119.4, 116.8, 113.1, 112.8, 101.0, 70.2, 56.0, 55.0, 54.8; **HRMS** (ESI-QTOF) *m*/*z* [M+Na]^+^ calcd for C_17_H_18_NaO_6_ 341.0996, found 341.1006.

**2-Hydroxy-2-(2-hydroxy-4,5-dimethoxyphenyl)-1-(4-methoxyphenyl)ethan-1-one (7i).** Ivory solid, mp: 109.7–110.0 °C (100 mg, 95%); **^1^H NMR** (400 MHz, CDCl_3_) δ 7.97 (d, *J* = 8.0 Hz, 2H), 6.86 (d, *J* = 8.0 Hz, 2H), 6.52 (s, 2H), 6.37 (s, 1H), 6.16 (s, 1H), 4.59 (s, 1H), 3.82 (s, 3H), 3.73 (s, 3H), 3.70 (s, 3H); **^13^C NMR** (100 MHz, CDCl_3_) δ 197.6, 164.2, 150.2, 148.4, 143.3, 131.4, 126.2, 116.0, 113.9, 111.3, 101.9, 71.2, 56.5, 55.7, 55.5; **HRMS** (ESI-QTOF) *m*/*z* [M+H]^+^ calcd for C_17_H_19_O_6_ 319.1176, found 319.1253.

**1-(3-Chlorophenyl)-2-hydroxy-2-(2-hydroxy-4,5-dimethoxyphenyl)ethan-1-one (7j).** Ivory solid, mp: 139.6–140.2 °C (100 mg, 94%); **^1^H NMR** (400 MHz, (CD_3_)_2_CO) δ 8.05 (s, 1H), 7.98 (d, *J* = 7.6 Hz, 1H), 7.57 (d, *J* = 8.0 Hz, 1H), 7.46 (t, *J* = 7.6 Hz, 1H), 6.76 (s, 1H), 6.49 (s, 1H), 6.29 (s, 1H), 3.68 (s, 3H), 3.64 (s, 3H); **^13^C NMR** (100 MHz, CDCl_3_) δ 199.3, 161.2, 157.6, 142.1, 141.6, 135.0, 130.5, 129.0, 127.8, 125.7, 109.9, 108.0, 100.6, 74.6, 56.3, 56.2; **HRMS** (ESI-QTOF) *m*/*z* [M+Na]^+^ calcd for C_16_H_15_ClNaO_5_ 345.0500, found 340.0502.

**2-Hydroxy-2-(2-hydroxy-4,6-dimethoxyphenyl)-1-phenylethan-1-one (7k).** Ivory solid, mp: 145.9–146.3 °C (58 mg, 62%); **^1^H NMR** (400 MHz, (CD_3_)_2_CO) δ 7.91 (d, *J* = 5.2 Hz, 2H), 7.50 (t, *J* = 6.8 Hz, 1H), 7.39 (t, *J* = 7.6 Hz, 2H), 6.25 (s, 1H), 6.04 (d, *J* = 8.0 Hz, 2H), 3.72 (s, 3H), 3.68 (s, 3H); **^13^C NMR** (100 MHz, CDCl_3_) δ 200.2, 161.6, 158.3, 156.9, 133.9, 133.5, 128.4, 128.3, 106.1, 94.6, 91.7, 68.7, 55.6, 55.2; **HRMS** (ESI-QTOF) *m*/*z* [M+Na]^+^ calcd for C_16_H_16_NaO_5_ 311.0890, found 311.0917.

**2-(5-(Benzyloxy)-2-hydroxy-4-methoxyphenyl)-2-hydroxy-1-phenylethan-1-one (7l).** Ivory solid, mp: 150.1–150.6 °C (72 mg, 60%); **^1^H NMR** (400 MHz, CDCl_3_) δ 7.86 (d, *J* = 7.2 Hz, 2H), 7.52 (t, *J* = 7.2 Hz, 1H), 7.37 (d, *J* = 7.6 Hz, 2H), 7.34–7.27 (m, 6H), 6.59 (s, 1H), 6.39 (s, 1H), 6.06 (s, 1H), 4.95 (s, 2H), 3.76 (s, 3H); **^13^C NMR** (100 MHz, CDCl_3_) δ 199.3, 149.5, 148.4, 143.8, 136.5, 134.0, 133.5, 129.0, 128.7, 128.5, 127.9, 127.2, 116.0, 112.3, 103.9, 71.7, 70.7, 56.7; **HRMS** (ESI-QTOF) *m*/*z* [M+Na]^+^ calcd for C_22_H_20_NaO_5_ 387.1203, found 387.1227.

**2-(5-(Benzyloxy)-2-hydroxy-4-methoxyphenyl)-2-hydroxy-1-(4-methoxyphenyl)ethan-1-one (7m).** Ivory solid, mp: 151.2–151.8 °C (54 mg, 42%); **^1^H NMR** (400 MHz, CDCl_3_) δ 7.97 (d, *J* = 8.4 Hz, 2H), 7.37–7.28 (m, 5H), 6.85 (d, *J* = 8.4 Hz, 2H), 6.55 (s, 1H), 6.50 (s, 1H), 6.38 (s, 1H), 6.14 (s, 1H), 4.96 (s, 2H), 4.58 (s, 1H), 3.81 (s, 3H), 3.68 (s, 3H); **^13^C NMR** (100 MHz, CDCl_3_) δ 197.6, 164.2, 149.4, 148.4, 143.8, 136.6, 131.5, 128.5, 127.9, 127.2, 126.2, 116.6, 113.9, 112.2, 103.9, 71.2, 70.7, 56.8, 55.5; **HRMS** (ESI-QTOF) *m*/*z* [M+Na]^+^ calcd for C_23_H_22_NaO_6_ 417.1309, found 417.1324.

**2-Hydroxy-2-(6-hydroxybenzo[*d*][1,3]dioxol-5-yl)-1-phenylethan-1-one (7n).** Ivory solid, mp: 139.2–139.9 °C (72 mg, 80%); **^1^H NMR** (400 MHz, CDCl_3_) δ 7.97 (d, *J* = 7.2 Hz, 2H), 7.54 (d, *J* = 7.6 Hz, 1H), 7.41 (t, *J* = 7.6 Hz, 2H), 6.48 (s, 1H), 6.44 (s, 1H), 6.31 (s, 1H), 6.21 (d, *J* = 4.4 Hz, 1H), 5.82 (d, *J* = 4.0 Hz, 2H), 4.55 (d, *J* = 4.4 Hz, 1H); **^13^C NMR** (100 MHz, CDCl_3_) δ 199.0, 149.3, 148.6, 141.8, 134.1, 133.3, 129.0, 128.7, 116.7, 107.4, 101.3, 99.6, 71.7; **HRMS** (ESI-QTOF) *m*/*z* [M+Na]^+^ calcd for C_15_H_12_NaO_5_ 295.0577, found 295.0592.

**1-(4-Bromophenyl)-2-hydroxy-2-(6-hydroxybenzo[*d*][1,3]dioxol-5-yl)ethan-1-one (7o).** Ivory solid, mp: 108.1–108.8 °C (81 mg, 70%); **^1^H NMR** (400 MHz, (CD_3_)_2_SO) δ 9.62 (s, 1H), 7.87 (d, *J* = 6.8 Hz, 2H), 7.67 (d, *J* = 8.0 Hz, 2H), 6.62 (s, 1H), 6.40 (s, 1H), 6.12 (s, 1H), 5.88 (s, 1H), 5.84 (s, 1H), 5.68 (s, 1H); **^13^C NMR** (100 MHz, (CD_3_)_2_SO) δ 198.6, 149.4, 147.7, 140.4, 134.4, 132.1, 130.6, 127.7, 118.0, 107.5, 101.3, 98.1, 69.7; **HRMS** (ESI-QTOF) *m*/*z* [M+Na]^+^ calcd for C_15_H_11_BrNaO_5_ 372.9682, found 372.9685.

**2-Hydroxy-2-(2-hydroxynaphthalen-1-yl)-1-phenylethan-1-one (7p).** White solid, mp: 114.8–115.5 °C (84 mg, 92%); **^1^H NMR** (400 MHz, (CD_3_)_2_CO) δ 8.04 (d, *J* = 6.8 Hz, 1H), 7.90–7.75 (m, 4H), 7.47 (t, *J* = 6.8 Hz, 1H), 7.42–7.29 (m, 4H), 7.21 (d, *J* = 8.0 Hz, 1H), 6.25 (s, 1H); **^13^C NMR** (100 MHz, (CD_3_)_2_CO) δ 155.3, 135.1, 132.5, 131.0, 129.4, 128.6, 128.5, 128.2, 127.6, 126.8, 126.1, 123.0, 122.8, 118.3, 117.7, 108.8; **HRMS** (ESI-QTOF) *m*/*z* [M+Na]^+^ calcd for C_18_H_14_NaO_3_ 301.0835, found 301.0854.

**2-Hydroxy-2-(1-hydroxynaphthalen-2-yl)-1-phenylethan-1-one (7q).** Ivory solid, mp: 118.8–119.2 °C (73 mg, 80%); **^1^H NMR** (400 MHz, (CD_3_)_2_CO) δ 8.23 (s, 1H), 8.06 (s, 2H), 7.76 (s, 1H), 7.56–7.51 (m, 1H), 7.49–7.41 (m, 4H), 7.36 (s, 2H), 6.54 (s, 1H); **^13^C NMR** (100 MHz, CDCl_3_) δ 198.9, 151.2, 134.4, 134.1, 133.6, 129.0, 128.7, 127.5, 126.9, 125.7, 125.6, 125.5, 121.9, 120.7, 117.0, 74.2; **HRMS** (ESI-QTOF) *m*/*z* [M+Na]^+^ calcd for C_18_H_14_NaO_3_ 301.0835, found 301.0855.

#### 3.1.2. General Procedure for the Synthesis of **8**

A reaction mixture of **7** (0.08 mmol, 1 equiv), indole (1.5 equiv), and PTSA (0.2 equiv) in CHCl_3_ (2.0 mL) was stirred at 60 °C for 18 h. The reaction mixture was concentrated in vacuo to give the crude residue, which was purified by silica gel column chromatography (hexane/ethyl acetate/dichloromethane = 30:1:2) to afford **8**.

**3-(6-Methoxy-2-phenylbenzofuran-3-yl)-1*H*-indole (8a).** Ivory solid, mp: 69.5–70.1 °C (26 mg, 97%); **^1^H NMR** (400 MHz, CDCl_3_) δ 8.36 (s, 1H), 7.72 (d, *J* = 7.2 Hz, 2H), 7.49 (d, *J* = 7.6 Hz, 1H), 7.37 (s, 1H), 7.32 (t, *J* = 8.8 Hz, 2H), 7.25–7.21 (m, 3H), 7.14 (s, 1H), 7.06 (t, *J* = 6.8 Hz, 1H), 6.86 (d, *J* = 8.8 Hz, 1H), 3.91 (s, 3H); **^13^C NMR** (100 MHz, CDCl_3_) δ 158.3, 155.0, 150.0, 136.4, 131.3, 128.3, 127.6, 126.6, 126.2, 124.7, 123.5, 122.5, 120.8, 120.7, 112.0, 111.6, 111.3, 110.2, 107.9, 95.7, 55.8; **HRMS** (ESI-QTOF) *m*/*z* [M+H]^+^ calcd for C_23_H_18_NO_2_ 340.1332, found 340.1316.

**3-(6-Methoxy-2-phenylbenzofuran-3-yl)-4-methyl-1*H*-indole (8b).** Brown solid, mp: 74.8–75.2 °C (27 mg, 97%); **^1^H NMR** (400 MHz, CDCl_3_) δ 8.34 (s, 1H), 7.67 (d, *J* = 8.0 Hz, 2H), 7.35 (d, *J* = 8.0 Hz, 1H), 7.25–7.21 (m, 2H), 7.20–7.14 (m, 4H), 7.12 (s, 1H), 6.88–6.81 (m, 2H), 3.90 (s, 3H), 2.18 (s, 3H); **^13^C NMR** (100 MHz, CDCl_3_) δ 158.3, 154.4, 150.7, 136.6, 131.7, 131.3, 128.4, 127.4, 126.6, 126.3, 125.6, 123.6, 122.6, 121.3, 120.8, 112.0, 111.8, 109.1, 107.6, 95.5, 55.8, 18.8; **HRMS** (ESI-QTOF) *m*/*z* [M+H]^+^ calcd for C_24_H_20_NO_2_ 354.1489, found 354.1472.

**5-Chloro-3-(6-methoxy-2-phenylbenzofuran-3-yl)-2-methyl-1*H*-indole (8c).** Yellow solid, mp: 94.2–94.8 °C (30 mg, 96%); **^1^H NMR** (400 MHz, CDCl_3_) δ 8.13 (s, 1H), 7.64 (d, *J* = 7.6 Hz, 2H), 7.32–7.27 (m, 3H), 7.25–7.21 (m, 2H), 7.18–7.12 (m, 3H), 6.85 (d, *J* = 8.4 Hz, 1H), 3.91 (s, 3H), 2.17 (s, 3H); **^13^C NMR** (100 MHz, CDCl_3_) δ 158.4, 155.0, 150.5, 134.6, 134.1, 131.4, 129.4, 128.4, 127.6, 125.6, 124.3, 121.8, 120.8, 118.9, 111.7, 111.4, 109.2, 107.5, 104.5, 95.8, 55.8, 12.6; **HRMS** (ESI-QTOF) *m*/*z* [M+H]^+^ calcd for C_24_H_19_ClNO_2_ 388.1099, found 388.1025.

**6-Chloro-3-(6-methoxy-2-phenylbenzofuran-3-yl)-1*H*-indole (8d).** Brown solid, mp: 128.5–128.9 °C (29 mg, 98%); **^1^H NMR** (400 MHz, CDCl_3_) δ 8.35 (s, 1H), 7.66 (d, *J* = 6.8 Hz, 2H), 7.48 (s, 1H), 7.37 (s, 1H), 7.29 (d, *J* = 8.8 Hz, 2H), 7.25–7.22 (m, 2H), 7.20 (d, *J* = 8.4 Hz, 1H), 7.13 (s, 1H), 7.01 (d, *J* = 8.8 Hz, 1H), 6.86 (d, *J* = 8.4 Hz, 1H), 3.90 (s, 3H); **^13^C NMR** (100 MHz, CDCl_3_) δ 158.4, 155.0, 150.2, 136.7, 131.1, 128.4, 128.4, 127.7, 126.2, 125.1, 124.4, 124.1, 121.6, 120.8, 120.5, 111.8, 111.2, 109.5, 108.2, 95.8, 55.8; **HRMS** (ESI-QTOF) *m*/*z* [M+H]^+^ calcd for C_23_H_17_ClNO_2_ 374.0942, found 374.0922.

**5-Bromo-3-(6-methoxy-2-phenylbenzofuran-3-yl)-1*H*-indole (8e).** Brown solid, mp: 183.5–184.6 °C (31 mg, 92%); **^1^H NMR** (400 MHz, CDCl_3_) δ 8.38 (s, 1H), 7.69–7.65 (m, 2H), 7.46 (s, 1H), 7.35 (s, 3H), 7.30–7.27 (m, 3H), 7.25–7.24 (m, 1H), 7.16–7.13 (m, 1H), 6.87 (d, *J* = 8.4 Hz, 1H), 3.91 (s, 3H); **^13^C NMR** (100 MHz, CDCl_3_) δ 158.4, 155.0, 150.4, 135.0, 131.1, 128.5, 128.4, 127.8, 126.2, 125.5, 124.6, 124.4, 123.1, 120.5, 113.4, 112.7, 111.8, 109.3, 107.8, 95.8, 55.8; **HRMS** (ESI-QTOF) *m*/*z* [M+H]^+^ calcd for C_23_H_17_BrNO_2_ 418.0437, found 418.0437.

**7-Bromo-3-(6-methoxy-2-phenylbenzofuran-3-yl)-1*H*-indole (8f).** Brown solid, mp: 107.2–107.9 °C (23 mg, 69%); **^1^H NMR** (400 MHz, CDCl_3_) δ 8.55 (s, 1H), 7.68 (d, *J* = 7.2 Hz, 2H), 7.43–7.39 (m, 2H), 7.32–7.27 (m, 2H), 7.25–7.22 (m, 3H), 7.14 (s, 1H), 6.93 (t, *J* = 8.0 Hz, 1H), 6.86 (d, *J* = 8.4 Hz, 1H), 3.90 (s, 3H); **^13^C NMR** (100 MHz, CDCl_3_) δ 158.4, 155.0, 150.2, 135.1, 131.1, 128.3, 127.7, 126.2, 125.9, 124.8, 124.4, 124.0, 121.2, 120.5, 120.0, 111.8, 109.6, 109.3, 104.8, 95.8, 55.8; **HRMS** (ESI-QTOF) *m*/*z* [M+H]^+^ calcd for C_23_H_17_BrNO_2_ 418.0437, found 418.0415.

**5-Iodo-3-(6-methoxy-2-phenylbenzofuran-3-yl)-1*H*-indole (8g).** Brown solid, mp: 135.1–135.9 °C (35 mg, 94%); **^1^H NMR** (400 MHz, CDCl_3_) δ 8.39 (s, 1H), 7.65 (s, 3H), 7.50 (d, *J* = 8.4 Hz, 1H), 7.32–7.27 (m, 4H), 7.25–7.23 (m, 2H), 7.13 (s, 1H), 6.87 (d, *J* = 7.6 Hz, 1H), 3.90 (s, 3H); **^13^C NMR** (100 MHz, CDCl_3_) δ 158.4, 155.0, 150.4, 135.4, 131.1, 130.9, 129.4, 129.2, 128.3, 127.8, 126.2, 124.4, 124.2, 120.5, 113.2, 111.8, 109.3, 107.4, 95.8, 83.5, 55.8; **HRMS** (ESI-QTOF) *m*/*z* [M+H]^+^ calcd for C_23_H_17_INO_2_ 466.0298, found 466.0244.

**3-(6-Methoxy-2-phenylbenzofuran-3-yl)-1-methyl-1*H*-indole (8h).** Ivory solid, mp: 148.9–149.5 °C (15 mg, 54%); **^1^H NMR** (400 MHz, CDCl_3_) δ 7.72 (d, *J* = 6.4 Hz, 2H), 7.42 (d, *J* = 7.6 Hz, 1H), 7.35–7.27 (m, 3H), 7.26–7.21 (m, 4H), 7.12 (s, 1H), 7.04 (t, *J* = 7.2 Hz, 1H), 6.85 (d, *J* = 8.4 Hz, 1H), 3.90–3.87 (m, 6H); **^13^C NMR** (100 MHz, CDCl_3_) δ 158.3, 155.0, 149.8, 137.2, 131.4, 128.2, 128.0, 127.5, 127.0, 126.2, 124.7, 121.9, 120.9, 120.8, 119.5, 111.6, 110.2, 109.4, 106.2, 95.7, 55.8, 33.0; **HRMS** (ESI-QTOF) *m*/*z* [M+H]^+^ calcd for C_24_H_20_NO_2_ 354.1489, found 354.1474.

**3-(6-Methoxy-2-(3-methoxyphenyl)benzofuran-3-yl)-1*H*-indole (8i).** White solid, mp: 136.5–136.9 °C (19 mg, 65%); **^1^H NMR** (400 MHz, CDCl_3_) δ 8.37 (s, 1H), 7.48 (d, *J* = 7.2 Hz, 1H), 7.40 (s, 1H), 7.32 (d, *J* = 8.4 Hz, 3H), 7.24 (s, 2H), 7.16 (d, *J* = 7.6 Hz, 1H), 7.13 (s, 1H), 7.06 (t, *J* = 7.6 Hz, 1H), 6.86 (d, *J* = 8.4 Hz, 1H), 6.76 (d, *J* = 7.6 Hz, 1H), 3.90 (s, 3H), 3.51 (s, 3H); **^13^C NMR** (100 MHz, CDCl_3_) δ 159.3, 158.4, 154.9, 149.8, 136.3, 132.5, 129.3, 126.4, 124.6, 123.6, 122.5, 120.8, 120.7, 120.0, 118.6, 114.1, 111.7, 111.2, 110.7, 107.9, 106.4, 95.6, 55.8, 54.9; **HRMS** (ESI-QTOF) *m*/*z* [M+H]^+^ calcd for C_24_H_20_NO_3_ 370.1438, found 370.1427.

**3-(6-Methoxy-2-(naphthalen-2-yl)benzofuran-3-yl)-1-methyl-1*H*-indole (8j).** Ivory solid, mp: 110.5–110.9 °C (21 mg, 65%); **^1^H NMR** (400 MHz, CDCl_3_) δ 8.30 (s, 1H), 7.80–7.70 (m, 3H), 7.61 (d, *J* = 8.8 Hz, 1H), 7.47–7.42 (m, 3H), 7.36 (t, *J* = 8.4 Hz, 2H), 7.31–7.27 (m, 2H), 7.17 (s, 1H), 7.02 (t, *J* = 7.6 Hz, 1H), 6.87 (t, *J* = 8.4 Hz, 1H), 3.91 (s, 6H); **^13^C NMR** (100 MHz, CDCl_3_) δ 158.4, 155.1, 149.7, 137.2, 133.3, 132.6, 128.9, 128.3, 128.2, 127.6, 127.6, 127.1, 126.2, 126.0, 125.0, 124.7, 124.2, 122.0, 121.0, 120.9, 119.6, 111.6, 110.9, 109.4, 106.2, 95.6, 55.8, 33.1; **HRMS** (ESI-QTOF) *m*/*z* [M+H]^+^ calcd for C_28_H_22_NO_2_ 404.1645, found 404.1626.

**3-(2-(3-Chlorophenyl)-6-methoxybenzofuran-3-yl)-1*H*-indole (8k).** Ivory solid, mp: 140.3–140.8 °C (26 mg, 87%); **^1^H NMR** (400 MHz, CDCl_3_) δ 8.38 (s, 1H), 7.78 (s, 1H), 7.49 (d, *J* = 8.0 Hz, 2H), 7.37 (s, 1H), 7.34–7.28 (m, 2H), 7.26–7.23 (m, 1H), 7.18–7.14 (m, 1H), 7.12–7.05 (m, 3H), 6.85 (d, *J* = 8.4 Hz, 1H), 3.89 (s, 3H); **^13^C NMR** (100 MHz, CDCl_3_) δ 158.7, 155.1, 148.3, 136.3, 134.3, 133.0, 129.5, 128.7, 127.4, 126.3, 125.8, 124.3, 124.1, 123.5, 122.6, 121.0, 120.6, 120.1, 111.9, 111.3, 107.5, 95.6, 55.8; **HRMS** (ESI-QTOF) *m*/*z* [M+H]^+^ calcd for C_23_H_17_ClNO_2_ 374.0942, found 374.0919.

**4-Bromo-3-(2-(4-bromophenyl)-6-methoxybenzofuran-3-yl)-1*H*-indole (8l).** Yellow solid, mp: 207.5–207.9 °C (30 mg, 75%); **^1^H NMR** (400 MHz, CDCl_3_) δ 8.47 (s, 1H), 7.47 (t, *J* = 8.4 Hz, 3H), 7.34 (d, *J* = 8.4 Hz, 2H), 7.29 (d, *J* = 7.6 Hz, 1H), 7.25–7.23 (m, 1H), 7.16–7.08 (m, 3H), 6.84 (d, *J* = 8.4 Hz, 1H), 3.89 (s, 3H); **^13^C NMR** (100 MHz, CDCl_3_) δ 158.4, 154.4, 150.4, 137.2, 131.5, 130.4, 127.2, 126.6, 125.9, 125.2, 124.7, 123.6, 121.4, 121.0, 114.5, 111.9, 110.9, 110.7, 108.0, 95.4, 55.8; **HRMS** (ESI-QTOF) *m*/*z* [M+H]^+^ calcd for C_23_H_16_Br_2_NO_2_ 495.9542, found 495.9517.

**3-(2-(5-Bromothiophen-2-yl)-6-methoxybenzofuran-3-yl)-1*H*-indole (8m).** Brown solid, mp: 74.8–75.2 °C (18 mg, 54%); **^1^H NMR** (400 MHz, CDCl_3_) δ 8.40 (s, 1H), 7.50 (d, *J* = 8.4 Hz, 1H), 7.45 (d, *J* = 2.0 Hz, 1H), 7.39 (d, *J* = 8.0 Hz, 1H), 7.30–7.27 (m, 2H), 7.17 (d, *J* = 4.4 Hz, 1H), 7.13–7.07 (m, 2H), 6.94 (t, *J* = 4.8 Hz, 1H), 6.84 (dd, *J* = 8.4, 1.6 Hz, 1H), 3.89 (s, 3H); **^13^C NMR** (100 MHz, CDCl_3_) δ 158.4, 154.8, 146.6, 136.3, 133.2, 127.2, 126.6, 125.0, 124.7, 124.4, 124.1, 122.5, 120.6, 120.6, 120.0, 111.7, 111.3, 109.4, 107.0, 95.7, 55.8; **HRMS** (ESI-QTOF) *m*/*z* [M+H]^+^ calcd for C_21_H_15_BrNO_2_S 424.0001, found 424.0004.

**3-(5,6-Dimethoxy-2-phenylbenzofuran-3-yl)-1*H*-indole (8n).** Brown solid, mp: 183.7–184.2 °C (24 mg, 80%); **^1^H NMR** (400 MHz, CDCl_3_) δ 8.39 (s, 1H), 7.67 (d, *J* = 8.0 Hz, 2H), 7.50 (d, *J* = 8.0 Hz, 1H), 7.38–7.34 (m, 2H), 7.27–7.19 (m, 4H), 7.15 (s, 1H), 7.07 (t, *J* = 8.0 Hz, 1H), 6.85 (s, 1H), 3.98 (s, 3H), 3.80 (s, 3H); **^13^C NMR** (100 MHz, CDCl_3_) δ 150.2, 148.7, 148.3, 146.6, 136.4, 131.4, 128.2, 127.4, 126.6, 126.0, 123.4, 123.2, 122.5, 120.7, 120.0, 111.3, 110.4, 108.1, 101.7, 95.1, 56.4; **HRMS** (ESI-QTOF) *m*/*z* [M+H]^+^ calcd for C_24_H_20_NO_3_ 370.1438, found 370.1428.

**3-(5,6-Dimethoxy-2-(3-methoxyphenyl)benzofuran-3-yl)-1*H*-indole (8o).** Brown solid, mp: 128.9–129.4 °C (28 mg, 89%); **^1^H NMR** (400 MHz, CDCl_3_) δ 8.44 (s, 1H), 7.49 (d, *J* = 7.2 Hz, 1H), 7.42–7.34 (m, 2H), 7.32–7.27 (m, 1H), 7.22–7.12 (m, 4H), 7.08 (t, *J* = 7.2 Hz, 1H), 6.86 (s, 1H), 6.76 (d, *J* = 5.2 Hz, 1H), 3.99 (s, 3H), 3.81 (s, 3H), 3.51 (s, 3H); **^13^C NMR** (100 MHz, CDCl_3_) δ 159.3, 150.0, 148.6, 148.4, 146.6, 136.3, 132.5, 129.3, 126.6, 123.5, 123.2, 122.5, 120.7, 120.1, 118.4, 113.9, 111.3, 110.7, 110.6, 108.0, 101.7, 95.1, 56.4, 54.9; **HRMS** (ESI-QTOF) *m*/*z* [M+H]^+^ calcd for C_25_H_22_NO_4_ 400.1543, found 400.1550.

**6-Bromo-3-(5,6-dimethoxy-2-(4-methoxyphenyl)benzofuran-3-yl)-1*H*-indole (8p).** Brown solid, mp: 249.9–250.4 °C (31 mg, 81%); **^1^H NMR** (400 MHz, CDCl_3_) δ 8.43 (s, 1H), 7.64 (s, 1H), 7.56 (d, *J* = 8.4 Hz, 2H), 7.34 (s, 1H), 7.21–7.13 (m, 3H), 6.79 (s, 2H), 6.77 (s, 1H), 3.97 (s, 3H), 3.81 (s, 3H), 3.78 (s, 3H); **^13^C NMR** (100 MHz, CDCl_3_) δ 159.1, 150.5, 148.4, 147.9, 146.5, 137.1, 127.4, 125.5, 124.0, 123.9, 123.4, 123.1, 121.9, 116.1, 114.2, 113.8, 108.5, 108.0, 101.3, 95.1, 56.4, 55.2; **HRMS** (ESI-QTOF) *m*/*z* [M+Na]^+^ calcd for C_25_H_20_BrNNaO_4_ 500.0468, found 500.0473.

**3-(2-(3-Chlorophenyl)-5,6-dimethoxybenzofuran-3-yl)-5-iodo-1*H*-indole (8q).** Brown solid, mp: 230.7–231.0 °C (42 mg, 99%); **^1^H NMR** (400 MHz, CDCl_3_) δ 8.48 (s, 1H), 7.71 (s, 2H), 7.53 (d, *J* = 8.4 Hz, 1H), 7.45 (d, *J* = 7.6 Hz, 1H), 7.34–7.28 (m, 2H), 7.20–7.12 (m, 3H), 6.79 (s, 1H), 3.99 (s, 3H), 3.83 (s, 3H); **^13^C NMR** (100 MHz, CDCl_3_) δ 148.8, 148.7, 146.8, 135.4, 134.3, 132.8, 131.1, 129.6, 129.3, 129.0, 127.5, 125.7, 124.2, 123.9, 122.6, 113.4, 110.8, 107.0, 101.5, 95.1, 83.7, 56.4; **HRMS** (ESI-QTOF) *m*/*z* [M+Na]^+^ calcd for C_24_H_17_ClINNaO_3_ 551.9834, found 551.9838.

**3-(4,6-Dimethoxy-2-phenylbenzofuran-3-yl)-6-methyl-1*H*-indole (8r).** Brown solid, mp: 154.9–155.5 °C (25 mg, 80%); **^1^H NMR** (400 MHz, CDCl_3_) δ 8.10 (s, 1H), 7.58 (d, *J* = 8.0 Hz, 2H), 7.24–7.17 (m, 6H), 6.86 (d, *J* = 8.0 Hz, 1H), 6.76 (s, 1H), 6.30 (s, 1H), 3.89 (s, 3H), 3.58 (s, 3H), 2.47 (s, 3H); **^13^C NMR** (100 MHz, CDCl_3_) δ 159.0, 156.0, 155.0, 149.2, 136.4, 131.6, 131.4, 128.1, 127.2, 126.1, 125.3, 123.8, 121.4, 120.6, 114.0, 110.9, 109.5, 108.3, 94.5, 87.9, 55.8, 55.5, 21.8; **HRMS** (ESI-QTOF) *m*/*z* [M+H]^+^ calcd for C_25_H_22_NO_3_ 384.1594, found 384.1590.

**3-(5-(Benzyloxy)-6-methoxy-2-phenylbenzofuran-3-yl)-1*H*-indole (8s).** Brown solid, mp: 162.7–163.2 °C (35 mg, 99%); **^1^H NMR** (400 MHz, CDCl_3_) δ 8.38 (s, 1H), 7.68 (d, *J* = 6.8 Hz, 2H), 7.48 (d, *J* = 8.0 Hz, 1H), 7.43–7.39 (m, 2H), 7.37–7.22 (m, 9H), 7.18 (s, 1H), 7.05 (t, *J* = 7.2 Hz, 1H), 6.95 (s, 1H), 5.05 (s, 2H), 3.98 (s, 3H); **^13^C NMR** (100 MHz, CDCl_3_) δ 150.1, 149.2, 149.2, 145.6, 137.2, 136.3, 131.4, 128.4, 128.2, 127.8, 127.6, 127.4, 126.5, 126.1, 123.4, 123.2, 122.4, 120.7, 120.1, 111.2, 110.4, 108.0, 105.4, 95.6, 71.9, 56.5; **HRMS** (ESI-QTOF) *m*/*z* [M+H]^+^ calcd for C_30_H_24_NO_3_ 446.1751, found 446.1755.

**2-(6-(Benzyloxy)-5-methoxy-2-(4-methoxyphenyl)benzofuran-3-yl)-3-methyl-1*H*-indole (8t).** Brown solid, mp: 194.8–195.2 °C (25 mg, 65%); **^1^H NMR** (400 MHz, CDCl_3_) δ 7.98 (s, 1H), 7.67 (d, *J* = 7.6 Hz, 1H), 7.53 (d, *J* = 8.8 Hz, 2H), 7.49 (d, *J* = 7.6 Hz, 2H), 7.43–7.37 (m, 3H), 7.36–7.31 (m, 1H), 7.25–7.18 (m, 2H), 7.12 (s, 1H), 6.83 (d, *J* = 8.8 Hz, 2H), 6.80 (s, 1H), 5.24 (s, 2H), 3.85 (s, 3H), 3.79 (s, 3H), 2.20 (s, 3H); **^13^C NMR** (100 MHz, CDCl_3_) δ 159.5, 151.7, 148.1, 147.5, 147.0, 136.9, 136.3, 129.4, 128.6, 127.9, 127.3, 126.0, 123.3, 122.9, 122.1, 119.3, 118.9, 114.1, 110.9, 107.0, 101.7, 97.9, 71.5, 56.6, 55.2, 9.5; **HRMS** (ESI-QTOF) *m*/*z* [M+H]^+^ calcd for C_32_H_28_NO_4_ 490.2013, found 490.2007.

**3-(6-Phenyl-[1,3]dioxolo[4,5-*f*]benzofuran-7-yl)-1*H*-indole (8u).** Brown solid, mp: 193.9–194.4 °C (19 mg, 68%); **^1^H NMR** (400 MHz, CDCl_3_) δ 8.33 (s, 1H), 7.66 (d, *J* = 8.0 Hz, 2H), 7.47 (d, *J* = 8.0 Hz, 1H), 7.34–7.30 (m, 2H), 7.25–7.18 (m, 4H), 7.08–7.03 (m, 2H), 6.79 (s, 1H), 5.97 (s, 2H); **^13^C NMR** (100 MHz, CDCl_3_) δ 150.6, 149.2, 146.4, 144.5, 136.3, 131.3, 128.3, 127.5, 126.5, 126.0, 124.7, 123.4, 122.5, 120.6, 120.0, 111.3, 110.7, 107.9, 101.3, 99.1, 93.3; **HRMS** (ESI-QTOF) *m*/*z* [M+H]^+^ calcd for C_23_H_16_NO_3_ 354.1125, found 354.1118.

**7-Methyl-3-(6-phenyl-[1,3]dioxolo[4,5-*f*]benzofuran-7-yl)-1*H*-indole (8v).** Brown solid, mp: 185.8–186.4 °C (27 mg, 92%); **^1^H NMR** (400 MHz, CDCl_3_) δ 8.27 (s, 1H), 7.69 (d, *J* = 7.6 Hz, 2H), 7.31 (s, 1H), 7.25–7.20 (m, 4H), 7.11–7.05 (m, 2H), 7.00 (t, *J* = 6.4 Hz, 1H), 6.82–6.80 (m, 1H), 5.99 (s, 2H), 2.58 (s, 3H); **^13^C NMR** (100 MHz, CDCl_3_) δ 150.5, 149.2, 146.3, 144.5, 136.0, 131.3, 128.3, 127.5, 126.1, 126.0, 124.7, 123.2, 123.0, 120.5, 120.2, 118.4, 110.9, 108.3, 101.3, 99.1, 93.3, 16.7; **HRMS** (ESI-QTOF) *m*/*z* [M+H]^+^ calcd for C_24_H_18_NO_3_ 368.1281, found 368.1259.

**3-(6-(4-Bromophenyl)-[1,3]dioxolo[4,5-*f*]benzofuran-7-yl)-5-methyl-1*H*-indole (8w).** Brown solid, mp: 205.4–206.0 °C (32 mg, 89%); **^1^H NMR** (400 MHz, CDCl_3_) δ 8.28 (s, 1H), 7.53 (d, *J* = 8.4 Hz, 2H), 7.38 (d, *J* = 8.8 Hz, 1H), 7.35 (d, *J* = 8.8 Hz, 2H), 7.13–7.09 (m, 2H), 7.06 (s, 1H), 6.78 (s, 1H), 5.99 (s, 2H), 2.36 (s, 3H); **^13^C NMR** (100 MHz, CDCl_3_) δ 149.5, 149.2, 146.6, 144.6, 134.6, 131.4, 130.2, 129.6, 127.3, 126.7, 124.7, 124.3, 123.5, 121.3, 119.9, 111.5, 111.0, 106.9, 101.3, 99.1, 93.3, 21.5; **HRMS** (ESI-QTOF) *m*/*z* [M+H]^+^ calcd for C_24_H_16_BrNO_3_ 445.0314, found 445.0315.

**3-(2-Phenylnaphtho[2,1-*b*]furan-1-yl)-1*H*-indole (8x).** Brown solid, mp: 148.2–148.9 °C (25 mg, 88%); **^1^H NMR** (400 MHz, CDCl_3_) δ 8.41 (s, 1H), 7.92 (d, *J* = 8.0 Hz, 1H), 7.79 (s, 2H), 7.67–7.61 (m, 3H), 7.57 (d, *J* = 8.4 Hz, 1H), 7.43 (d, *J* = 7.6 Hz, 1H), 7.39–7.32 (m, 2H), 7.31–7.29 (m, 1H), 7.25–7.19 (m, 3H), 7.15 (t, *J* = 7.6 Hz, 1H), 7.09 (t, *J* = 7.2 Hz, 1H); **^13^C NMR** (100 MHz, CDCl_3_) δ 151.69, 151.66, 136.5, 131.1, 130.8, 128.7, 128.6, 128.3, 127.8, 127.7, 126.1, 125.9, 125.7, 124.6, 124.2, 123.6, 123.2, 122.7, 120.5, 120.4, 112.3, 111.3, 111.2, 109.2; **HRMS** (ESI-QTOF) *m*/*z* [M+H]^+^ calcd for C_26_H_18_NO 360.1383, found 360.1370.

**2-Methyl-3-(2-phenylnaphtho[1,2-*b*]furan-3-yl)-1*H*-indole (8y).** Brown solid, mp: 95.3–95.9 °C (19 mg, 62%); **^1^H NMR** (400 MHz, CDCl_3_) δ 8.51 (d, *J* = 8.4 Hz, 1H), 8.13 (s, 1H), 7.96 (d, *J* = 8.0 Hz, 1H), 7.83 (d, *J* = 8.0 Hz, 2H), 7.69–7.61 (m, 2H), 7.53 (t, *J* = 7.6 Hz, 1H), 7.43 (d, *J* = 7.6 Hz, 2H), 7.37 (d, *J* = 8.0 Hz, 1H), 7.32 (t, *J* = 7.2 Hz, 2H), 7.28–7.27 (m, 1H), 7.25–7.21 (m, 1H), 7.09 (t, *J* = 8.0 Hz, 1H), 2.23 (s, 3H); **^13^C NMR** (100 MHz, CDCl_3_) δ 150.6, 149.5, 135.8, 133.1, 131.7, 131.6, 130.1, 129.2, 128.5, 128.3, 127.6, 126.7, 126.5, 126.3, 125.8, 125.1, 123.2, 121.6, 121.4, 120.2, 119.9, 119.7, 119.6, 110.4, 12.7; **HRMS** (ESI-QTOF) *m*/*z* [M+H]^+^ calcd for C_27_H_20_NO 374.1539, found 374.1544.

**5-Chloro-3-(6-methoxy-2-phenylbenzofuran-3-yl)-1*H*-indole (8z).** Ivory solid, mp: 88.2–88.9 °C (28 mg, 92%); **^1^H NMR** (400 MHz, CDCl_3_) δ 8.40 (s, 1H), 7.67 (d, *J* = 8.0 Hz, 2H), 7.42–7.36 (m, 2H), 7.31–7.27 (m, 3H), 7.25–7.19 (m, 3H), 7.14 (s, 1H), 6.87 (d, *J* = 8.4 Hz, 1H), 3.91 (s, 3H); **^13^C NMR** (100 MHz, CDCl_3_) δ 158.4, 155.0, 150.3, 134.7, 131.1, 128.4, 127.8, 126.1, 125.8, 124.8, 124.4, 122.9, 120.5, 120.0, 112.3, 111.8, 109.4, 107.8, 95.7, 55.8; **HRMS** (ESI-QTOF) *m*/*z* [M+H]^+^ calcd for C_23_H_17_ClNO_2_ 374.0942, found 374.0951.

**5-Bromo-3-(5,6-dimethoxy-2-phenylbenzofuran-3-yl)-1*H*-indole (8aa).** White solid, mp: 100.2–100.5 °C (29 mg, 81%); **^1^H NMR** (400 MHz, CDCl_3_) δ 8.46 (s, 1H), 7.63 (d, *J* = 7.6 Hz, 2H), 7.49 (s, 1H), 7.37–7.31 (m, 3H), 7.25–7.21 (m, 3H), 7.15 (s, 1H), 6.79 (s, 1H), 3.97 (s, 3H), 3.81 (s, 3H); **^13^C NMR** (100 MHz, CDCl_3_) δ 150.4, 148.6, 148.3, 146.6, 134.9, 131.1, 128.5, 128.3, 127.6, 125.9, 125.5, 124.6, 123.0, 122.9, 113.4, 112.8, 109.5, 107.8, 101.4, 95.1, 56.4, 56.3; **HRMS** (ESI-QTOF) *m*/*z* [M+Na]^+^ calcd for C_24_H_18_BrNNaO_3_ 470.0362, found 470.0356.

**5-Bromo-3-(5,6-dimethoxy-2-(4-methoxyphenyl)benzofuran-3-yl)-1*H*-indole (8ab).** Ivory solid, mp: 92.2–92.9 °C (34 mg, 89%); **^1^H NMR** (400 MHz, (CD_3_)_2_CO) δ 7.67 (s, 1H), 7.59–7.55 (m, 2H), 7.52 (d, *J* = 8.4 Hz, 1H), 7.30–7.28 (m, 1H), 7.26–7.24 (m, 1H), 6.88 (s, 1H), 6.84 (d, *J* = 8.8 Hz, 3H), 3.90 (s, 3H), 3.77 (s, 3H), 3.73 (s, 3H); **^13^C NMR** (100 MHz, (CD_3_)_2_CO) δ 159.4, 150.0, 148.8, 148.5, 147.2, 135.6, 128.3, 127.2, 126.2, 126.0, 124.4, 124.0, 122.9, 122.2, 113.8, 112.1, 108.5, 106.5, 102.0, 95.5, 55.68, 55.65, 54.7; **HRMS** (ESI-QTOF) *m*/*z* [M+Na]^+^ calcd for C_25_H_20_BrNNaO_4_ 500.0468, found 500.0454.

**5-Bromo-3-(6-phenyl-[1,3]dioxolo[4,5-*f*]benzofuran-7-yl)-1*H*-indole (8ac).** Ivory solid, mp: 110.2–110.9 °C (33 mg, 96%); **^1^H NMR** (400 MHz, CDCl_3_) δ 8.38 (s, 1H), 7.63–7.59 (m, 2H), 7.44 (s, 1H), 7.34 (s, 2H), 7.31–7.29 (m, 1H), 7.25–7.21 (m, 3H), 7.07 (s, 1H), 6.73 (s, 1H), 5.98 (s, 2H); **^13^C NMR** (100 MHz, CDCl_3_) δ 150.9, 149.2, 146.5, 144.6, 134.9, 131.0, 128.4, 128.3, 127.7, 125.9, 125.5, 124.6, 124.5, 122.9, 113.4, 112.8, 109.9, 107.6, 101.3, 98.8, 93.4; **HRMS** (ESI-QTOF) *m*/*z* [M+H]^+^ calcd for C_23_H_15_BrNO_3_ 432.0230, found 423.0211.

**5-Iodo-3-(6-phenyl-[1,3]dioxolo[4,5-*f*]benzofuran-7-yl)-1*H*-indole (8ad).** Ivory solid, mp: 113.9–114.2 °C (36 mg, 94%); **^1^H NMR** (400 MHz, CDCl_3_) δ 8.38 (s, 1H), 7.63 (s, 1H), 7.60 (d, *J* = 8.0 Hz, 2H), 7.49 (d, *J* = 8.8 Hz, 1H), 7.28–7.21 (m, 5H), 7.07 (s, 1H), 6.72 (s, 1H), 5.98 (s, 2H); **^13^C NMR** (100 MHz, CDCl_3_) δ 151.0, 149.2, 146.5, 144.6, 135.4, 131.0, 130.9, 129.2, 129.1, 128.3, 127.7, 125.9, 124.5, 124.2, 113.3, 109.8, 107.3, 101.3, 98.7, 93.4, 83.6; **HRMS** (ESI-QTOF) *m*/*z* [M+H]^+^ calcd for C_23_H_15_INO_3_ 480.0091, found 480.0061.

### 3.2. Bioassay

#### 3.2.1. Cell Culture

A549, HT29, MCF7, HepG2, PC3, and HEK293T cells were purchased from the Korean Cell Line Bank (Seoul, Republic of Korea). HaCaT, H1975, and PC9 cells were obtained from Prof. Sohee Kwon (Yonsei University, Incheon, Republic of Korea), Prof. Dosik Min, and Daegu Gyeongbuk Medical Innovation Foundation, respectively. The hepatocellular carcinoma cells (HepG2), breast adenocarcinoma cells (MCF-7), human keratinocyte cells (HaCaT), and human embryonic kidney cells (HEK293T) were cultured in high-glucose DMEM (Welgene Inc., Gyeongsan, Republic of Korea), and the non-small-cell lung carcinoma cells (PC9, A549, H1975), colorectal adenocarcinoma cells (HT29), and prostate adenocarcinoma cells (PC3) were grown in RPMI 1640 medium (Welgene Inc., Gyeongsan, Republic of Korea). In addition, 10% fetal bovine serum (FBS), 100 μg/mL streptomycin, and 100 Units/mL penicillin were supplemented in all media. All cells were grown at 5% CO_2_, 37 °C, and 95% humidity.

#### 3.2.2. Cell Proliferation Assay

Our cell proliferation assay was performed using the Cell Titer 96^®^ AQueous One Solution Cell proliferation Assay kit (Promega, Madison, WI, USA). All cells were grown in 96-well microplates in RPMI1640 medium containing 10% fetal bovine serum (FBS) for 24 h. After 24 h incubation, the cells were treated with DMSO and test compounds. The medium and test compounds were changed every 24 h. To estimate cell viability, the cells were incubated with MTS solution for 50 min. The absorbance was quantified by using an Infinite M200 microplate reader (Tecan, Männedorf, Switzerland) at 490 nm.

#### 3.2.3. In Vitro Wound Healing Assay

The inhibitory effect of **8aa** on cell migration was measured through an in vitro wound healing assay. PC9 and A549 cells were cultured to approximately 100% confluence in a 96-well microplate for 24 h to form a monolayer. After 24 h, wounds were formed through 96-well wound maker (Essen BioScience, Ann Arbor, MI, USA). Then, the growth medium was washed out three times with phosphate-buffered saline (PBS) and replaced with 200 μL of DMEM and RPMI 1640 medium containing **8aa** or DMSO. Images of the wound area were acquired by using IncuCyte ZOOM (Essen BioScience, Ann Arbor MI, USA), and wound closure rate was measured using IncuCyte software (2018A).

#### 3.2.4. Caspase-3 Activity Assay

PC9 and A549 cells were grown in 96-black well plates to approximately 50% confluence, and then the cells were incubated with **8aa** for 24 h. To measure caspase-3 activity, the growth medium was replaced with 100 μL of PBS containing 1 μM of caspase-3 substrate and NucView 488 and incubated at room temperature for 30 min. Then, the cells were stained with 1 μM of Hoechst 33342. Caspase-3 activity was completely inhibited by Ac-DEVD-CHO, a potent caspase-3 inhibitor. The FLUOstar Omega microplate reader (BMG Labtech, Ortenberg, Germany) was used to measure the fluorescence of Hoechst 33342 and NucView 488, and a Lionheart FX Automated Microscope (BioTek, Winooski, VT, USA) was used to obtain the fluorescence microscopy images.

#### 3.2.5. Immunoblot Analysis

The preparation of the protein sample was conducted as described previously [5]. The samples were centrifuged at 13,000 RPM for 20 min at 4 °C to eliminate cell debris, and the samples were separated using 4–12% Tris Glycine Precast Gel (KOMA BIOTECH, Seoul, Republic of Korea) for 60 min at 130 V. After 60 min, the samples were transferred onto a polyvinylidene Fluoride membrane (PVDF) (Millipore, Billerica, MA, USA) for 90 min at 30 V. Membrane blocking was conducted using Tris-buffered saline with 0.1% Tween 20 (TBST) containing 5% bovine serum albumin (BSA) at room temperature for 60 min. After membrane blocking, the membranes were incubated overnight at 4 °C with the following primary antibodies: anticleaved PARP (BD Biosciences, Franklin Lakes, NJ, USA), anti-β-actin (Santa Cruz Biotechnology, Dallas, TX, USA), anti-phospho-EGFR (Tyr1068) (Cell Signaling, Danvers, MA, USA), anti-EGFR (Santa Cruz Biotechnology, Dallas, TX, USA), anti-phospho-AKT (Santa Cruz Biotechnology, Dallas, TX, USA), anti-AKT (Santa Cruz Biotechnology, Dallas, TX, USA), anti-phospho-p42/44 (Cell Signaling, Danvers, MA, USA), and anti-p42/44 (Cell Signaling, Danvers, MA, USA). Then, the membranes were washed out three times every five minutes with 0.1% TBST and incubated with horseradish peroxidase (HRP) conjugated secondary IgG antibodies at room temperature for 60 min. After washing three times, the membranes were visualized using the EzWestLumi Plus (mid-femto-grade ECL) (ATTO, Amherst, NY, USA) immunoblot analysis detection system (GE Healthcare, Piscataway, NJ, USA). All experiments were repeated three times independently.

#### 3.2.6. Cell Cycle Analysis

PC9 and A549 cells were grown to ~60% confluence in a 6-well plate; then, the cells were treated with 10 μM of **8aa** for 24 h. After 24 h, the PC9 and A549 cells were washed out twice with phosphate-buffered saline (PBS) and trypsinized using 0.5% trypsin-EDTA before the cells were centrifuged at 1000 RPM for 2 min at room temperature. Finally, the cells were stained with propidium iodide (PI) for 30 min, and then cell cycle phases were measured by using FACS (Beckman Coulter, Fullerton, CA, USA).

### 3.3. Molecular Docking Analysis

Molecular docking was studied using Maestro (Schrödinger Release 2022-1). The X-ray crystal structures of the EGFR (1M17.pdb) and EGFR^L858R/T790M^ (4I22.pdb) were prepared by removing all water and hydrogen assignments at pH 7.0 using the Protein Preparation Wizard module. Compounds were minimized by using the conjugate gradient algorithm and the OPLS2005 force field with Minimization module in Maestro. The Glide module was used to generate the receptor grid and carry out ligand docking. The docking model figures were generated using PyMOL version 1.8.6.1. The amino acid numbers of 1M17.pdb were corrected based on other published X-ray cocrystal structures (7UKV, 7U99.pdb).

### 3.4. EGFR Kinase Activity Assay

The inhibitory effect of **8aa** on EGFR kinase activity was evaluated using an EGFR kinase assay kit (BPS Bioscience, San Diego, CA, USA) according to the manufacturer’s instructions. Briefly, a mixture of 5X kinase buffer 1, ATP (500 µM), PTK substrate (10 mg/mL), and water was prepared. Subsequently, **8aa** was treated at various concentrations, and the reaction was initiated by adding the EGFR (1 ng/µL). After a 40-min incubation period at 30 °C, each well was treated with Kinase-Glo Max reagent (Promega, Madison, WI, USA) and incubated for 15 min at room temperature. Luminescence was measured using an Infinite M200 microplate reader (Tecan, Männedorf, Switzerland).

## 4. Conclusions

In summary, we established highly efficient modular access to a range of 2-arylbenzofurans with an indole at the C3 position via the HFIP-catalyzed hydroxyalkylation of phenols with (hetero) arylglyoxals, followed by PTSA-catalyzed substitution–cyclodehydration with indoles, enabling the installation of two distinct substituents at the C2 and C3 sites of benzofuran with the formation of two C-C bonds and one C-O bond. Biological evaluations and structure–activity relationship (SAR) studies of these products against the EGFR in NSCLC cells led us to identify **8aa** as a novel EGFR inhibitor. Notably, **8aa** potently inhibited the EGFR and EGFR-mediated signaling pathways such as AKT and ERK1/2 in a dose-dependent manner, and it also showed selective reductions in cell viability against human NSCLC cell lines PC9 and A549. **8aa** exhibited limited impact on cell viability in other cancer cell lines, including MCF7, HepG2, PC3, and HT29 cells, as well as non-tumorigenic cells such as HaCaT and HEK293T cells. Moreover, **8aa** significantly inhibited cell migration and induced apoptosis via increasing caspase-3 activity and PARP cleavage in PC9 and A549 cells. Of interest, **8aa** exhibited significant efficacy in suppressing the EGFR^L858R/T790M^ resistance mutation, which frequently occurs in NSCLC. Molecular docking analysis suggests that this results from a hydrogen bonding interaction between **8aa** and Asp855 of EGFR^L858R/T790M^. Overall, **8aa** has the potential to be developed as a novel EGFR inhibitor to treat NSCLC patients in general, as well as those with L858R and T790M mutations that are resistant to conventional EGFR-TKIs.

## Figures and Tables

**Table 1:** Synthesis of **8** *^a^*.

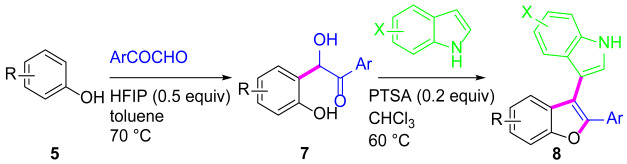				
**Entry**	**7**	**Yield (%) *^b^***	**8**	**Yield (%) *^b^***
1	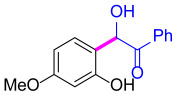	**7a** (95%)	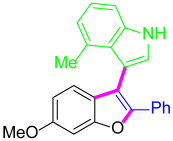	**8b** (97%)
			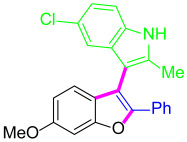	**8c** (96%)
			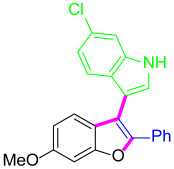	**8d** (98%)
			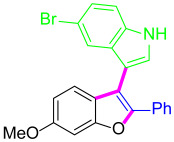	**8e** (92%)
			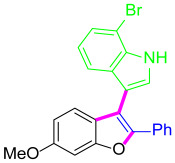	**8f** (69%) *^d^*
			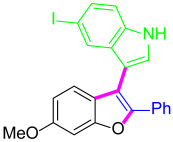	**8g** (94%)
			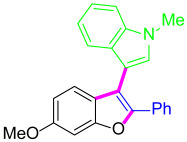	**8h** (54%)
2	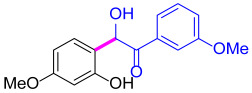	**7b** (96%)	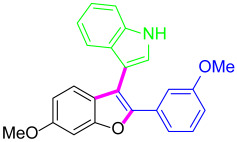	**8i** (65%)
3	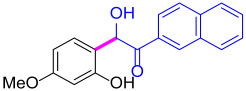	**7c** (64%)	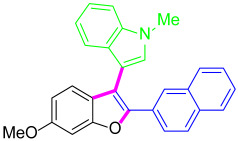	**8j** (65%)
4	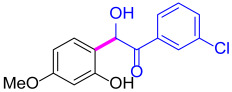	**7d** (89%)	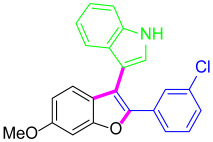	**8k** (87%)
5	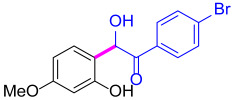	**7e** (65%)	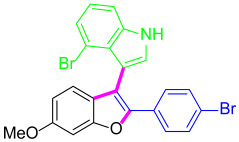	**8l** (75%)
6	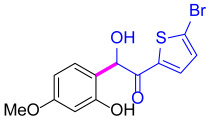	**7f** (81%)	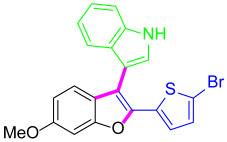	**8m** (54%) *^d^*
7	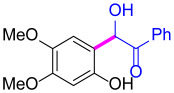	**7g** (98%)	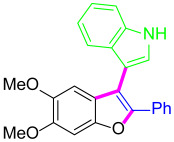	**8n** (80%)
8	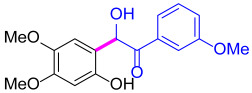	**7h** (81%)	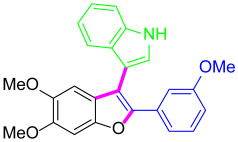	**8o** (89%)
9	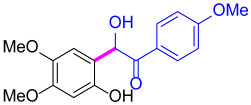	**7i** (95%)	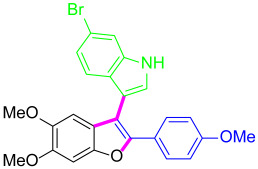	**8p** (81%)
10	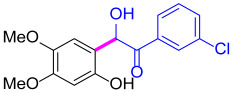	**7j** (94%)	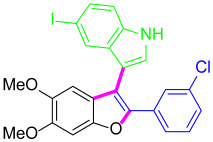	**8q** (99%)
11	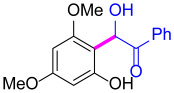	**7k** (62%) *^c^*	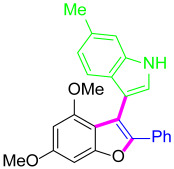	**8r** (80%)
12	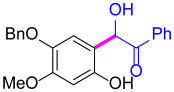	**7l** (60%)	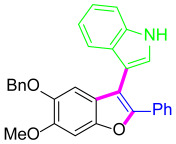	**8s** (99%)
13	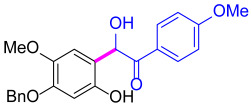	**7m** (42%) *^c^*	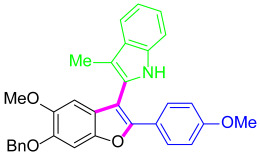	**8t** (65%)
14	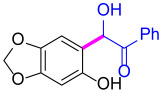	**7n** (80%)	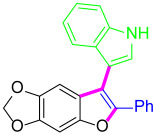	**8u** (68%)
			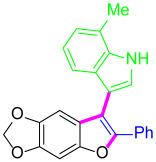	**8v** (92%)
15	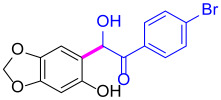	**7o** (70%)	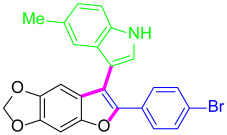	**8w** (89%)
16	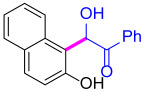	**7p** (92%)	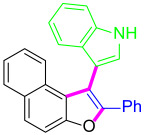	**8x** (88%)
17	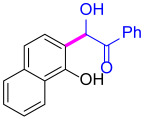	**7q** (80%)	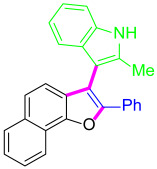	**8y** (62%)

*^a^* A mixture of **5** (2 equiv), arylglyoxal (0.33 mmol, 1 equiv), and HFIP (0.5 equiv) in toluene (4 mL) was stirred at 70 °C for 36 h. A mixture of **7** (0.08 mmol, 1 equiv), indole (1.5 equiv), and PTSA (0.2 equiv) in CHCl_3_ (2 mL) was stirred at 60 °C for 18 h. *^b^* Isolated yield (%). *^c^* Reaction at 60 °C. *^d^* Reaction at rt.

**Table 2:** Inhibitory effects of **8a–y** on cell viability of PC9 cells (mean, *n* = 4).

Compound	IC_50_ (μM)
**8a**	7.53
**8b**	24.67
**8c**	6.3
**8d**	13.38
**8e**	0.56
**8f**	31.98
**8g**	0.85
**8h**	18.59
**8i**	11.33
**8j**	6.33
**8k**	6.76
**8l**	14.99
**8m**	10.61
**8n**	7.38
**8o**	2.44
**8p**	4.04
**8q**	2.24
**8r**	5.59
**8s**	4.46
**8t**	9.58
**8u**	2.32
**8v**	2.11
**8w**	1.58
**8x**	27.86
**8y**	1.58

**Table 3:** Inhibitory effects of **8e** and **8z–ad** on cell viabilities of PC9 and A549 cells (mean, *n* = 6).

Compound	Structure	IC_50_ (μM)	
		PC9	A549
**8e**	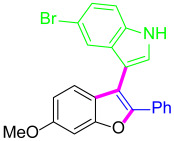	0.50	2.74
**8z**	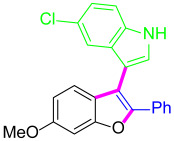	0.58	1.36
**8aa**	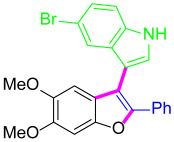	0.32	0.89
**8ab**	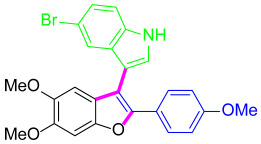	0.45	6.51
**8ac**	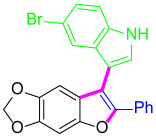	2.42	3.53
**8ad**	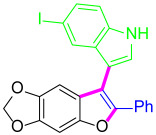	1.65	3.21

## Data Availability

Data is contained within the article and Supplementary Materials.

## Supplementary Materials

The following supporting information can be downloaded at: https://www.mdpi.com/article/10.3390/ph17020231/s1, Supplementary Materials: NMR spectra (^1^H and ^13^C NMR) and HRMS of synthesized compounds and HPLC chromatogram of **8aa**.

## References

[1] Lee Y., Joshi D.R., Namkung W., Kim I. (2022). Generation of a poly-functionalized indolizine scaffold and its anticancer activity in pancreatic cancer cells. Bioorg. Chem..

[2] Nam S., Lee Y., Park S.-H., Namkung W., Kim I. (2022). Synthesis and Biological Evaluation of a Fused Structure of Indolizine and Pyrrolo[1,2-*c*]pyrimidine: Identification of Its Potent Anticancer Activity against Liver Cancer Cells. Pharmaceuticals.

[3] Lee S., Dagar A., Cho I., Kim K., Park I.W., Yoon S., Cha M., Shin J., Kim H.Y., Kim I. (2023). 4-Acyl-3,4-dihydropyrrolo[1,2-*a*]pyrazine Derivative Rescued the Hippocampal-Dependent Cognitive Decline of 5XFAD Transgenic Mice by Dissociating Soluble and Insoluble Aβ Aggregates. ACS Chem. Neurosci..

[4] Gridelli C., Rossi A., Carbone D.P., Guarize J., Karachaliou N., Mok T., Petrella F., Spaggiari L., Rosell R. (2015). Non-small-cell lung cancer. Nat. Rev. Dis. Primers.

[5] Cruz C.S.D., Tanoue L.T., Matthay R.A. (2011). Lung cancer: Epidemiology, etiology, and prevention. Clin. Chest Med..

[6] Fontanini G., Vignati S., Chinè S., Lucchi M., Silvestri V., Mussi A., Placido S.D., Tortora G., Bianco A.R., Gullick W. (1998). Evaluation of epidermal growth factor-related growth factors and receptors and of neoangiogenesis in completely resected stage I-IIIA non-small-cell lung cancer: Amphiregulin and microvessel count are independent prognostic indicators of survival. Clin. Cancer Res..

[7] Bethune G., Bethune D., Ridgway N., Xu Z. (2010). Epidermal growth factor receptor (EGFR) in lung cancer: An overview and update. J. Thorac. Dis..

[8] Gupta R., Dastane A.M., Forozan F., Riley-Portuguez A., Chung F., Lopategui J., Marchevsky A.M. (2009). Evaluation of EGFR abnormalities in patients with pulmonary adenocarcinoma: The need to test neoplasms with more than one method. Mod. Pathol..

[9] Shigematsu H., Gazdar A.F. (2006). Somatic mutations of epidermal growth factor receptor signaling pathway in lung cancers. Int. J. Cancer.

[10] Inamura K., Ninomiya H., Ishikawa Y., Matsubara O. (2010). Is the epidermal growth factor receptor status in lung cancers reflected in clinicopathologic features?. Arch. Pathol. Lab. Med..

[11] Massarelli E., Varella-Garcia M., Tang X., Xavier A.C., Ozburn N.C., Liu D.D., Bekele B.N., Herbst R.S., Wistuba I.I. (2007). KRAS mutation is an important predictor of resistance to therapy with epidermal growth factor receptor tyrosine kinase inhibitors in non–small-cell lung cancer. Clin. Cancer Res..

[12] Gerber D.E. (2008). EGFR inhibition in the treatment of non-small cell lung cancer. Drug Dev. Res..

[13] Westover D., Zugazagoitia J., Cho B., Lovly C., Paz-Ares L. (2018). Mechanisms of acquired resistance to first-and second-generation EGFR tyrosine kinase inhibitors. Ann. Oncol..

[14] Lüth A., Löwe W. (2008). Syntheses of 4-(indole-3-yl)quinazolines–A new class of epidermal growth factor receptor tyrosine kinase inhibitors. Eur. J. Med. Chem..

[15] Park S., Kim E.H., Kim J., Kim S.H., Kim I. (2018). Biological evaluation of indolizine-chalcone hybrids as new anticancer agents. Eur. J. Med. Chem..

[16] Seo Y., Lee J.H., Park S.-H., Namkung W., Kim I. (2020). Expansion of chemical space based on a pyrrolo[1,2-*a*]pyrazine core: Synthesis and its anticancer activity in prostate cancer and breast cancer cells. Eur. J. Med. Chem..

[17] Nevagi R.J., Dighe S.N., Dighe S.N. (2015). Biological and medicinal significance of benzofuran. Eur. J. Med. Chem..

[18] Heravi M.M., Amiri Z., Kafshdarzadeh K., Zadsirjan V. (2021). Synthesis of indole derivatives as prevalent moieties present in selected alkaloids. RSC Adv..

[19] Abbas H.-A.S., El-Karim S.S.A. (2019). Design, synthesis and anticervical cancer activity of new benzofuran–pyrazol-hydrazono-thiazolidin-4-one hybrids as potential EGFR inhibitors and apoptosis inducing agents. Bioorg. Chem..

[20] Mphahlele M.J., Maluleka M.M., Parbhoo N., Malindisa S.T. (2018). Synthesis, evaluation for cytotoxicity and molecular docking studies of benzo[*c*]furan-chalcones for potential to inhibit tubulin polymerization and/or EGFR-tyrosine kinase phosphorylation. Int. J. Mol. Sci..

[21] Sheng J., Liu Z., Yan M., Zhang X., Wang D., Xu J., Zhang E., Zou Y. (2017). Biomass-involved synthesis of *N*-substituted benzofuro[2,3-*d*]pyrimidine-4-amines and biological evaluation as novel EGFR tyrosine kinase inhibitors. Org. Biomol. Chem..

[22] Al-Wahaibi L.H., Mostafa Y.A., Abdelrahman M.H., El-Bahrawy A.H., Trembleau L., Youssif B.G. (2022). Synthesis and Biological Evaluation of Indole-2-Carboxamides with Potent Apoptotic Antiproliferative Activity as EGFR/CDK2 Dual Inhibitors. Pharm. Res..

[23] Al-Wahaibi L.H., Mohammed A.F., Abdelrahman M.H., Trembleau L., Youssif B.G. (2023). Design, Synthesis, and Biological Evaluation of Indole-2-carboxamides as Potential Multi-Target Antiproliferative Agents. Pharm. Res..

[24] Eftekhari-Sis B., Zirak M., Akbari A. (2013). Arylglyoxals in synthesis of heterocyclic compounds. Chem. Rev..

[25] Jung E., Jeong Y., Kim H., Kim I. (2023). C3 Functionalization of Indolizines via HFIP-Promoted Friedel–Crafts Reactions with (Hetero)arylglyoxals. ACS Omega.

[26] Cheng C., Liu C., Gu Y. (2015). One-pot three-component reactions of methyl ketones, phenols and a nucleophile: An expedient way to synthesize densely substituted benzofurans. Tetrahedron.

[27] Ramalingam S.S., Vansteenkiste J., Planchard D., Cho B.C., Gray J.E., Ohe Y., Zhou C., Reungwetwattana T., Cheng Y., Chewaskulyong B. (2020). Overall survival with osimertinib in untreated, EGFR-mutated advanced NSCLC. N. Engl. J. Med..

[28] Rolfo C., Giovannetti E., Hong D.S., Bivona T., Raez L.E., Bronte G., Buffoni L., Reguart N., Santos E.S., Germonpre P. (2014). Novel therapeutic strategies for patients with NSCLC that do not respond to treatment with EGFR inhibitors. Cancer Treat. Rev..

[29] Phillips J.C., Braun R., Wang W., Gumbart J., Tajkhorshid E., Villa E., Chipot C., Skeel R.D., Kale L., Schulten K. (2005). Scalable molecular dynamics with NAMD. J. Comput. Chem..

[30] Goel S., Hidalgo M., Perez-Soler R. (2007). EGFR inhibitor-mediated apoptosis in solid tumors. J. Exp. Ther. Oncol..

[31] Okamoto K., Okamoto I., Okamoto W., Tanaka K., Takezawa K., Kuwata K., Yamaguchi H., Nishio K., Nakagawa K. (2010). Role of survivin in EGFR inhibitor–induced apoptosis in non–small cell lung cancers positive for EGFR mutations. Cancer Res..

[32] Johnson B.E., Jänne P.A. (2005). Epidermal growth factor receptor mutations in patients with non–small cell lung cancer. Cancer Res..

[33] Paez J.G., Jänne P.A., Lee J.C., Tracy S., Greulich H., Gabriel S., Herman P., Kaye F.J., Lindeman N., Boggon T.J. (2004). EGFR mutations in lung cancer: Correlation with clinical response to gefitinib therapy. Science.

[34] Sharma S.V., Bell D.W., Settleman J., Haber D.A. (2007). Epidermal growth factor receptor mutations in lung cancer. Nat. Rev. Cancer.

[35] Hong W., Wu Q., Zhang J., Zhou Y. (2019). Prognostic value of EGFR 19-del and 21-L858R mutations in patients with non-small cell lung cancer. Oncol. Lett..

[36] Yu H.A., Arcila M.E., Rekhtman N., Sima C.S., Zakowski M.F., Pao W., Kris M.G., Miller V.A., Ladanyi M., Riely G.J. (2013). Analysis of tumor specimens at the time of acquired resistance to EGFR-TKI therapy in 155 patients with EGFR-mutant lung cancers. Clin. Cancer Res..

