# Supplementary material for: Anticancer Evaluation of Novel Benzofuran–Indole Hybrids as Epidermal Growth Factor Receptor Inhibitors against Non-Small-Cell Lung Cancer Cells
Source: Pharmaceuticals (Basel). 2024 Feb 9;17(2):231. doi: 10.3390/ph17020231 (PMC10893492; doi:10.3390/ph17020231)
Supplement: Supplementary file 1 [file pharmaceuticals-17-00231-s001.zip › pharmaceuticals-2843608-supplementary.pdf]

Supplementary Materials

Anticancer Evaluation of Novel Benzofuran-Indole Hybrids as EGFR Inhibitors Against Non-Small Cell Lung Cancer Cells

Table of Contents

Copies of ^{1}H and ^{13}C NMR spectra of **7** **S3-19**

Copies of ^{1}H and ^{13}C NMR spectra of **8** **S20-49**

Copies of HRMS spectra of **7** **S50-58**

Copies of HRMS spectra of **8** **S59-73**

HPLC Chromatogram of **8aa** **S74-75**

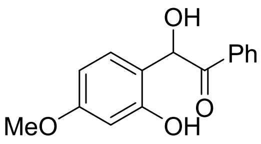

7a

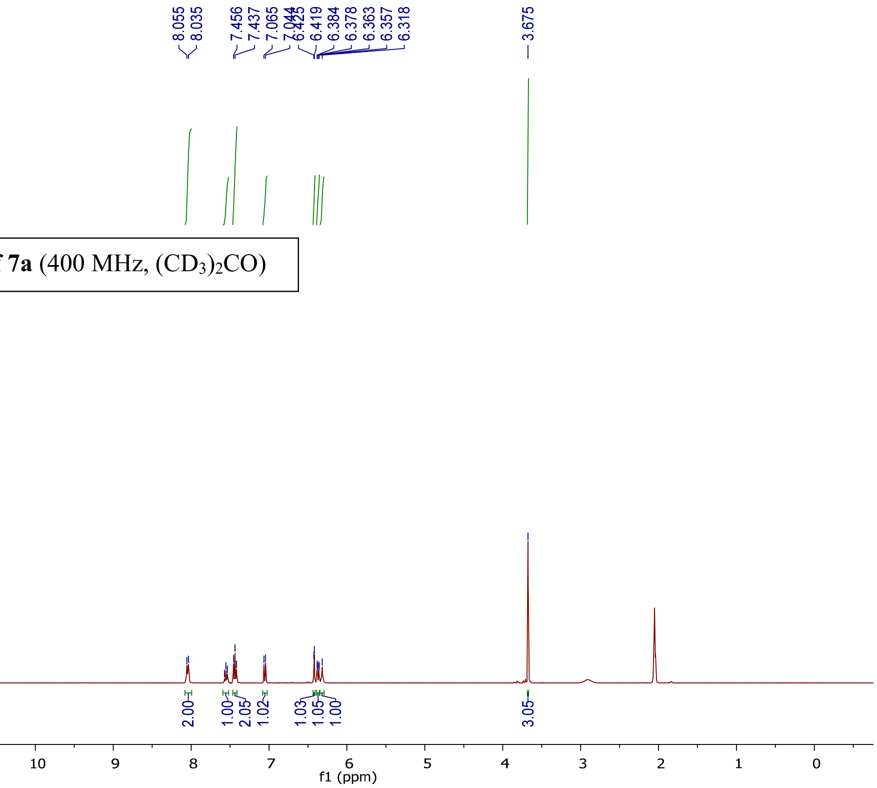

^{1}H NMR spectrum of 7a (400 MHz, $(CD_3)_2CO$)

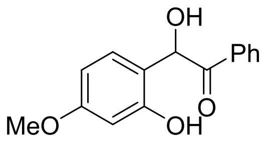

7a

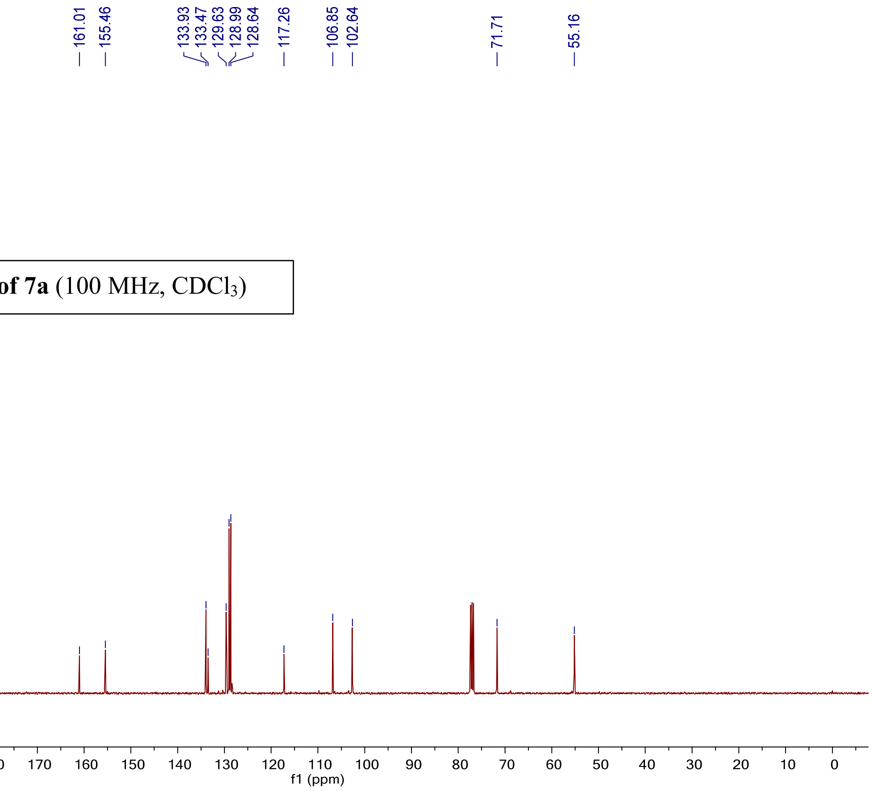

^{13}C NMR spectrum of 7a (100 MHz, $CDCl_3$)

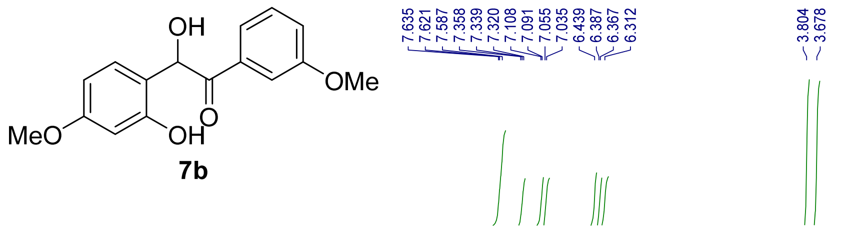

^{1}H NMR spectrum of 7b (400 MHz, $(CD_3)_2CO$)

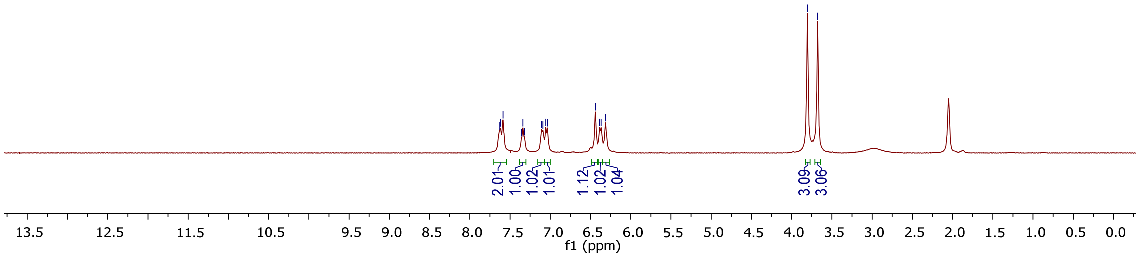

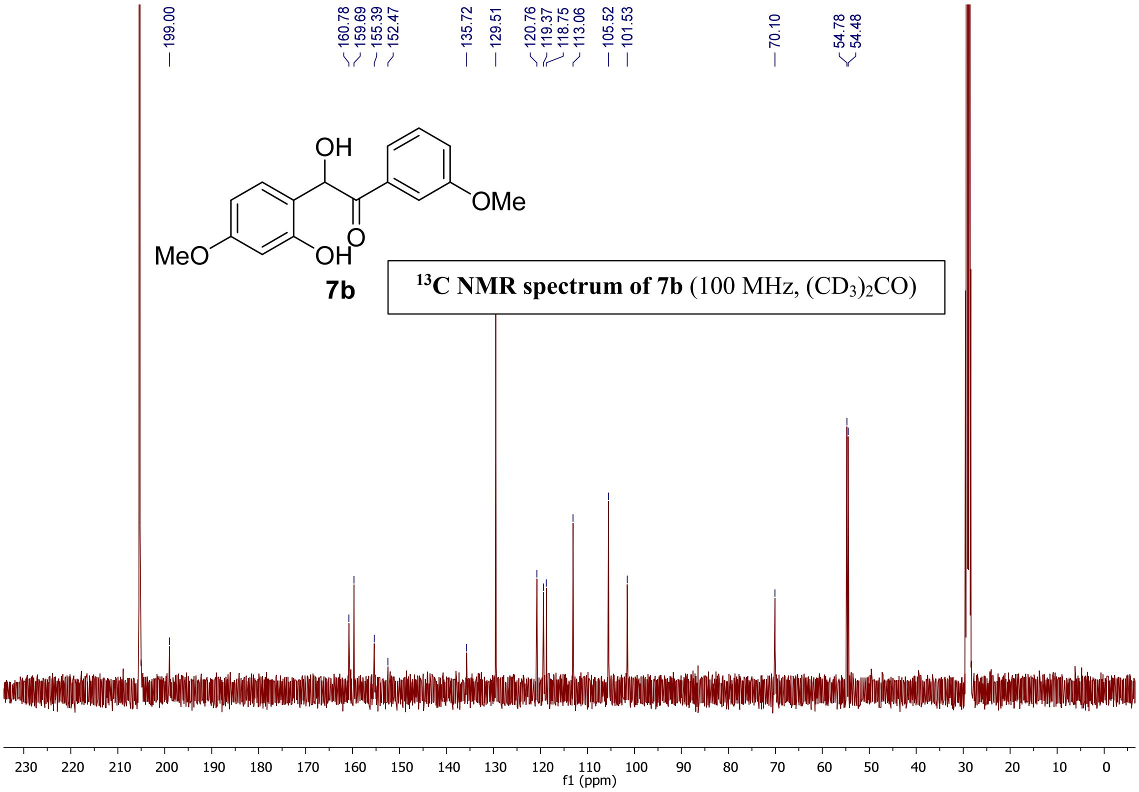

^{13}C NMR spectrum of 7b (100 MHz, $(CD_3)_2CO$)

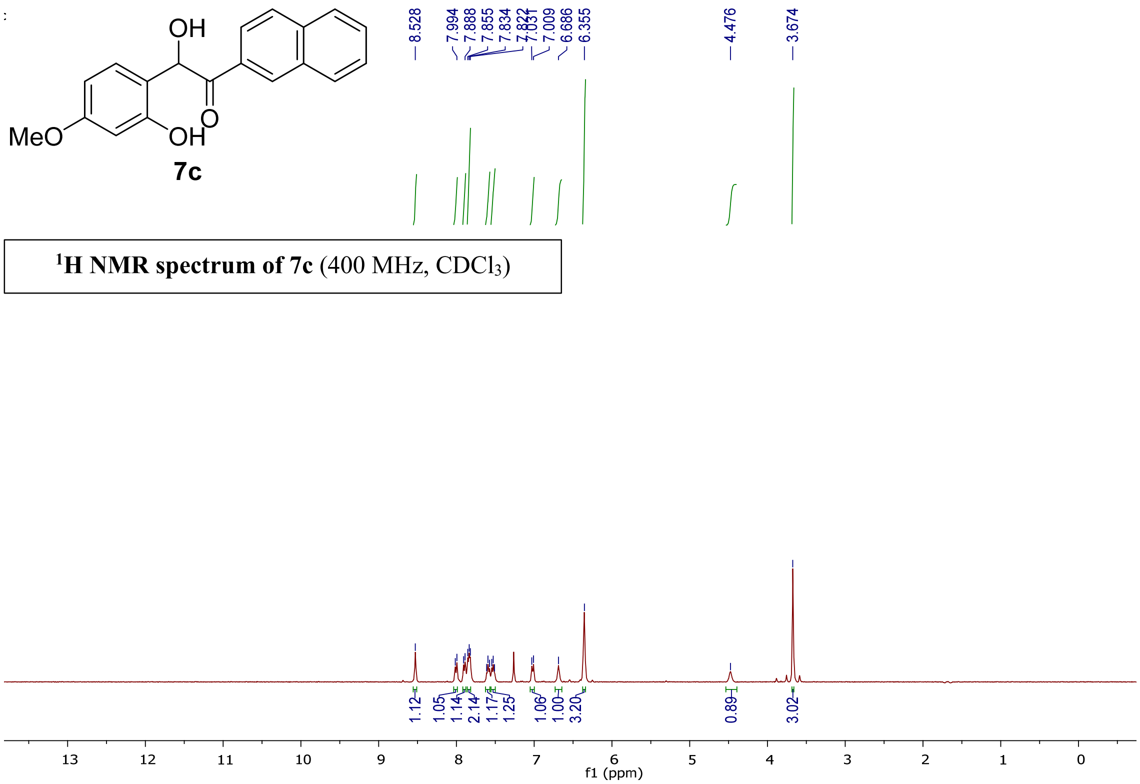

7c

^{1}H NMR spectrum of 7c (400 MHz, $CDCl_3$)

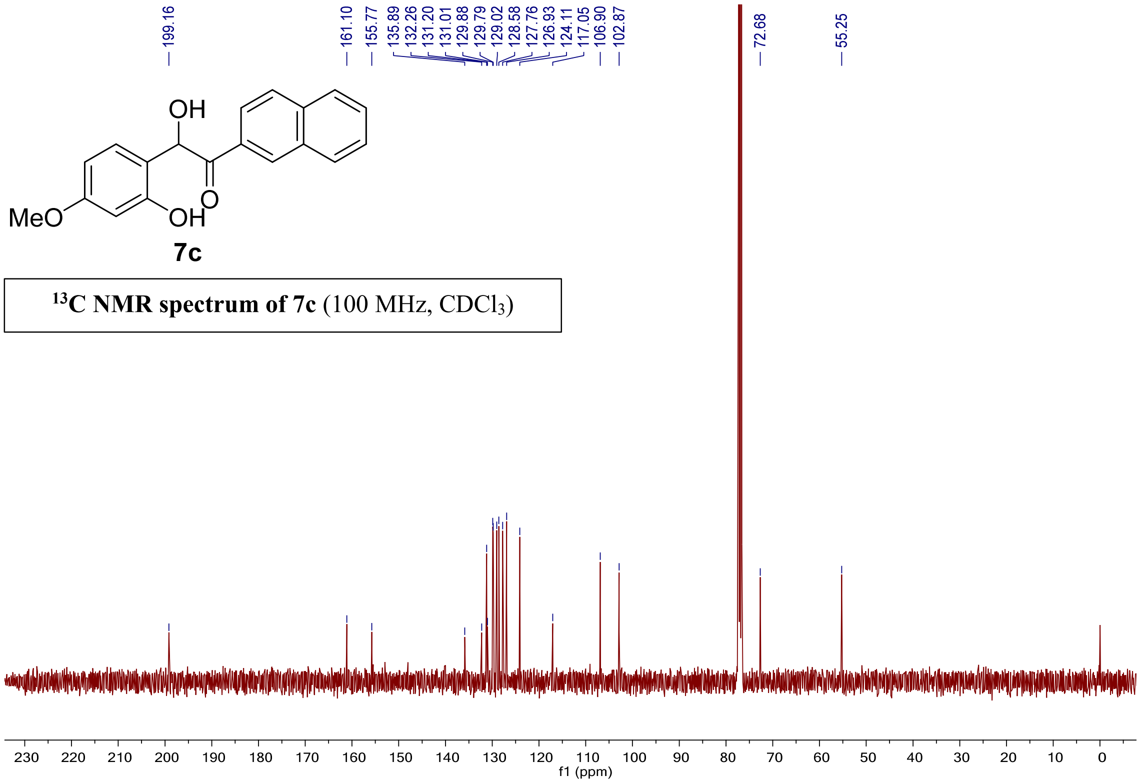

7c

^{13}C NMR spectrum of 7c (100 MHz, $CDCl_3$)

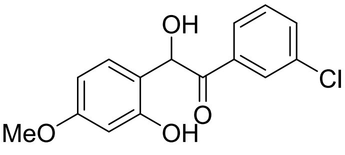

7d

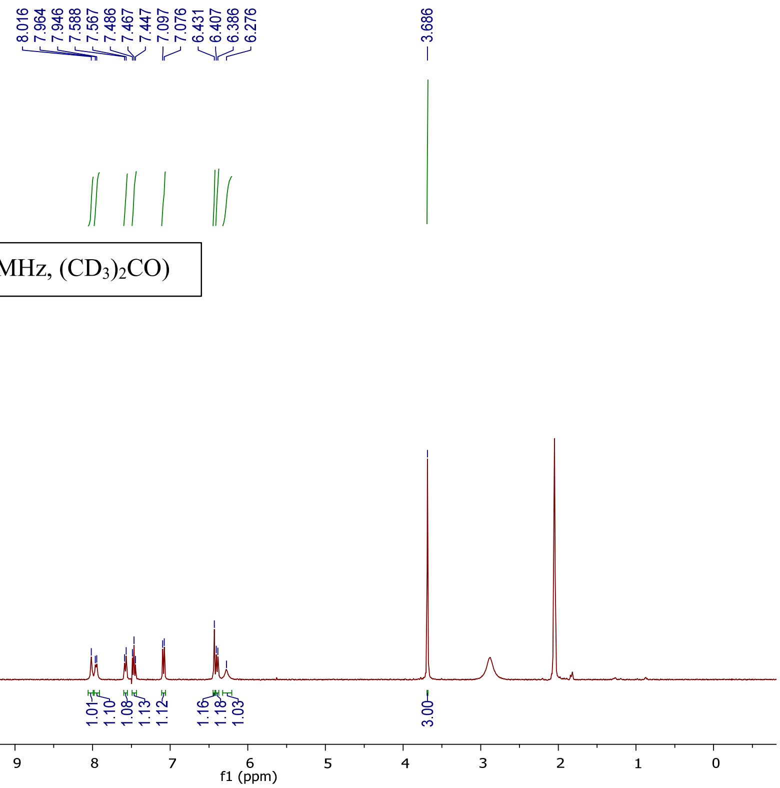

^{1}H NMR spectrum of 7d (400 MHz, $(CD_3)_2CO$)

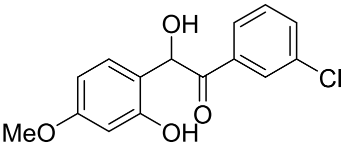

7d

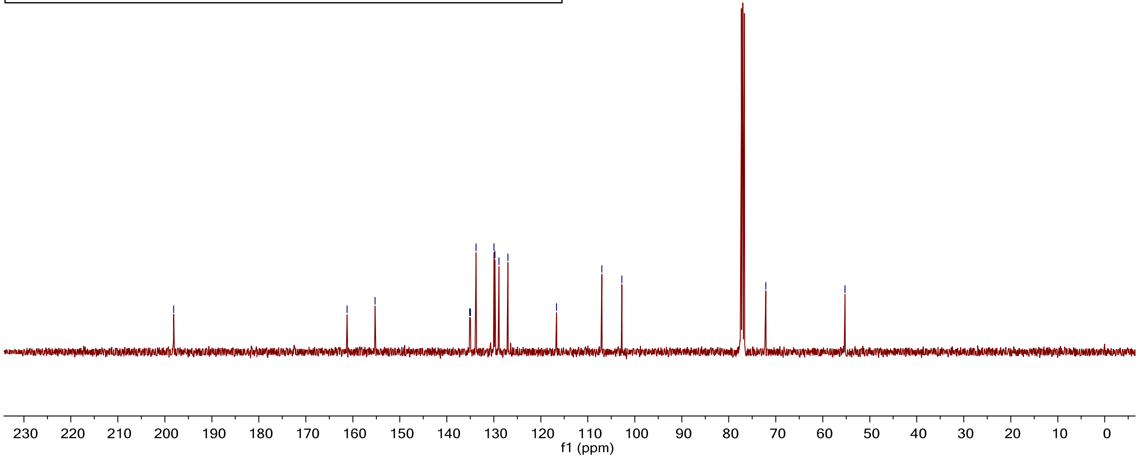

^{13}C NMR spectrum of 7d (100 MHz, $CDCl_3$)

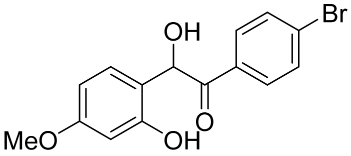

7e

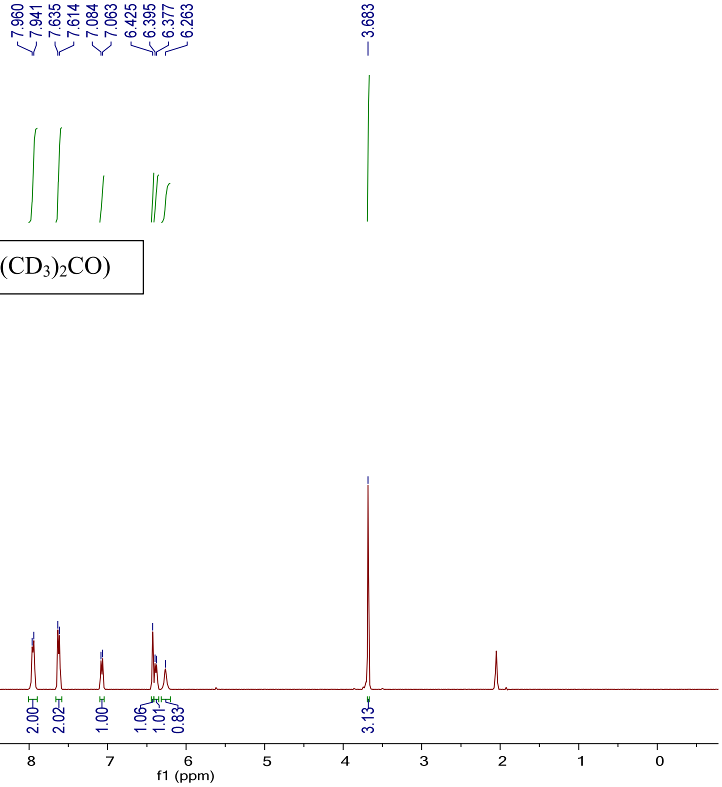

^{1}H NMR spectrum of 7e (400 MHz, $(CD_3)_2CO$)

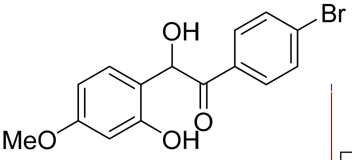

7e

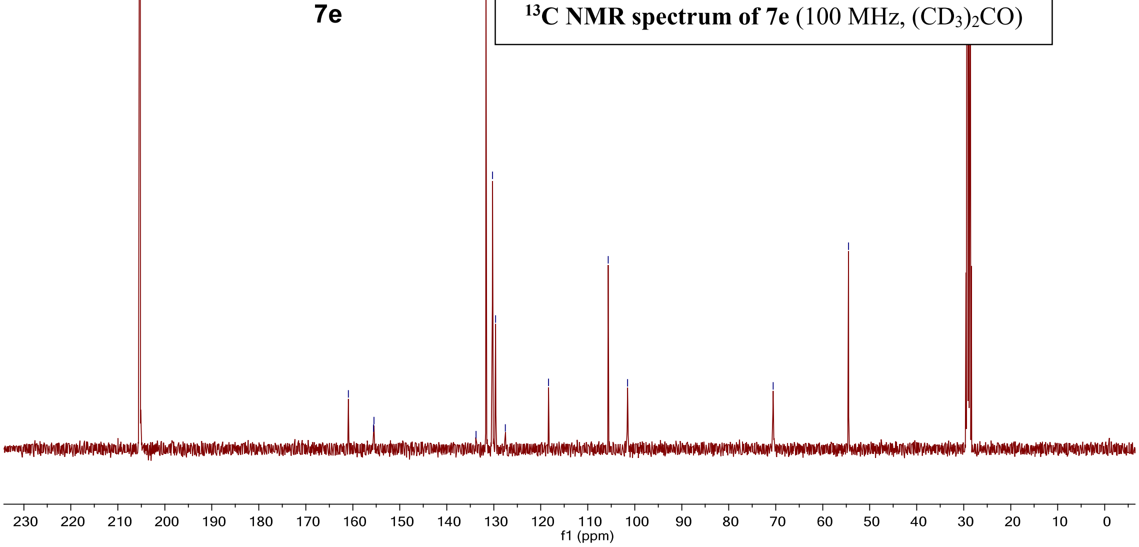

^{13}C NMR spectrum of 7e (100 MHz, $(CD_3)_2CO$)

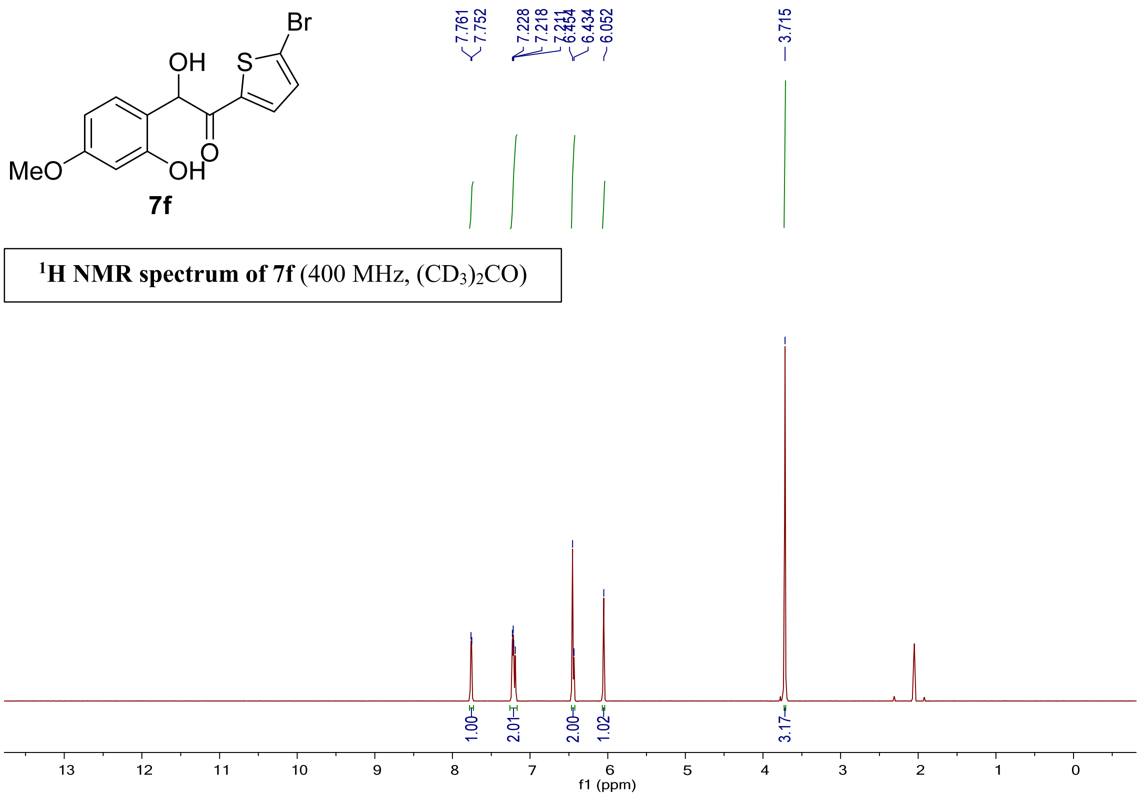

^{1}H NMR spectrum of 7f (400 MHz, $(CD_3)_2CO$)

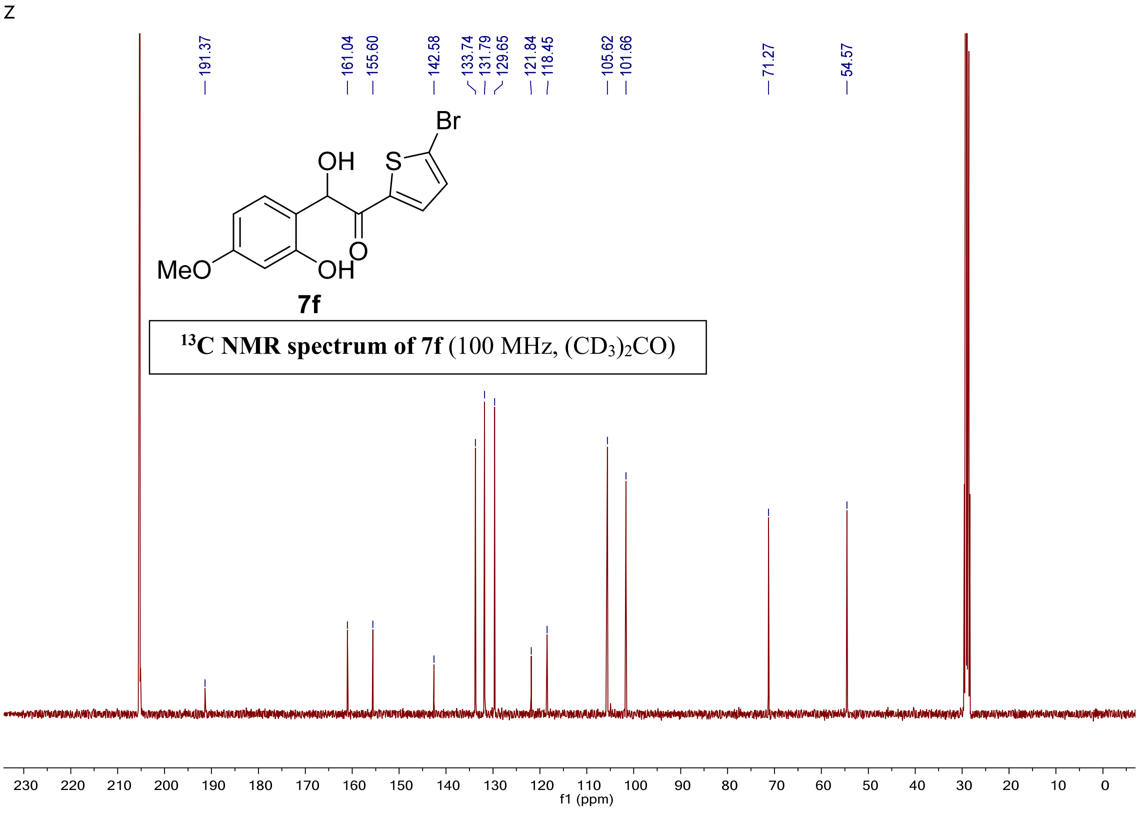

^{13}C NMR spectrum of 7f (100 MHz, $(CD_3)_2CO$)

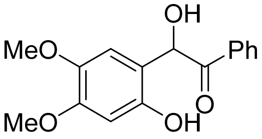

7g

^{1}H NMR spectrum of 7g (400 MHz, $CDCl_3$)

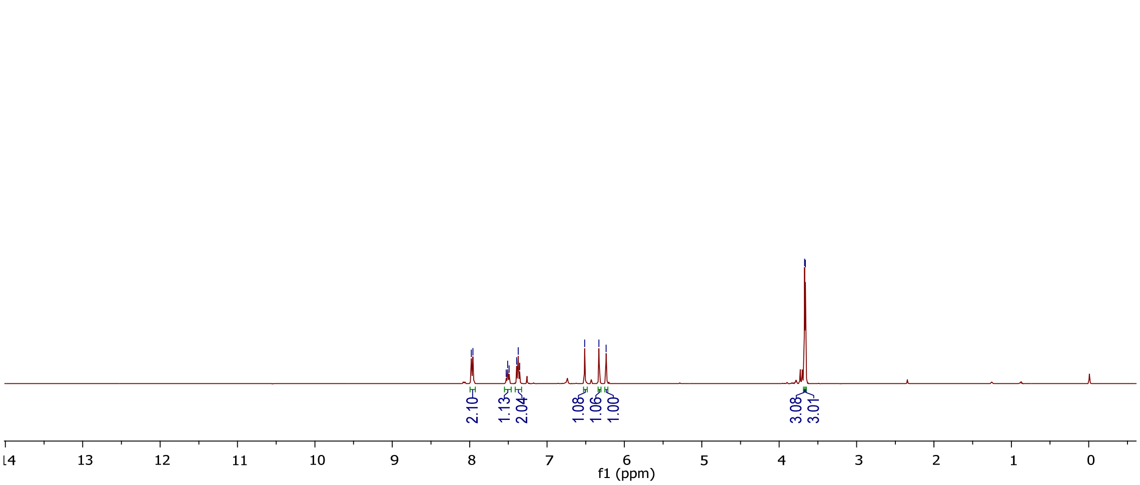

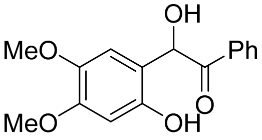

7g

^{13}C NMR spectrum of 7g (100 MHz, $CDCl_3$)

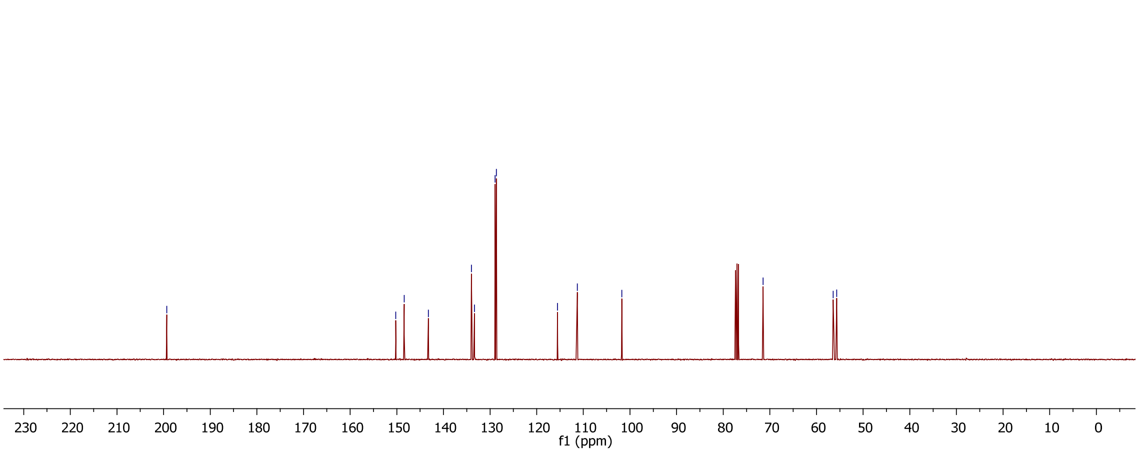

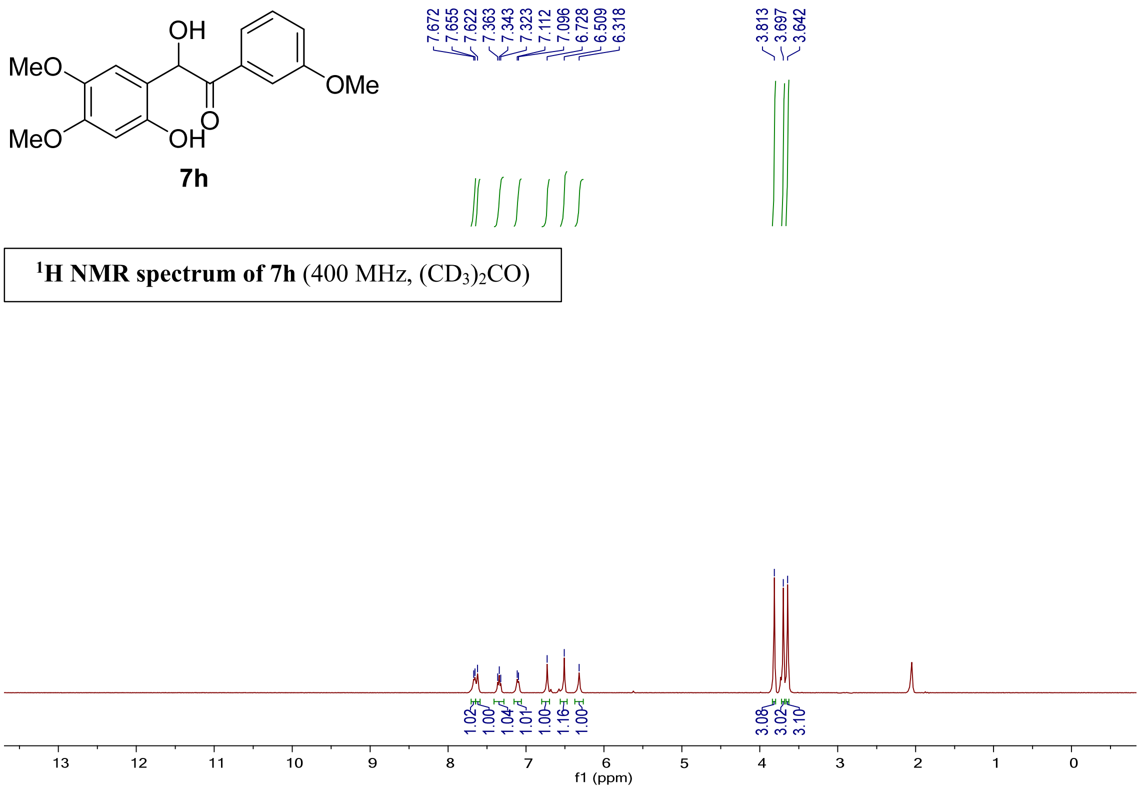

^{1}H NMR spectrum of 7h (400 MHz, $(CD_3)_2CO$)

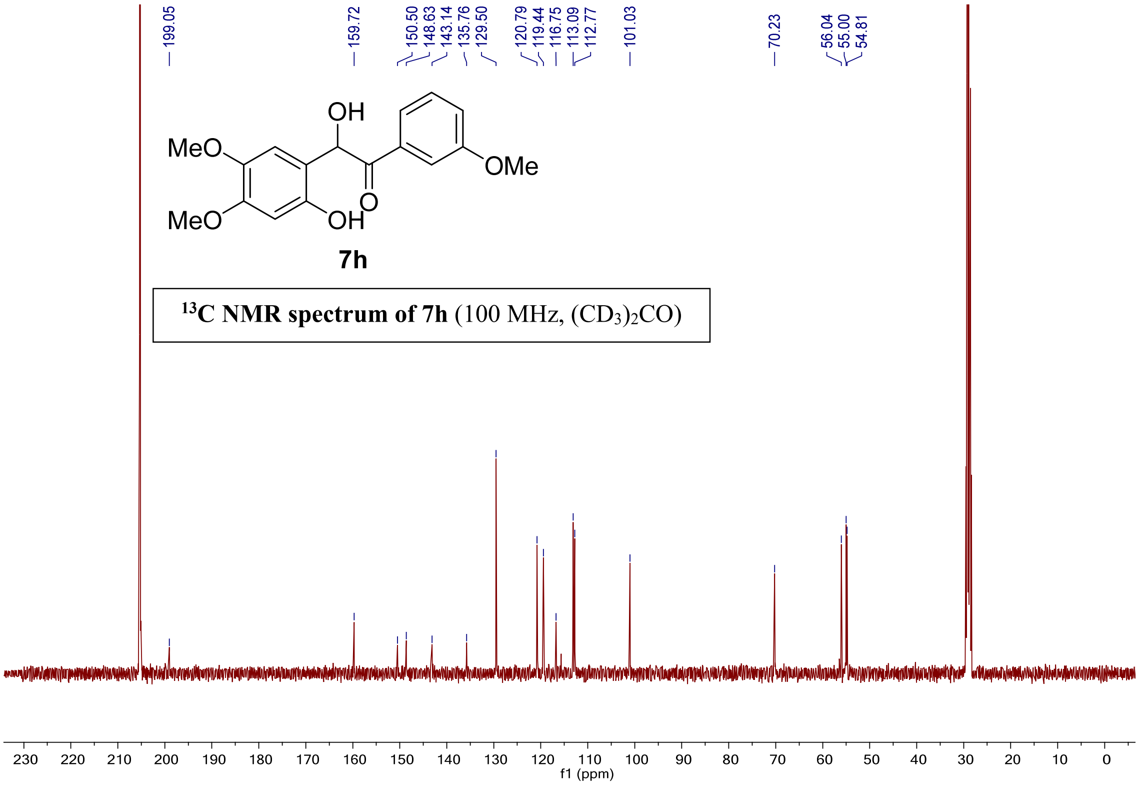

^{13}C NMR spectrum of 7h (100 MHz, $(CD_3)_2CO$)

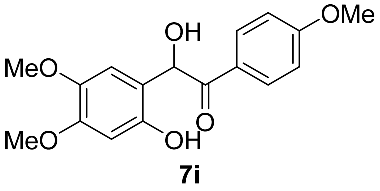

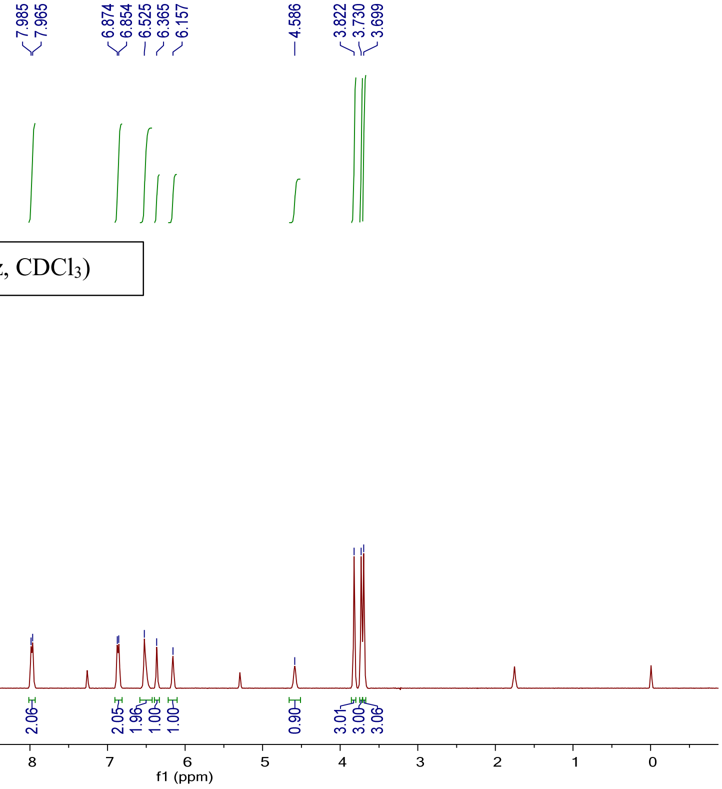

^{1}H NMR spectrum of 7i (400 MHz, $CDCl_3$)

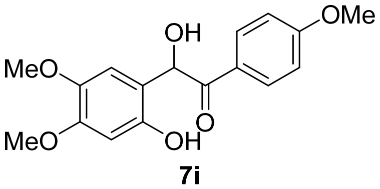

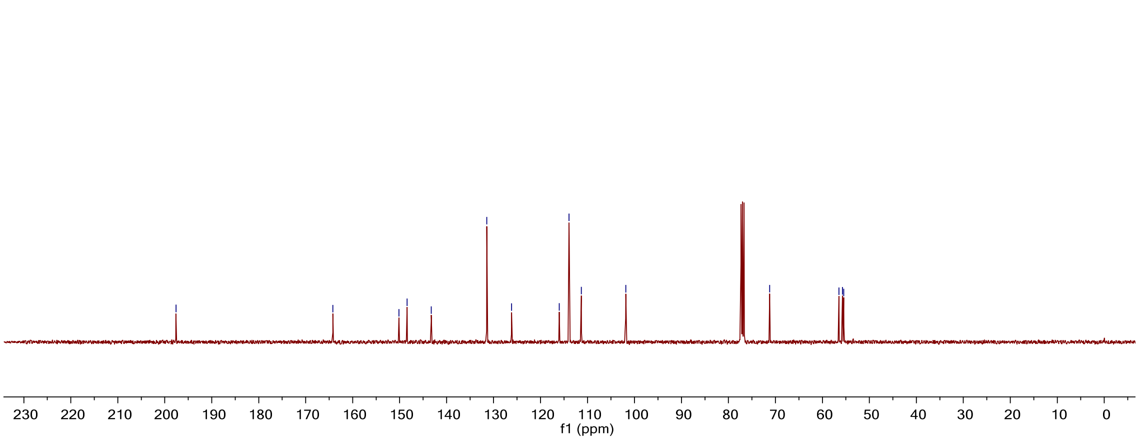

^{13}C NMR spectrum of 7i (100 MHz, $CDCl_3$)

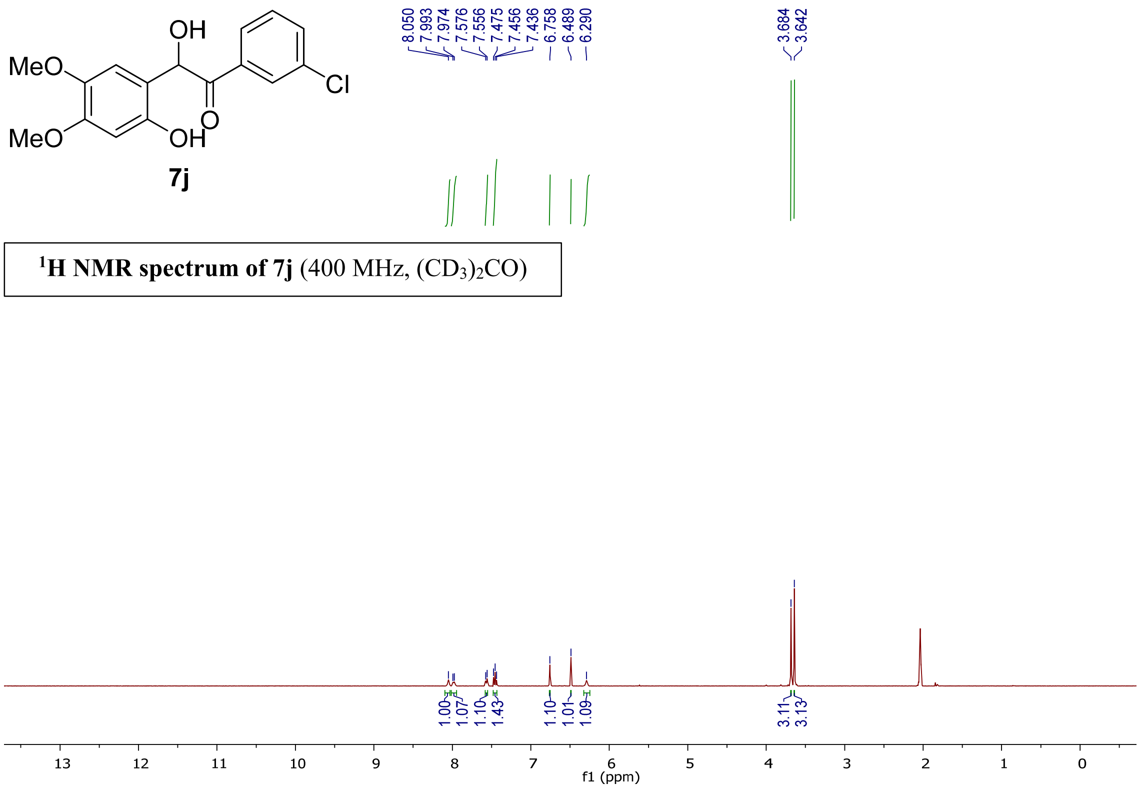

^{1}H NMR spectrum of 7j (400 MHz, $(CD_3)_2CO$)

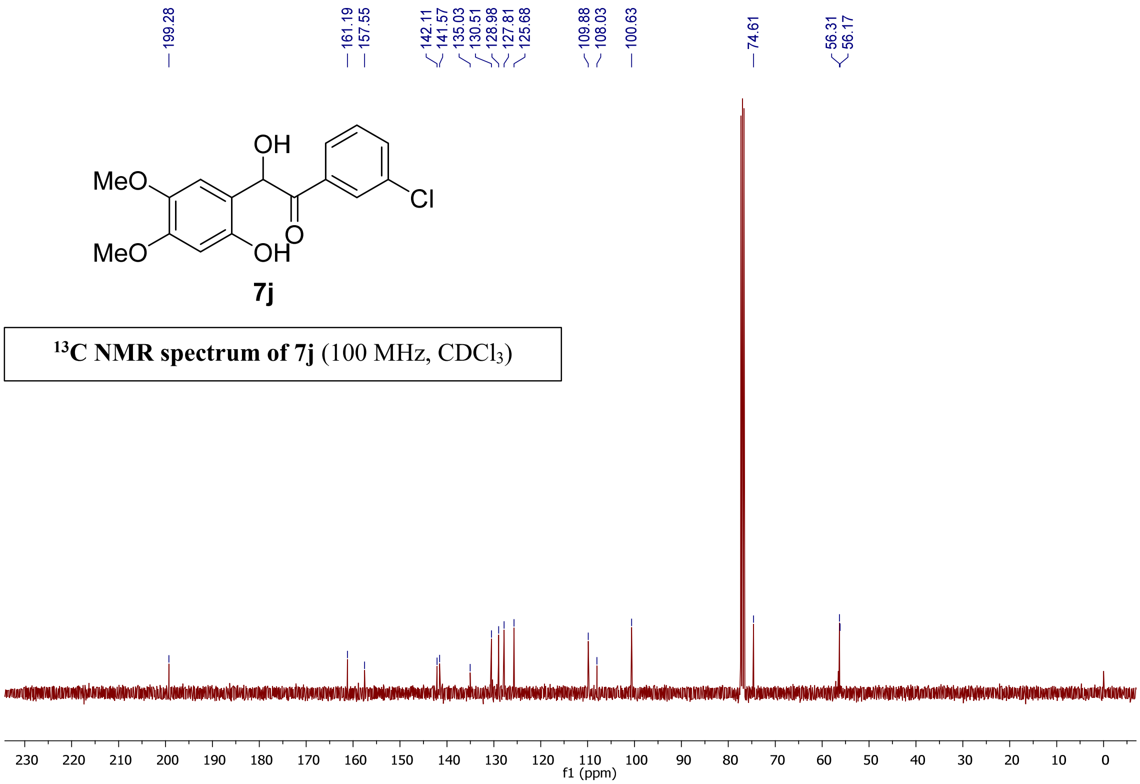

^{13}C NMR spectrum of 7j (100 MHz, $CDCl_3$)

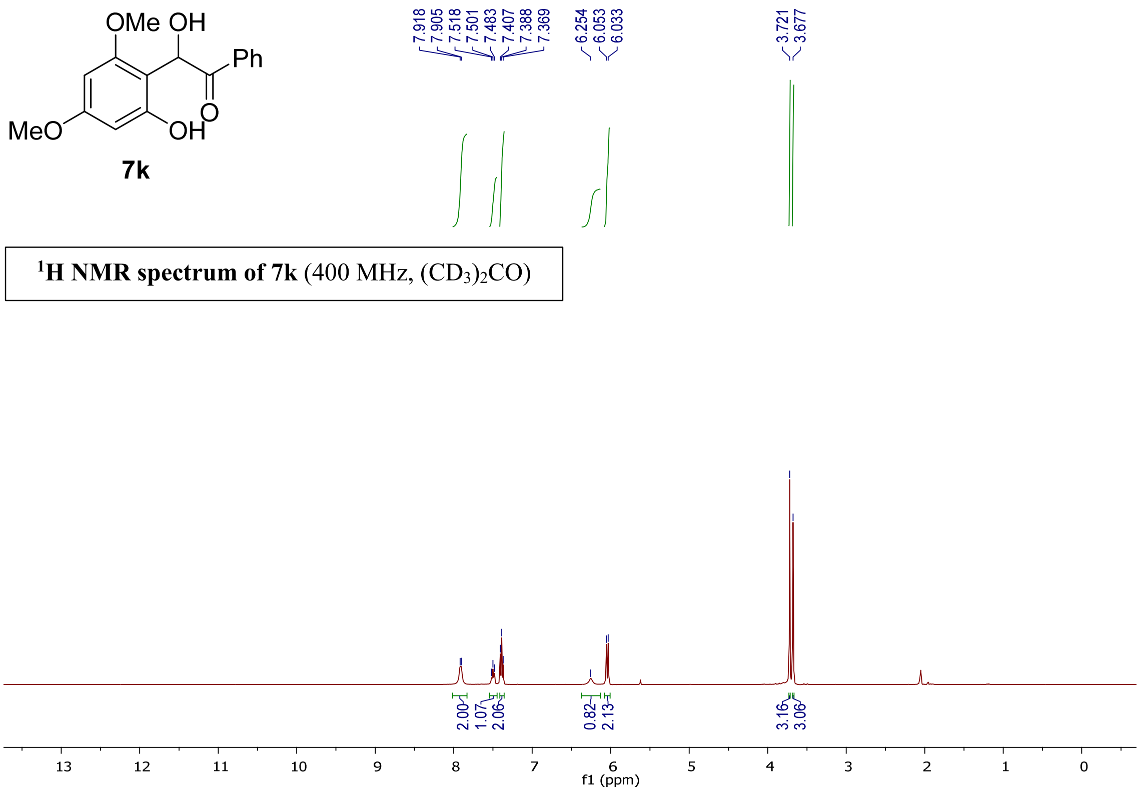

^{1}H NMR spectrum of 7k (400 MHz, $(CD_3)_2CO$)

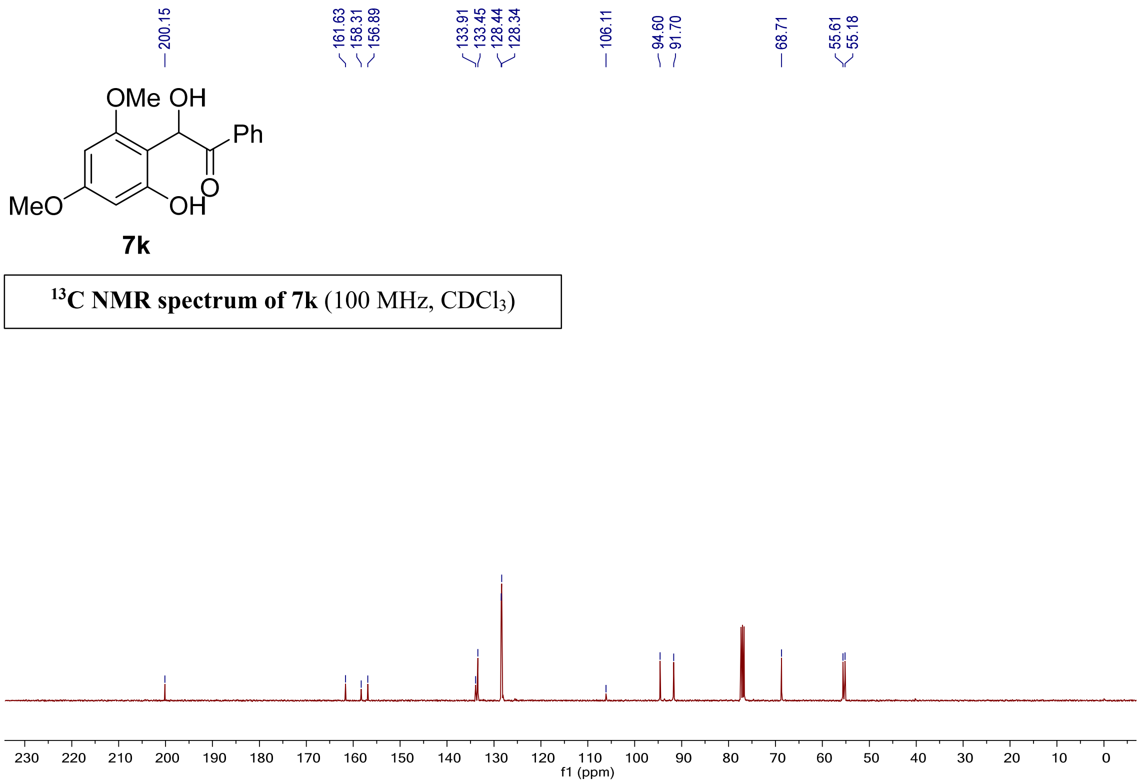

^{13}C NMR spectrum of 7k (100 MHz, $CDCl_3$)

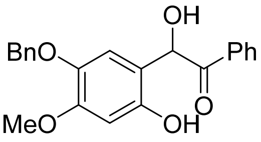

7l

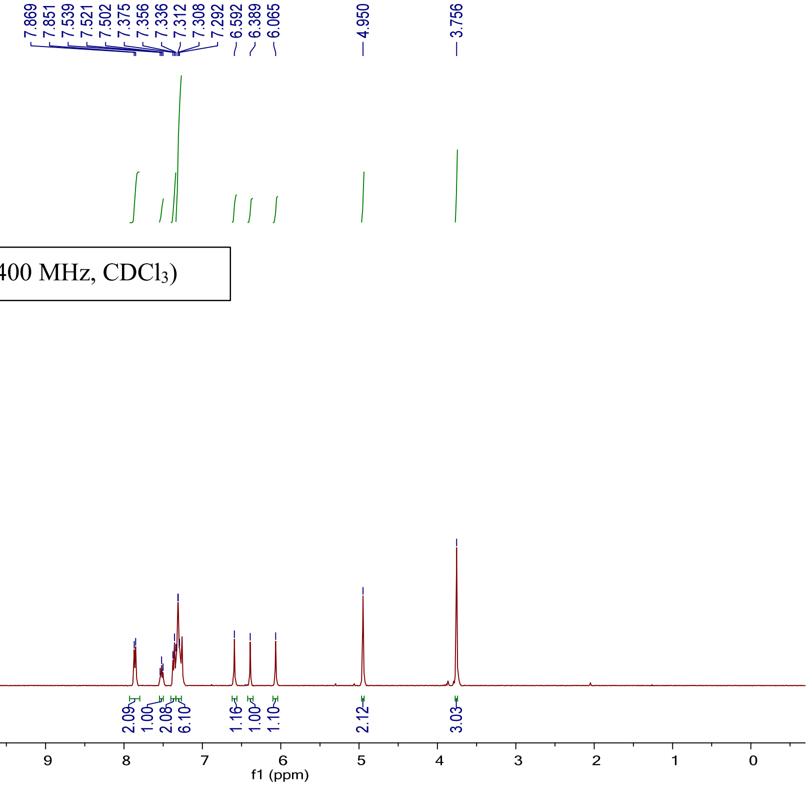

^{1}H NMR spectrum of 7l (400 MHz, $CDCl_3$)

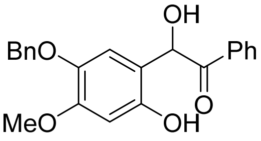

7l

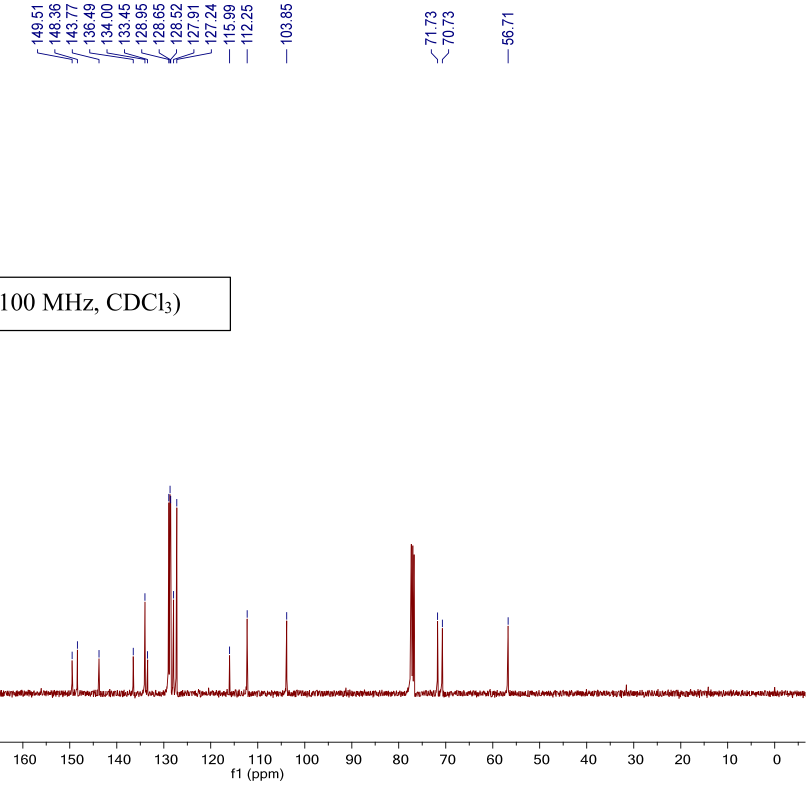

^{13}C NMR spectrum of 7l (100 MHz, $CDCl_3$)

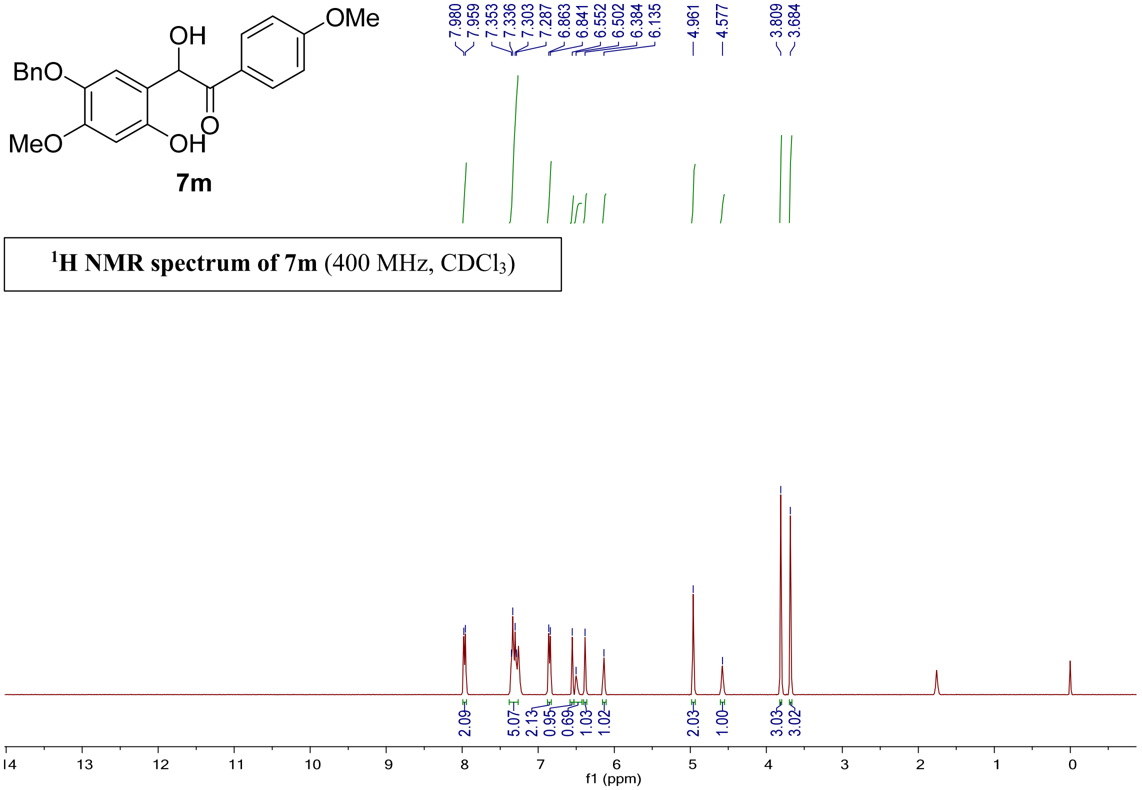

7m

^{1}H NMR spectrum of 7m (400 MHz, $CDCl_3$)

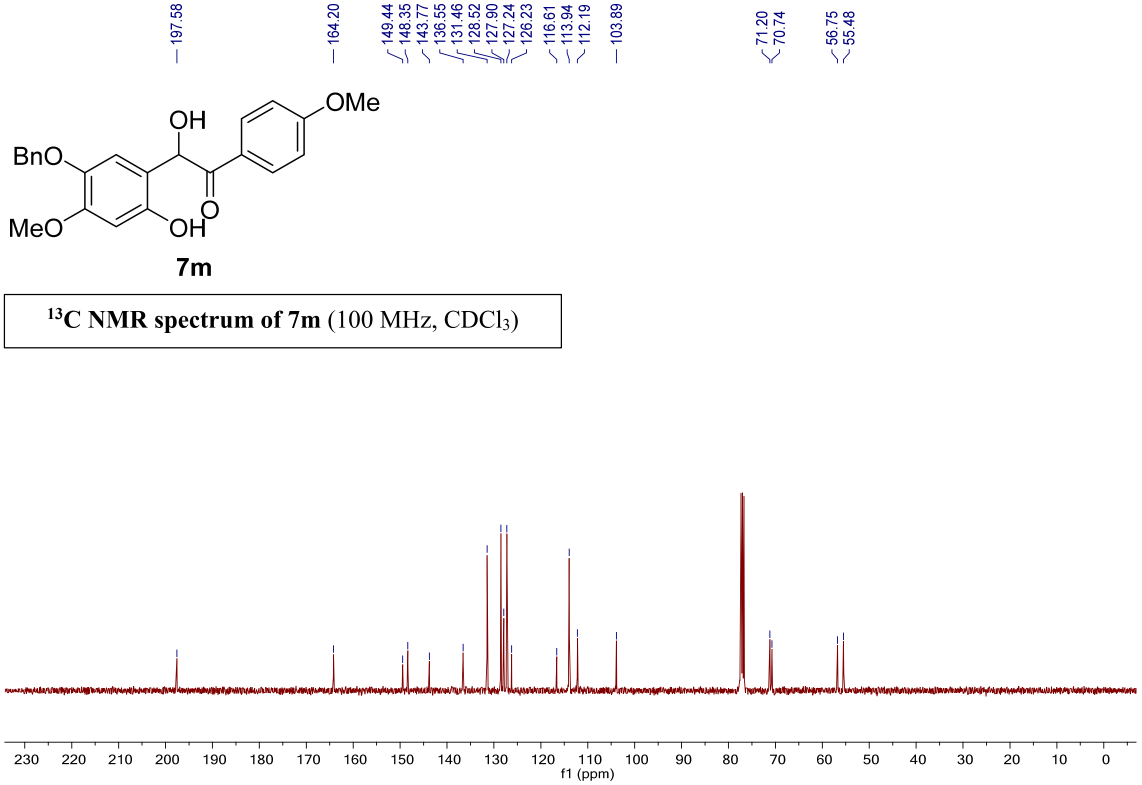

7m

^{13}C NMR spectrum of 7m (100 MHz, $CDCl_3$)

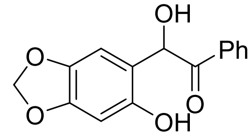

7n

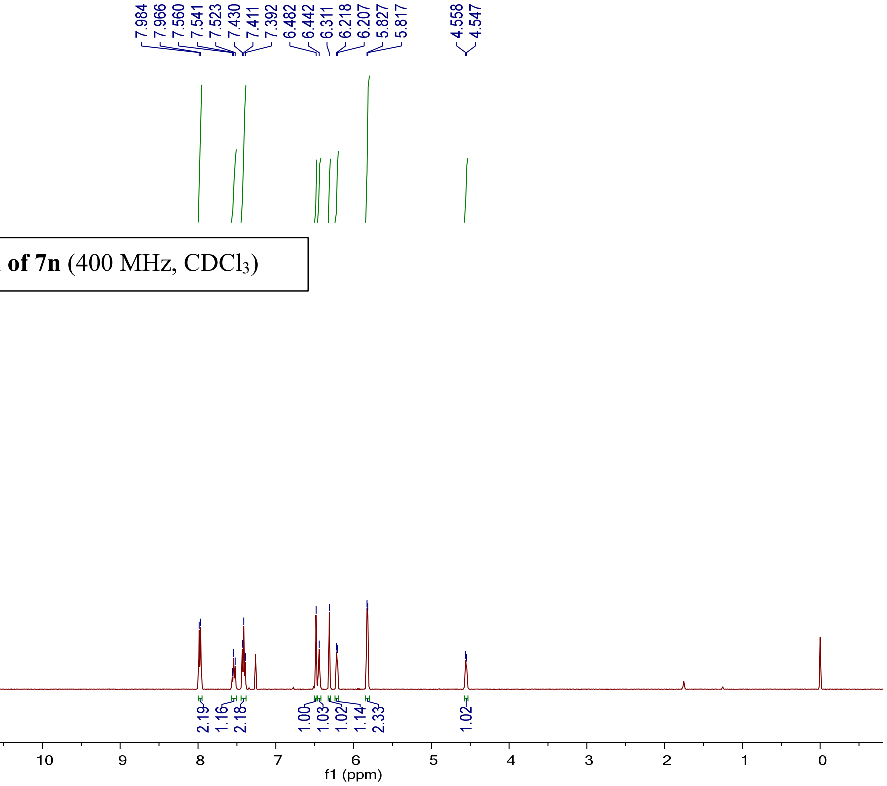

^{1}H NMR spectrum of 7n (400 MHz, $CDCl_3$)

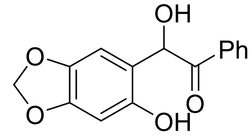

7n

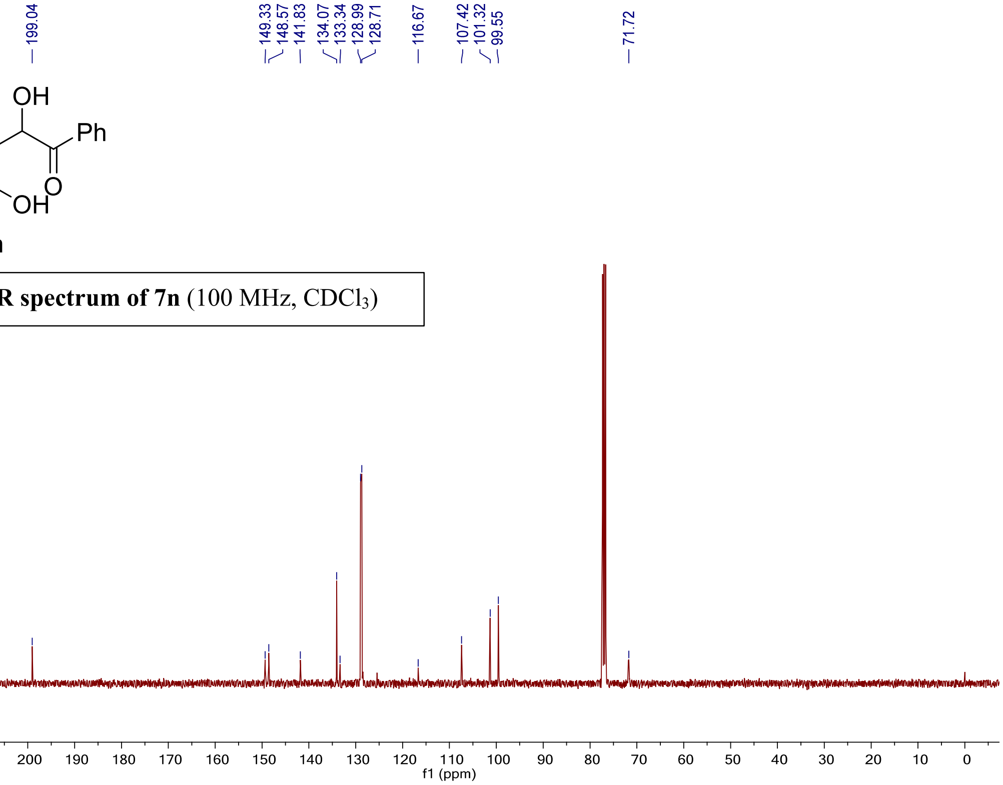

^{13}C NMR spectrum of 7n (100 MHz, $CDCl_3$)

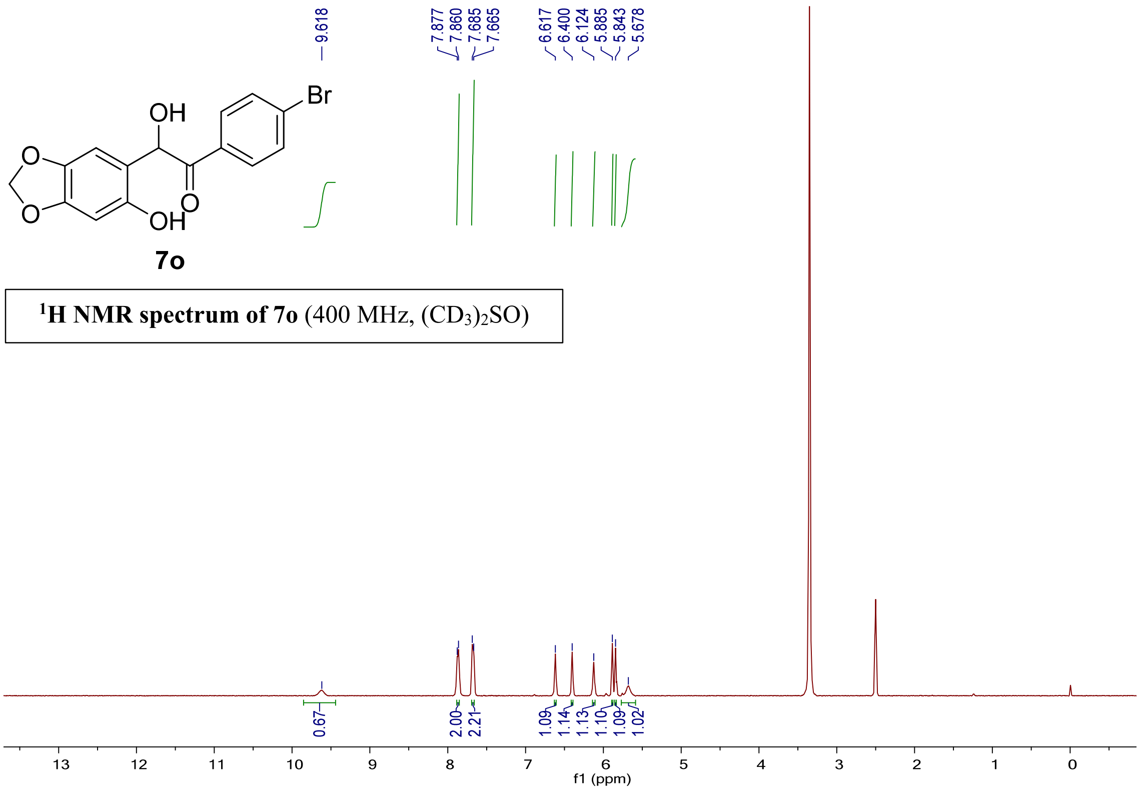

^{1}H NMR spectrum of 7o (400 MHz, $(CD_3)_2SO$)

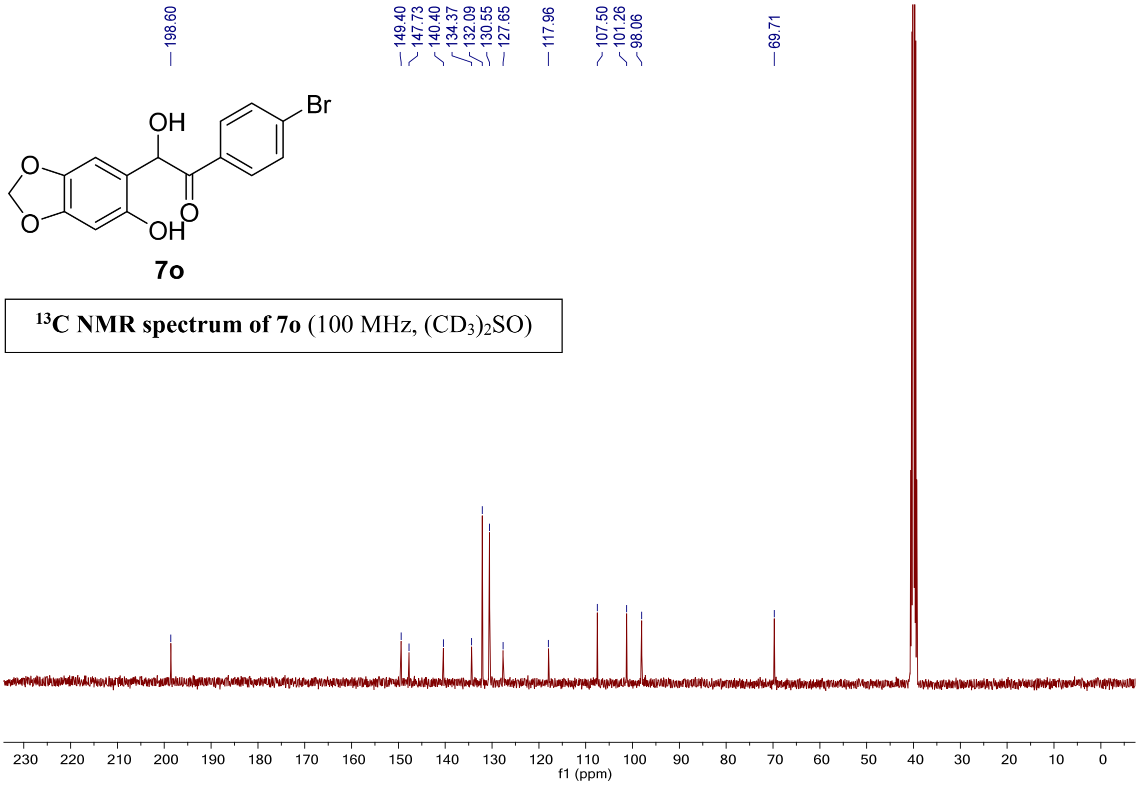

^{13}C NMR spectrum of 7o (100 MHz, $(CD_3)_2SO$)

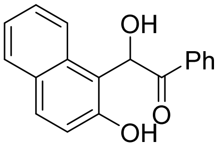

7p

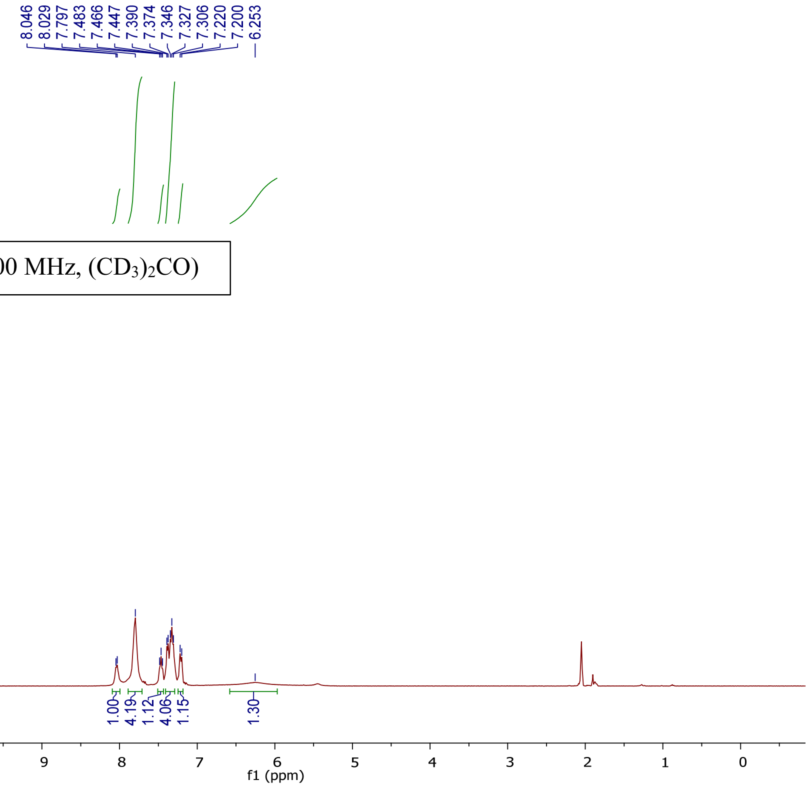

^{1}H NMR spectrum of 7p (400 MHz, $(CD_3)_2CO$)

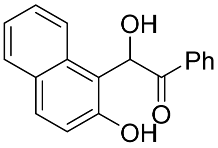

7p

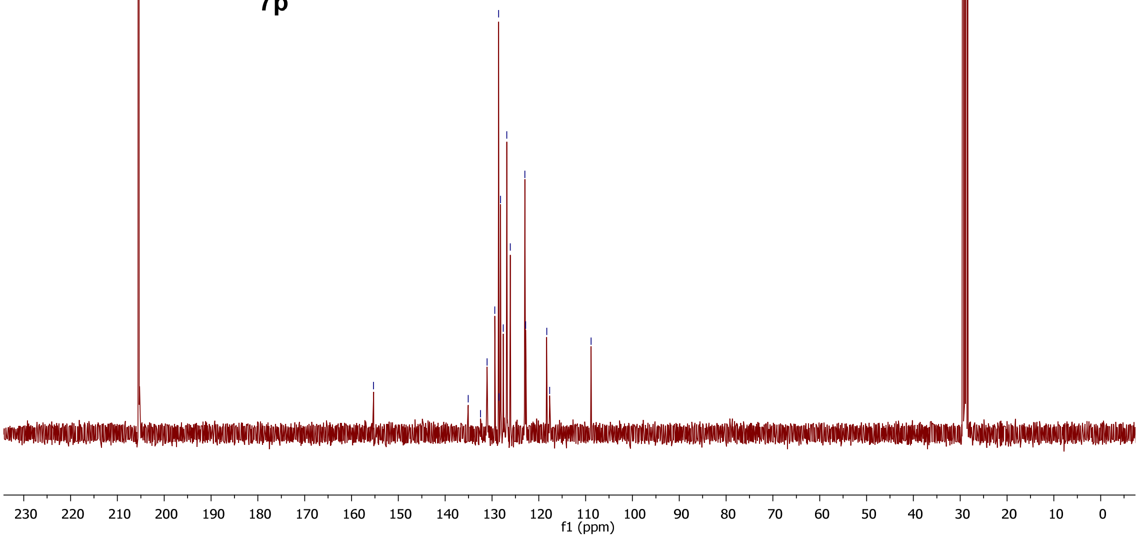

^{13}C NMR spectrum of 7p (100 MHz, $(CD_3)_2CO$)

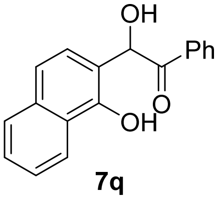

7q

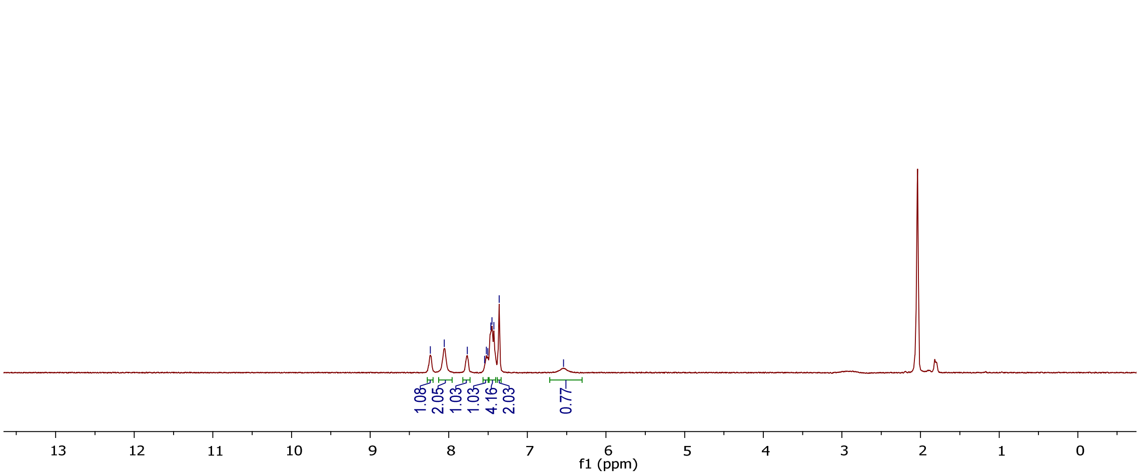

^{1}H NMR spectrum of 7q (400 MHz, $(CD_3)_2CO$)

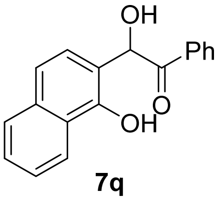

7q

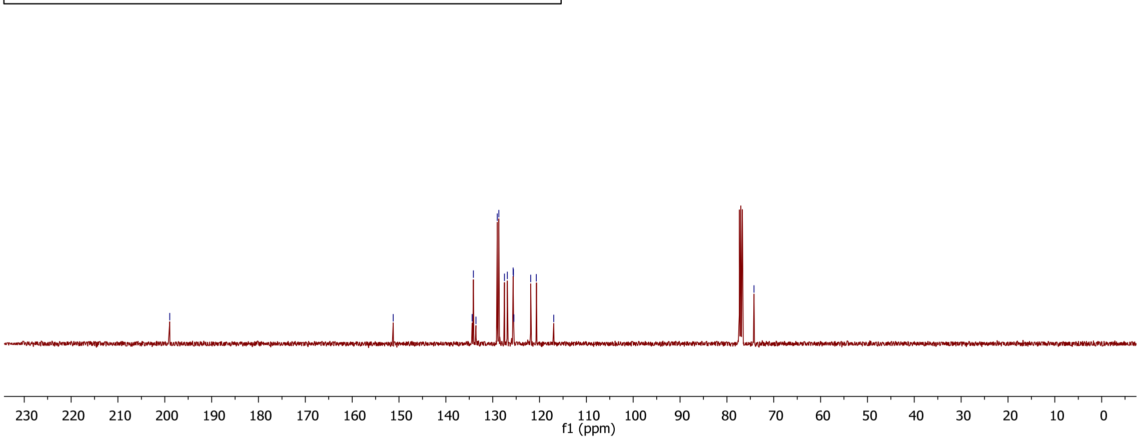

^{13}C NMR spectrum of 7q (100 MHz, $CDCl_3$)

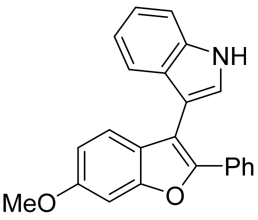

8a

^{1}H NMR spectrum of 8a (400 MHz, $CDCl_3$)

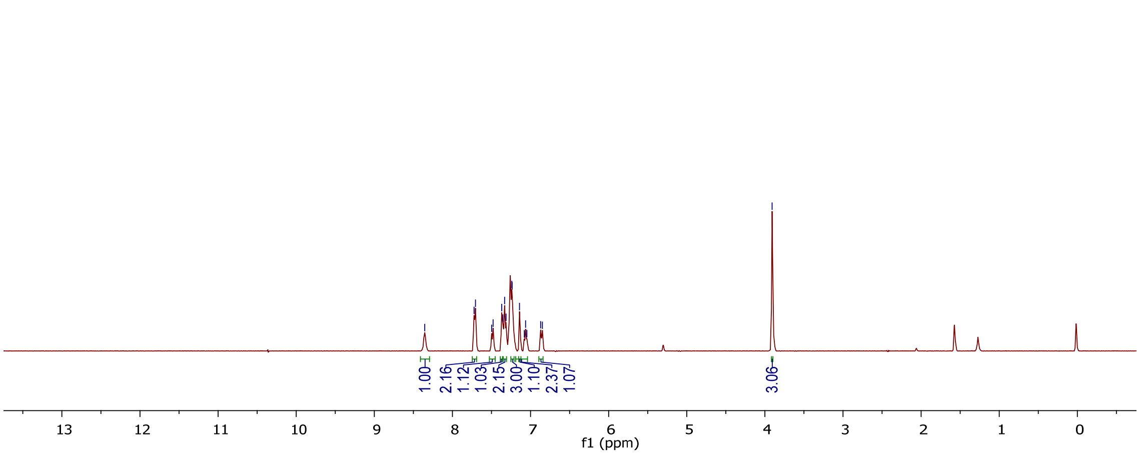

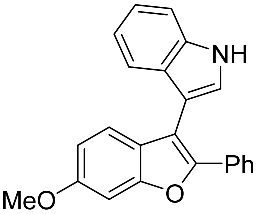

8a

^{13}C NMR spectrum of 8a (100 MHz, $CDCl_3$)

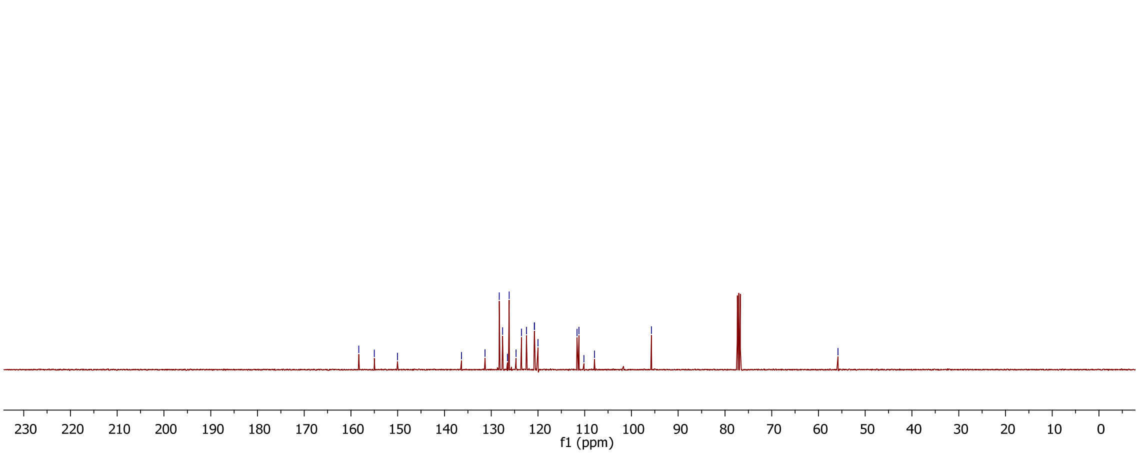

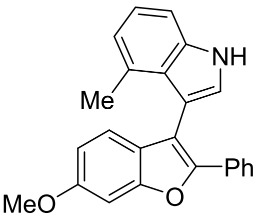

8b

^{1}H NMR spectrum of 8b (400 MHz, $CDCl_3$)

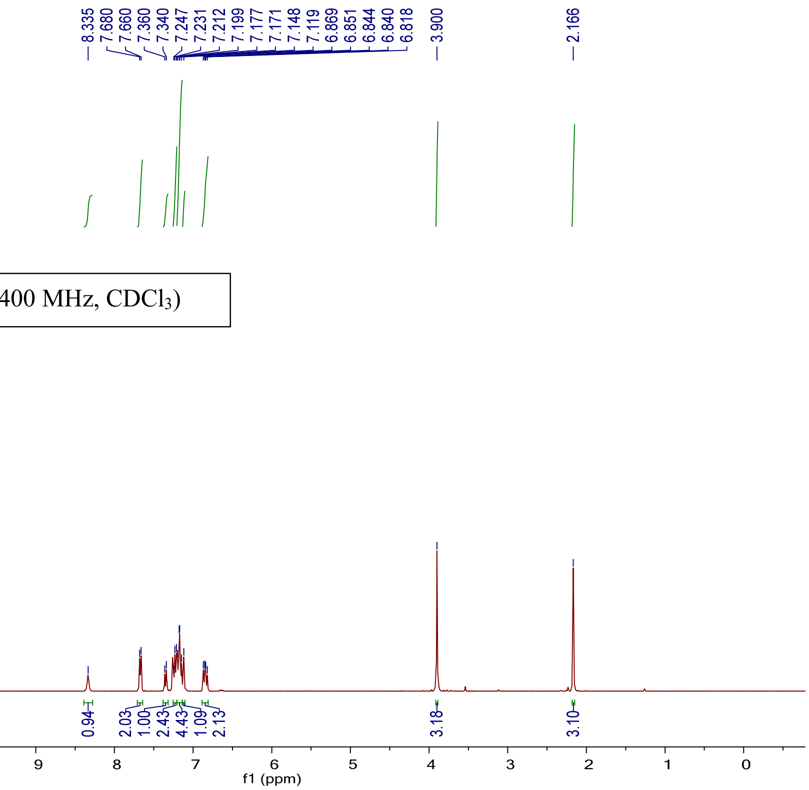

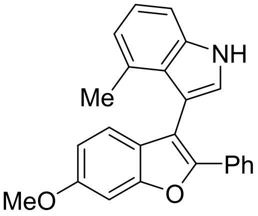

8b

^{13}C NMR spectrum of 8b (100 MHz, $CDCl_3$)

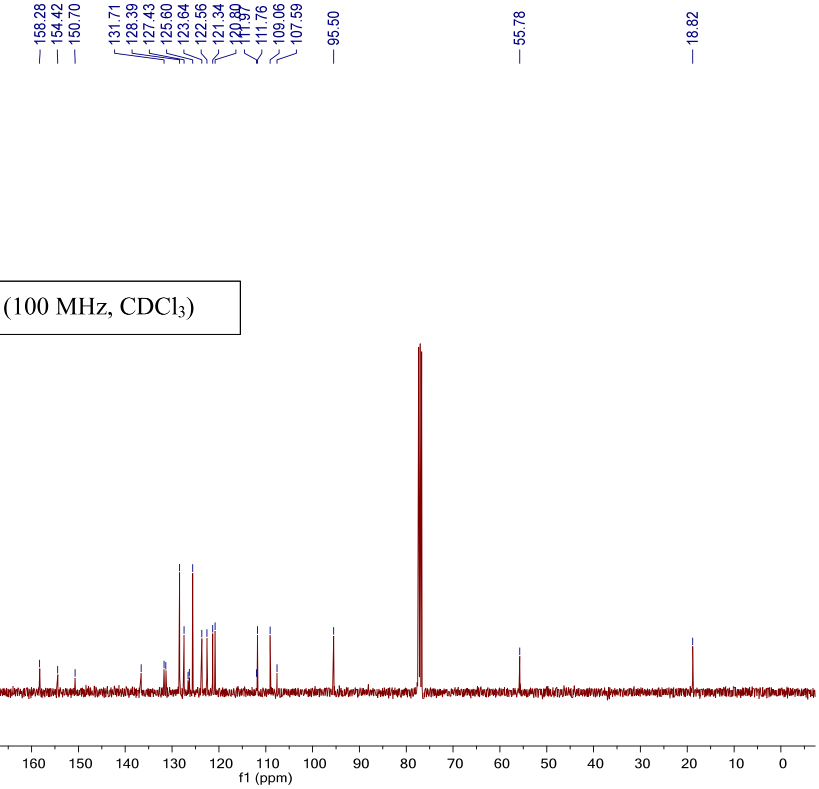

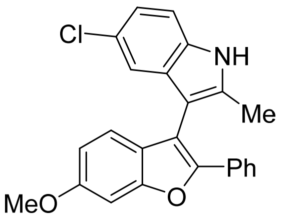

8c

^{1}H NMR spectrum of 8c (400 MHz, $CDCl_3$)

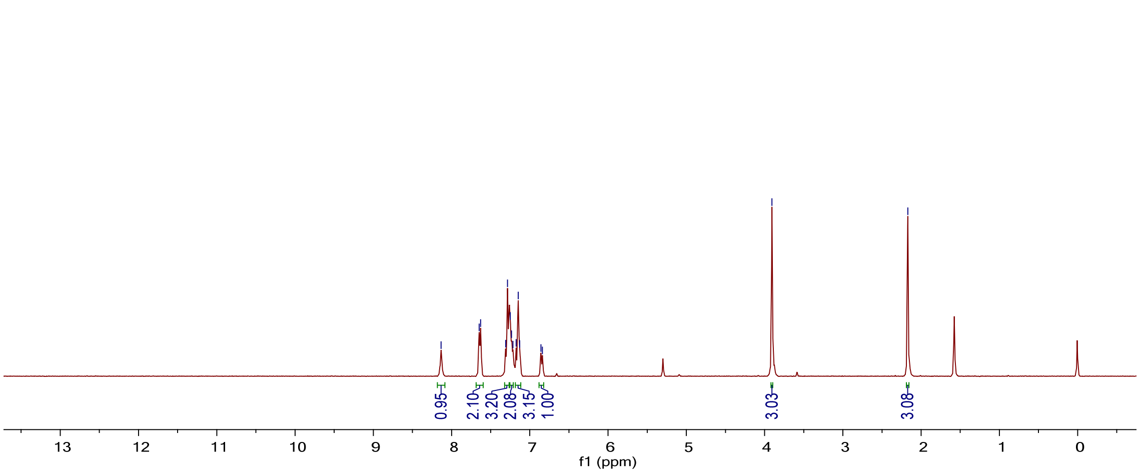

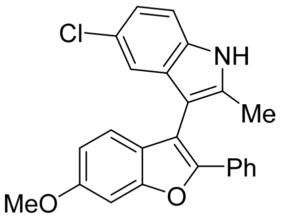

8c

^{13}C NMR spectrum of 8c (100 MHz, $CDCl_3$)

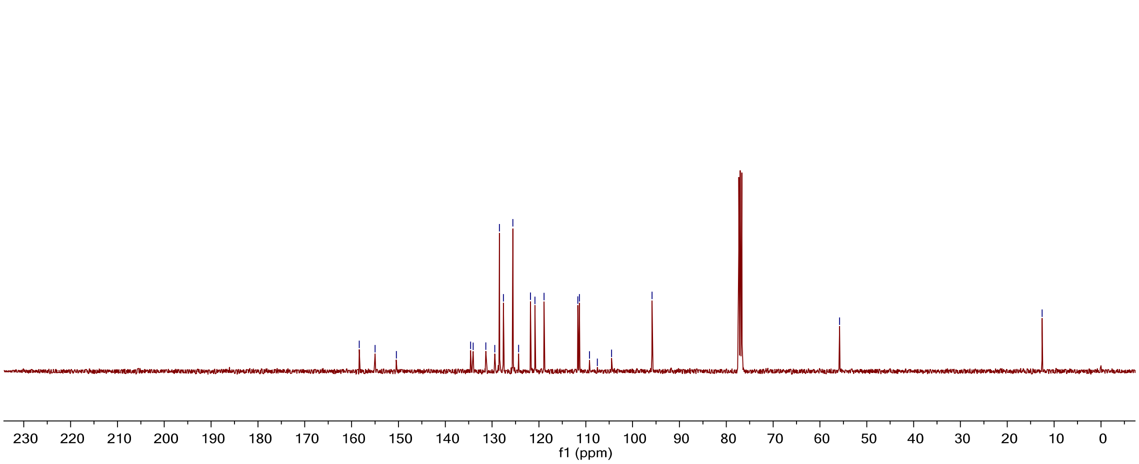

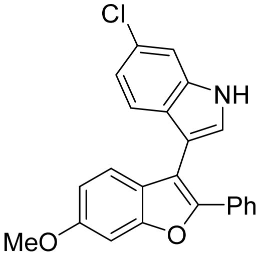

8d

^{1}H NMR spectrum of 8d (400 MHz, $CDCl_3$)

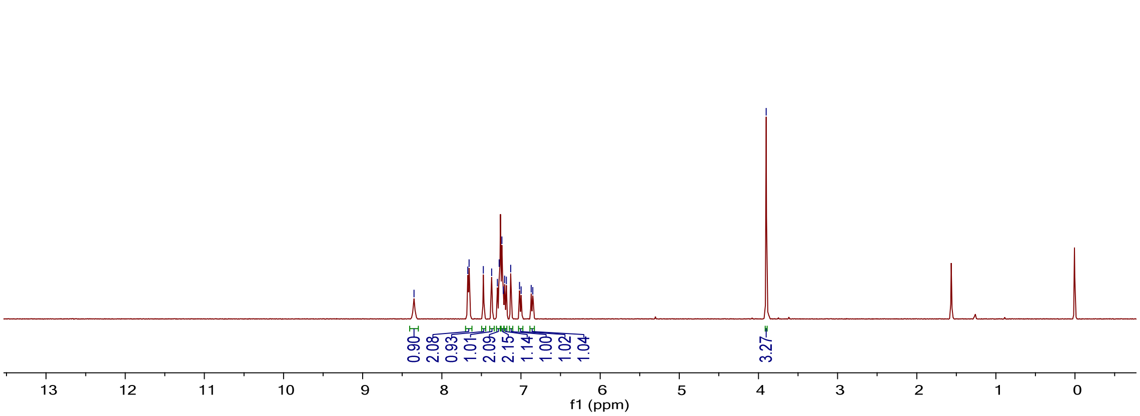

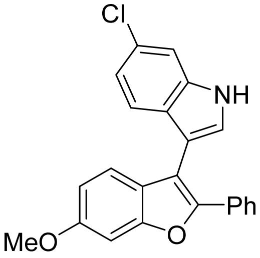

8d

^{13}C NMR spectrum of 8d (100 MHz, $CDCl_3$)

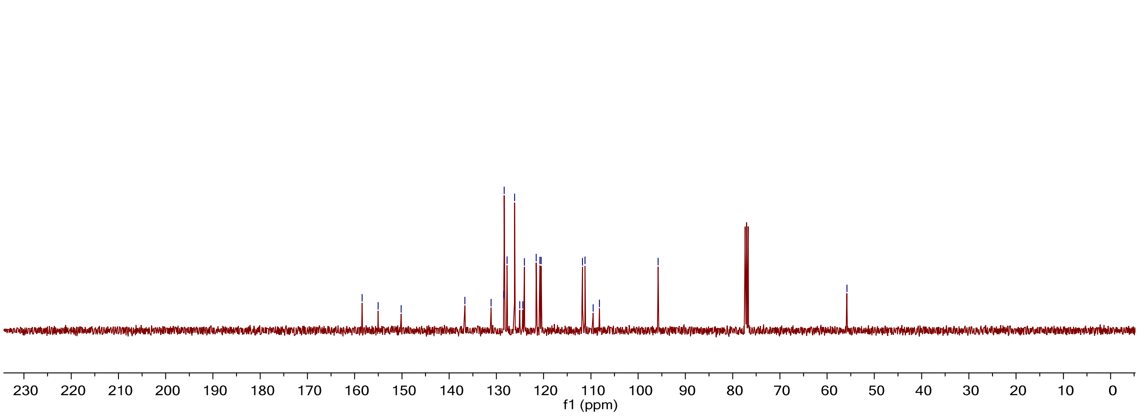

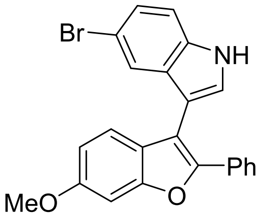

8e

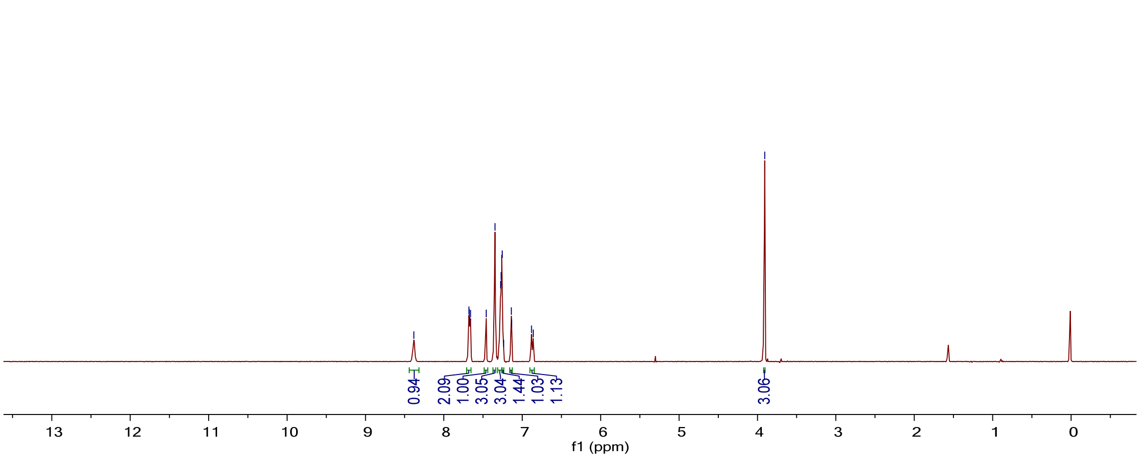

^{1}H NMR spectrum of 8e (400 MHz, $CDCl_3$)

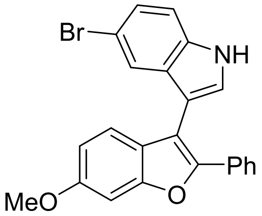

8e

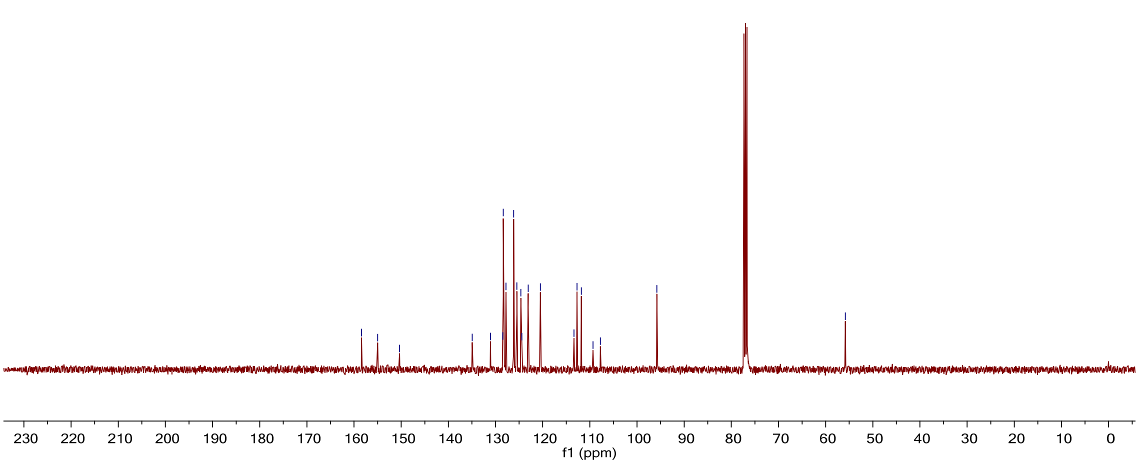

^{13}C NMR spectrum of 8e (100 MHz, $CDCl_3$)

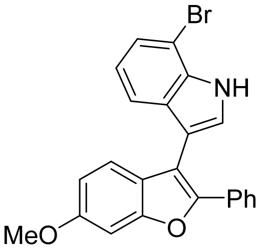

8f

^{1}H NMR spectrum of 8f (400 MHz, $CDCl_3$)

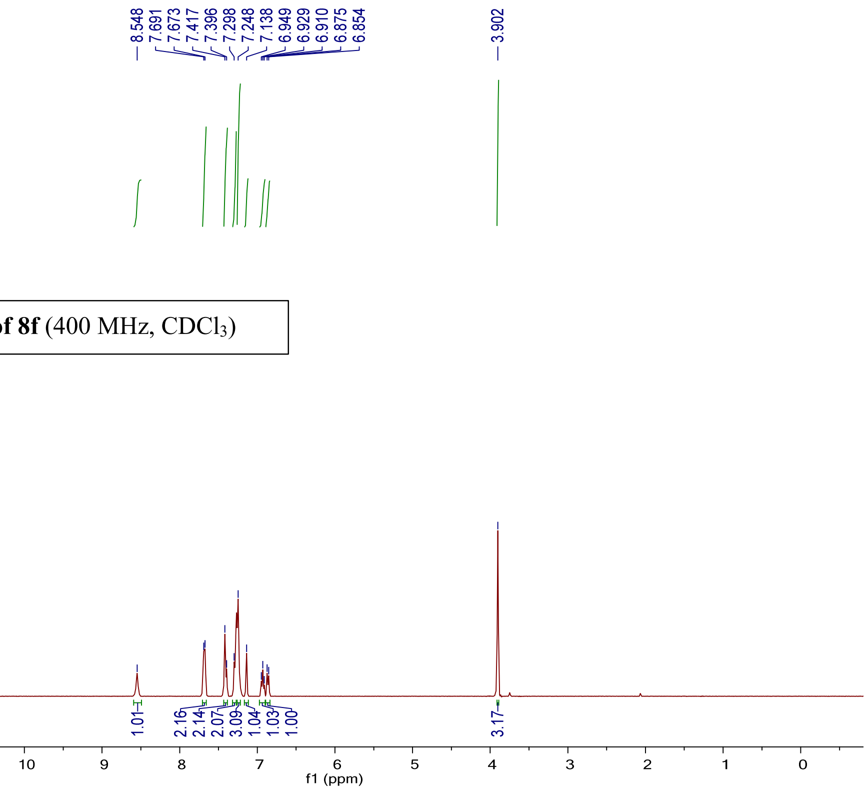

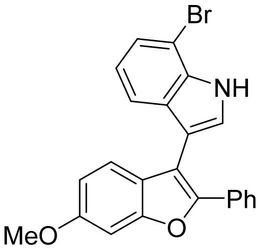

8f

^{13}C NMR spectrum of 8f (100 MHz, $CDCl_3$)

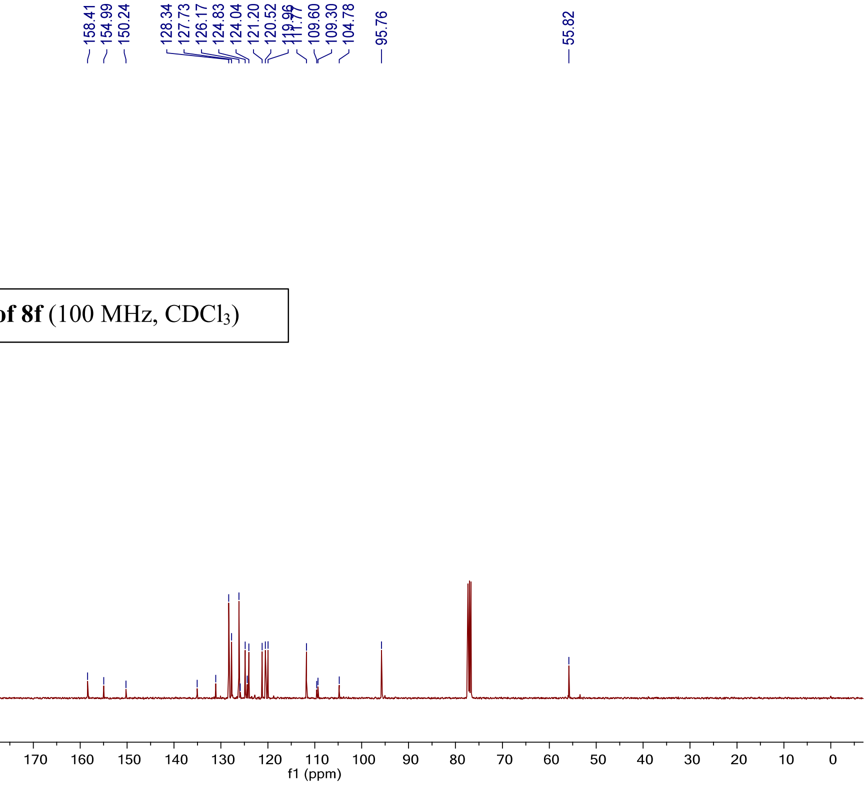

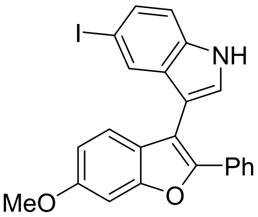

8g

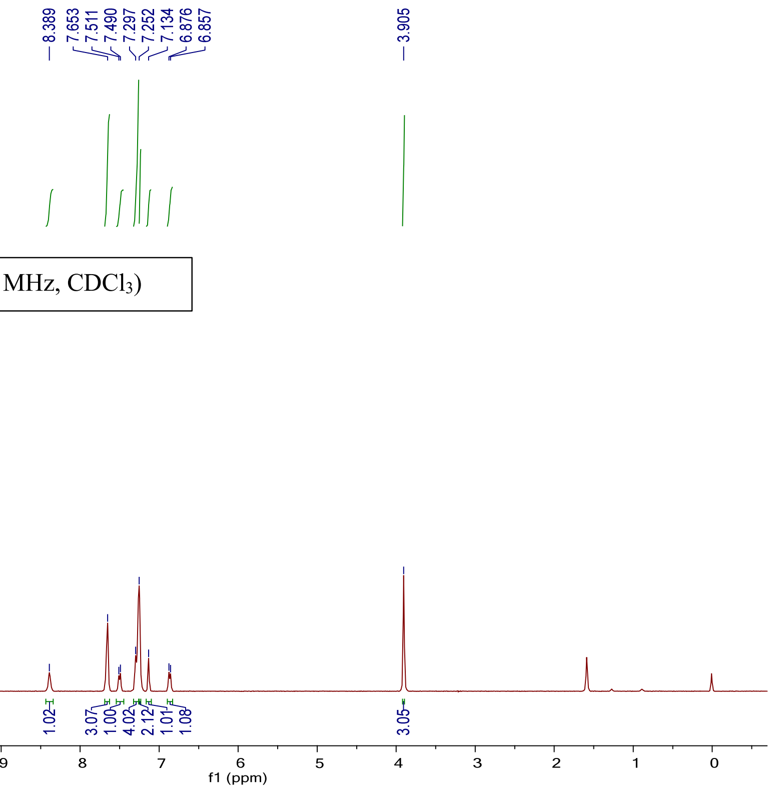

^{1}H NMR spectrum of 8g (400 MHz, $CDCl_3$)

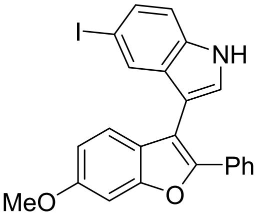

8g

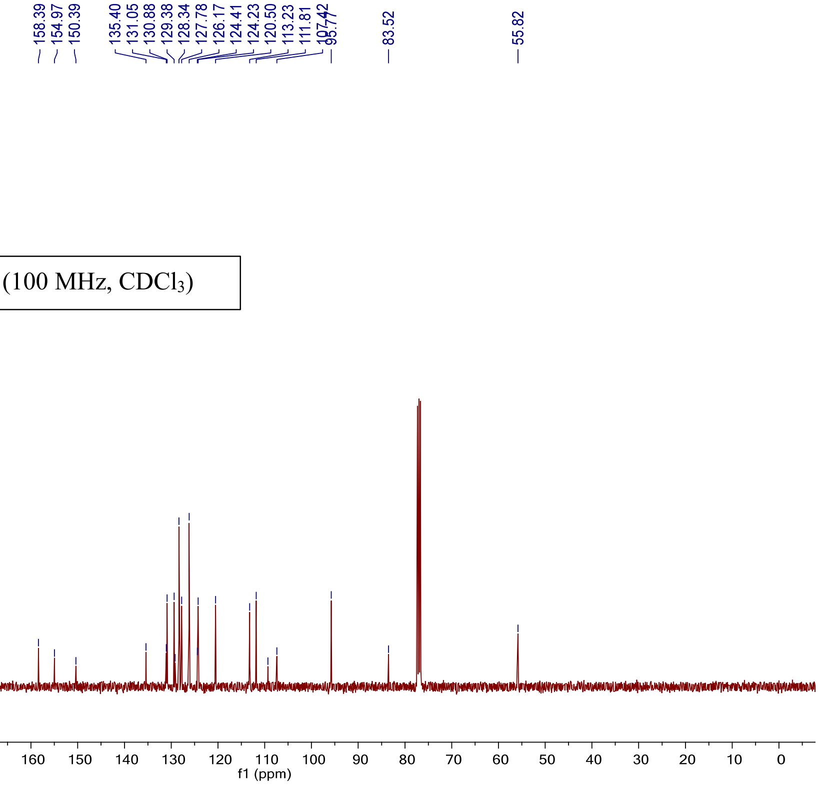

^{13}C NMR spectrum of 8g (100 MHz, $CDCl_3$)

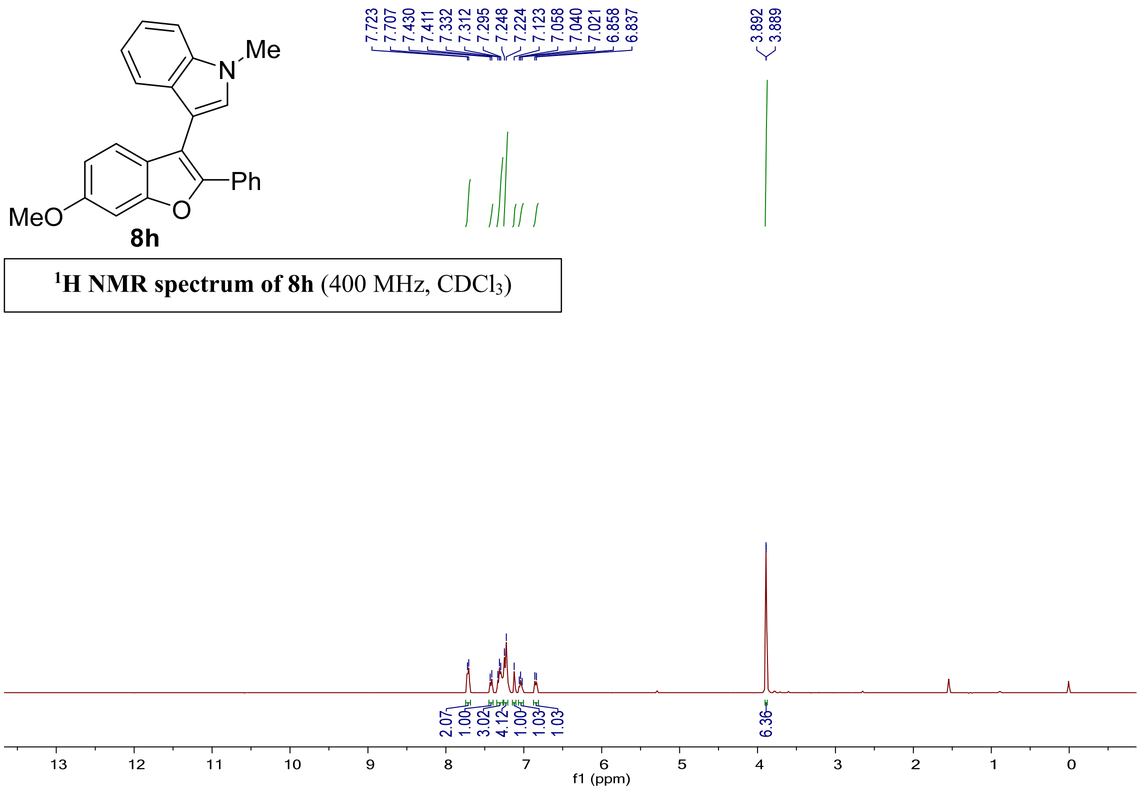

^{1}H NMR spectrum of 8h (400 MHz, $CDCl_3$)

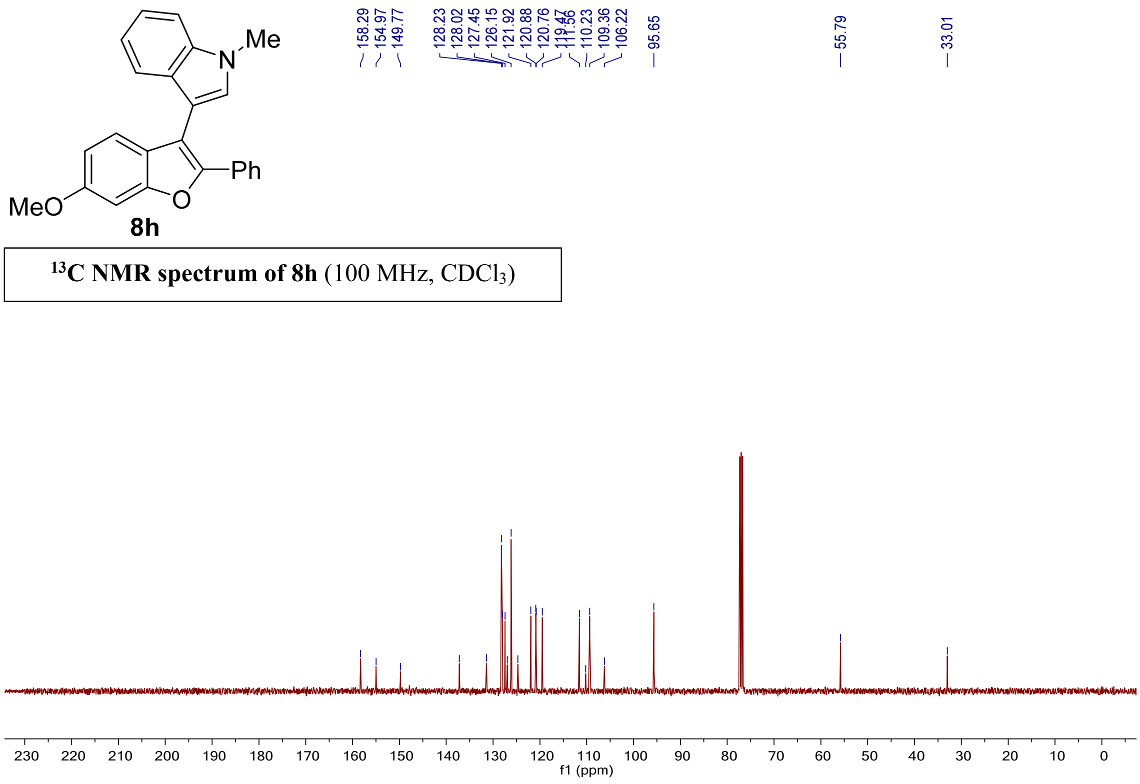

^{13}C NMR spectrum of 8h (100 MHz, $CDCl_3$)

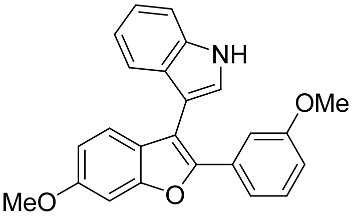

8i

^{1}H NMR spectrum of 8i (400 MHz, $CDCl_3$)

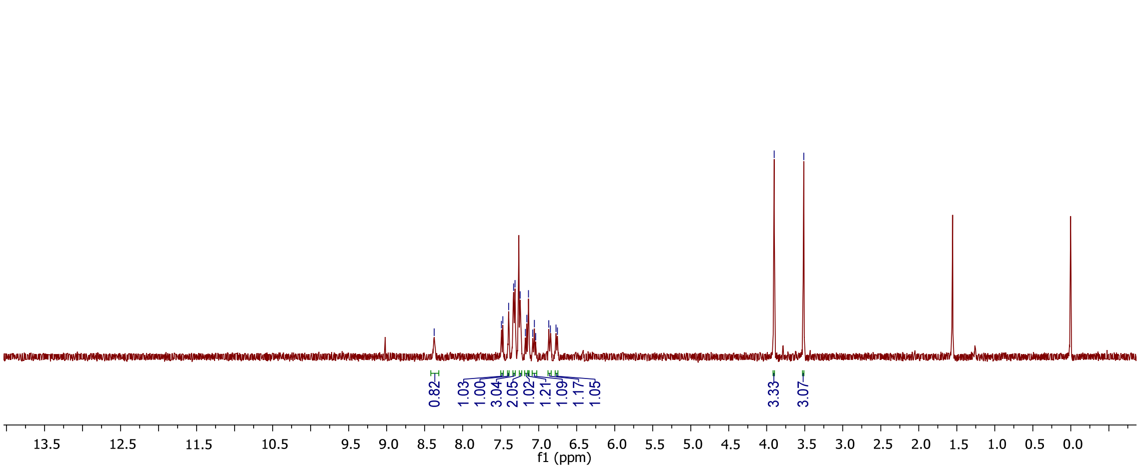

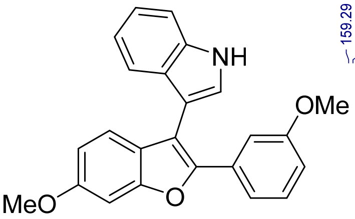

8i

^{13}C NMR spectrum of 8i (100 MHz, $CDCl_3$)

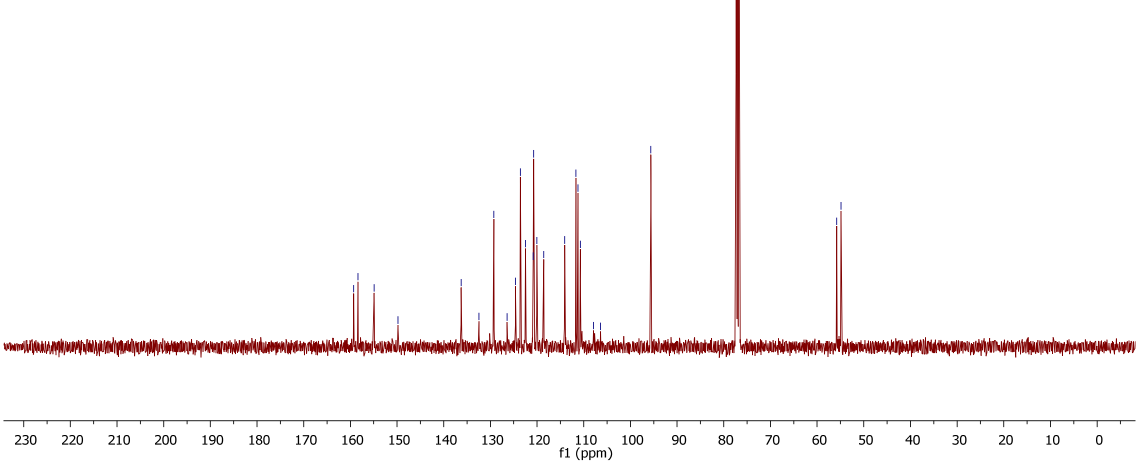

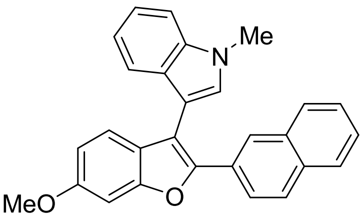

8j

^{1}H NMR spectrum of 8j (400 MHz, $CDCl_3$)

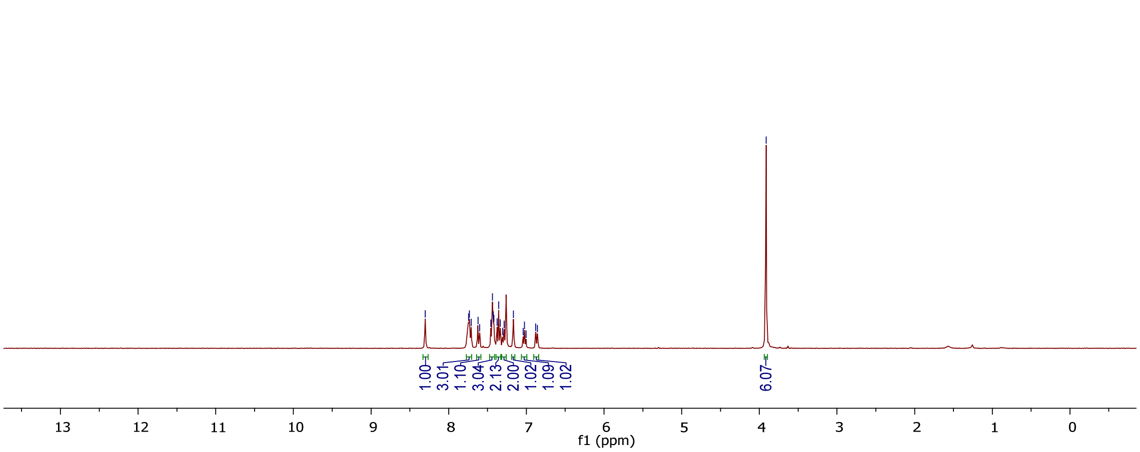

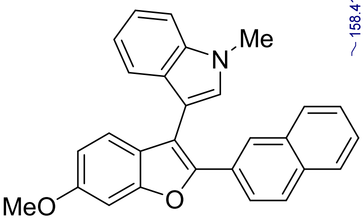

8j

^{13}C NMR spectrum of 8j (100 MHz, $CDCl_3$)

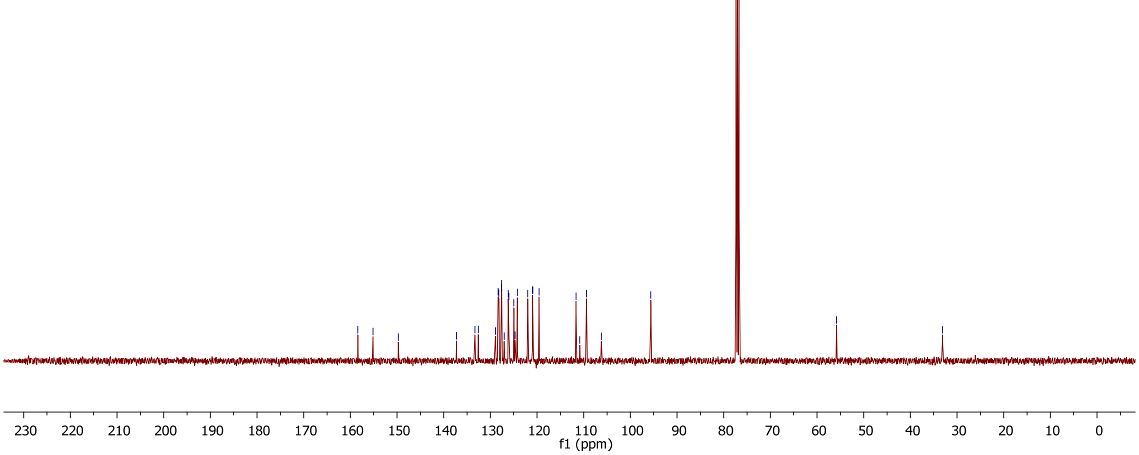

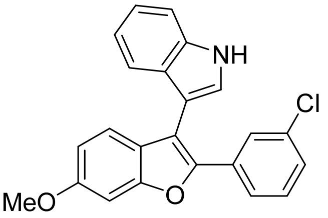

8k

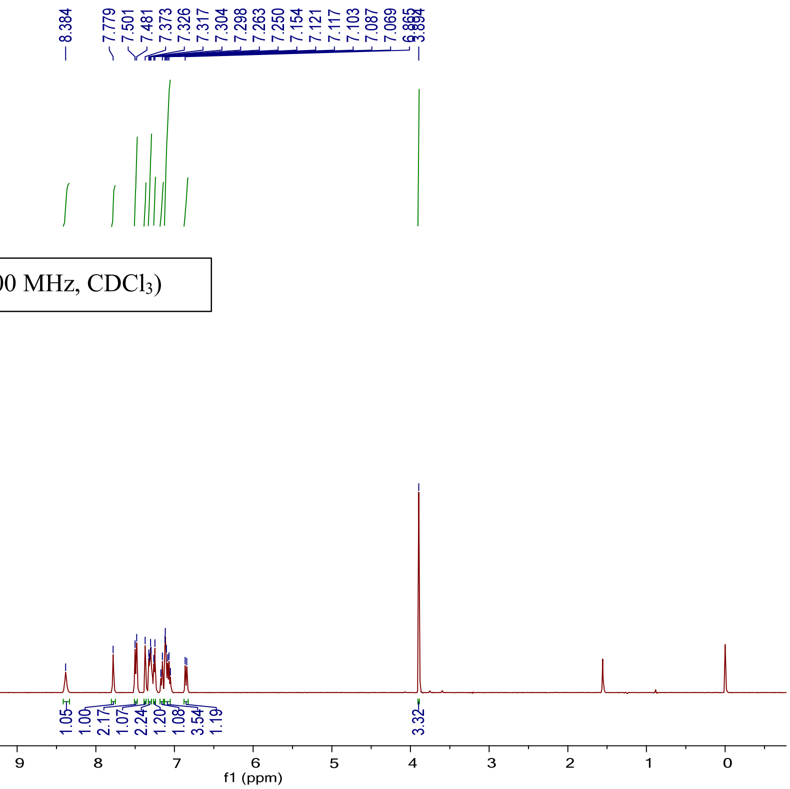

[1]**H NMR spectrum of 8k** (400 MHz, $CDCl_3$)

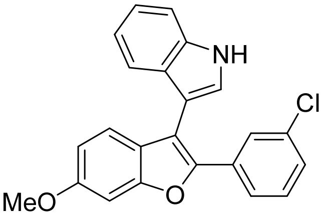

8k

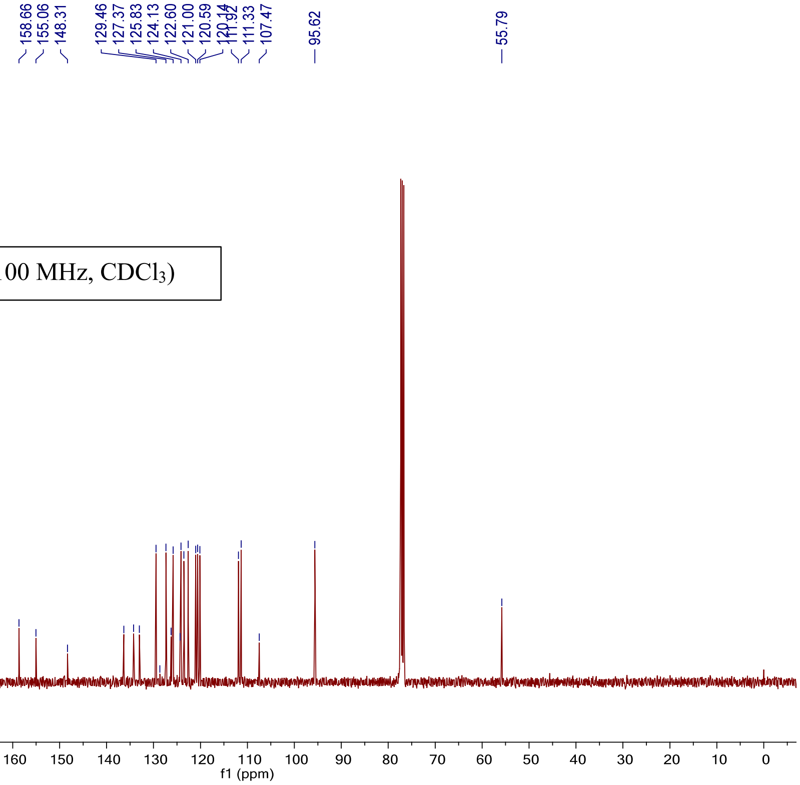

[13]**C NMR spectrum of 8k** (100 MHz, $CDCl_3$)

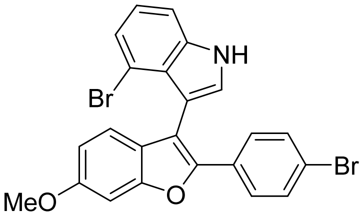

8l

^{1}H NMR spectrum of 8l (400 MHz, $CDCl_3$)

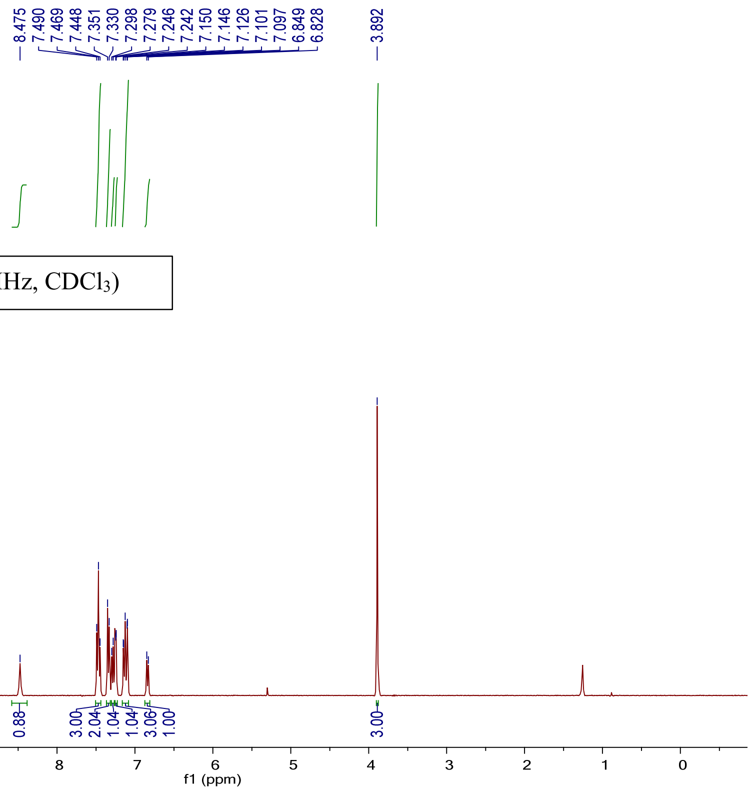

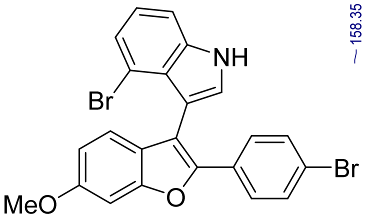

8l

^{13}C NMR spectrum of 8l (100 MHz, $CDCl_3$)

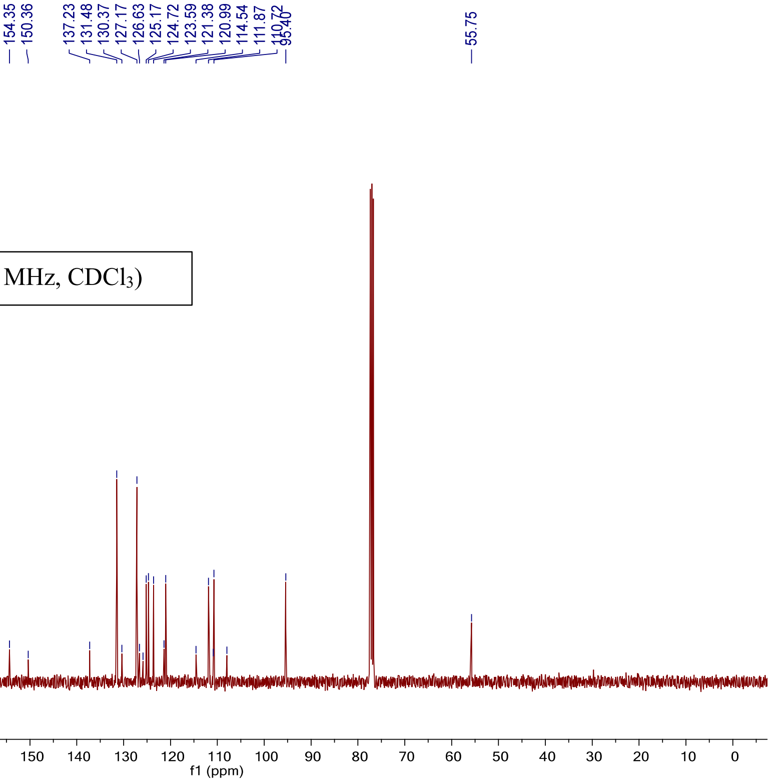

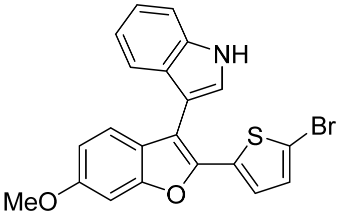

8m

^{1}H NMR spectrum of 8m (400 MHz, $CDCl_3$)

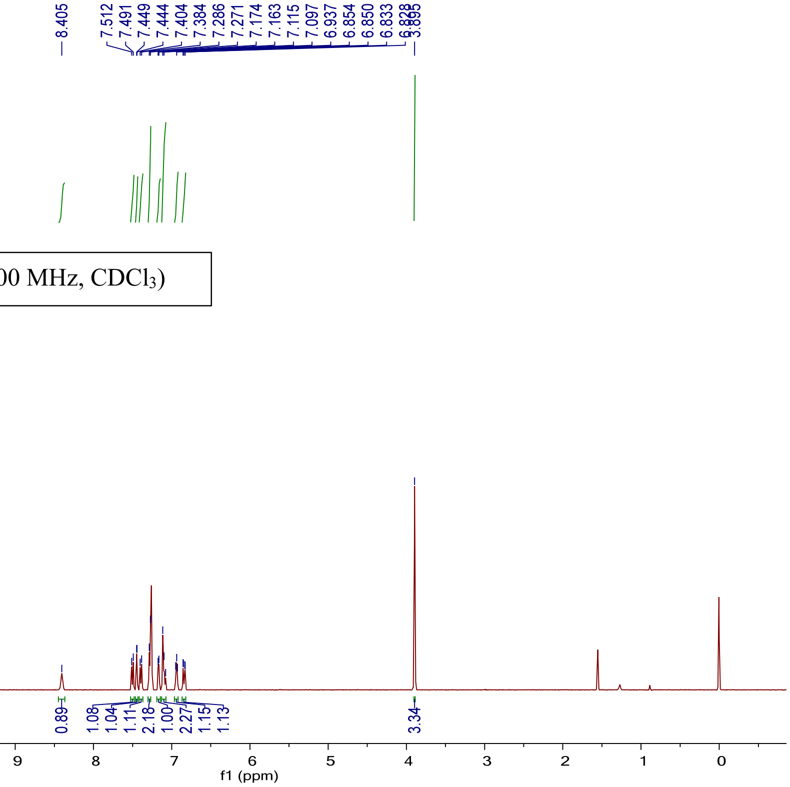

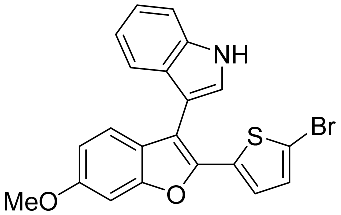

8m

^{13}C NMR spectrum of 8m (100 MHz, $CDCl_3$)

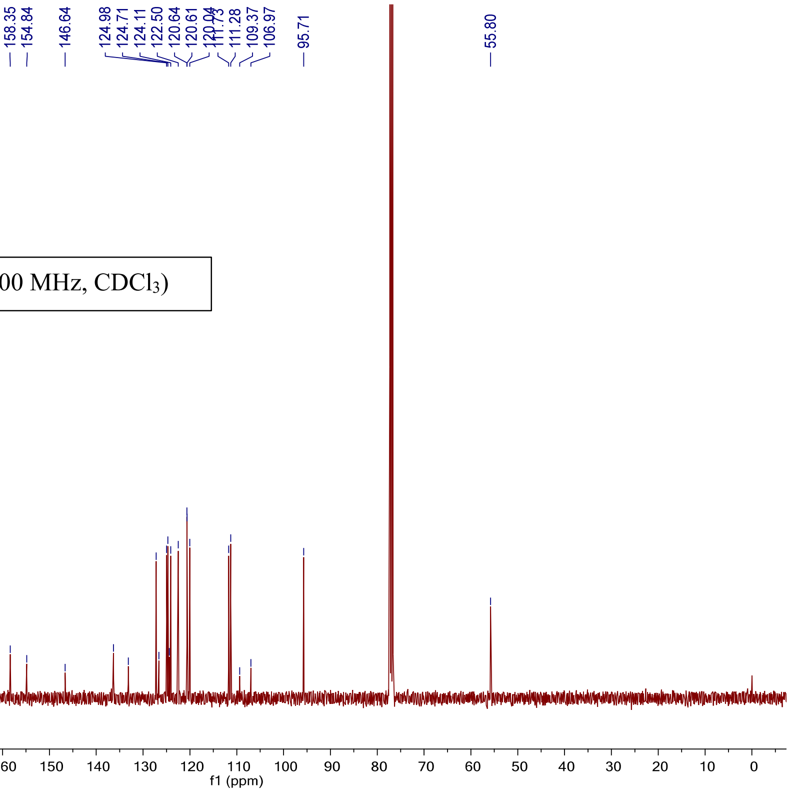

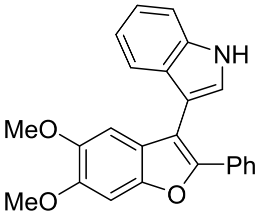

8n

¹H NMR spectrum of 8n (400 MHz, $CDCl_3$)

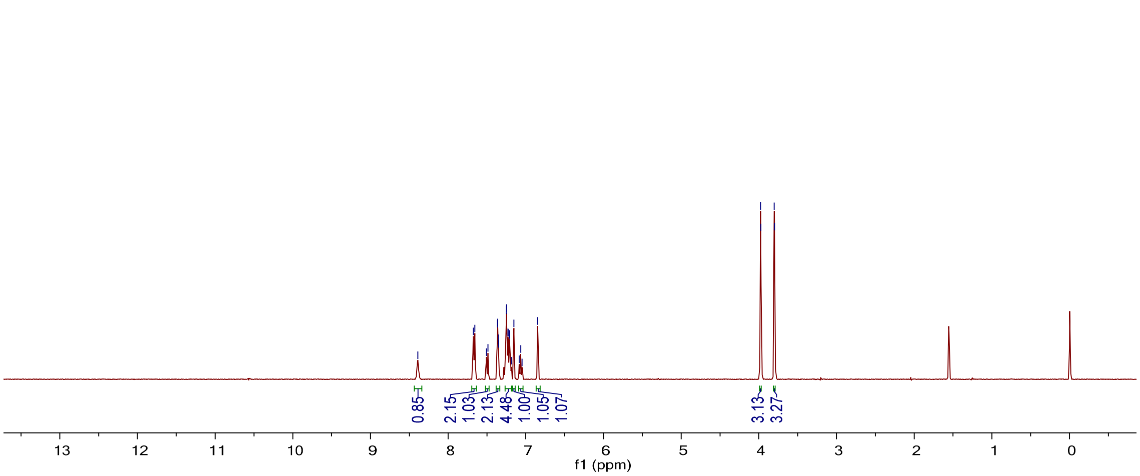

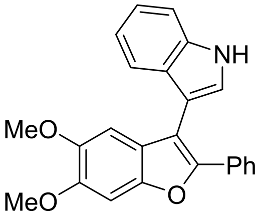

8n

¹³C NMR spectrum of 8n (100 MHz, $CDCl_3$)

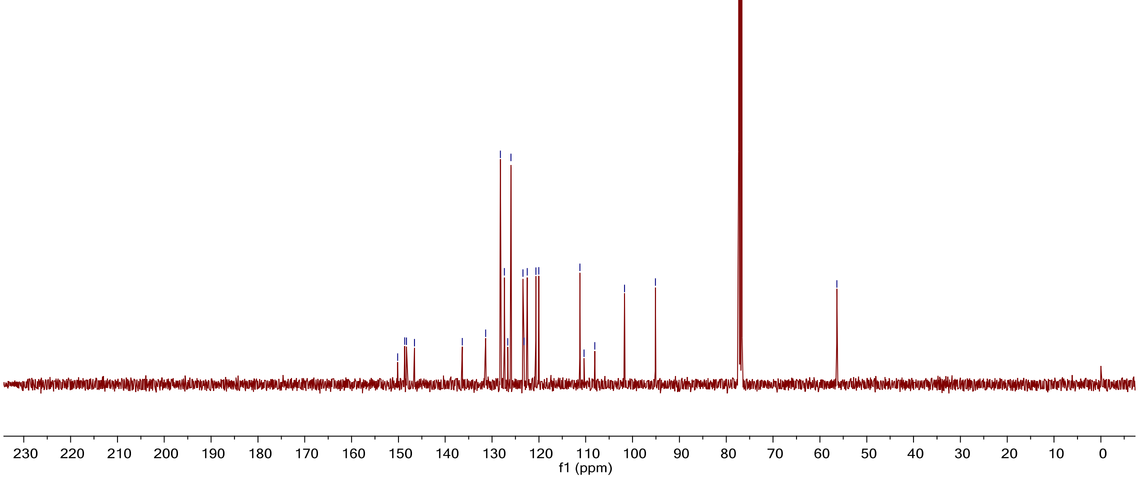

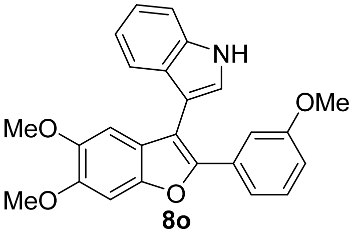

8o

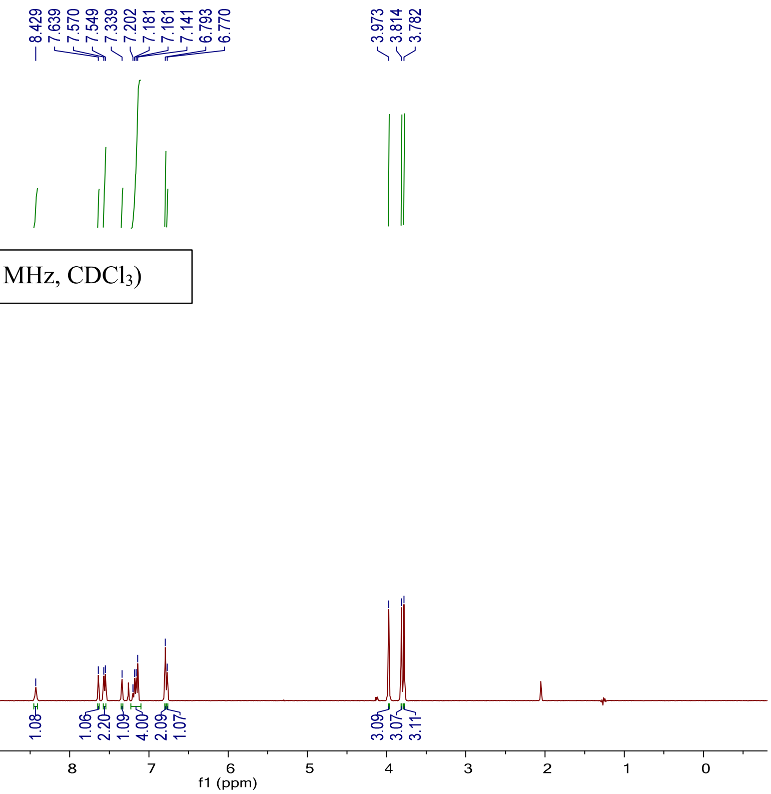

^{1}H NMR spectrum of 8o (400 MHz, $CDCl_3$)

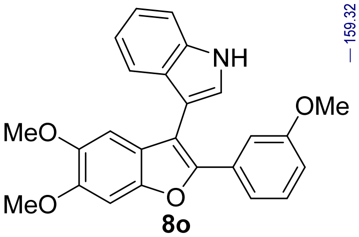

8o

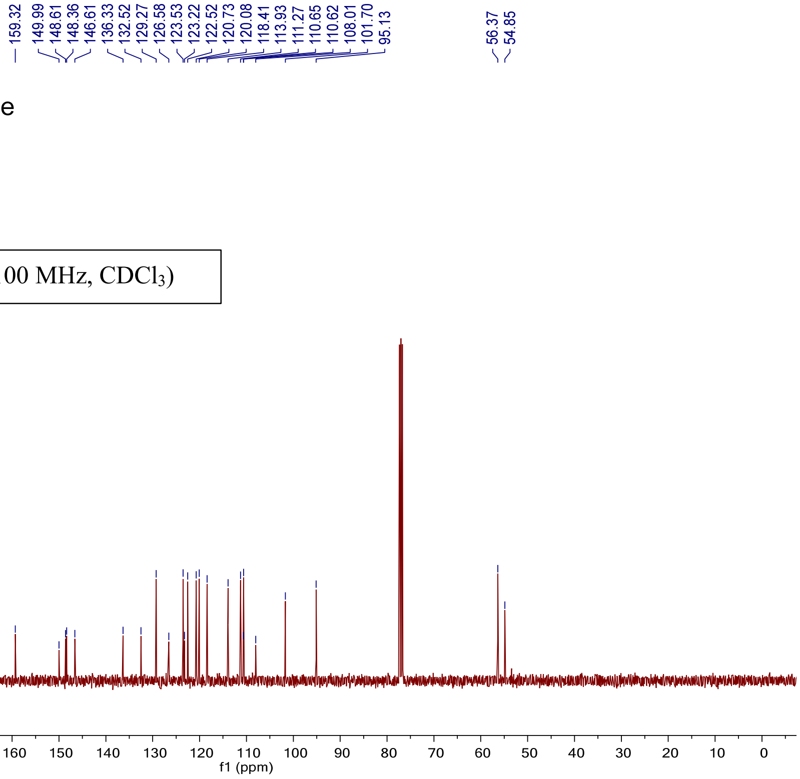

^{13}C NMR spectrum of 8o (100 MHz, $CDCl_3$)

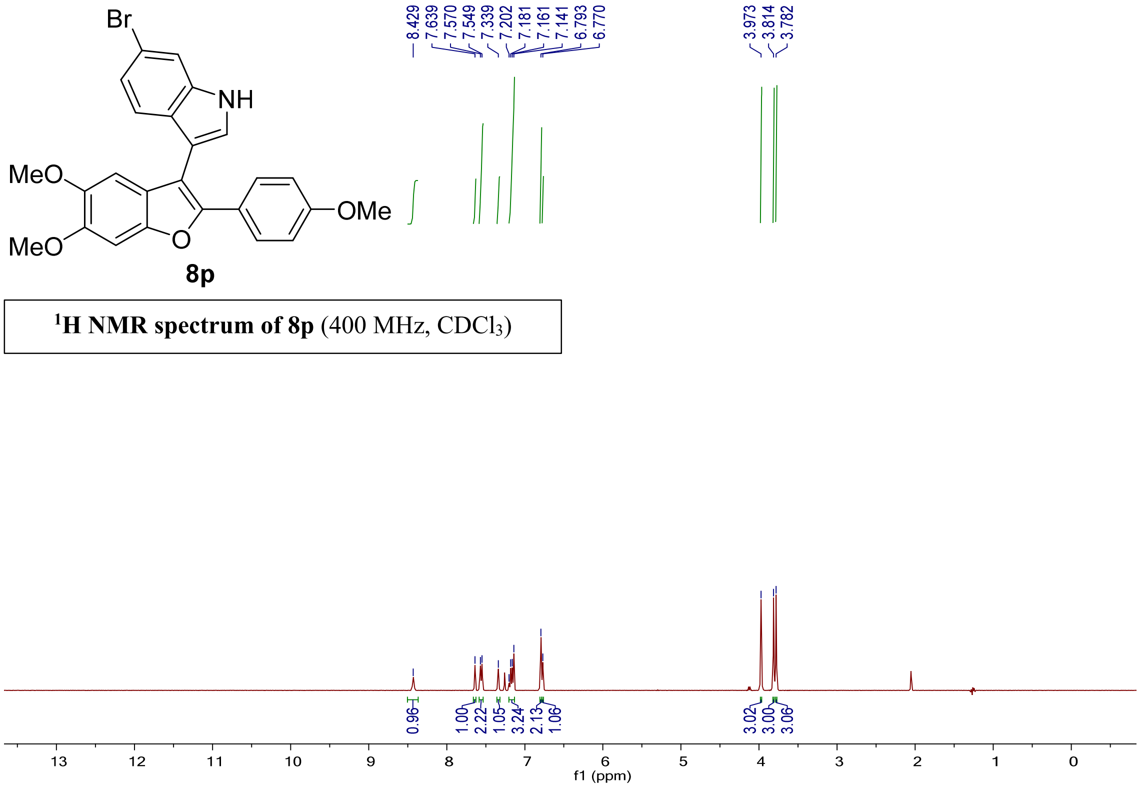

^{1}H NMR spectrum of 8p (400 MHz, $CDCl_3$)

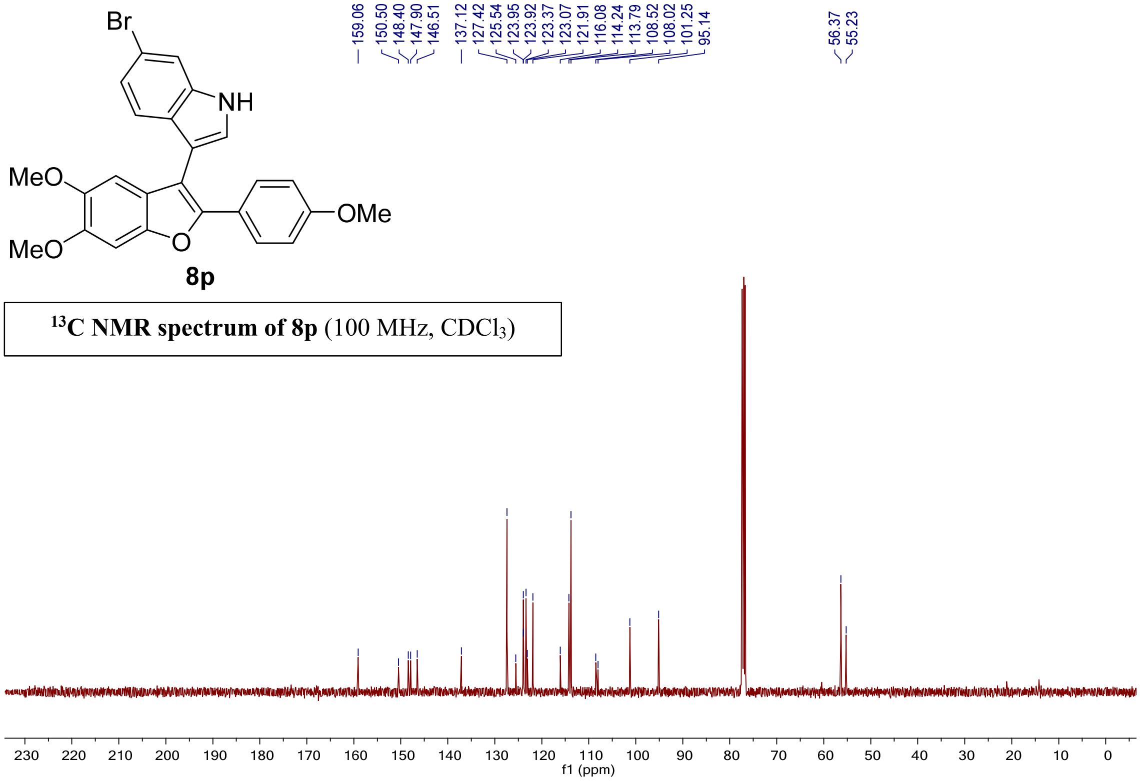

^{13}C NMR spectrum of 8p (100 MHz, $CDCl_3$)

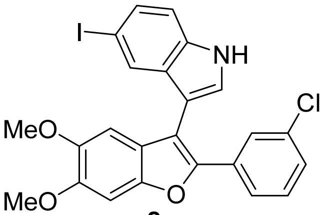

8q

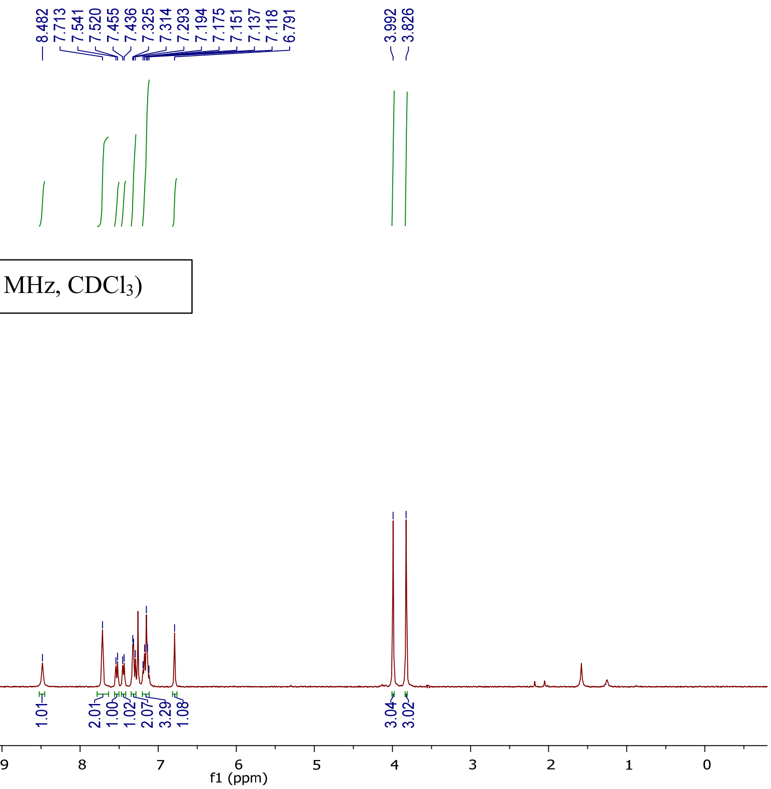

^{1}H NMR spectrum of 8q (400 MHz, $CDCl_3$)

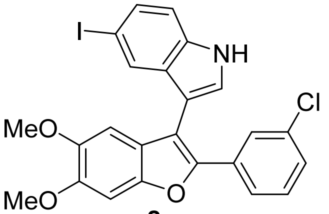

8q

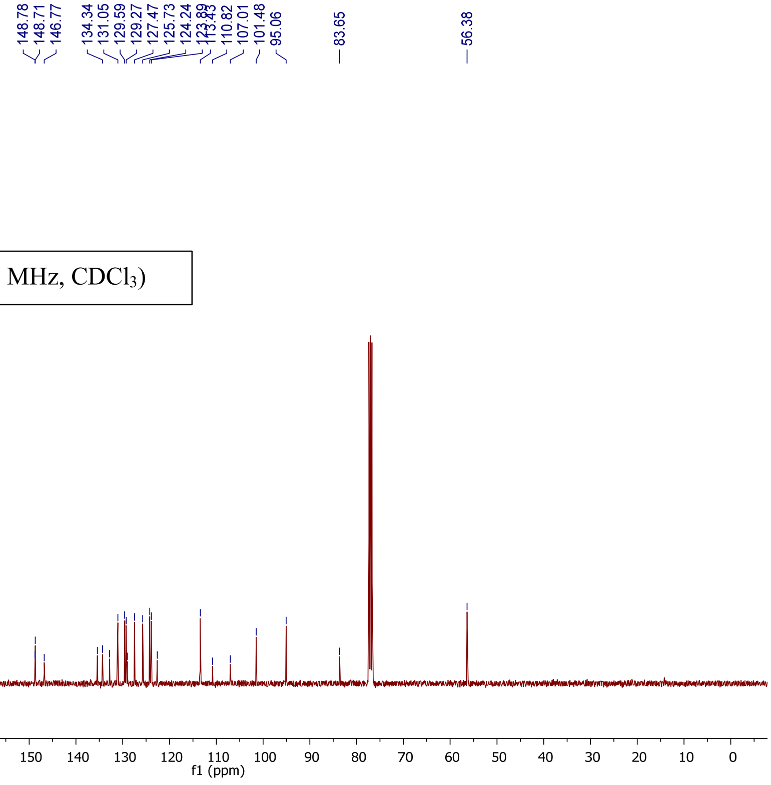

^{13}C NMR spectrum of 8q (100 MHz, $CDCl_3$)

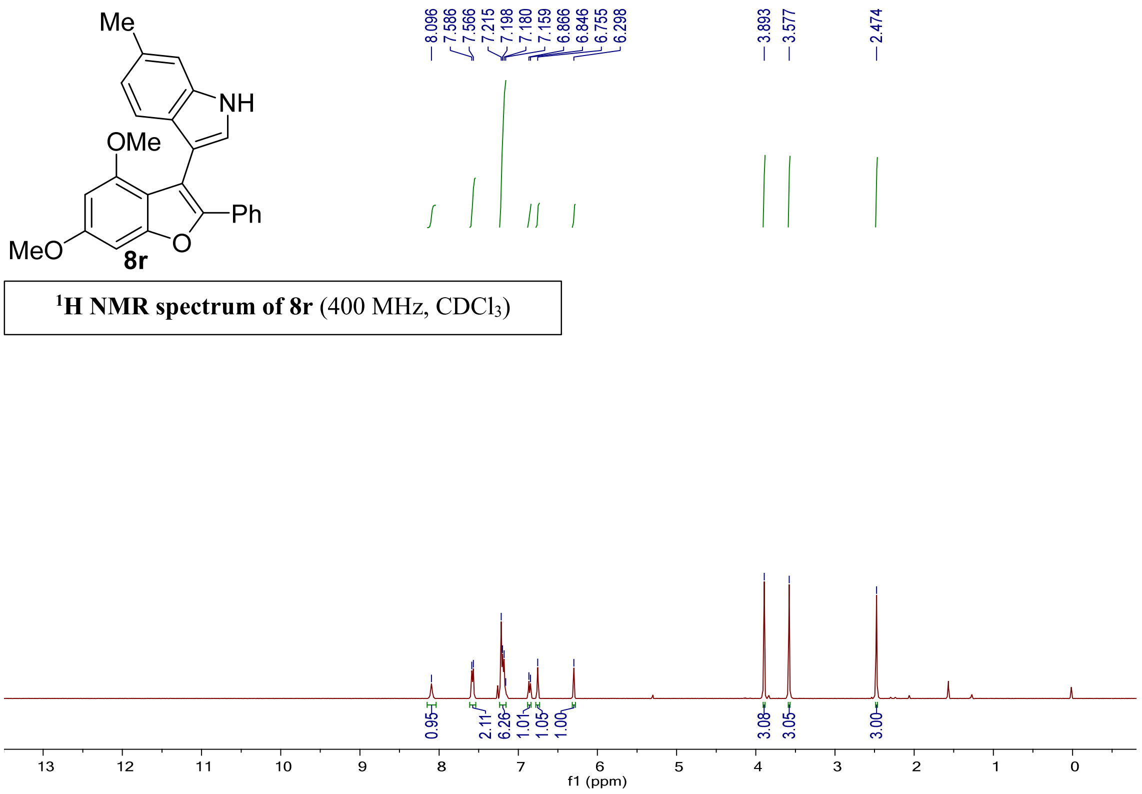

^{1}H NMR spectrum of 8r (400 MHz, $CDCl_3$)

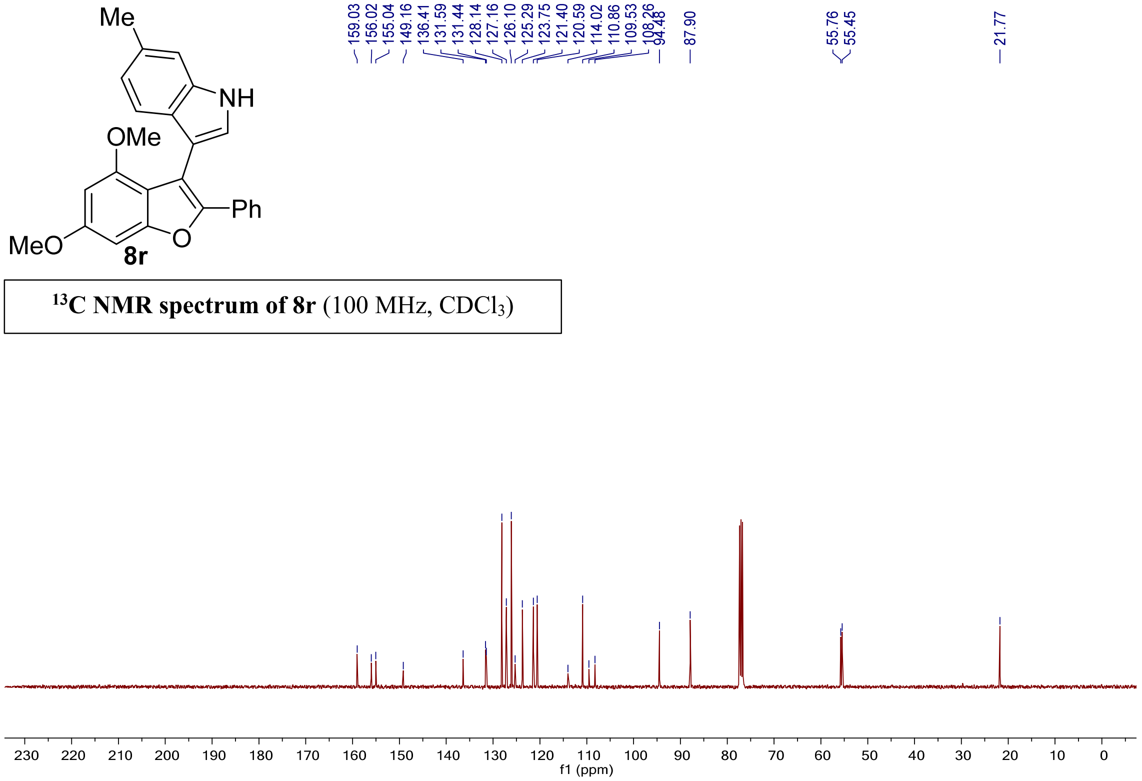

^{13}C NMR spectrum of 8r (100 MHz, $CDCl_3$)

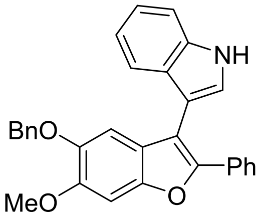

8s

¹H NMR spectrum of 8s (400 MHz, $CDCl_3$)

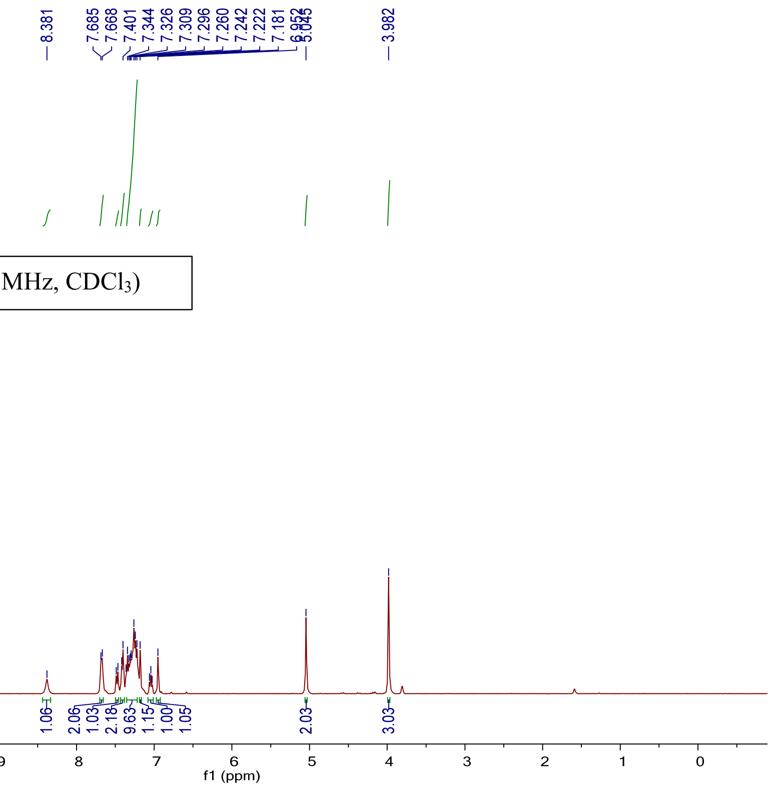

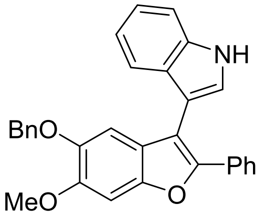

8s

¹³C NMR spectrum of 8s (100 MHz, $CDCl_3$)

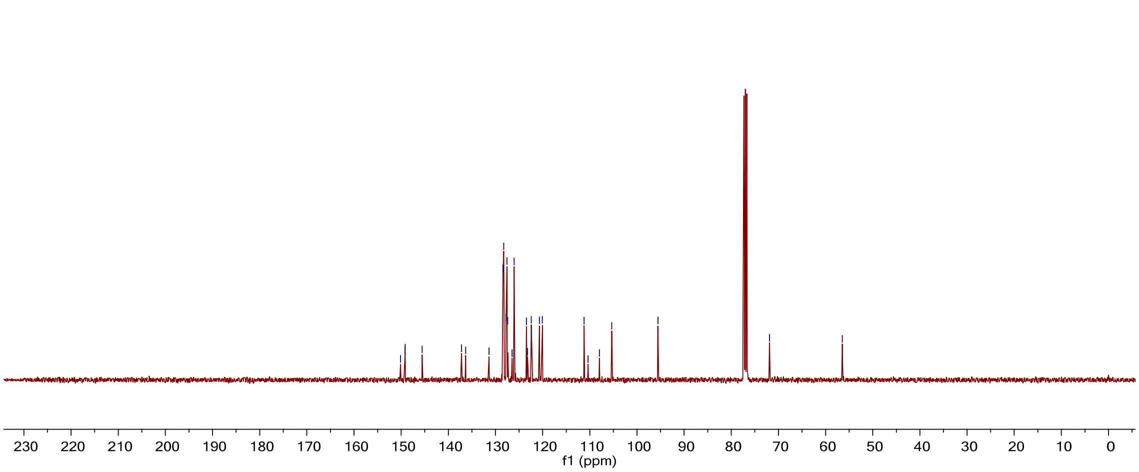

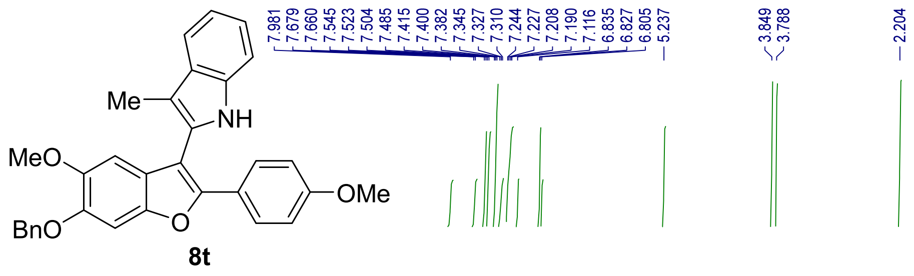

^{1}H NMR spectrum of 8t (400 MHz, $CDCl_3$)

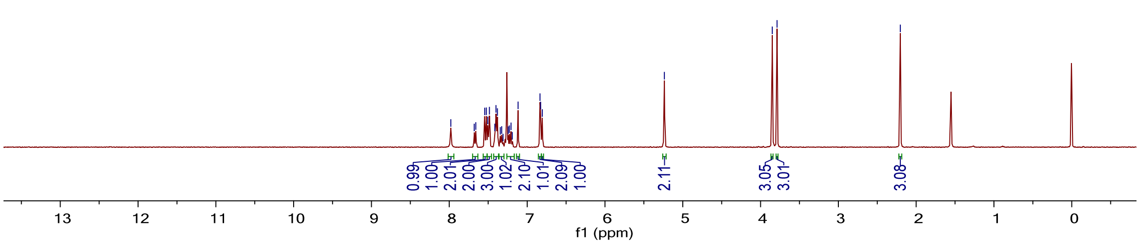

^{13}C NMR spectrum of 8t (100 MHz, $CDCl_3$)

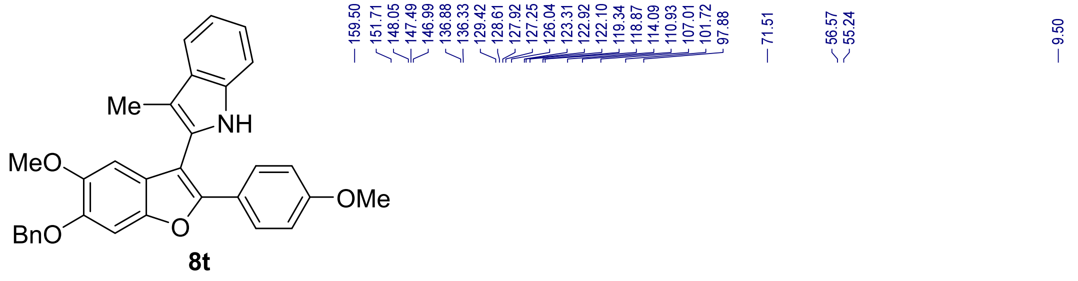

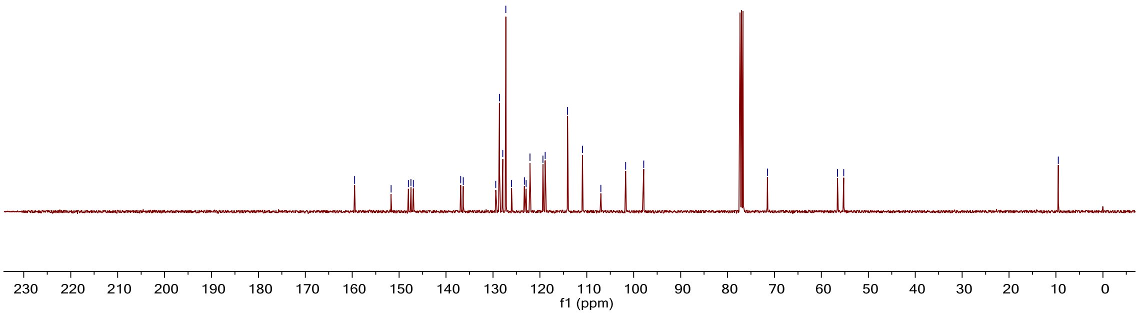

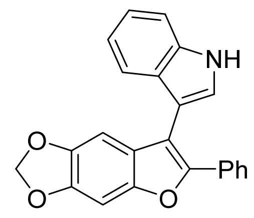

8u

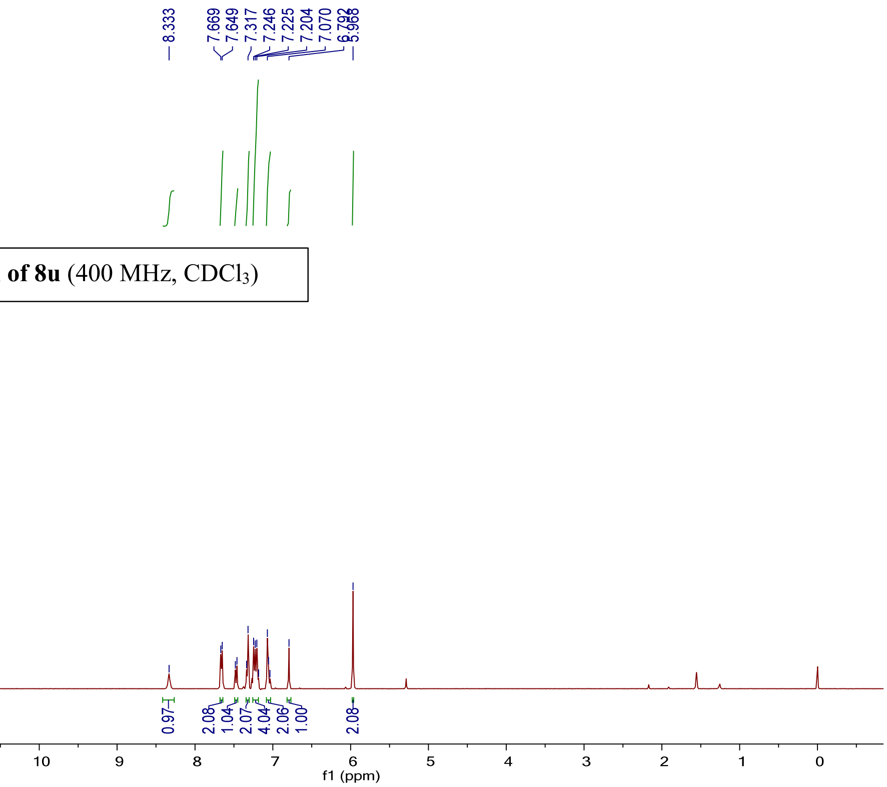

^{1}H NMR spectrum of 8u (400 MHz, $CDCl_3$)

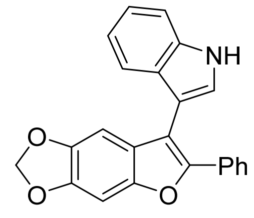

8u

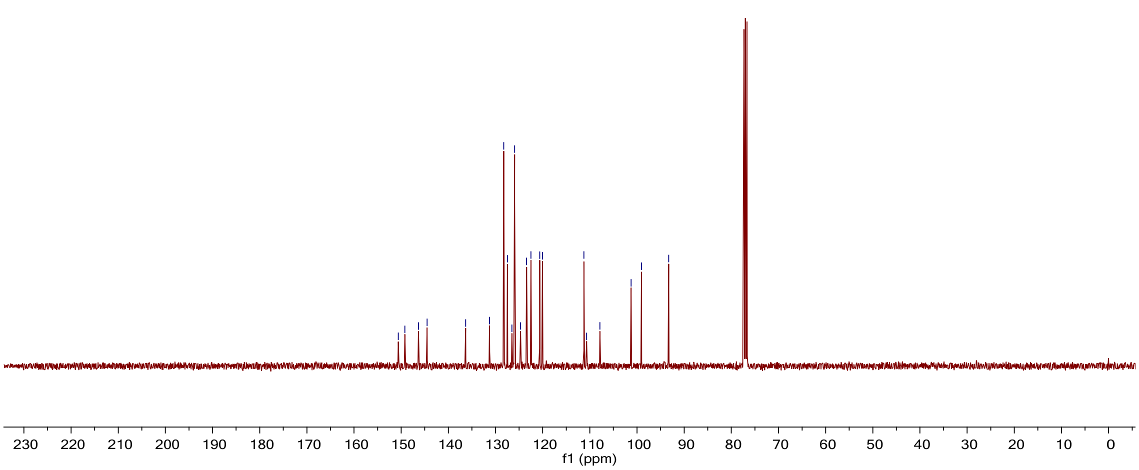

^{13}C NMR spectrum of 8u (100 MHz, $CDCl_3$)

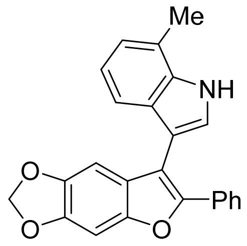

8v

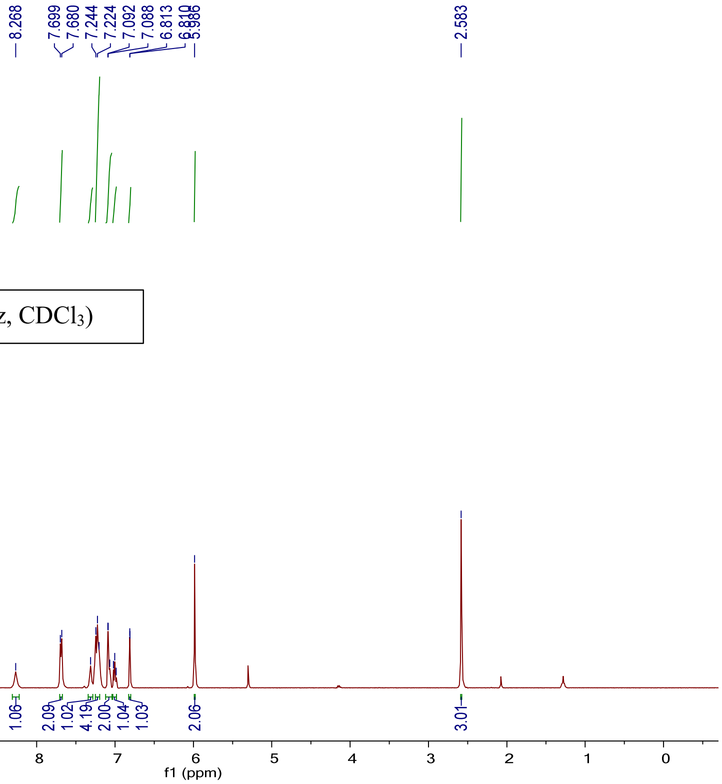

^{1}H NMR spectrum of 8v (400 MHz, $CDCl_3$)

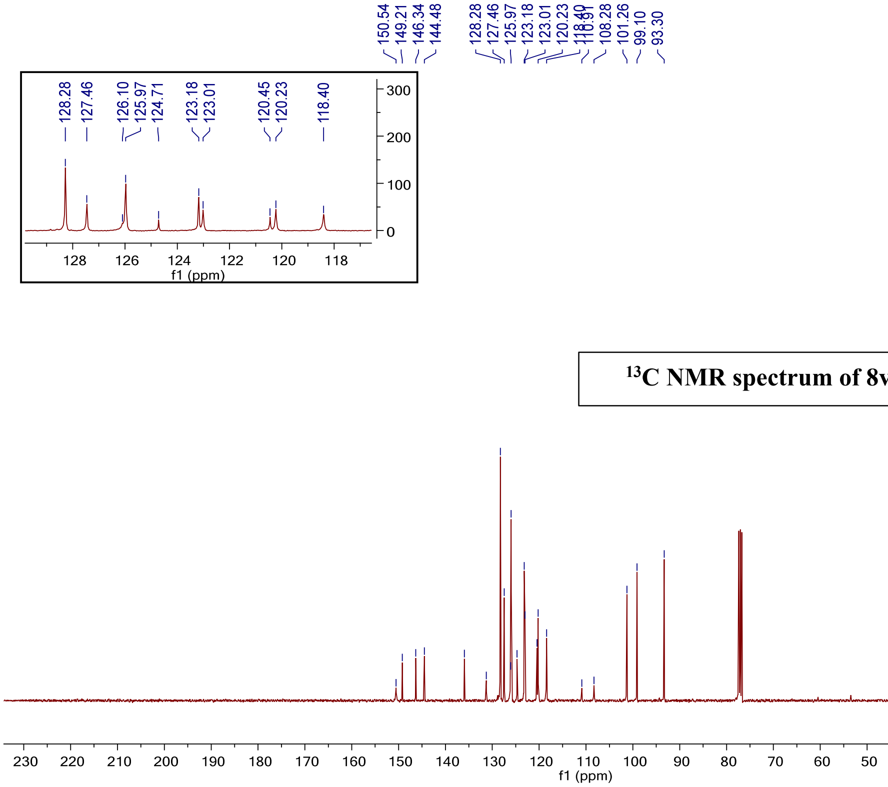

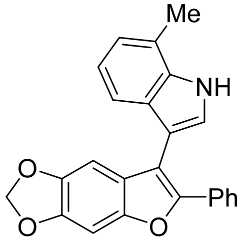

8v

^{13}C NMR spectrum of 8v (100 MHz, $CDCl_3$)

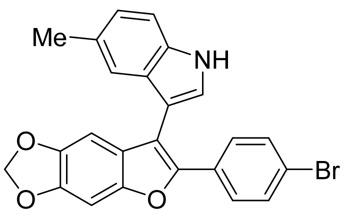

8w

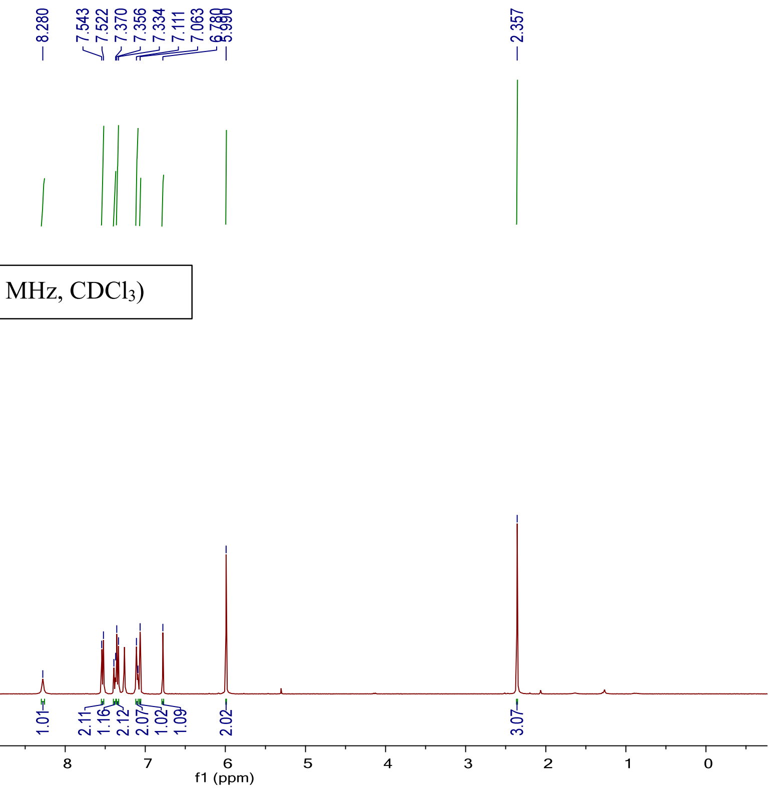

^{1}H NMR spectrum of 8w (400 MHz, $CDCl_3$)

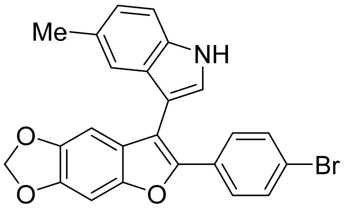

8w

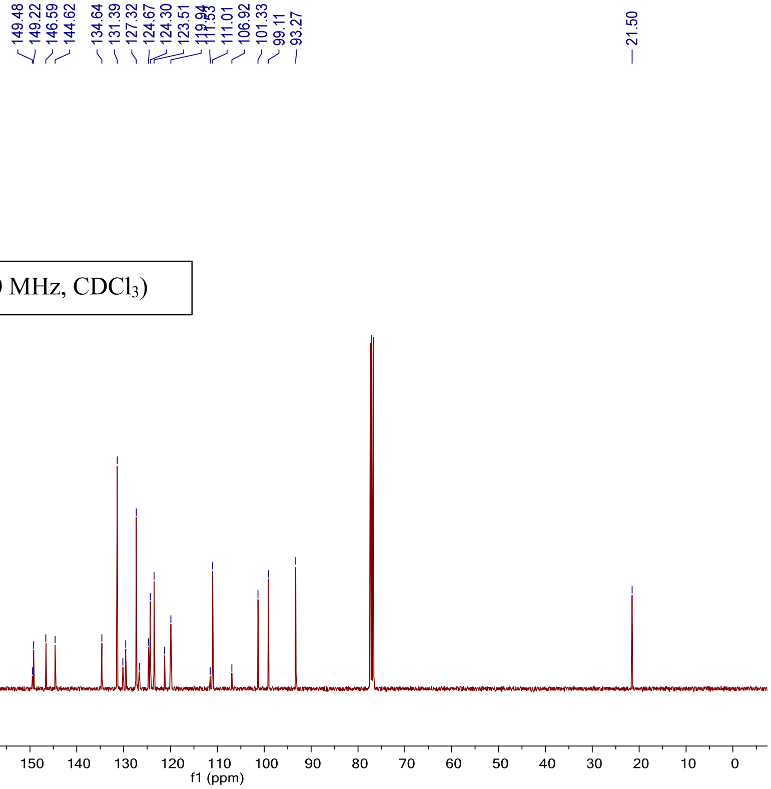

^{13}C NMR spectrum of 8w (100 MHz, $CDCl_3$)

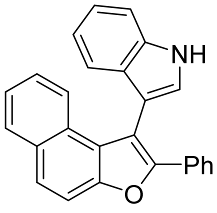

8x

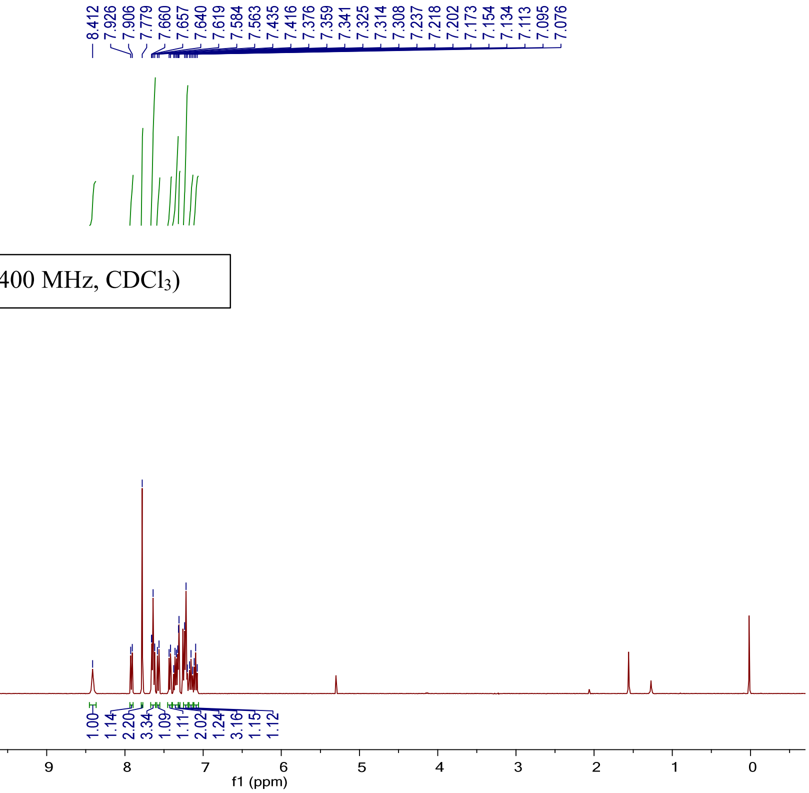

^{1}H NMR spectrum of 8x (400 MHz, $CDCl_3$)

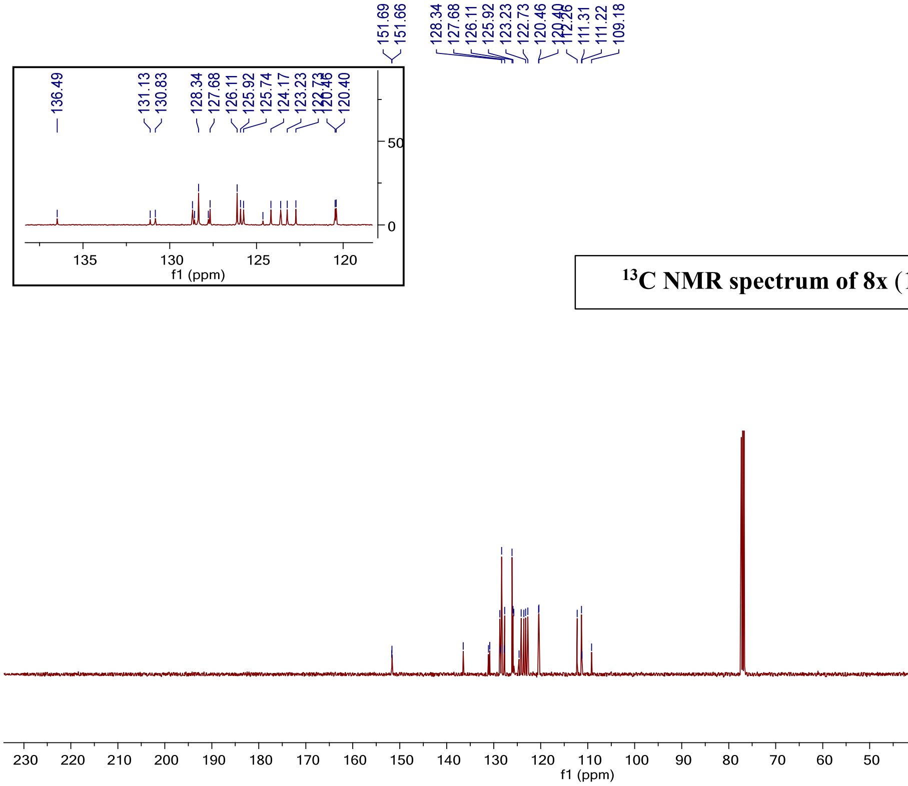

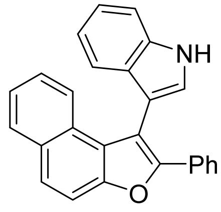

8x

^{13}C NMR spectrum of 8x (100 MHz, $CDCl_3$)

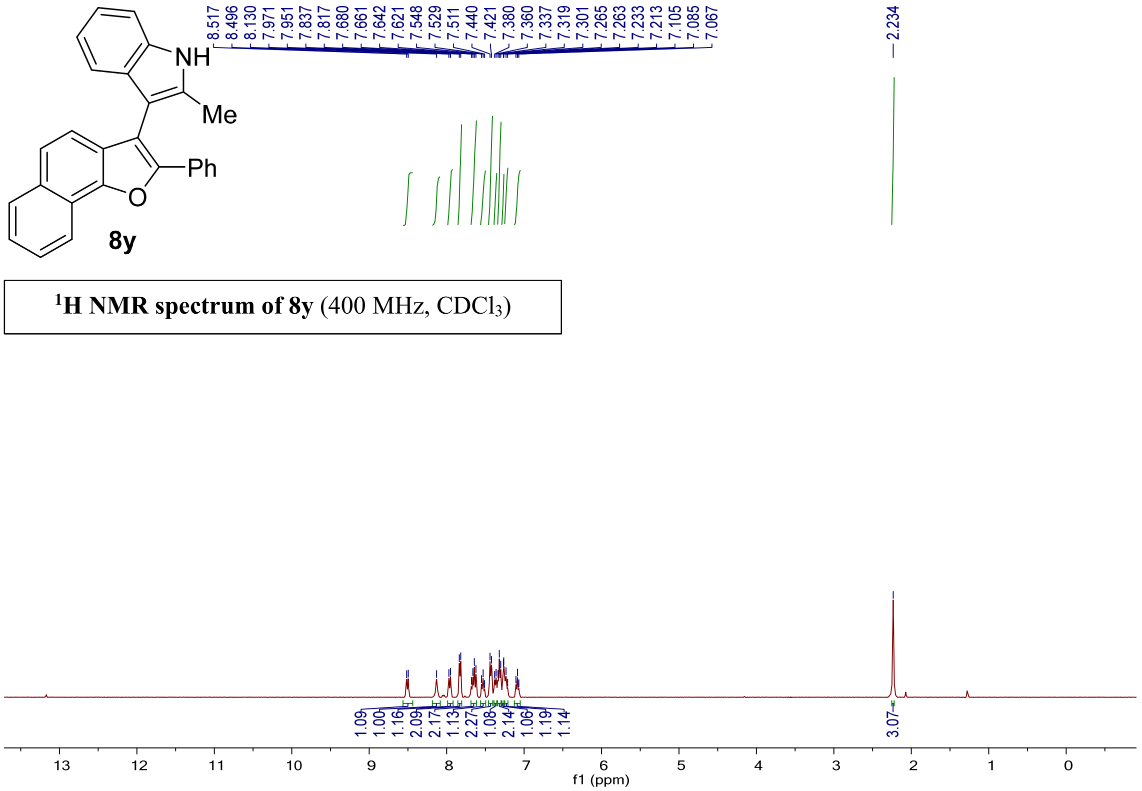

^{1}H NMR spectrum of 8y (400 MHz, $CDCl_3$)

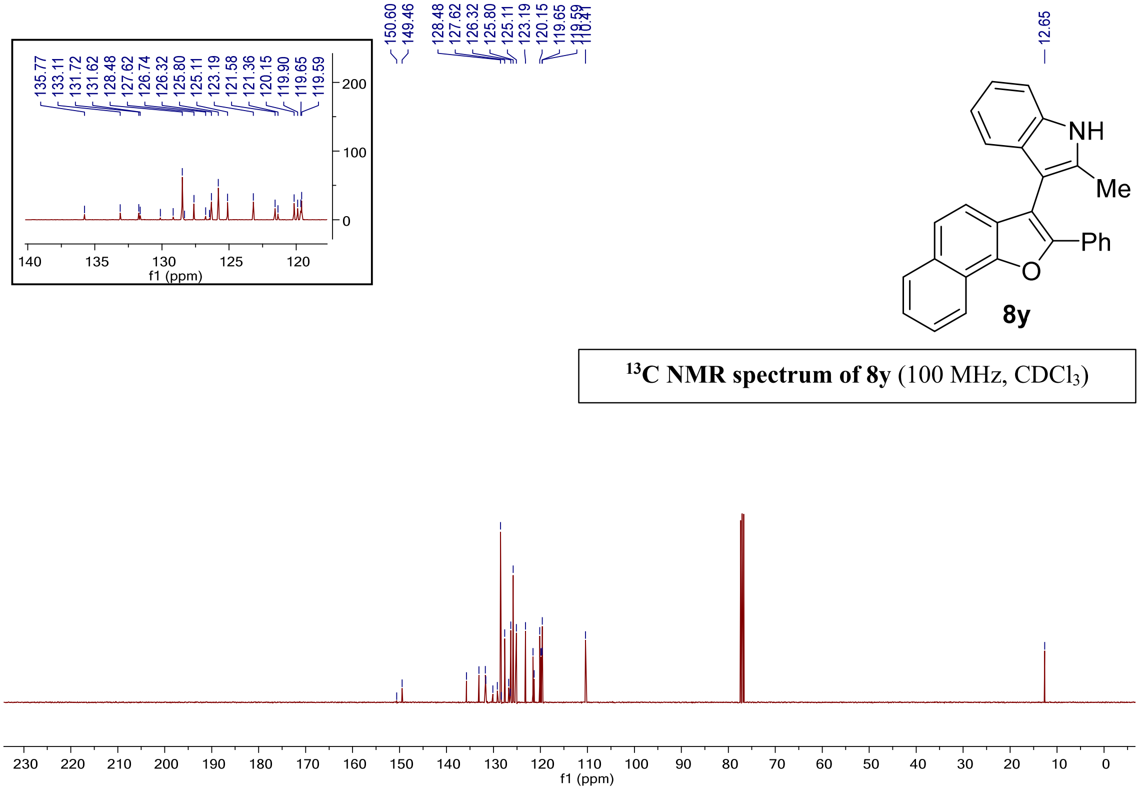

^{13}C NMR spectrum of 8y (100 MHz, $CDCl_3$)

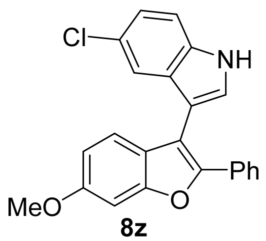

8z

^{1}H NMR spectrum of 8z (400 MHz, $CDCl_3$)

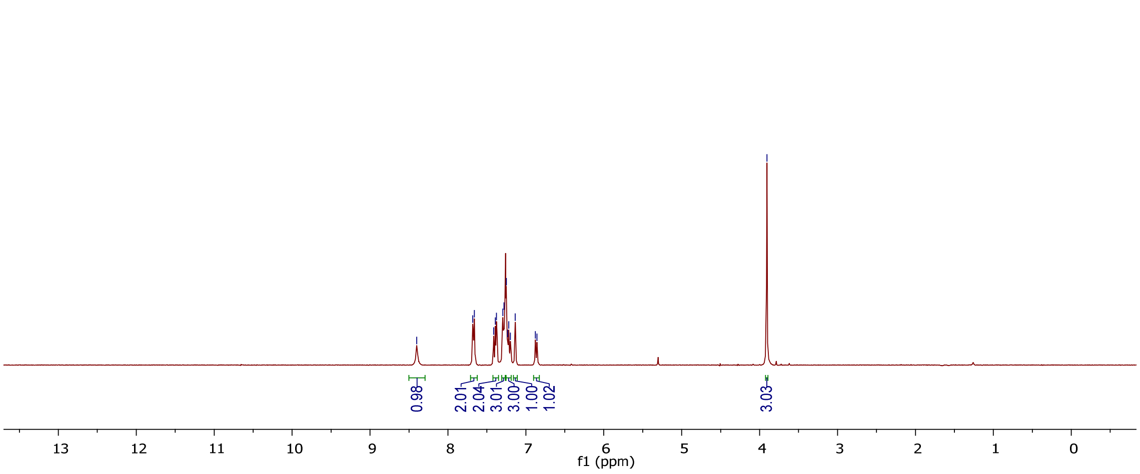

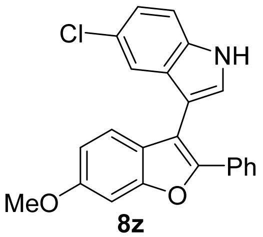

8z

^{13}C NMR spectrum of 8z (100 MHz, $CDCl_3$)

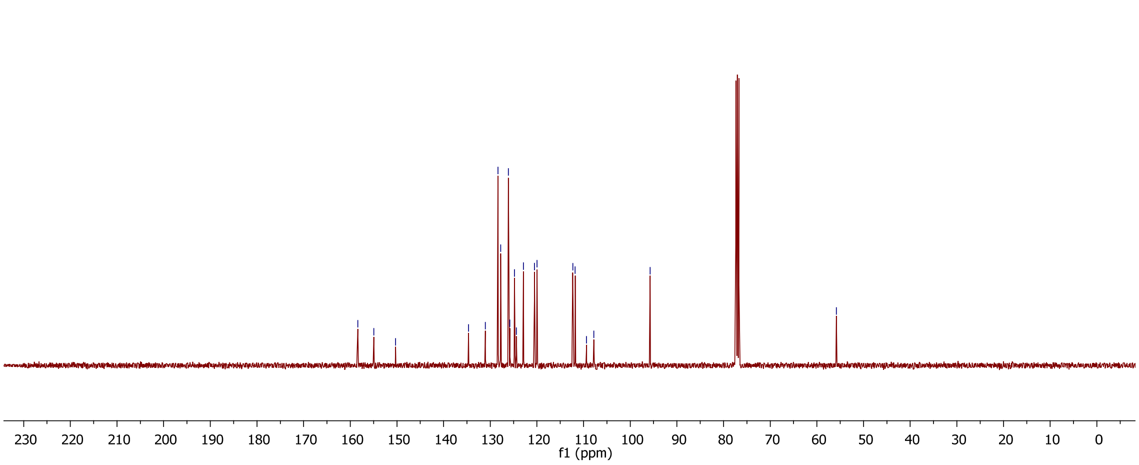

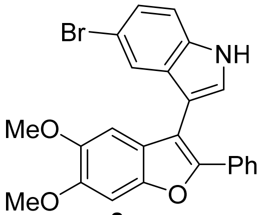

8aa

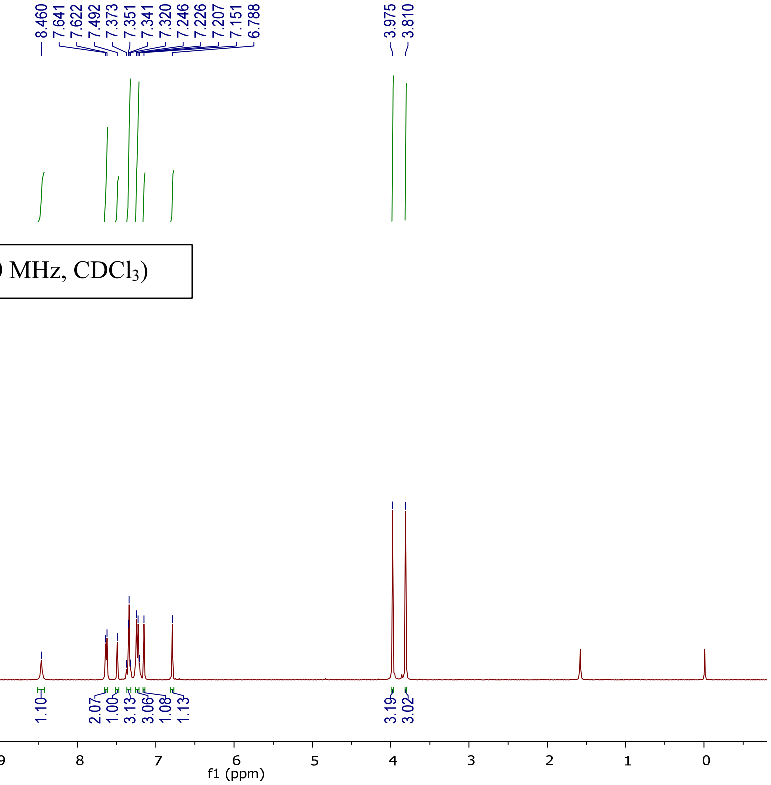

^{1}H NMR spectrum of 8aa (400 MHz, $CDCl_3$)

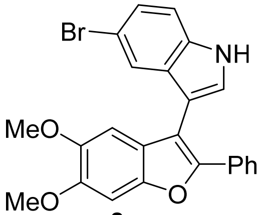

8aa

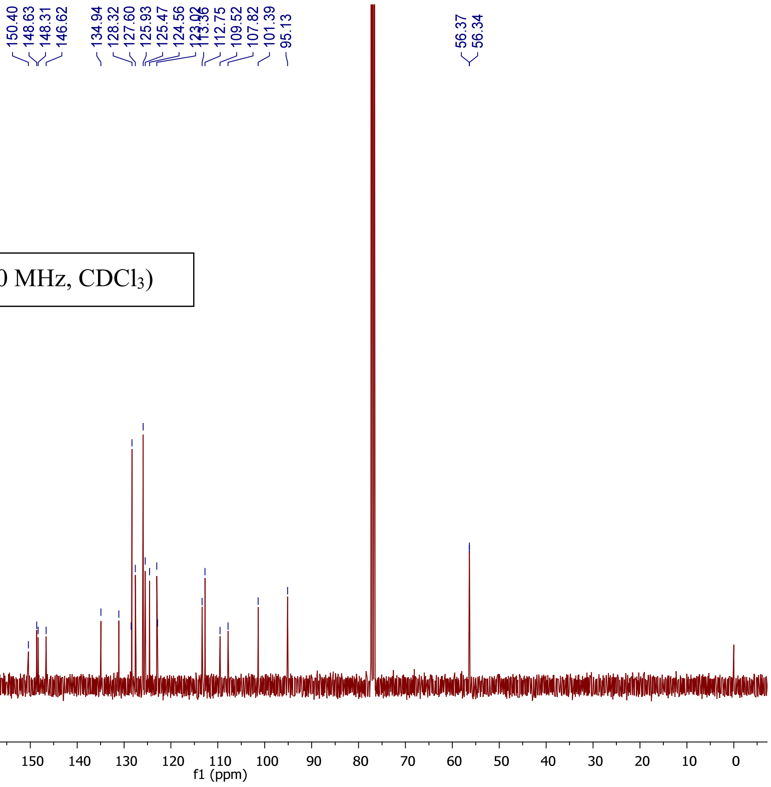

^{13}C NMR spectrum of 8aa (100 MHz, $CDCl_3$)

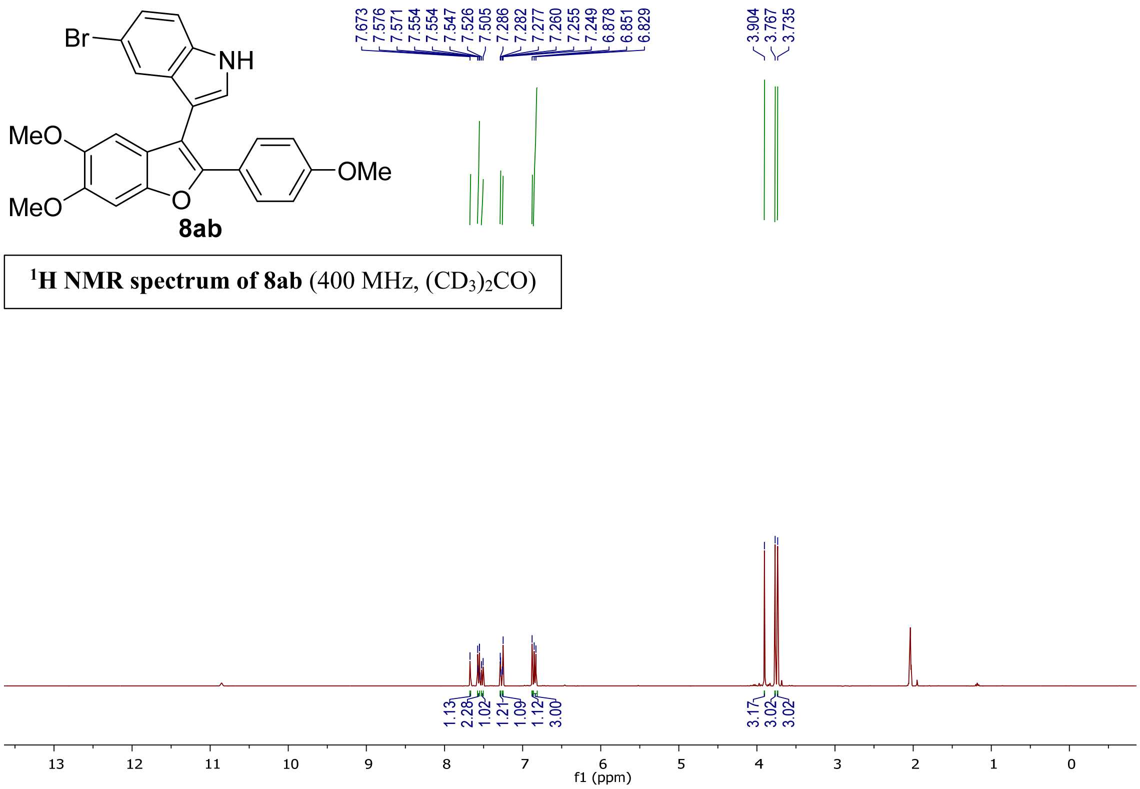

^{1}H NMR spectrum of 8ab (400 MHz, $(CD_3)_2CO$)

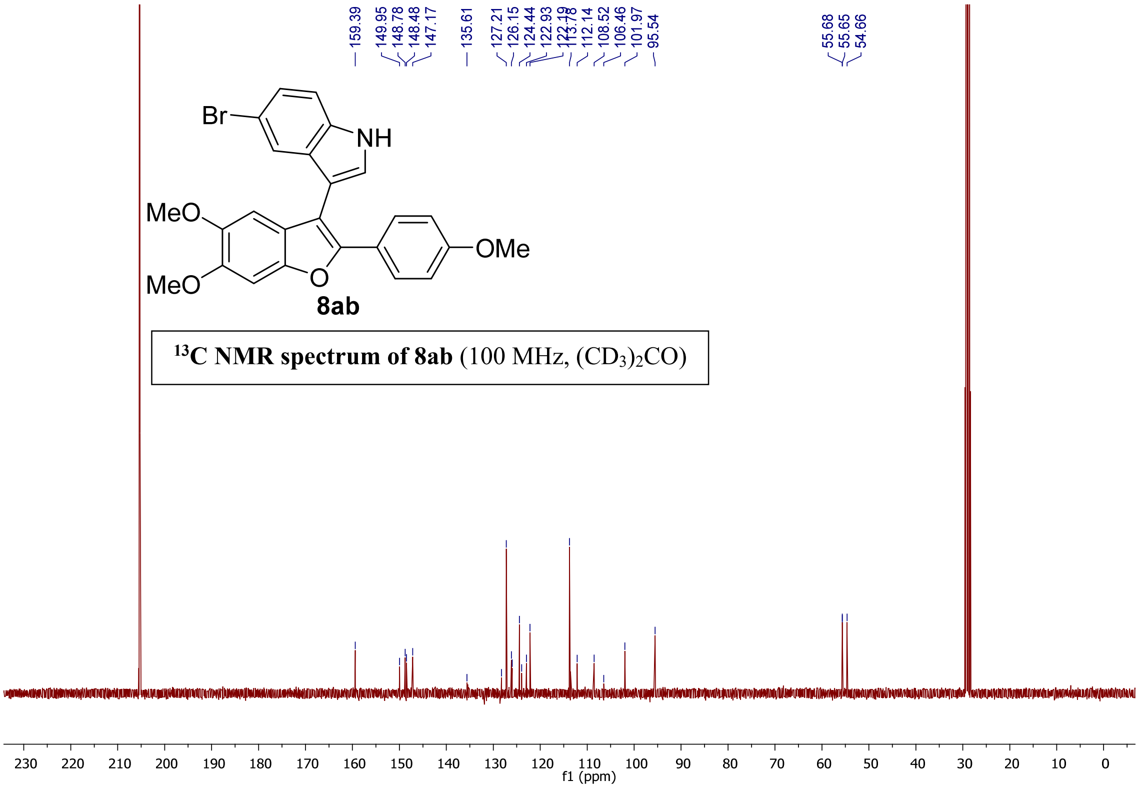

^{13}C NMR spectrum of 8ab (100 MHz, $(CD_3)_2CO$)

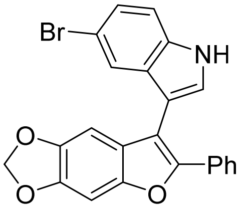

8ac

^{1}H NMR spectrum of 8ac (400 MHz, $CDCl_3$)

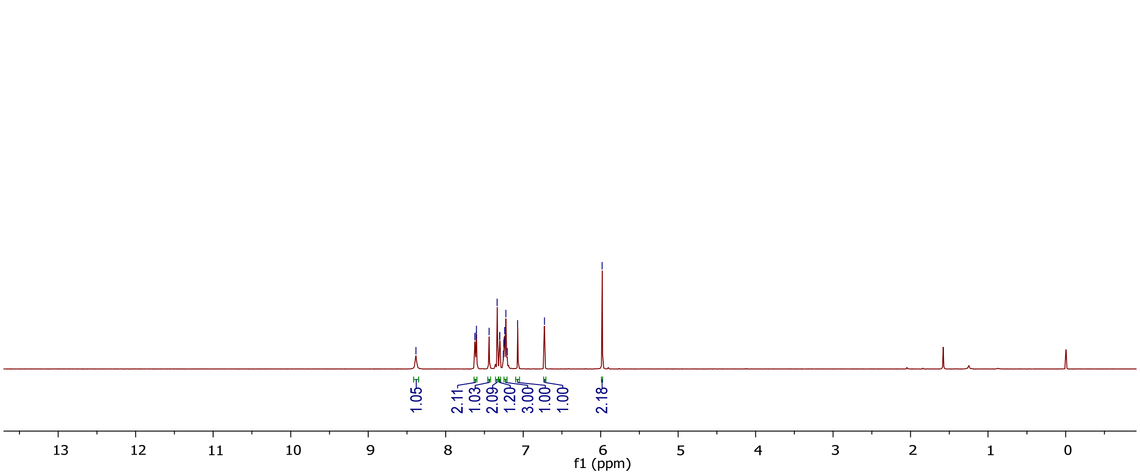

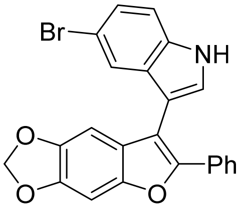

8ac

^{13}C NMR spectrum of 8ac (100 MHz, $CDCl_3$)

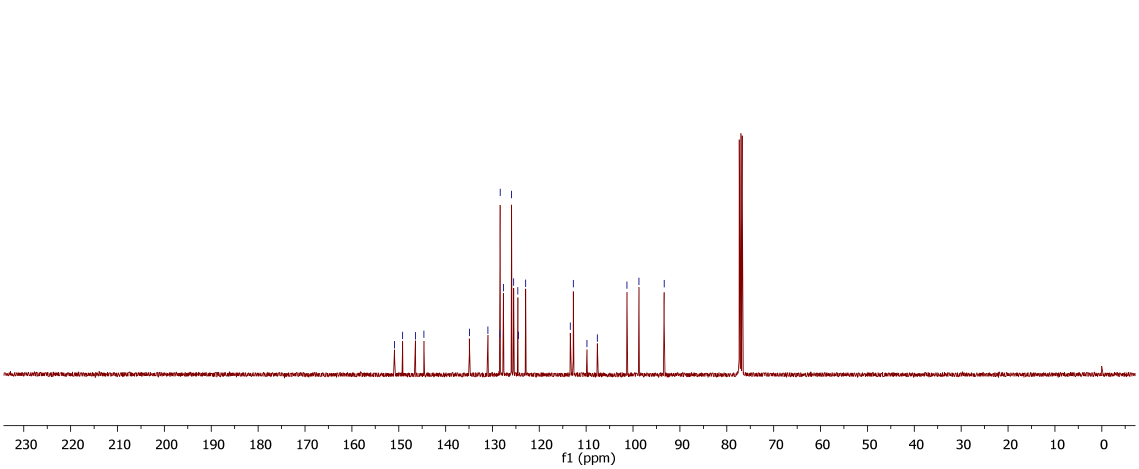

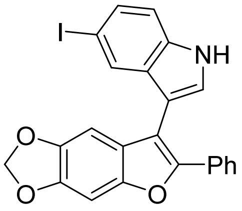

8ad

^{1}H NMR spectrum of 8ad (400 MHz, $CDCl_3$)

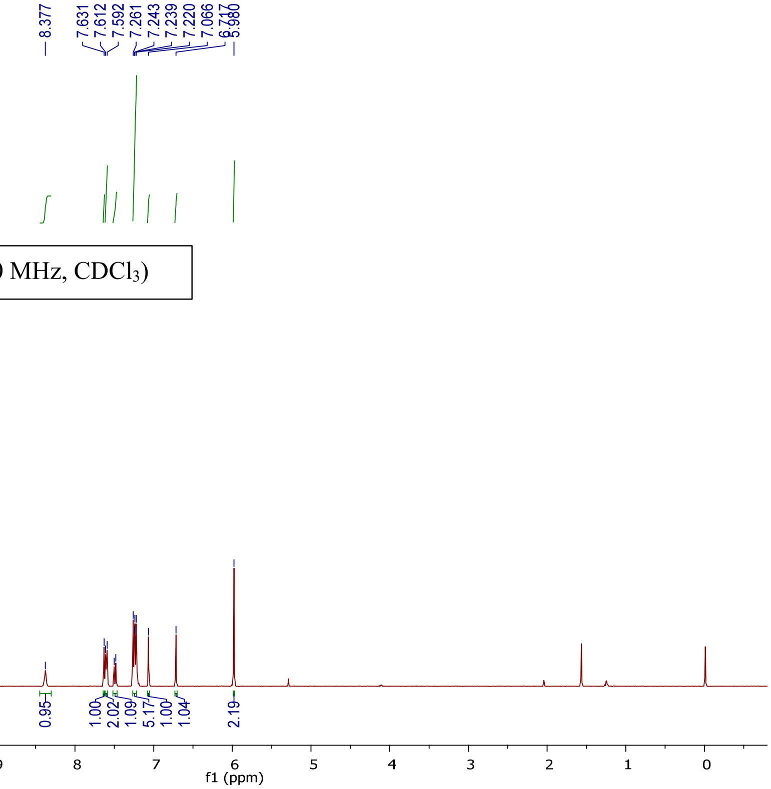

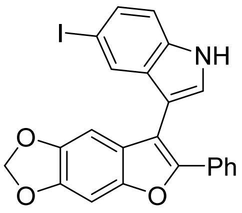

8ad

^{13}C NMR spectrum of 8ad (100 MHz, $CDCl_3$)

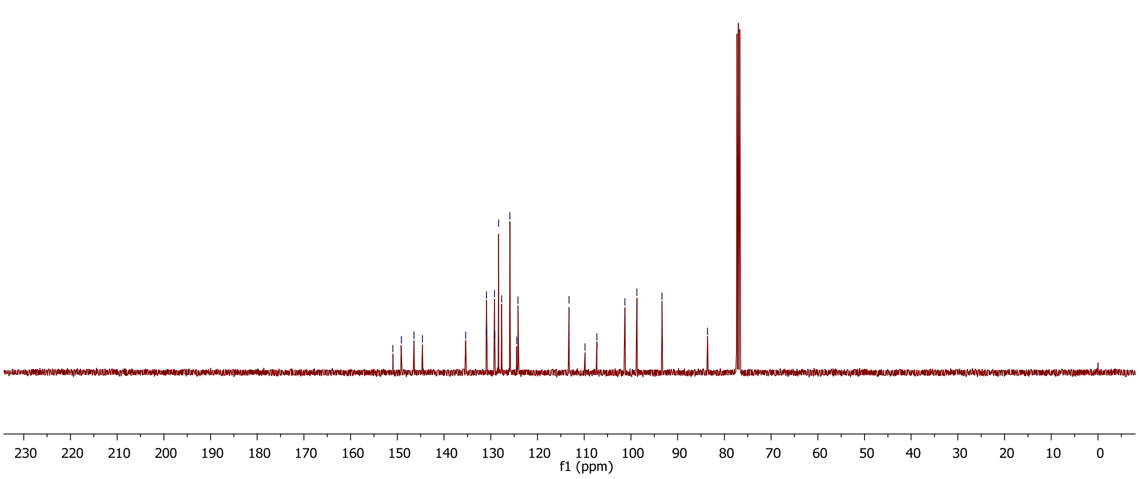

Data Filename 7a.d ACQ Method 506k_leesunhee.m Comment Acquired Time 5/19/2023 3:48:43 PM

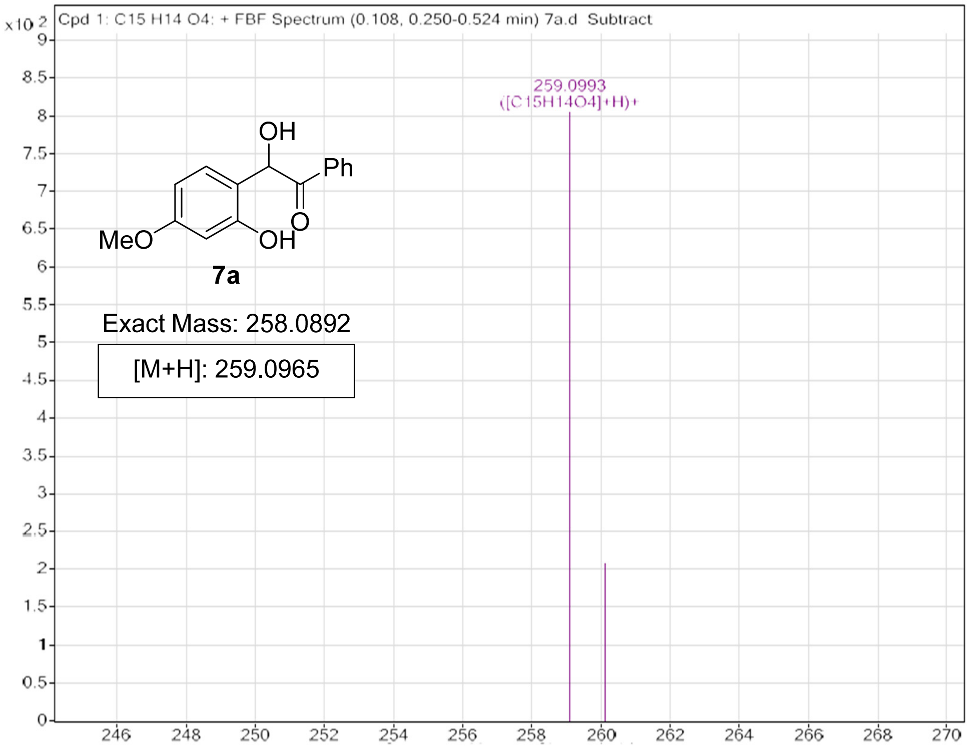

Data Filename 7b.d ACQ Method 506k_leesunhee.m Comment Acquired Time 5/19/2023 3:52:27 PM

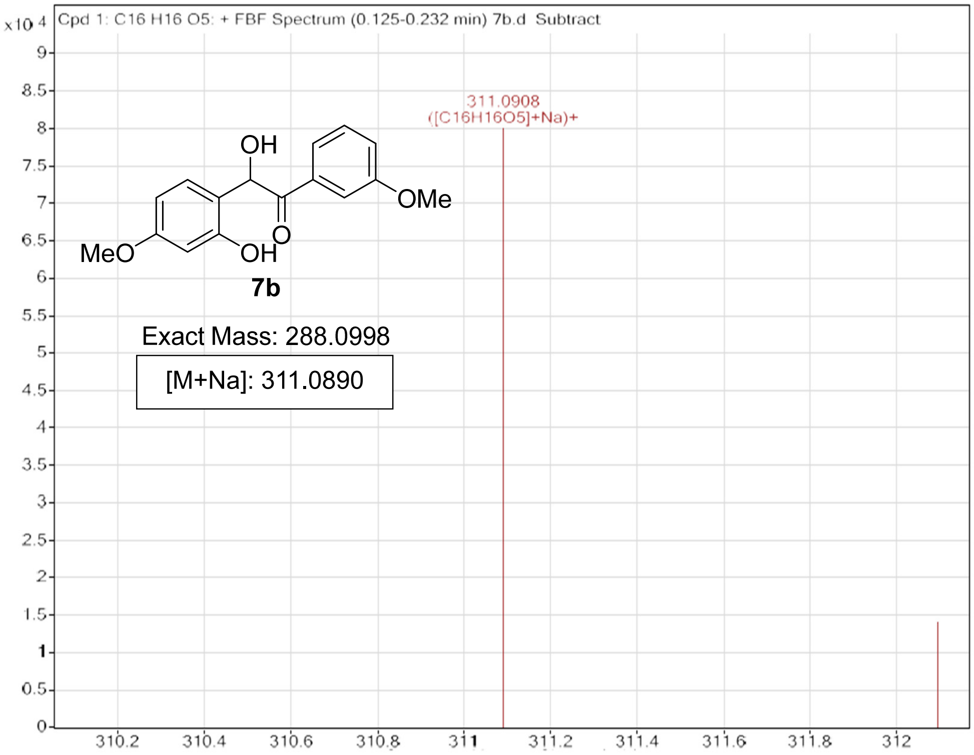

Data Filename 7c.d ACQ Method 506k_leesunhee.m_A2. Comment Acquired Time 1/29/2024 11:41:37 AM

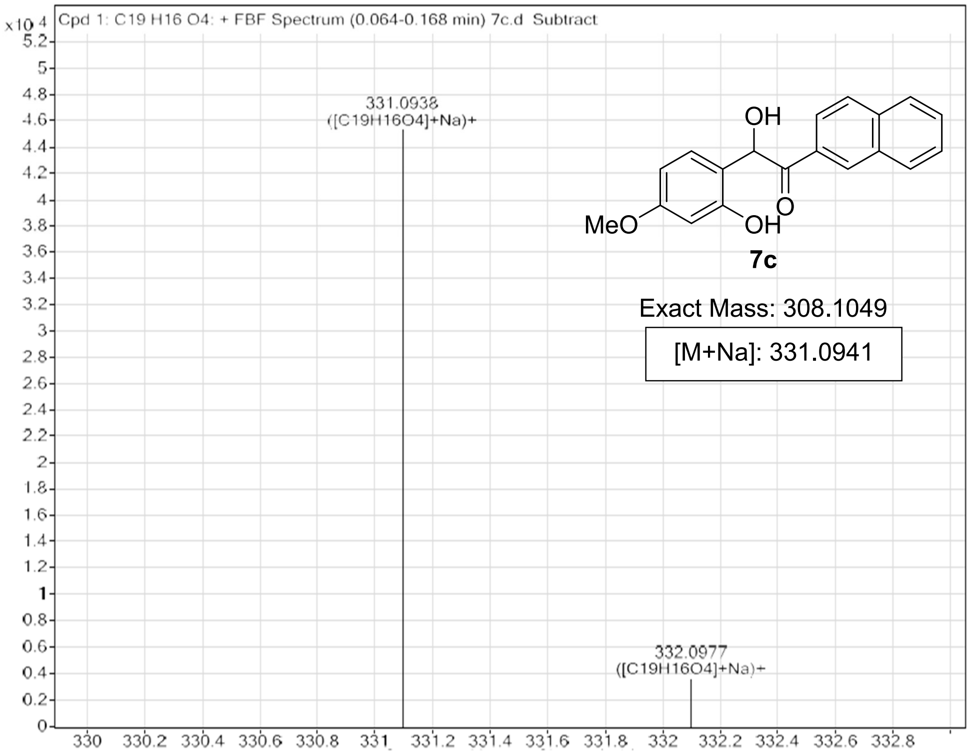

Data Filename 7d.d ACQ Method 506k_leesunhee.m Comment Acquired Time 5/19/2023 3:59:51 PM

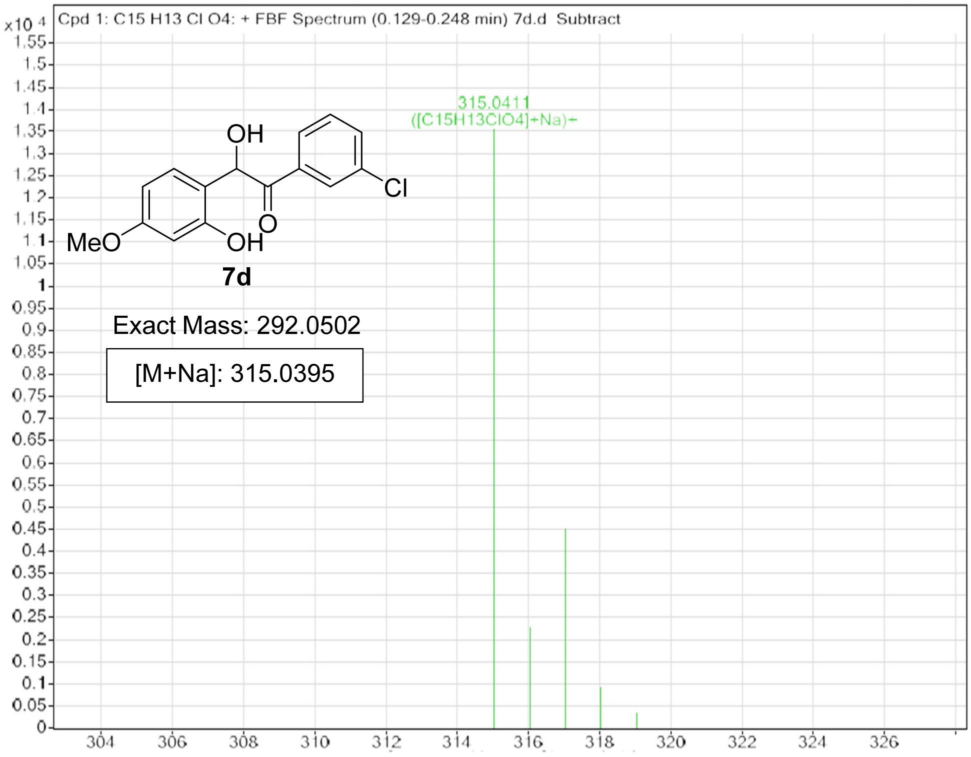

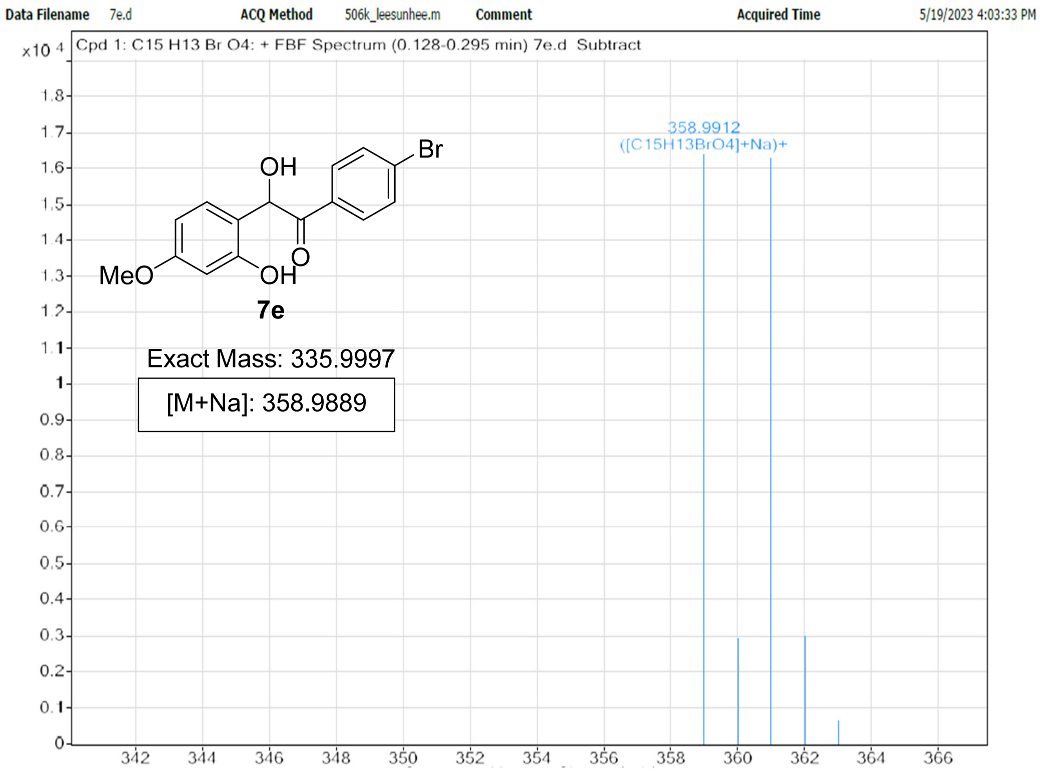
Data Filename 7e.d
ACQ Method 506k_leesunhee.m
Comment
Acquired Time 5/19/2023 4:03:33 PM
Cpd 1: C15 H13 Br O4: + FBF Spectrum (0.128-0.295 min) 7e.d Subtract
358.9912
([C15H13BrO4]+Na)+
OH
Br
MeO
OH
O
7e
Exact Mass: 335.9997
[M+Na]: 358.9889

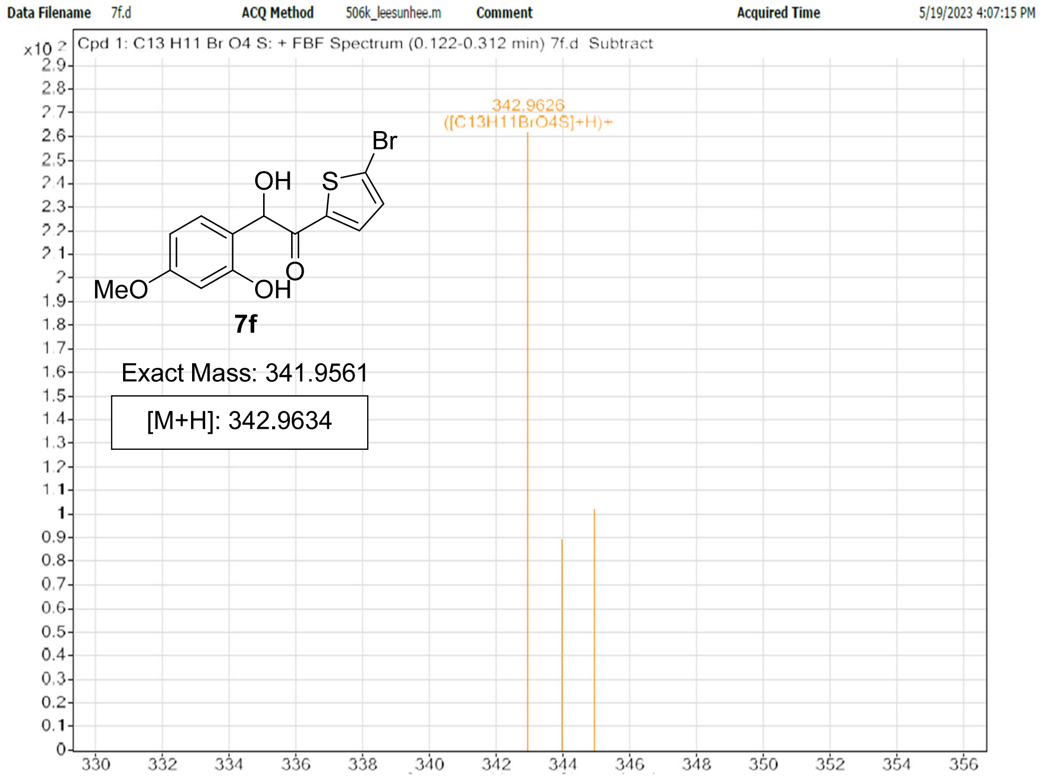
Data Filename 7f.d
ACQ Method 506k_leesunhee.m
Comment
Acquired Time 5/19/2023 4:07:15 PM
Cpd 1: C13 H11 Br O4 S: + FBF Spectrum (0.122-0.312 min) 7f.d Subtract
342.9626
([C13H11BrO4S]+H)+
Br
OH
S
MeO
OH
O
7f
Exact Mass: 341.9561
[M+H]: 342.9634

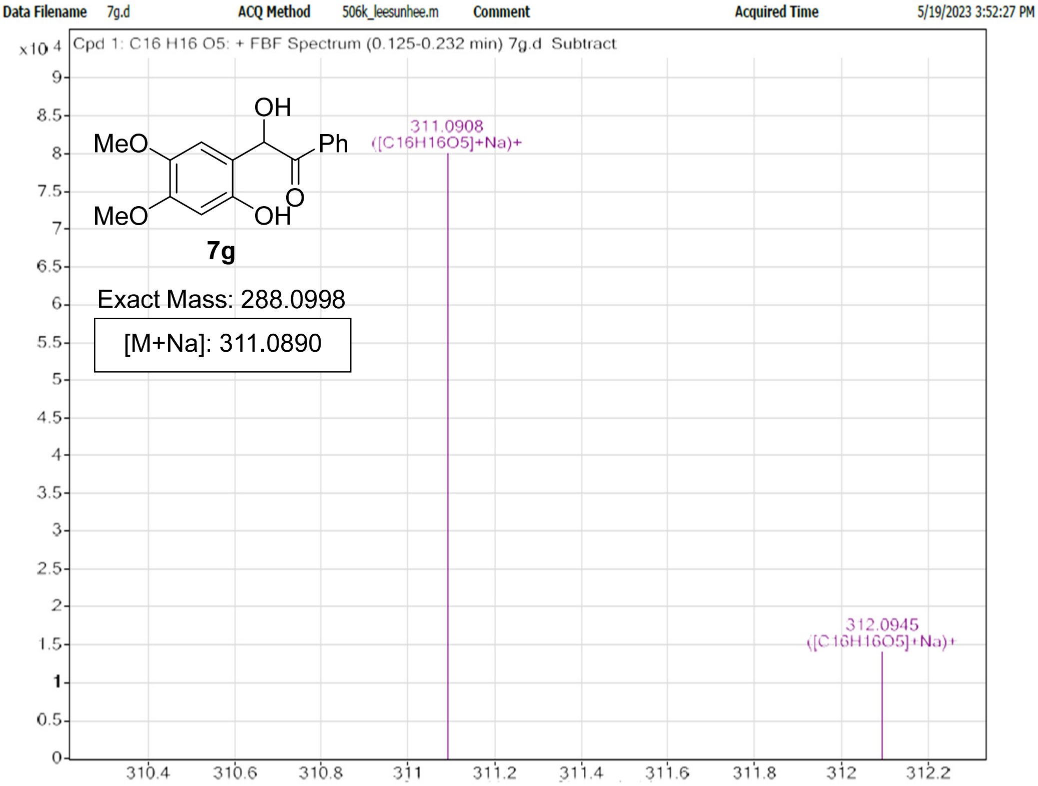
Data Filename 7g.d ACQ Method 506k_leesunhee.m Comment Acquired Time 5/19/2023 3:52:27 PM
x10 4
Cpd 1: C16 H16 O5: + FBF Spectrum (0.125-0.232 min) 7g.d Subtract
MeO
MeO
OH
OH
O
Ph
7g
Exact Mass: 288.0998
[M+Na]: 311.0890
311.0908
([C16H16O5]+Na)+
312.0945
([C16H16O5]+Na)+
310.4
310.6
310.8
311
311.2
311.4
311.6
311.8
312
312.2

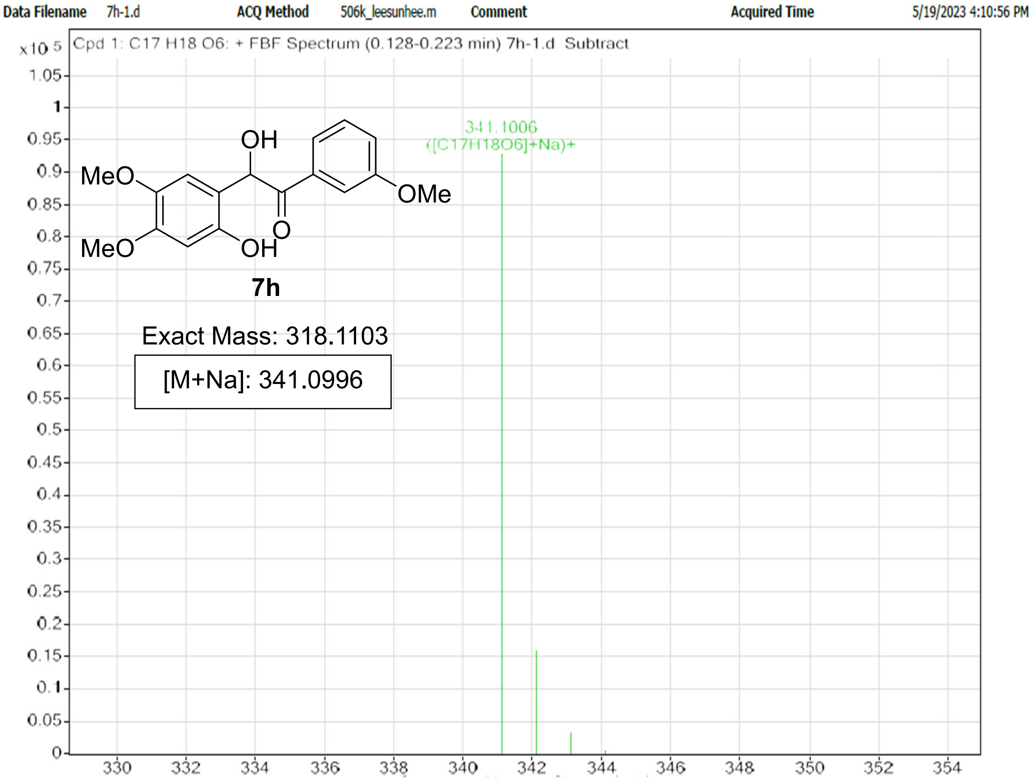
Data Filename 7h-1.d ACQ Method 506k_leesunhee.m Comment Acquired Time 5/19/2023 4:10:56 PM
x10 5
Cpd 1: C17 H18 O6: + FBF Spectrum (0.128-0.223 min) 7h-1.d Subtract
MeO
MeO
OH
OH
O
OMe
7h
Exact Mass: 318.1103
[M+Na]: 341.0996
341.1006
([C17H18O6]+Na)+
330
332
334
336
338
340
342
344
346
348
350
352
354

Data Filename 7i.d ACQ Method 506k_leesunhee.m Comment Acquired Time 5/19/2023 4:10:56 PM

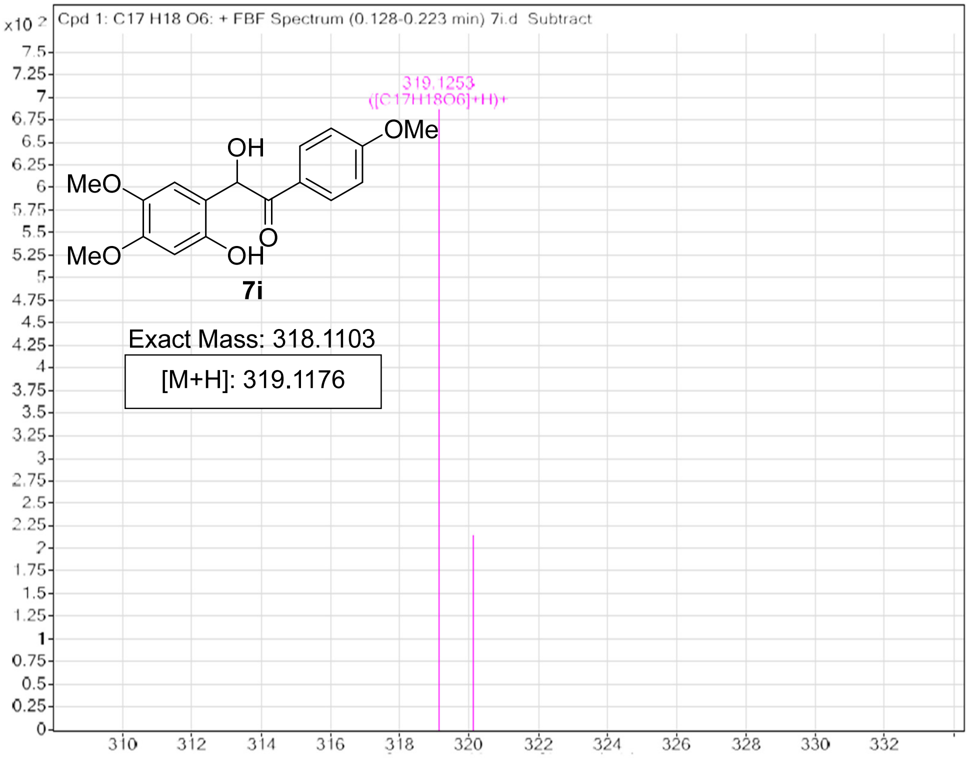

Data Filename 7j.d ACQ Method 506k_leesunhee.m Comment Acquired Time 5/19/2023 4:14:39 PM

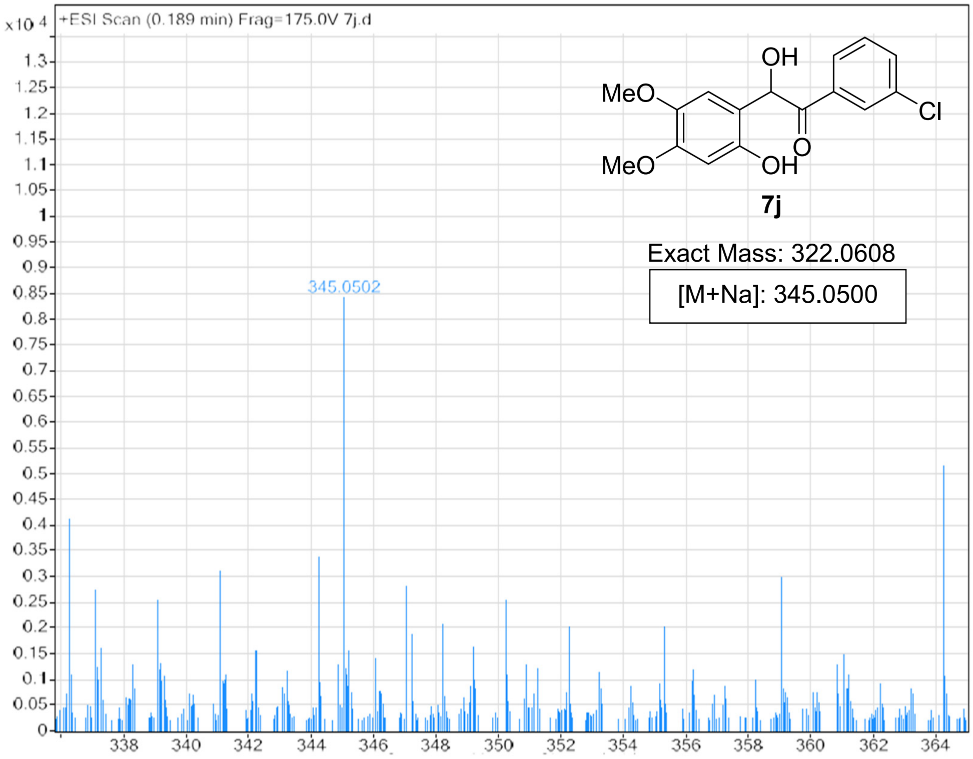

Data Filename 7k.d ACQ Method 506k_leesunhee.m Comment Acquired Time 5/19/2023 4:18:21 PM

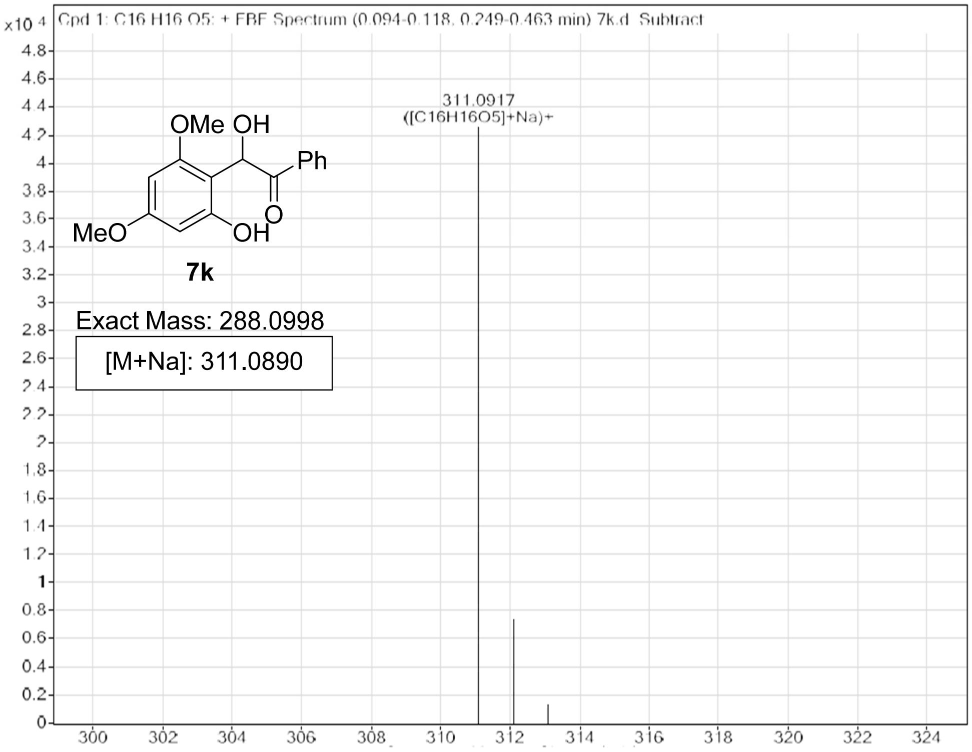

Data Filename 7l.d ACQ Method 506k_leesunhee.m Comment Acquired Time 5/19/2023 4:22:06 PM

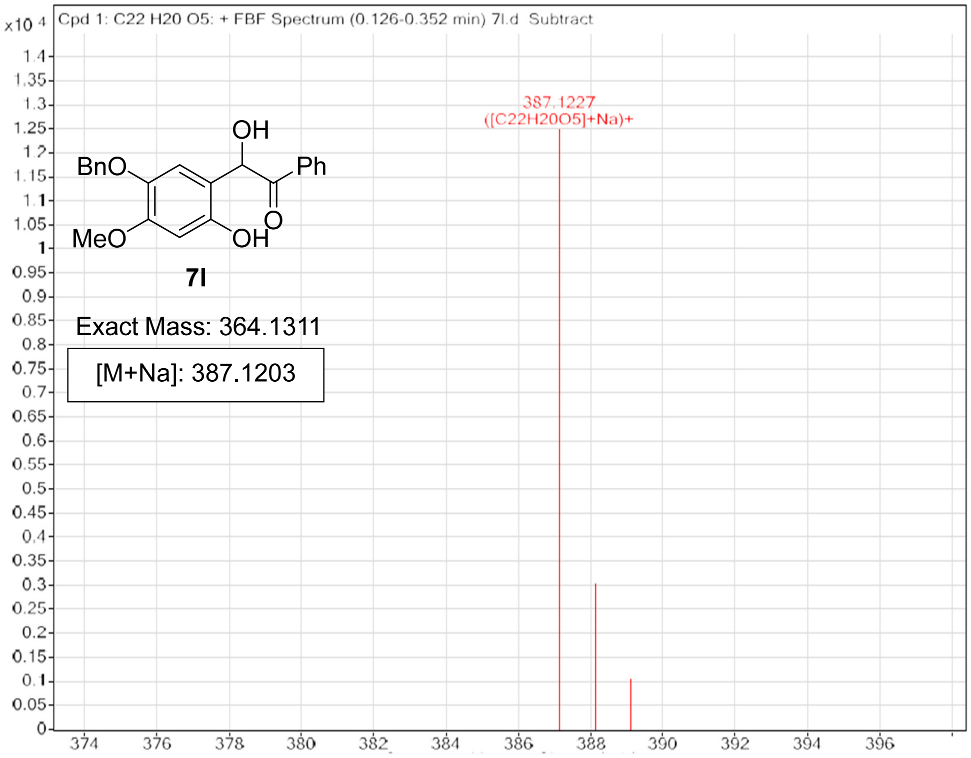

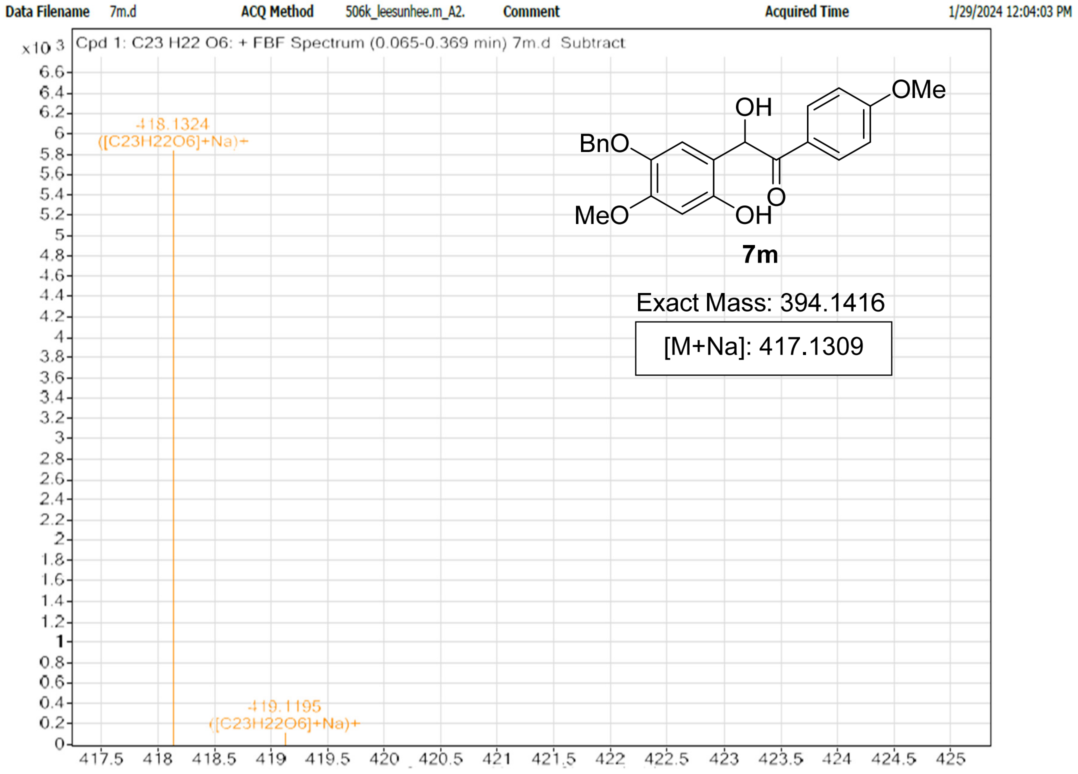

Data Filename 7m.d
ACQ Method 506k_leesunhee.m_A2.
Comment
Acquired Time 1/29/2024 12:04:03 PM
x10 3
Cpd 1: C23 H22 O6: + FBF Spectrum (0.065-0.369 min) 7m.d Subtract
418.1324
([C23H22O6]+Na)+
419.1195
([C23H22O6]+Na)+
BnO
MeO
OH
OH
O
OMe
7m
Exact Mass: 394.1416
[M+Na]: 417.1309

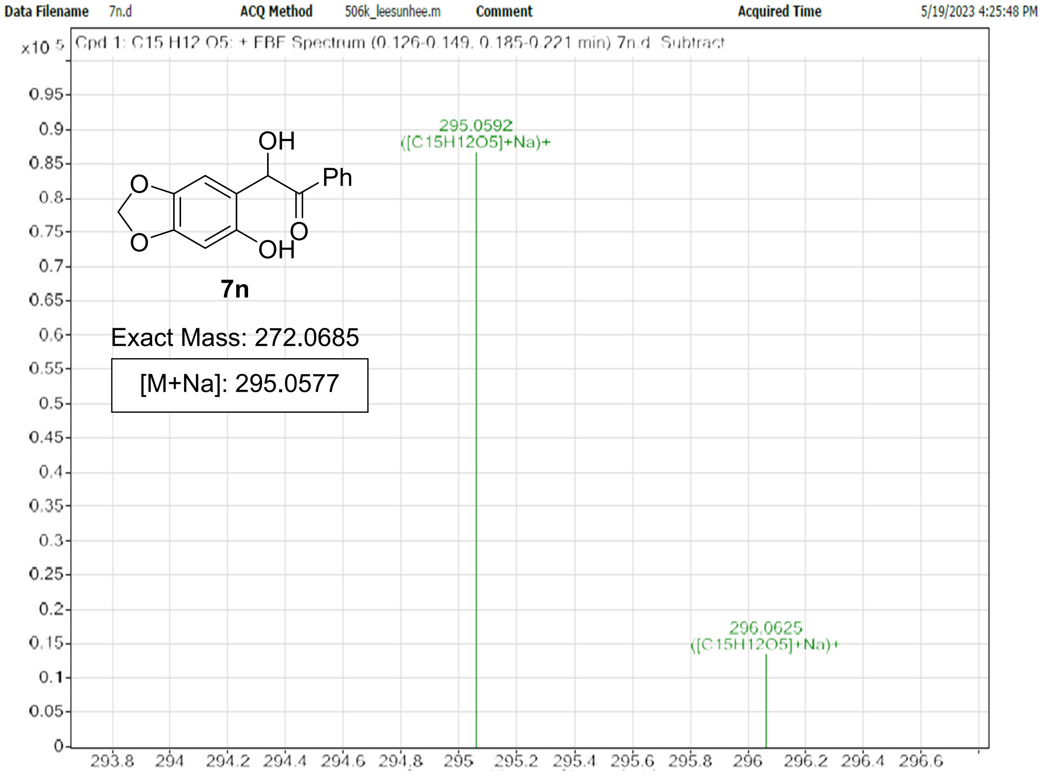

Data Filename 7n.d
ACQ Method 506k_leesunhee.m
Comment
Acquired Time 5/19/2023 4:25:48 PM
x10 5
Cpd 1: C15 H12 O5: + FBF Spectrum (0.126-0.149, 0.185-0.221 min) 7n.d Subtract
295.0592
([C15H12O5]+Na)+
296.0625
([C15H12O5]+Na)+
OH
Ph
O
O
O
OH
7n
Exact Mass: 272.0685
[M+Na]: 295.0577

Data Filename 7o.d ACQ Method 506k_leesunhee.m Comment Acquired Time 5/19/2023 4:29:30 PM

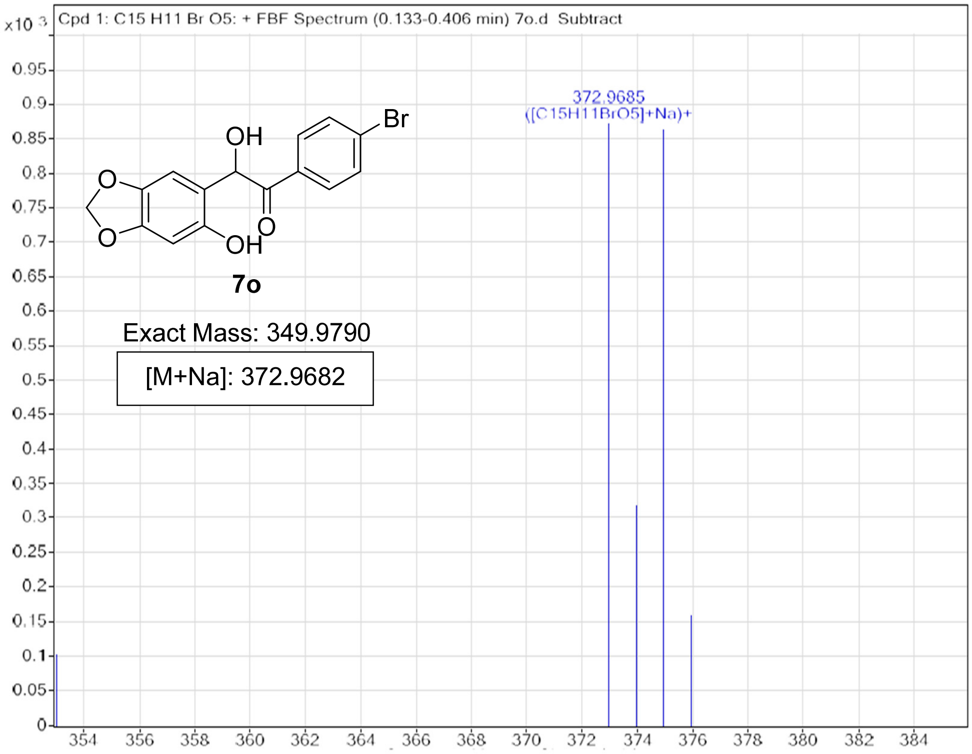

Data Filename 7p.d ACQ Method 506k_leesunhee.m Comment Acquired Time 5/19/2023 4:33:13 PM

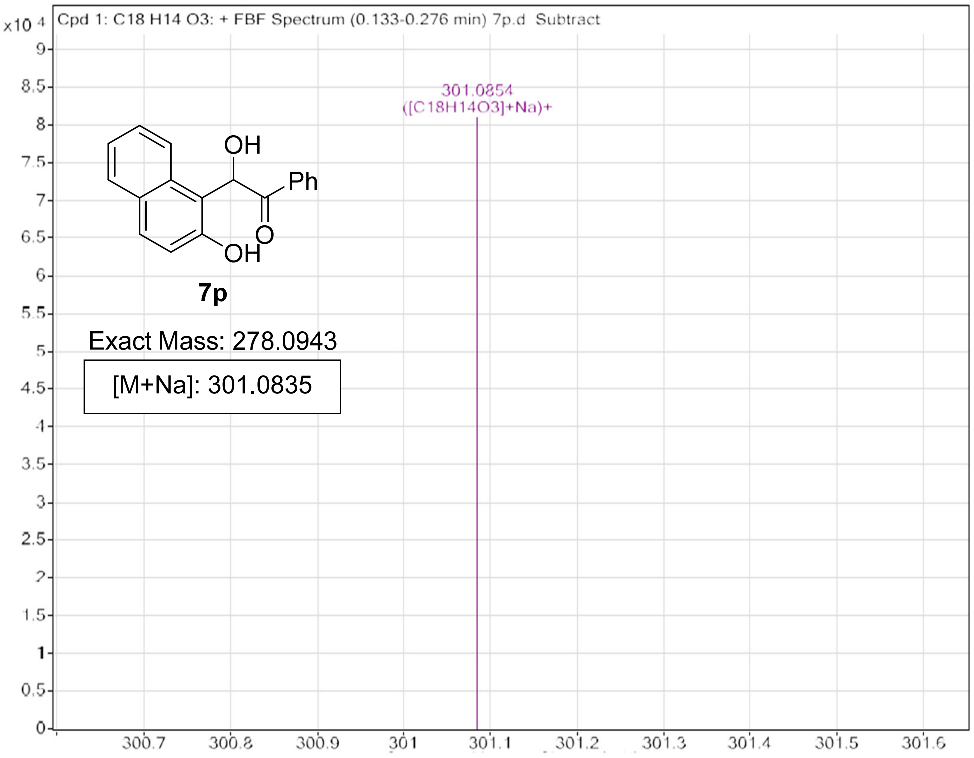

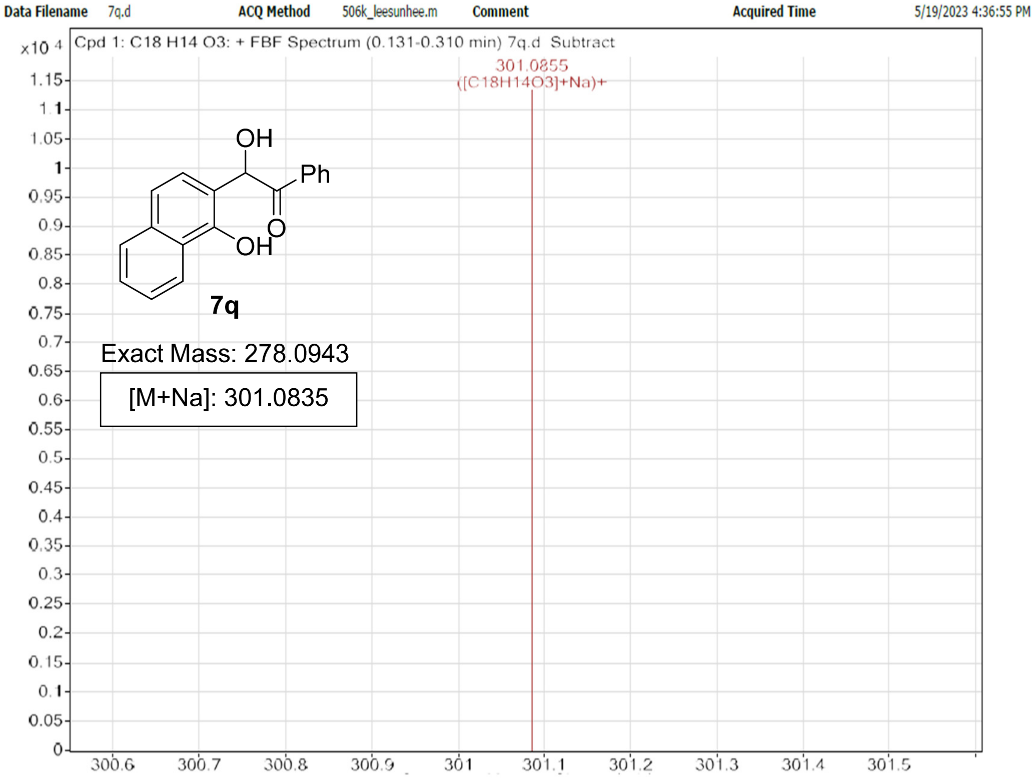
Data Filename 7q.d
ACQ Method 506k_leesunhee.m
Comment
Acquired Time 5/19/2023 4:36:55 PM
Cpd 1: C18 H14 O3: + FBF Spectrum (0.131-0.310 min) 7q.d Subtract
301.0855
([C18H14O3]+Na)+
OH
Ph
O
OH
7q
Exact Mass: 278.0943
[M+Na]: 301.0835

Data Filename 8a.d ACQ Method Comment Sample information is unavailable Acquired Time Unavailable

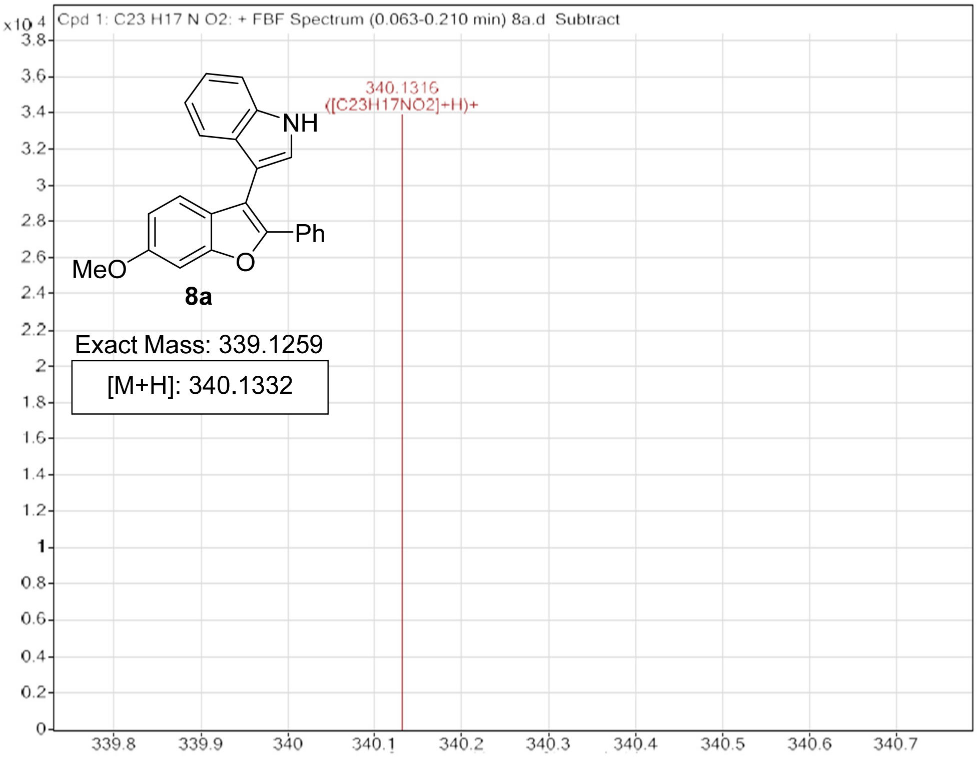

Data Filename 8b.d ACQ Method 506k_leesunhee.m Comment Acquired Time 5/19/2023 4:44:20 PM

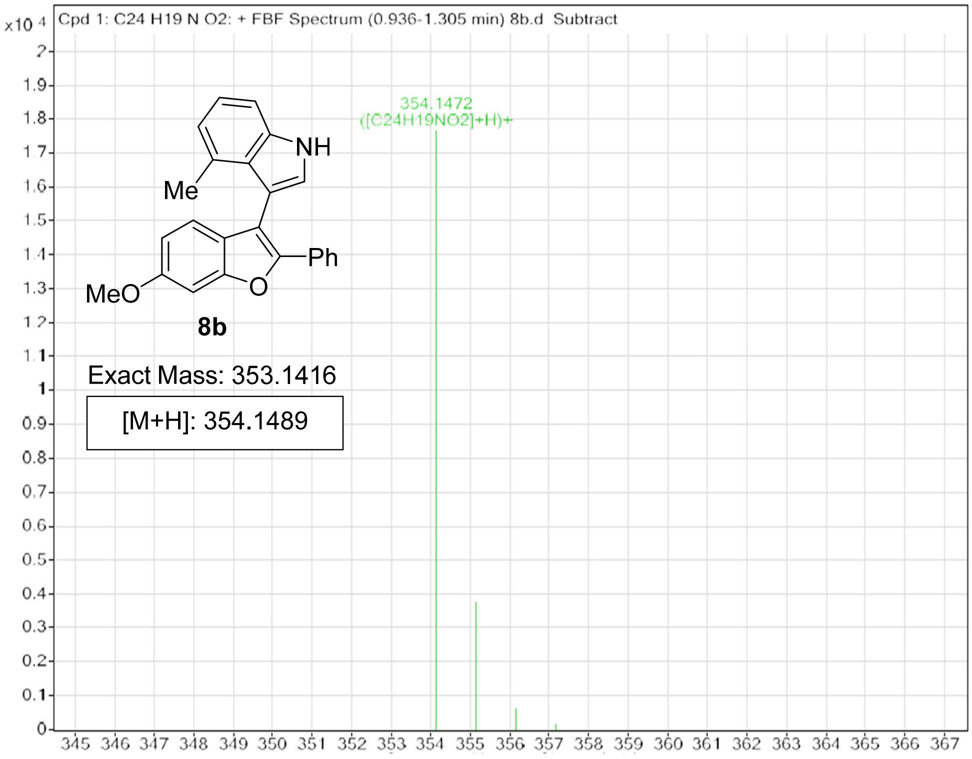

Data Filename 8c.d ACQ Method 506k_leesunhee.m_A2. Comment Acquired Time 1/29/2024 2:40:30 PM

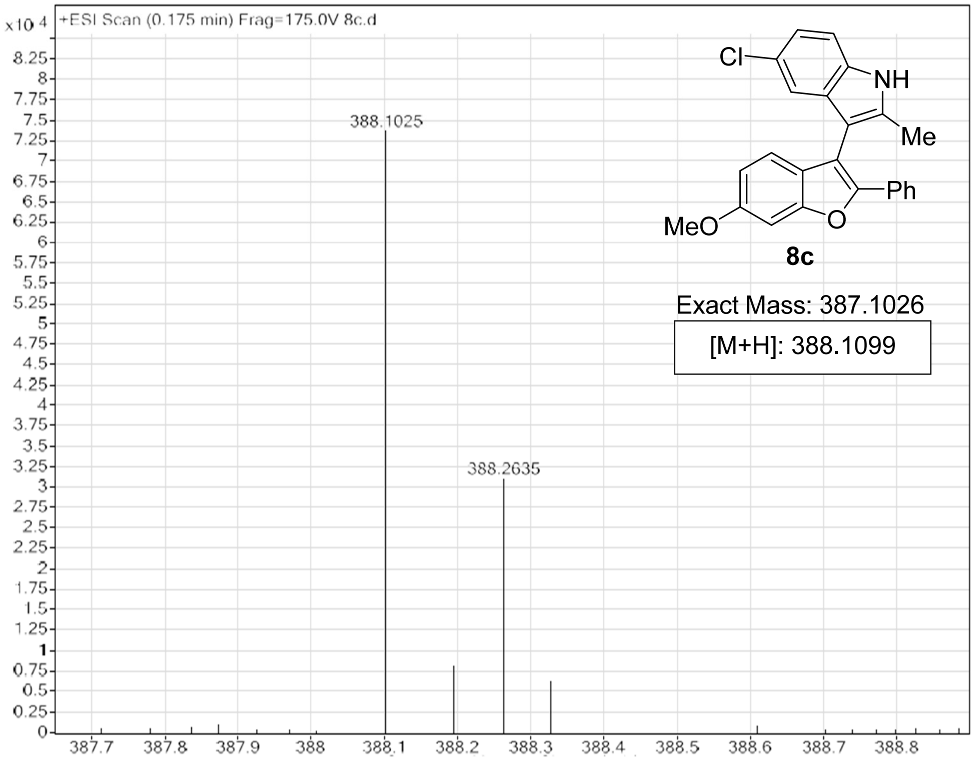

Data Filename 8d.d ACQ Method Comment Sample information is unavailable Acquired Time Unavailable

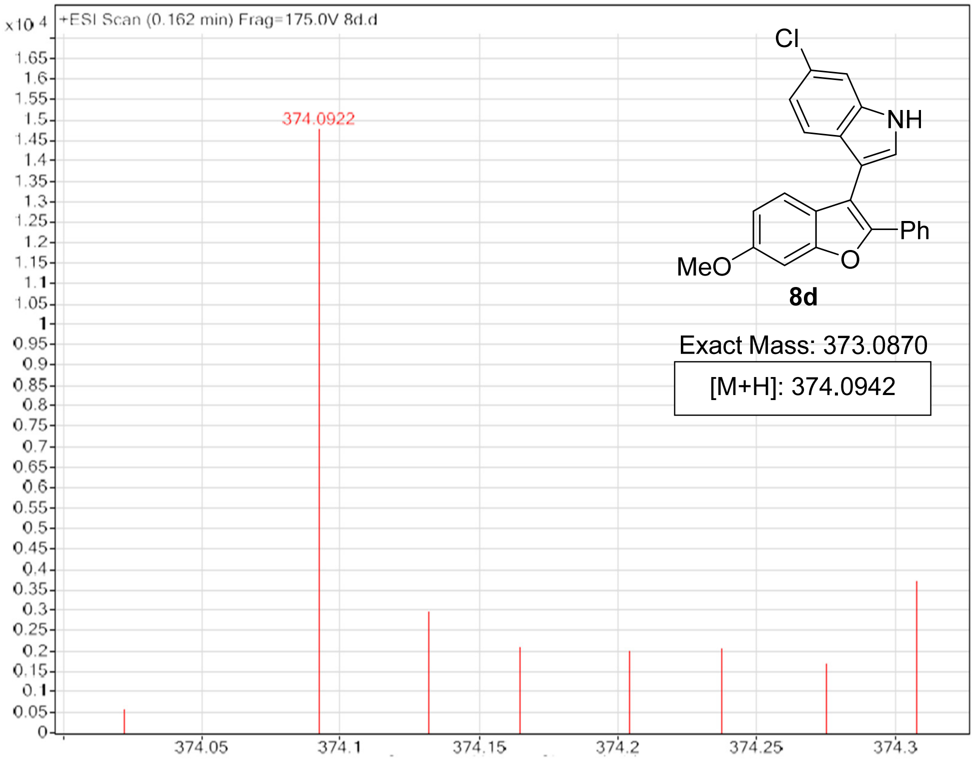

Data Filename 8e.d ACQ Method Comment Sample information is unavailable Acquired Time Unavailable

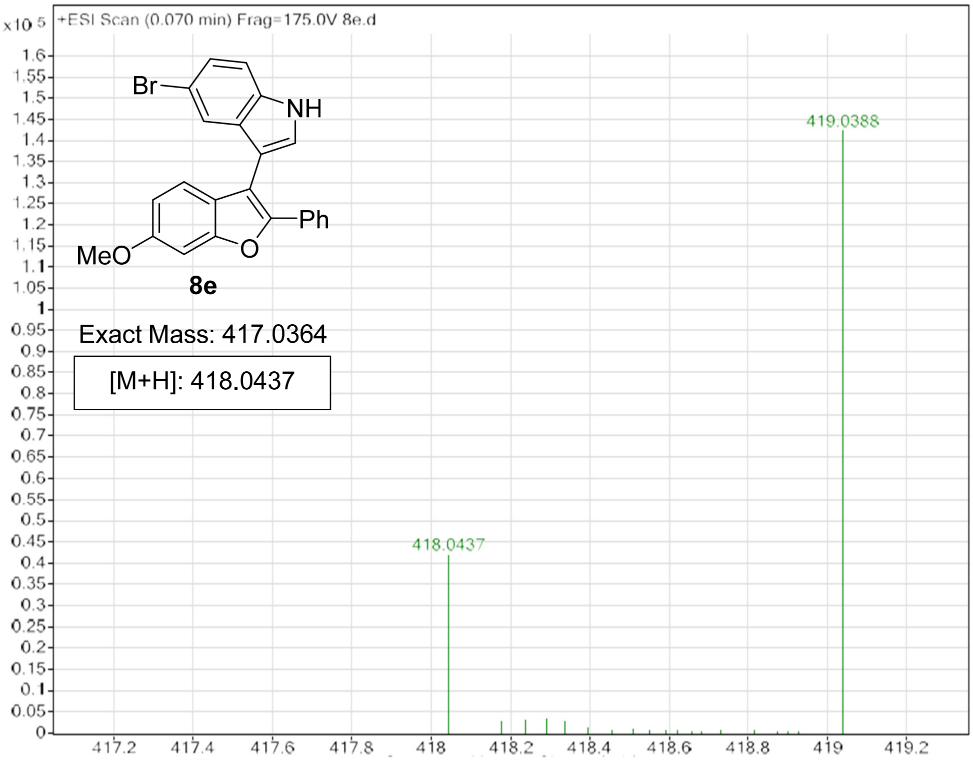

Data Filename 8f.d ACQ Method 506k_leesunhee.m_A2. Comment Acquired Time 1/29/2024 2:51:37 PM

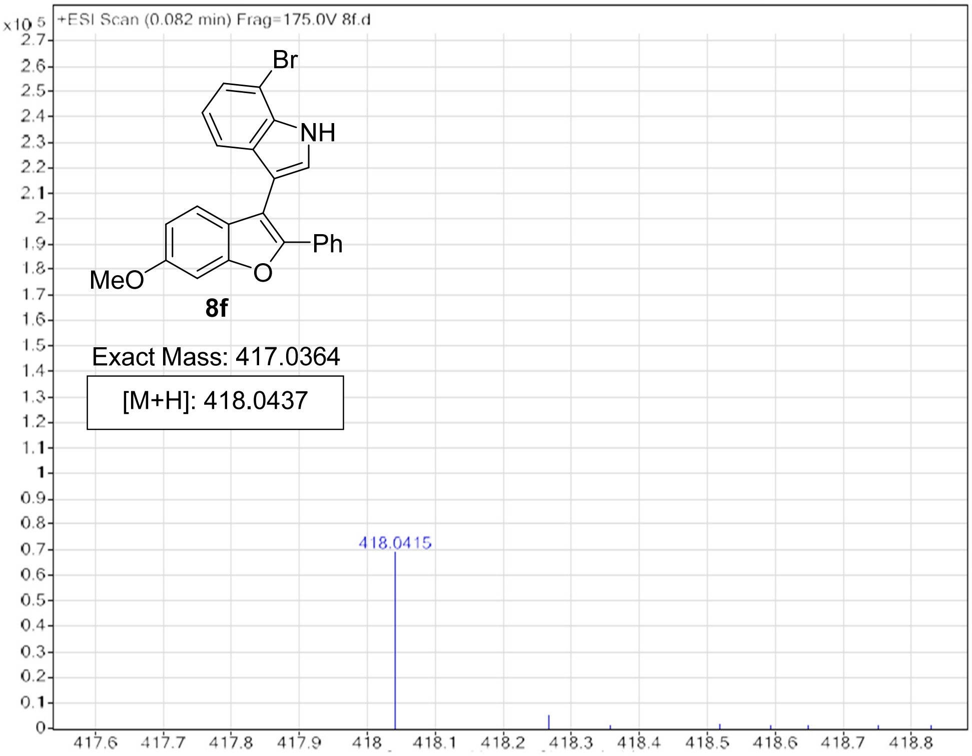

Data Filename 8g.d ACQ Method 506k_leesunhee.m_A2. Comment Acquired Time 1/29/2024 12:26:21 PM

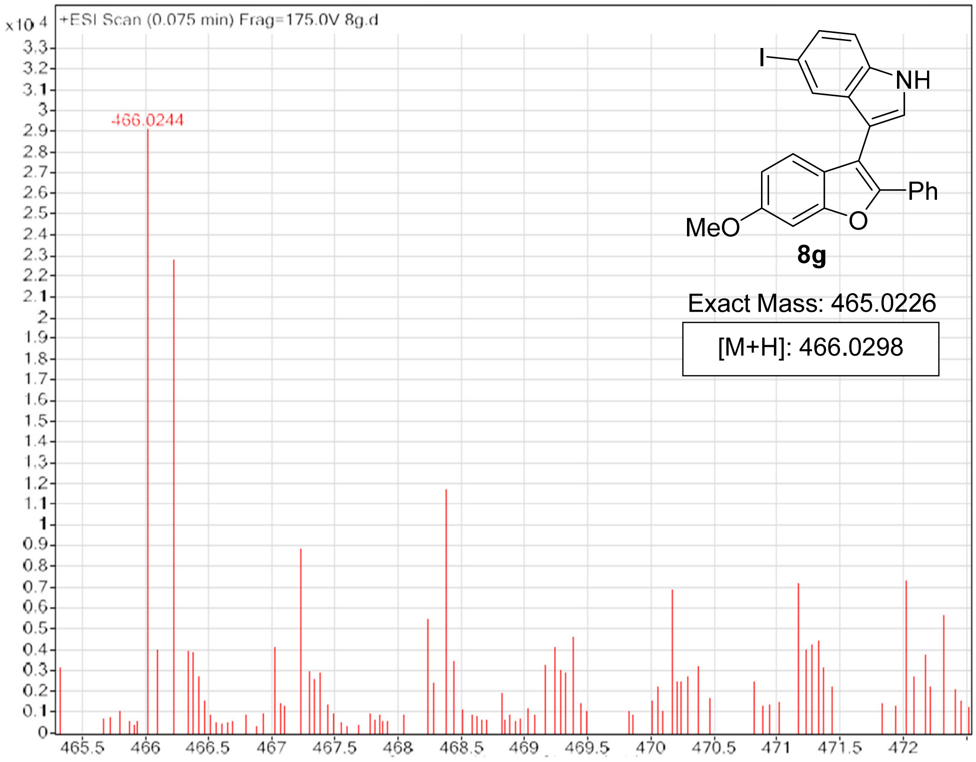

Data Filename 8h.d ACQ Method 506k_leesunhee.m Comment Acquired Time 5/19/2023 5:06:34 PM

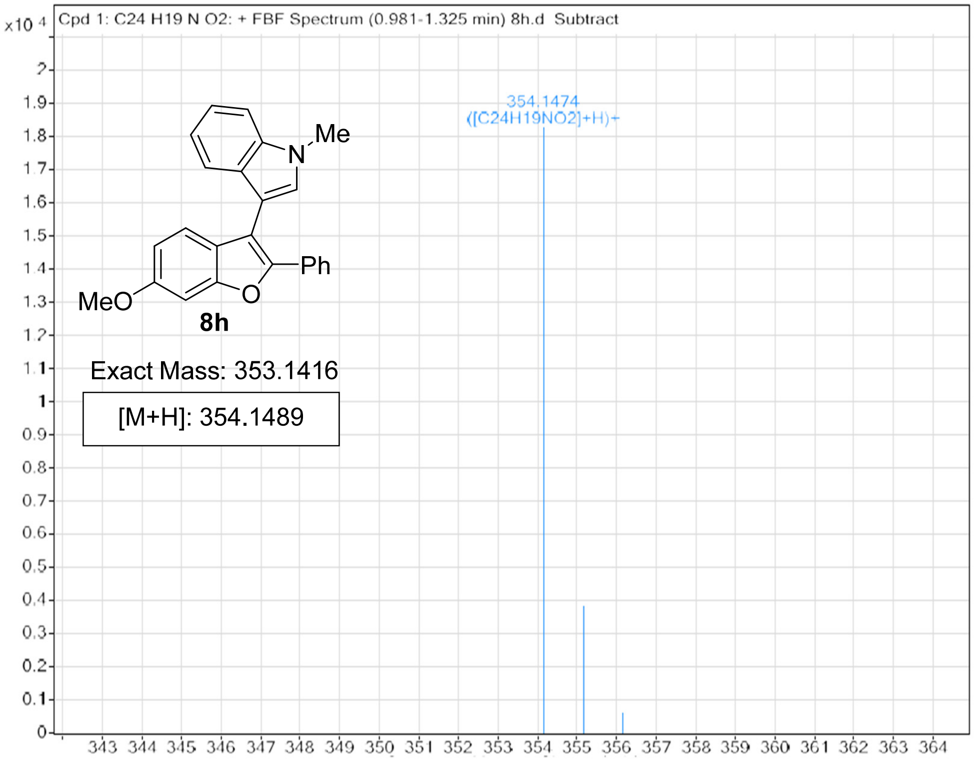

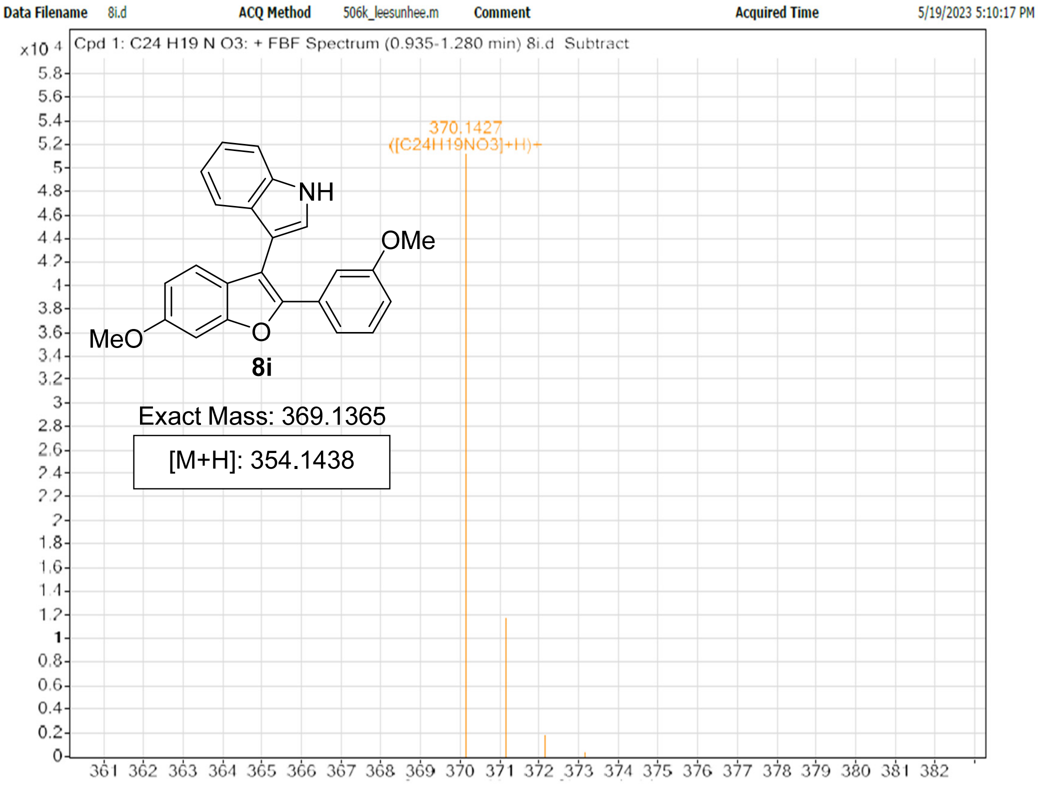

Data Filename 8i.d
ACQ Method 506k_leesunhee.m
Comment
Acquired Time 5/19/2023 5:10:17 PM
Cpd 1: C24 H19 N O3: + FBF Spectrum (0.935-1.280 min) 8i.d Subtract
370.1427
([C24H19NO3]+H)+
NH
OMe
MeO
O
8i
Exact Mass: 369.1365
[M+H]: 354.1438

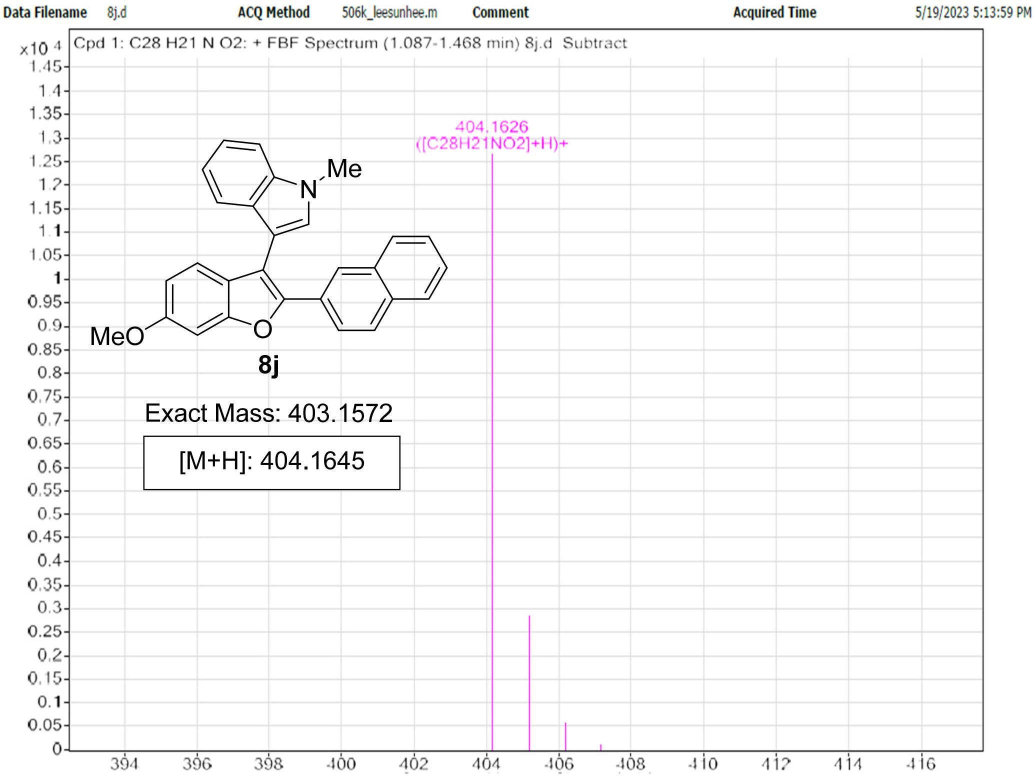

Data Filename 8j.d
ACQ Method 506k_leesunhee.m
Comment
Acquired Time 5/19/2023 5:13:59 PM
Cpd 1: C28 H21 N O2: + FBF Spectrum (1.087-1.468 min) 8j.d Subtract
404.1626
([C28H21NO2]+H)+
N
Me
MeO
O
8j
Exact Mass: 403.1572
[M+H]: 404.1645

Data Filename 8k.d ACQ Method 506k_leesunhee.m_A2. Comment Acquired Time 1/29/2024 2:59:05 PM

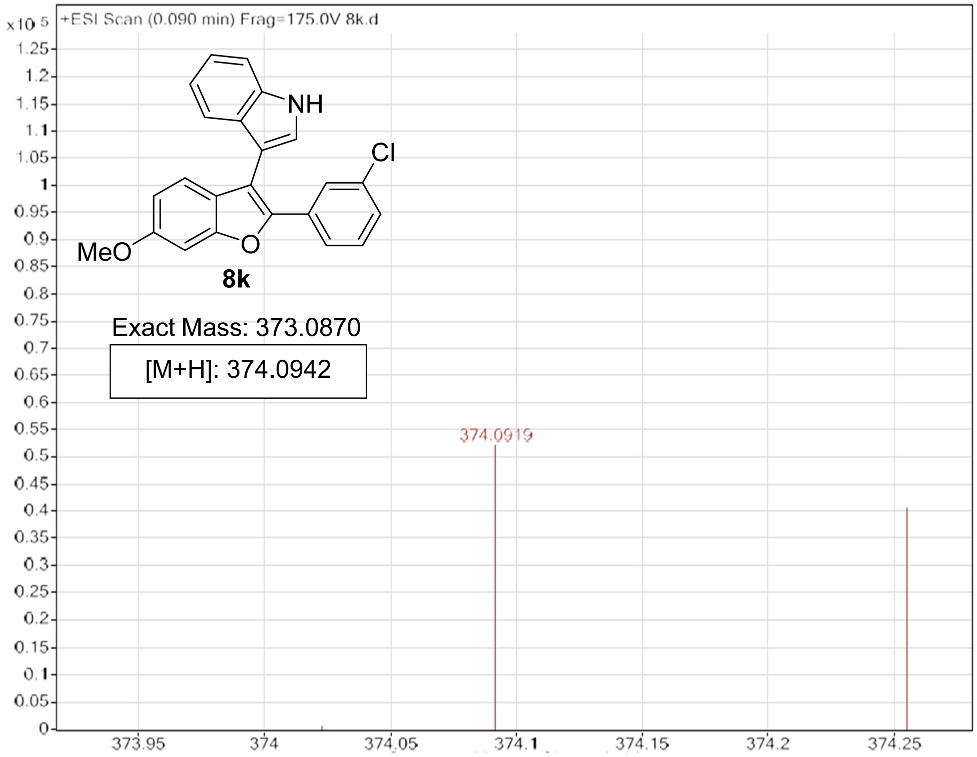

Data Filename 8l.d ACQ Method 506k_leesunhee.m_A2. Comment Acquired Time 1/29/2024 3:02:49 PM

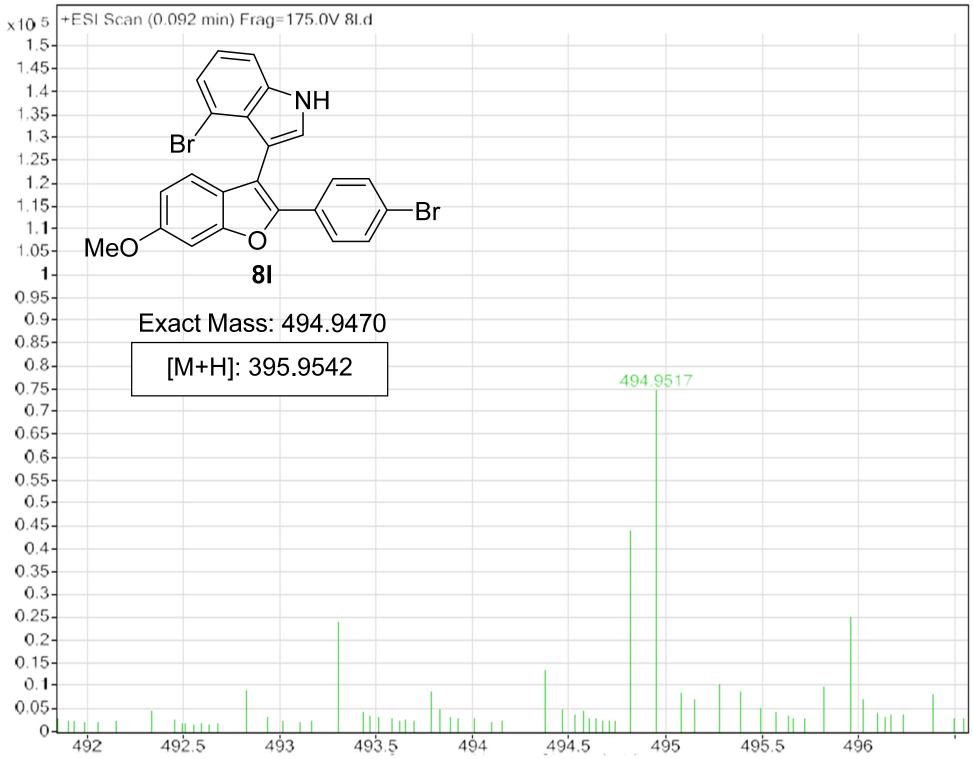

Data Filename 8n.d ACQ Method 506k_leesunhee.m Comment Acquired Time 5/19/2023 5:28:49 PM

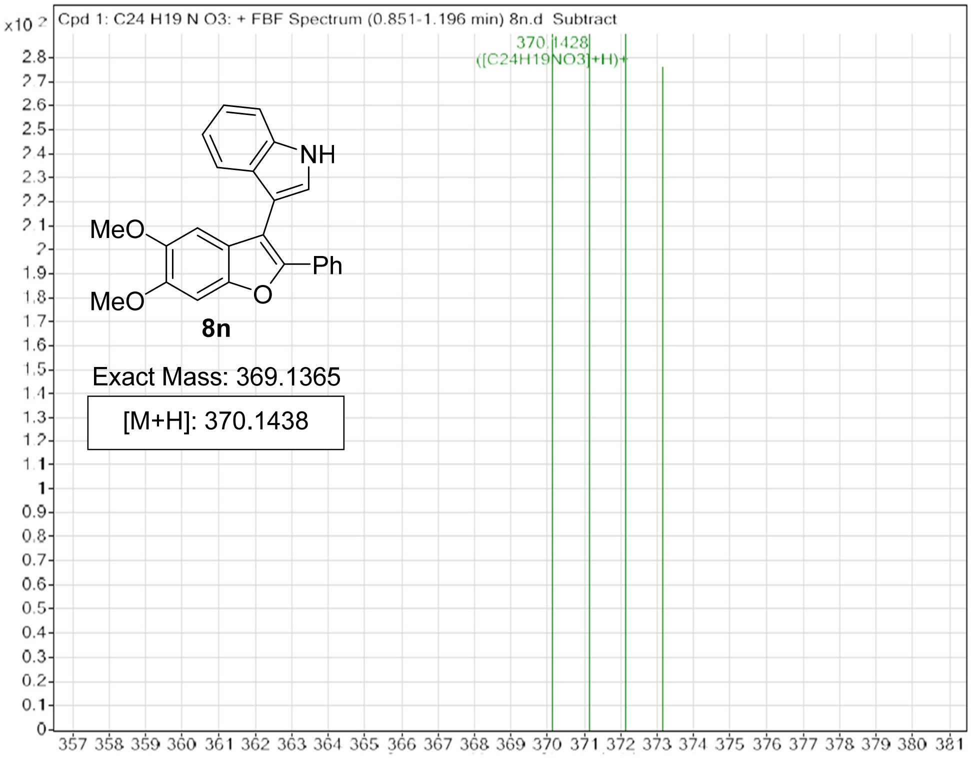

Data Filename 8o.d ACQ Method Comment Sample information is unavailable Acquired Time Unavailable

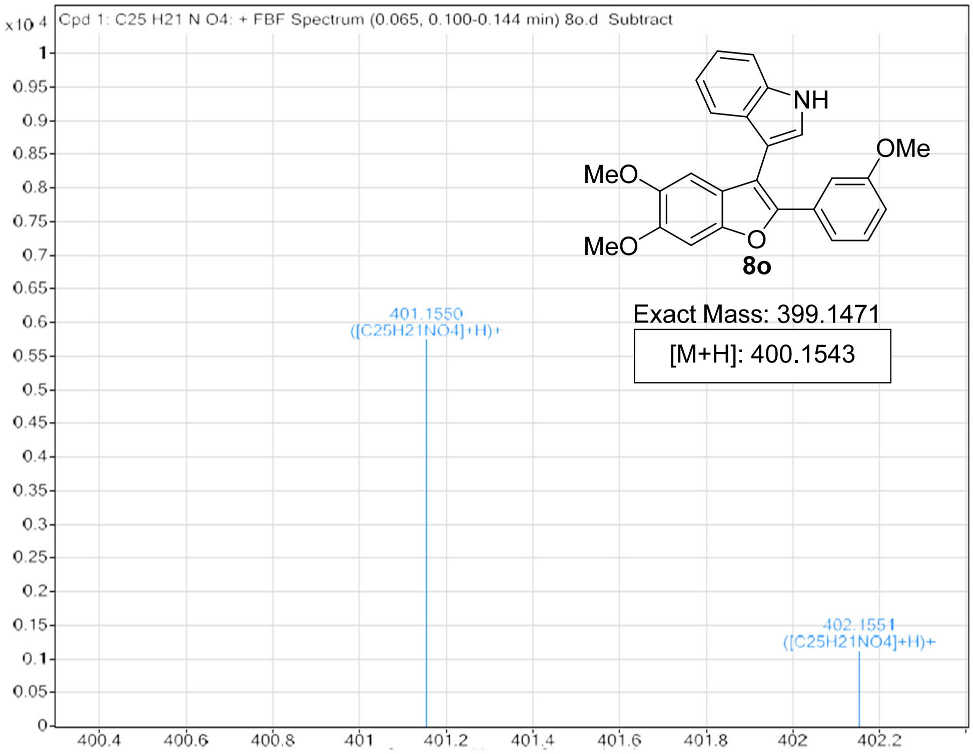

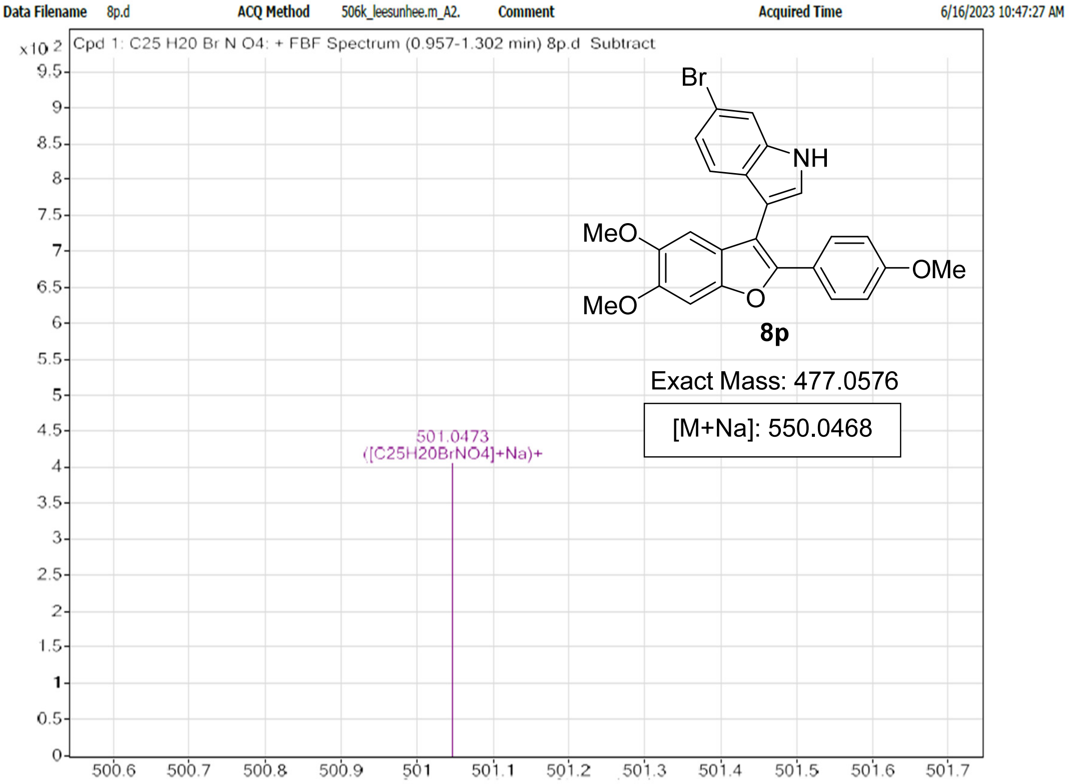
Data Filename 8p.d ACQ Method 506k_leesunhee.m_A2. Comment Acquired Time 6/16/2023 10:47:27 AM
x10 2
Cpd 1: C25 H20 Br N O4: + FBF Spectrum (0.957-1.302 min) 8p.d Subtract
Br
NH
MeO
MeO
O
OMe
8p
Exact Mass: 477.0576
[M+Na]: 550.0468
501.0473
([C25H20BrNO4]+Na)+
500.6 500.7 500.8 500.9 501 501.1 501.2 501.3 501.4 501.5 501.6 501.7

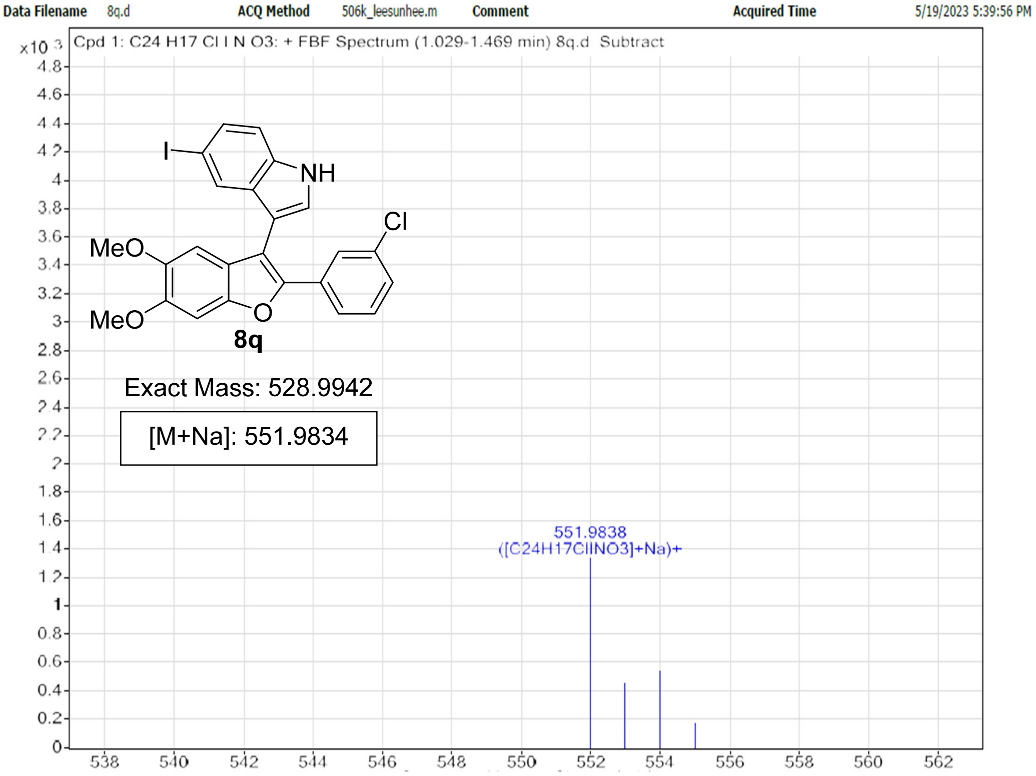
Data Filename 8q.d ACQ Method 506k_leesunhee.m Comment Acquired Time 5/19/2023 5:39:56 PM
x10 3
Cpd 1: C24 H17 Cl I N O3: + FBF Spectrum (1.029-1.469 min) 8q.d Subtract
I
NH
Cl
MeO
MeO
O
8q
Exact Mass: 528.9942
[M+Na]: 551.9834
551.9838
([C24H17ClINO3]+Na)+
538 540 542 544 546 548 550 552 554 556 558 560 562

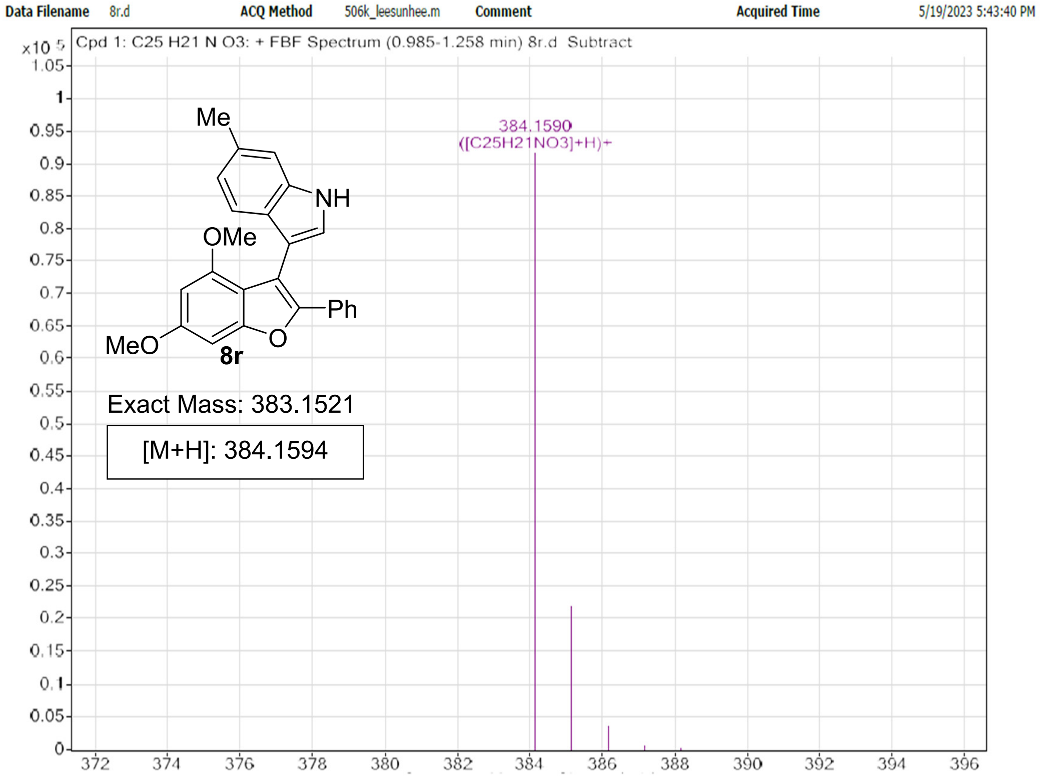
Data Filename 8r.d
ACQ Method 506k_leesunhee.m
Comment
Acquired Time 5/19/2023 5:43:40 PM
Cpd 1: C25 H21 N O3: + FBF Spectrum (0.985-1.258 min) 8r.d Subtract
x10 5
384.1590
([C25H21NO3]+H)+
Me
NH
OMe
Ph
MeO
O
8r
Exact Mass: 383.1521
[M+H]: 384.1594

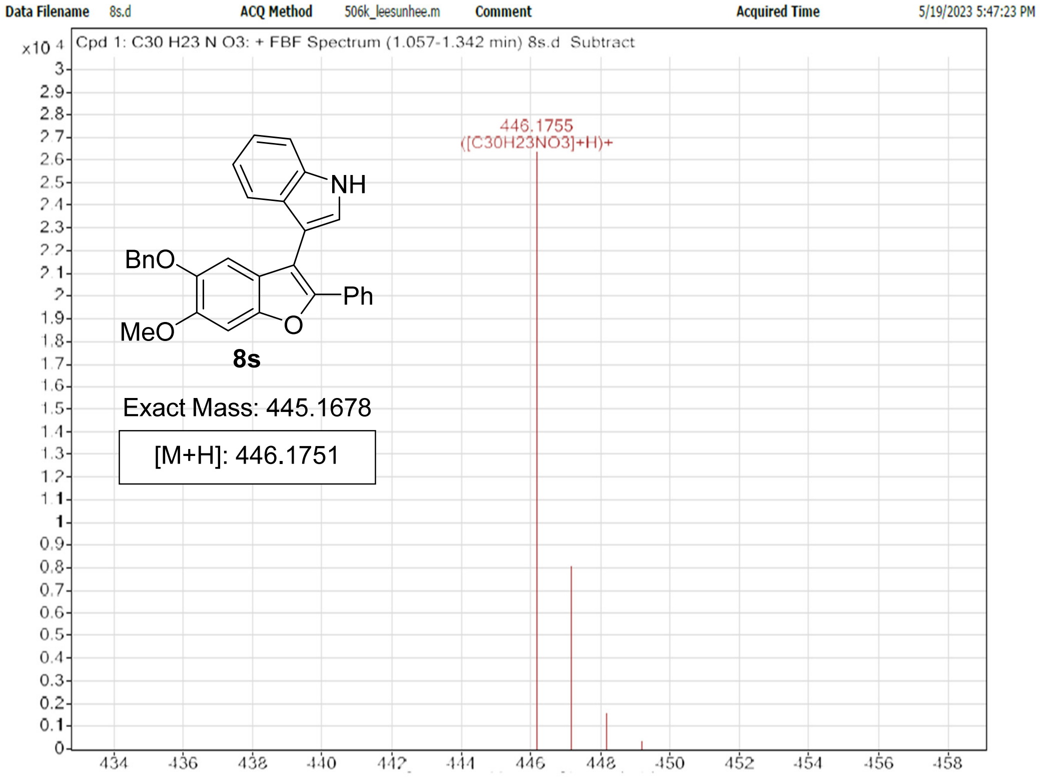
Data Filename 8s.d
ACQ Method 506k_leesunhee.m
Comment
Acquired Time 5/19/2023 5:47:23 PM
Cpd 1: C30 H23 N O3: + FBF Spectrum (1.057-1.342 min) 8s.d Subtract
x10 4
446.1755
([C30H23NO3]+H)+
NH
BnO
Ph
MeO
O
8s
Exact Mass: 445.1678
[M+H]: 446.1751

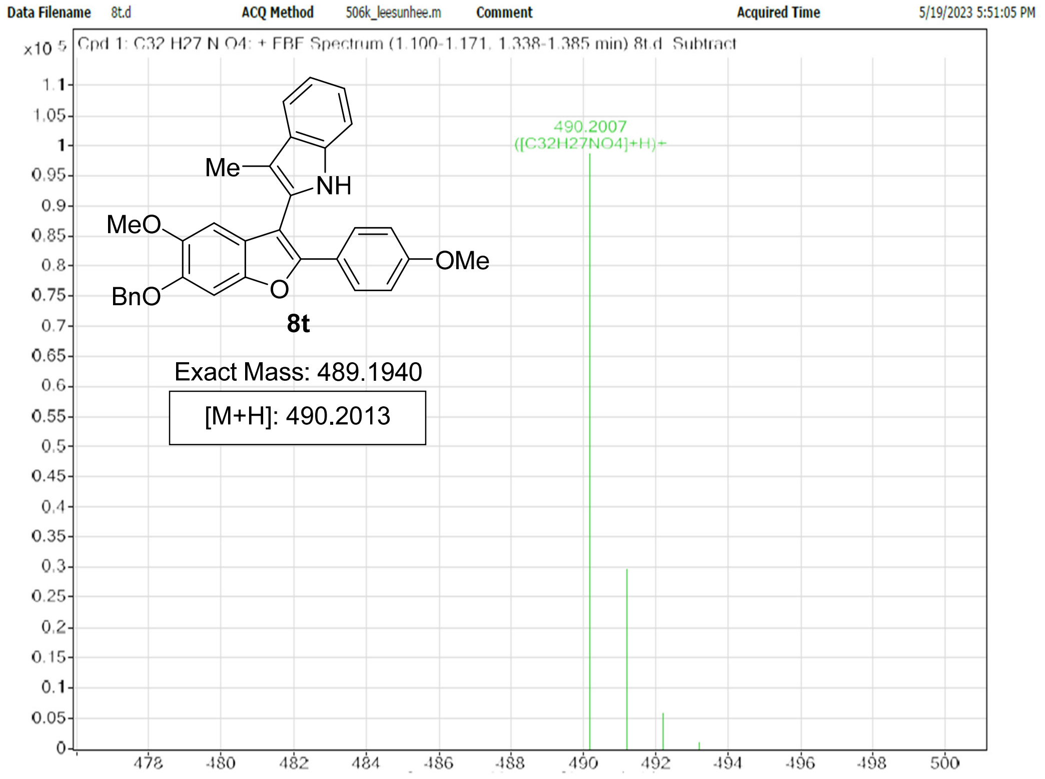

Data Filename 8t.d
ACQ Method 506k_leesunhee.m
Comment
Acquired Time 5/19/2023 5:51:05 PM
Cpd 1: C32 H27 N O4: + FBF Spectrum (1.100-1.171, 1.338-1.385 min) 8t.d Subtract
490.2007
([C32H27NO4]+H)+
Me
NH
MeO
BnO
O
OMe
8t
Exact Mass: 489.1940
[M+H]: 490.2013

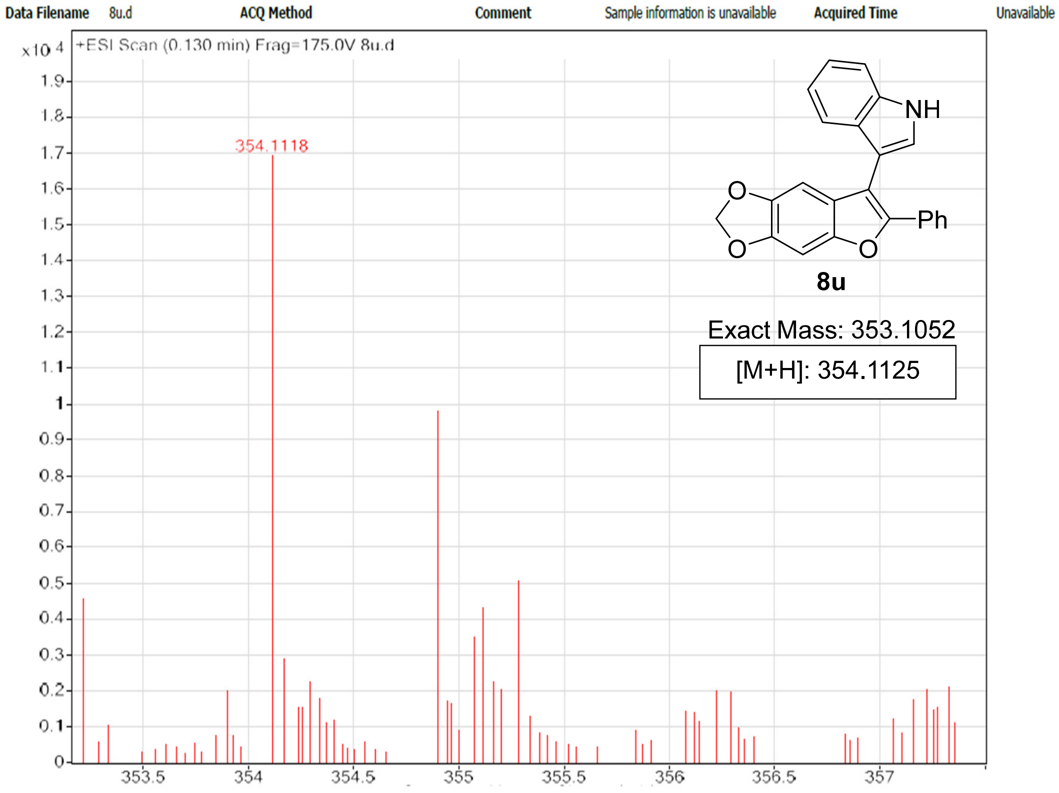

Data Filename 8u.d
ACQ Method
Comment
Sample information is unavailable
Acquired Time
Unavailable
+ESI Scan (0.130 min) Frag=175.0V 8u.d
354.1118
NH
O
O
O
Ph
8u
Exact Mass: 353.1052
[M+H]: 354.1125

Data Filename v.d ACQ Method 506k_leesunhee.m_A2. Comment Acquired Time 7/13/2023 5:57:48 PM

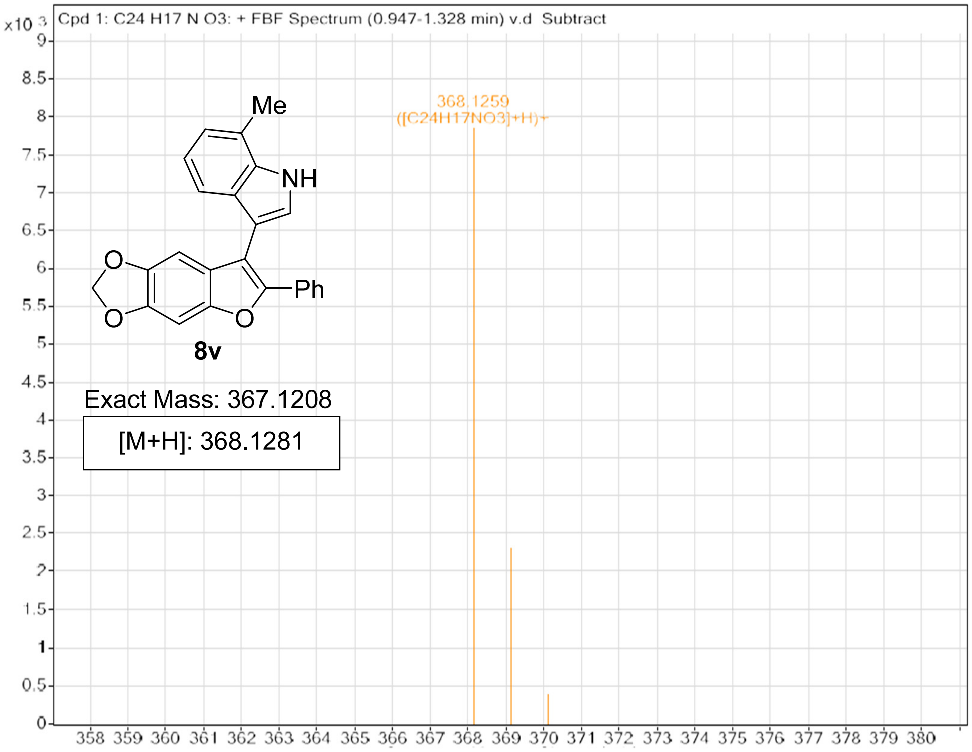

Data Filename 8w.d ACQ Method 506k_leesunhee.m Comment Acquired Time 5/19/2023 6:02:12 PM

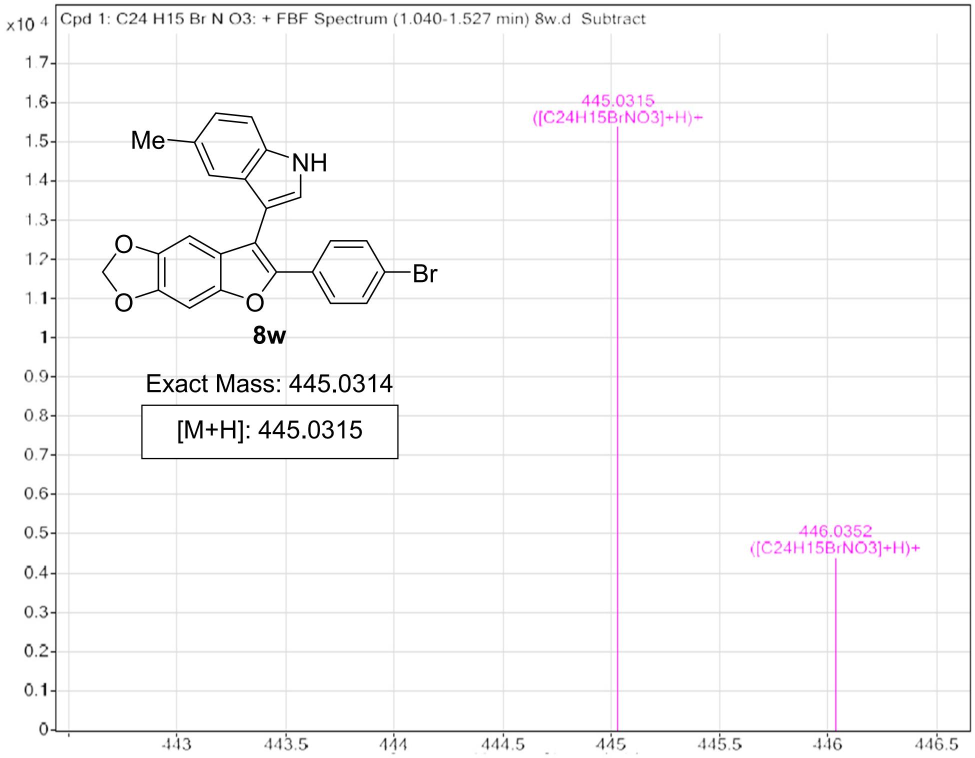

Data Filename x.d ACQ Method 506k_leesunhee.m_A2. Comment Acquired Time 7/13/2023 6:05:14 PM

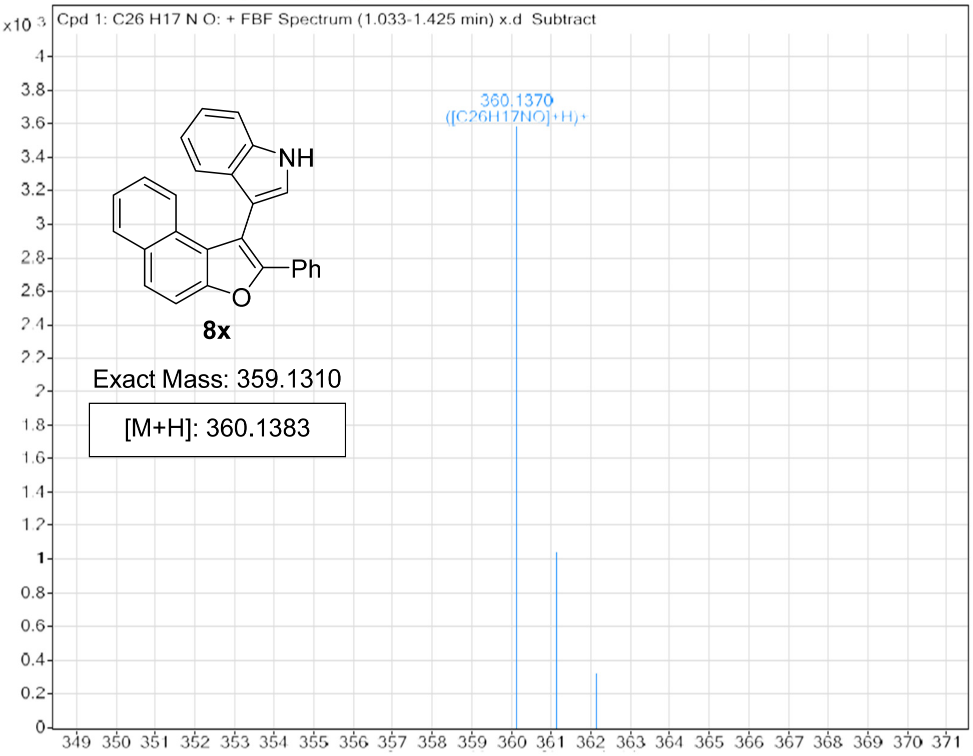

Data Filename 8y.d ACQ Method 506k_leesunhee.m_A2. Comment Acquired Time 1/29/2024 3:21:23 PM

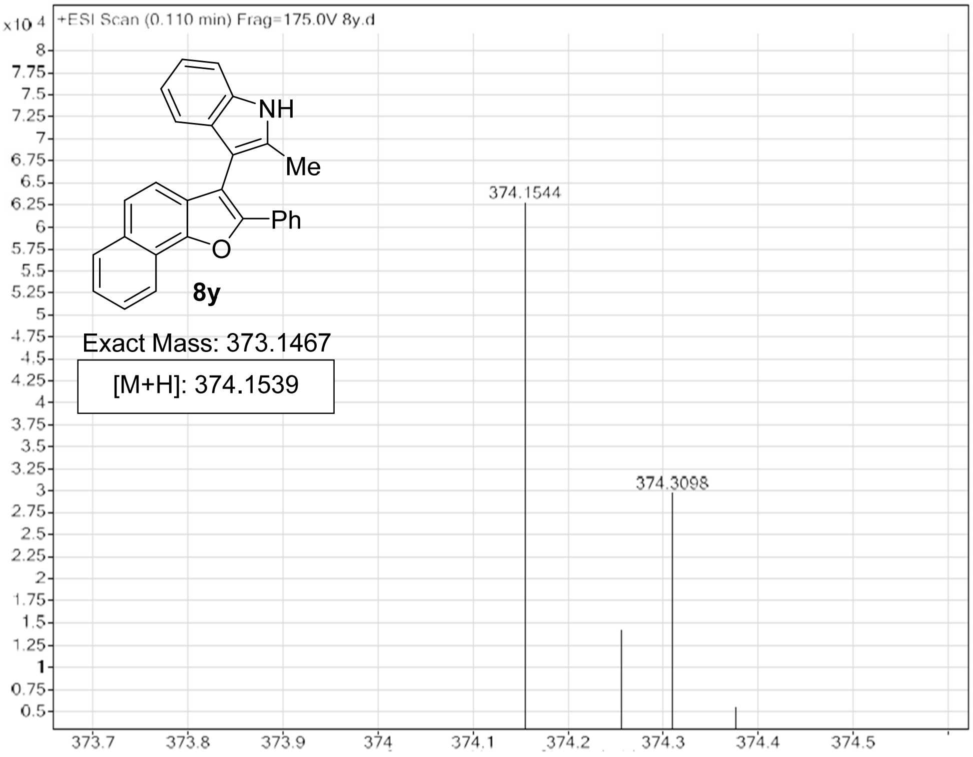

Data Filename 8z.d ACQ Method 506k_leesunhee.m_A2. Comment Acquired Time 1/29/2024 3:25:08 PM

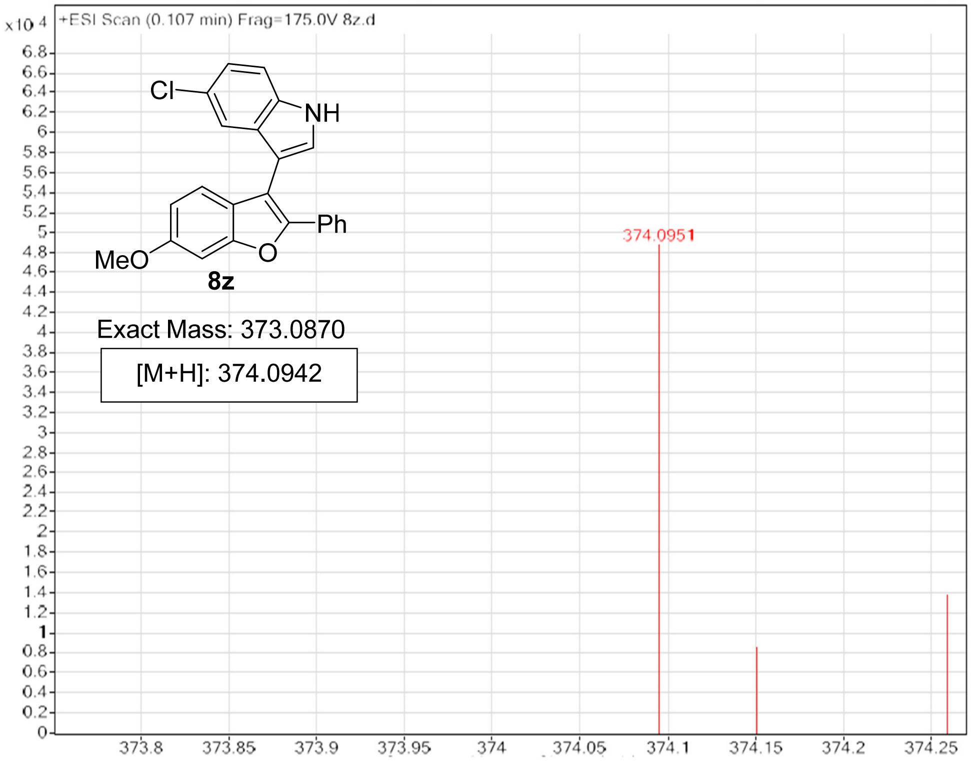

Data Filename 8aa.d ACQ Method 506k_leesunhee.m_A2. Comment Acquired Time 6/23/2023 11:29:32 AM

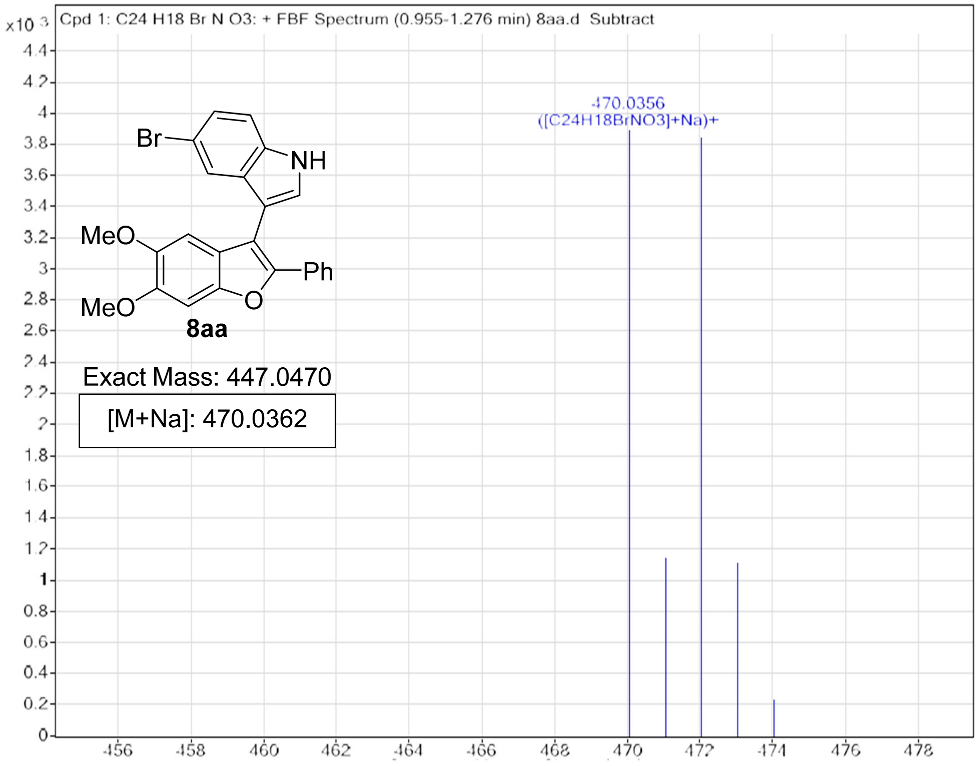

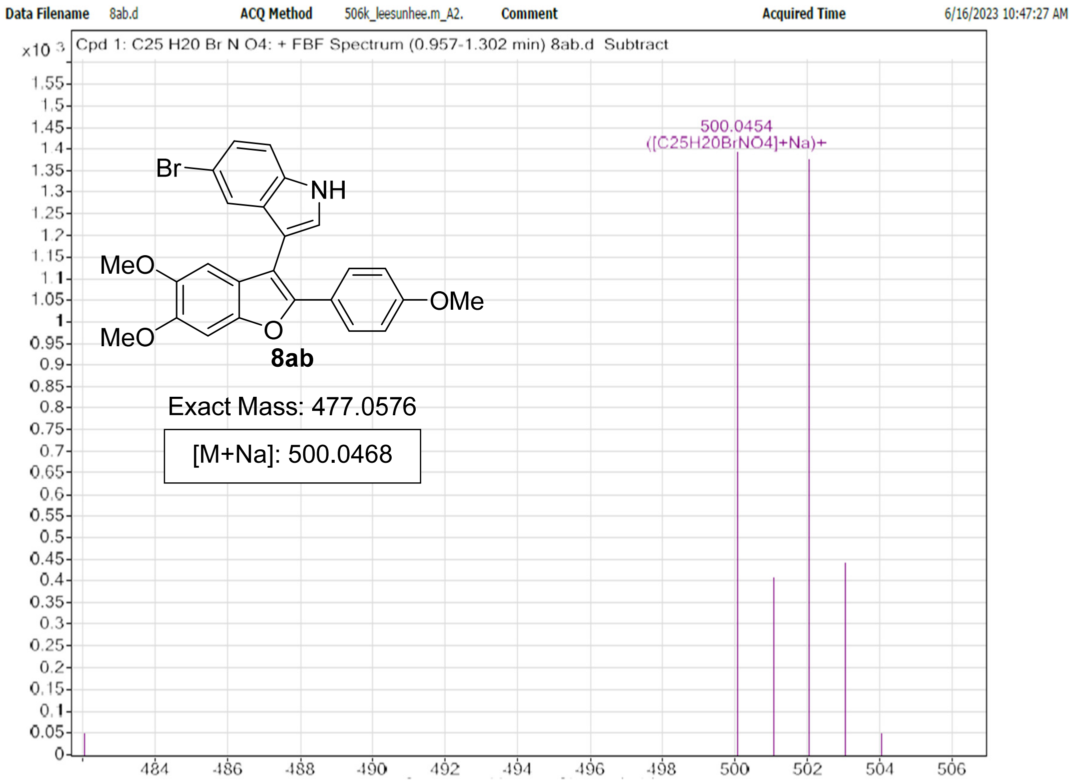
Data Filename 8ab.d
ACQ Method 506k_leesunhee.m_A2.
Comment
Acquired Time 6/16/2023 10:47:27 AM
x10 3
Cpd 1: C25 H20 Br N O4: + FBF Spectrum (0.957-1.302 min) 8ab.d Subtract
500.0454
([C25H20BrNO4]+Na)+
Br
NH
MeO
MeO
O
OMe
8ab
Exact Mass: 477.0576
[M+Na]: 500.0468

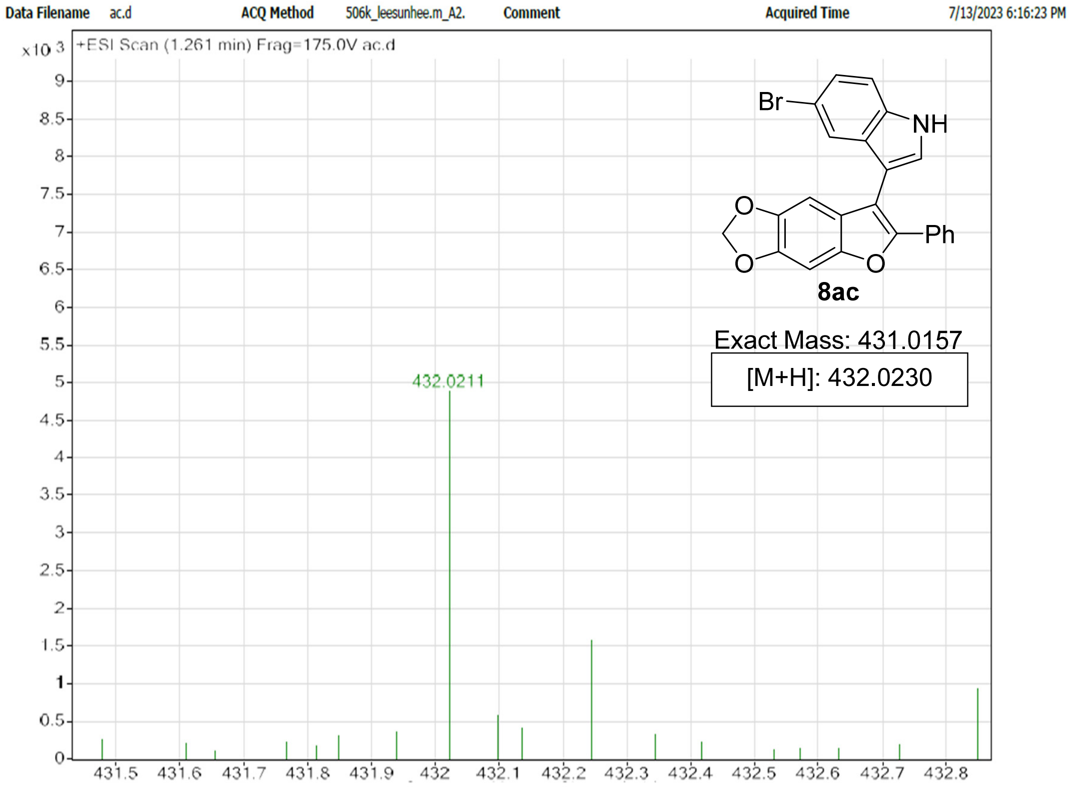
Data Filename ac.d
ACQ Method 506k_leesunhee.m_A2.
Comment
Acquired Time 7/13/2023 6:16:23 PM
x10 3
+ESI Scan (1.261 min) Frag=175.0V ac.d
432.0211
Br
NH
O
O
O
Ph
8ac
Exact Mass: 431.0157
[M+H]: 432.0230

Data Filename ad.d ACQ Method 506k_leesunhee.m_A2. Comment Acquired Time 7/13/2023 6:20:07 PM

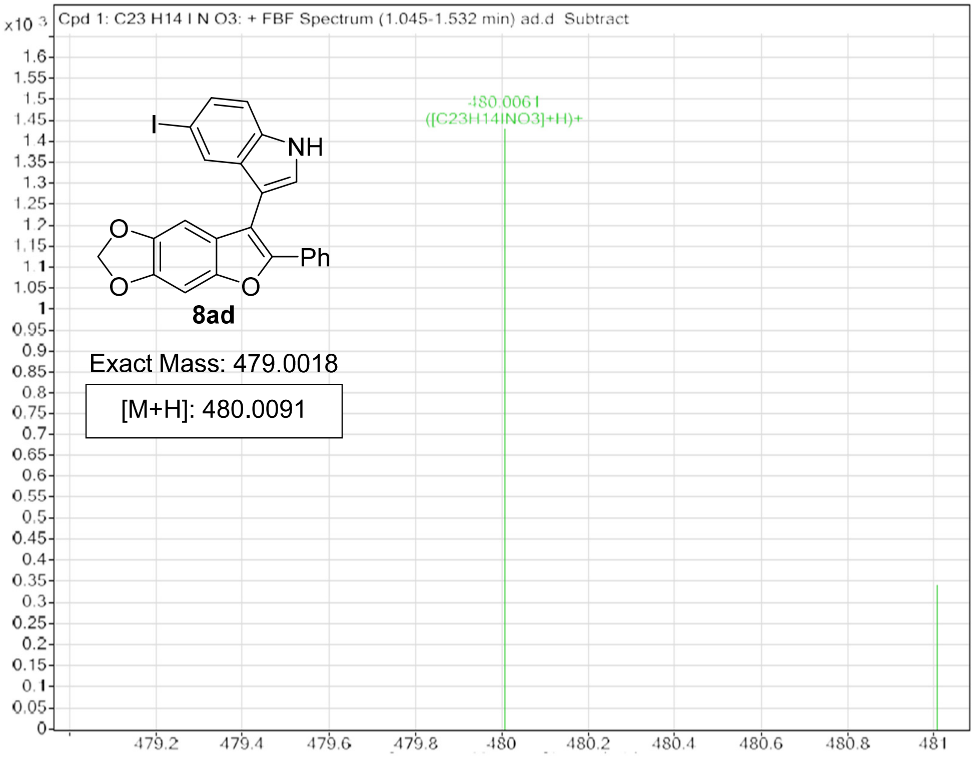

Purity of 8aa measured by HPLC

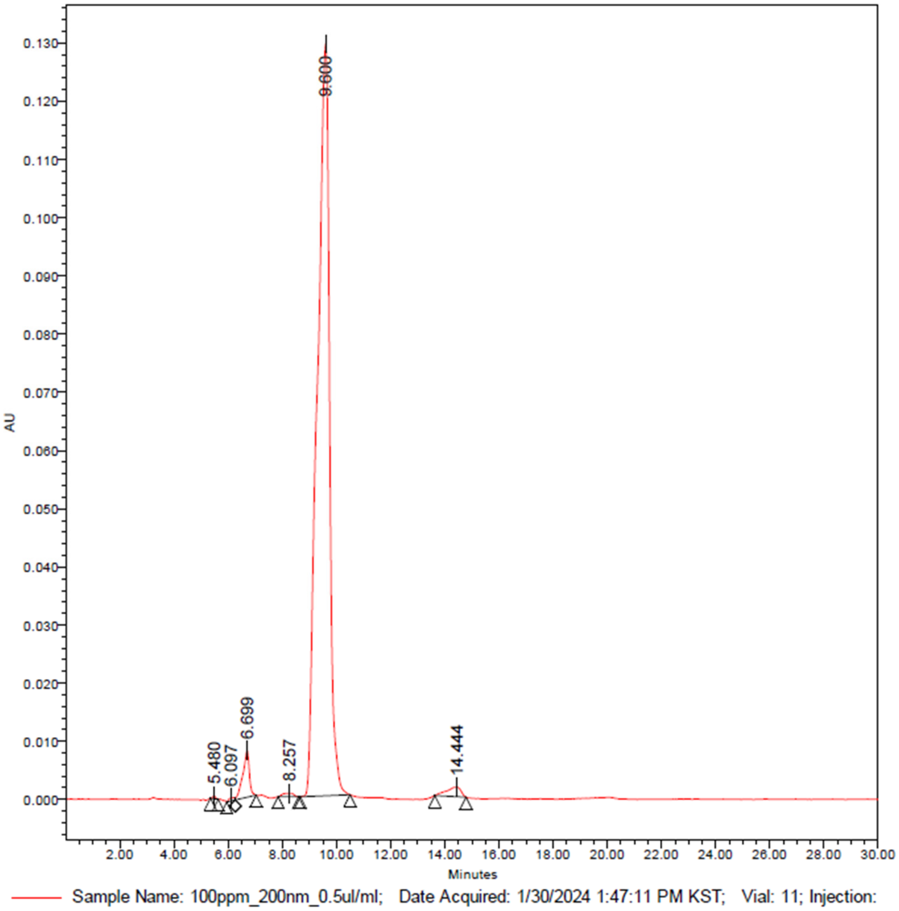

Peak Summary with Statistics
Name:

	Sample Name	Vial	Inj	Retention Time (min)	Area	% Area	Height
1	100ppm_200nm_0.5ul/ml	11	1	5.480	3532	0.08	510
2	100ppm_200nm_0.5ul/ml	11	1	6.097	7370	0.17	497
3	100ppm_200nm_0.5ul/ml	11	1	14.444	60905	1.44	1617
4	100ppm_200nm_0.5ul/ml	11	1	8.257	16691	0.39	601
5	100ppm_200nm_0.5ul/ml	11	1	9.600	4013420	94.75	129276
6	100ppm_200nm_0.5ul/ml	11	1	6.699	134010	3.16	8022
Mean				8.429			
Std. Dev.				3.308			
% RSD				39.24			

8aa HPLC conditions	
Column	CHIRALPAK® C18 UG 5 μm, 4.6 mm i.d. × 250 mm
Mobile phase	Acetonitrile : Water = 8 : 2
Flow rate	0.5 mL/min
Injection volume	10 μL
Detection	254 nm
Column Temperature	25 °C
Auto-sampler temperature	25 °C
Retention time	9.6 minutes
